# Comparative proteomics of root plasma membrane proteins reveals the involvement of calcium signalling in NaCl-facilitated nitrate uptake in *Salicornia europaea*

**DOI:** 10.1093/jxb/erv216

**Published:** 2015-05-08

**Authors:** Lingling Nie, Juanjuan Feng, Pengxiang Fan, Xianyang Chen, Jie Guo, Sulian Lv, Hexigeduleng Bao, Weitao Jia, Fang Tai, Ping Jiang, Jinhui Wang, Yinxin Li

**Affiliations:** ^1^Key Laboratory of Plant Molecular Physiology, Institute of Botany, Chinese Academy of Sciences, Beijing 100093, PR China; ^2^Department of Biochemistry and Molecular Biology, Michigan State University, 603 Wilson Road, East Lansing, MI 48824, USA; ^3^Shanghai Center for Plant Stress Biology (PSC), Chinese Academy of Sciences, No. 3888 Chenhua Road, Songjiang District, Shanghai 201602, PR China

**Keywords:** [Ca^2+^]_cyt_, calcium signalling, 2D-DIGE, NaCl, nitrate uptake, plasma membrane, *Salicornia europaea.*

## Abstract

Improving crop nitrogen (N) use efficiency under salinity is essential for the development of sustainable agriculture in marginal lands. *Salicornia europaea* is a succulent euhalophyte that can survive under high salinity and N-deficient habitat conditions, implying that a special N assimilation mechanism may exist in this plant. In this study, phenotypic and physiological changes of *S. europaea* were investigated under different nitrate and NaCl levels. The results showed that NaCl had a synergetic effect with nitrate on the growth of *S. europaea.* In addition, the shoot nitrate concentration and nitrate uptake rate of *S. europaea* were increased by NaCl treatment under both low N and high N conditions, suggesting that nitrate uptake in *S. europaea* was NaCl facilitated. Comparative proteomic analysis of root plasma membrane (PM) proteins revealed 81 proteins, whose abundance changed significantly in response to NaCl and nitrate. These proteins are involved in metabolism, cell signalling, transport, protein folding, membrane trafficking, and cell structure. Among them, eight proteins were calcium signalling components, and the accumulation of seven of the above-mentioned proteins was significantly elevated by NaCl treatment. Furthermore, cytosolic Ca^2+^ concentration ([Ca^2+^]_cyt_) was significantly elevated in *S. europaea* under NaCl treatment. The application of the Ca^2+^ channel blocker LaCl_3_ not only caused a decrease in nitrate uptake rate, but also attenuated the promoting effects of NaCl on nitrate uptake rates. Based on these results, a possible regulatory network of NaCl-facilitated nitrate uptake in *S. europaea* focusing on the involvement of Ca^2+^ signalling was proposed.

## Highlight

Unlike in glycophytes, NaCl facilitates the nitrate uptake in the euhalophyte *S. europaea*, while calcium signalling plays important roles in this process.

## Introduction

Salinity is among the most severe abiotic stresses in agriculture that affects ~6% of the total land on earth (Munns and Tester, 2008). In saline soil, the salt concentration is high, whereas the nitrogen (N) content is usually deficient (Hamed *et al.*, 2013); most crops could not survive on it. However, many halophytes can thrive in this type of infertile soil, suggesting that they may have specific N uptake and utilization mechanisms. *Salicornia europaea* is one of the most salt-tolerant plant species in the world (Ungar, 1987). A previous study demonstrated that *S. europaea* has a strong ability to utilize N; this plant can thrive under both N-limited and high N conditions (Nie *et al.*, 2012). However, the effect of NaCl on N utilization has not been investigated in *S. europaea*. Elucidating the regulatory networks of efficient N uptake of *S. europaea* under salinity would be helpful for creating salt-tolerant crops with high N use efficiency, which is essential for the development of sustainable agriculture in marginal lands.

N is an essential macronutrient for plant growth. Among various N forms (nitrate, ammonium, amino acids, and peptides), nitrate (NO_3_^–^) is the predominant form available in aerobic soils (Marschner and Rimmington, 1988). Salinity can severely influence nitrate assimilation in plants. Generally, a high Cl^–^ concentration acts as an antagonist and represses nitrate uptake; thus, NaCl negatively affects nitrate uptake, assimilation, and protein synthesis which may be responsible, at least in part, for the depressed plant growth under saline conditions (Bar *et al.*, 1997). In addition, the state of the membrane and/or membrane proteins also affects uptake of NO_3_^–^ by altering plasma membrane (PM) integrity under salinity (Frechilla *et al.*, 2001). However, in halophytic *Distichlis spicata* (Pessarakli *et al.*, 2012), NaCl has no negative effect on N uptake. Another report on the euhalophyte *Suaeda physophora* showed that NaCl application significantly increased leaf NO_3_^−^ concentration under N-sufficient conditions (Yuan *et al.*, 2010). In fact, sodium-dependent NO_3_^−^ uptake has been reported in the marine diatom *Phaeodactylum tricornutum* (Rees *et al.* 1980), cyanobacteria (Lara *et al.* 1993), and the marine halophyte *Zostera marina* (Rubio *et al.*, 2005). These results indicated that NaCl may have a promoting effect on nitrate uptake in some halophytes, which is different from glycophytes, whereas the underlying molecular networks are still unclear.

Nitrate uptake occurs in the outer cell layers of roots and relies on nitrate transporters, which are mainly localized at the PM together with the regulatory proteins. To date, four families of nitrate transporters have been identified: nitrate transporter 1/peptide transporter family (NRT1/PTR), nitrate transporter 2 family (NRT2), chloride channel family (CLC), and slow anion channel-associated homologues (SLAC1/SLAH) (Krapp *et al.*, 2014). More than 60 nitrate transporters have been estimated in *Arabidopsis*, the regulation of which involves complex networks that are not fully understood. Investigations on root PM protein accumulation under different NaCl and nitrate levels would significantly enhance our knowledge of nitrate signalling under salinity. Proteomics is a powerful tool to study PM proteins depending on two main approaches, namely gel-free and two-dimensional electrophoresis (2-DE) (Nouri and Komatsu, 2010). The gel-free method has better coverage and higher solubility for hydrophobic membrane proteins than 2-DE. However, proteins containing a single transmembrane domain or peripheral membrane-associated proteins can be more readily resolved in 2-DE. In addition, 2-DE can give information on post-translational modifications (Tang, 2012) and it is also a valuable tool in deciphering N-terminal processing of proteins (Taylor *et al.*, 2011). Nouri and Komastu (2010) used both gel-free and 2-DE methods to study soybean PM proteins, and concluded that the two techniques are complementary for comparative analysis. Meanwhile, Tang *et al.* (2008) used two-dimensional fluorescence difference gel electrophoresis (2D-DIGE) to study *Arabidopsis* PM proteins, and three homologous brasinosteroid-signalling kinases were successfully identified; thus, 2D-DIGE is demonstrated to be a powerful approach for studying signalling proteins localized in the PM.

Proteomic research on glycophytes has focused on PM proteins under salt stress (Cheng *et al.*, 2009). Meanwhile, in a few studies, whole-plant proteomics have been applied in *Arabidopsis* (Wang *et al.*, 2012), *Zea mays* (Prinsi *et al.*, 2009), and barley (Moller *et al.*, 2011) to detect their responses to different N conditions. However, no research has been performed to analyse the PM proteome of halophytes under different nitrate and NaCl levels. The aim of this study was to investigate the characteristics of nitrate uptake in *S. europaea* under NaCl treatment and to identify the underlying regulatory components. Through physiological analysis, it was demonstrated that in contrast to the situation in glycophytes, NaCl facilitates nitrate uptake in *S. europaea*. Comparative proteomics and cell biology studies further revealed that calcium signalling plays important roles in this process. These results open up exciting perspectives for further investigations on the specific regulatory pathways involved in efficient N uptake under NaCl conditions.

## Materials and methods

### Plant materials and growth conditions

*Salicornia europaea* seeds were collected from the coastal area of Dafeng City, Jiangsu Province, China. The plants were grown in a greenhouse with a day/night temperature regime of 25 °C/20 °C, photoperiod of 16h, and relative humidity of ~60%. At 20 d after sowing on perlite granules, seedlings were irrigated with four solutions containing different concentrations of NO_3_^–^ and NaCl in modified 1/2 Hoagland solution for 30 d. Then the plants were harvested and washed with distilled water to remove the surface ions and used for further analysis. The modified 1/2 Hoagland solution contained 0.5mM KH_2_PO_4_, 1mM MgSO_4_, 2.5mM CaCl_2_, 0.05mM Fe-EDTA, 2.5mM KCl, and micronutrients, with the pH adjusted to 6.5±0.1. The four treatments were 0.1mM NO_3_^–^ (LN), 0.1mM NO_3_^–^+200mM NaCl (LN+S), 10mM NO_3_^–^ (HN), and 10mM NO_3_^–^+200mM NaCl (HN+S).

#### Measurement of fresh weight (FW), dry weight (DW), root length, and root volume

FW was measured immediately after harvesting. DW was measured after drying for 48h in an oven at 80 °C. Roots were scanned with a digital scanner (Epson, Nagano, Japan) and then analysed with WinRHIZO software (Regent Instruments Inc., Quebec, Canada).

#### Quantification of total Na, total N contents, and NO_3_^–^ concentrations

Plants were dried for 72h in an oven at 80 °C and then weighted and subsequently ground into powder in a mixer mill (Retsch MM400, Hann, Germany). The powder was digested with a mixture of nitric acid and hydrogen peroxide using a microwave system (MARS; CEM Corporation, Matthews, NC, USA), which was used to determine total Na contents by atomic emission spectrometry (ICP-AES, Thermo, Waltham, MA, USA). N contents were determined by a Vario EL III CHNOS Elemental Analyzer (Elementar Analysensysteme GmbH, Germany). The NO_3_^–^ concentration was measured as previously described (Jampeetong and Brix, 2009). Dried material (5–10mg) was extracted with 10ml of Milli Q water at 80 °C in a water bath for 20min. Then, NO_3_^–^ concentrations were analysed by an AA3 continuous flow-analyser (Seal Analytical, Germany).

#### Analysis of nitrate reductase (NR) activities

NR activities were measured as previously described (Yaneva *et al.*, 2002) with some modifications. The seedlings were homogenized in extraction buffer [50mM HEPES-KOH, pH 7.5, 1mM EDTA, 10mM glutathione (GSH), 0.1% (w/v) polyvinylpyrrolidone (PVP), 1mM dithiothreitol (DTT), and 1mM phenylmethylsulphonyl fluoride (PMSF)] and centrifuged for 20min at 12 000 *g*. The supernatant was incubated with reaction buffer (50mM HEPES-KOH, pH 7.5, 5mM KNO_3_, 0.2mM NADH) in the dark at 25 °C for 20min; the reaction was terminated by addition of 1% sulphanylamide in 1.5M HCl and 0.01% *N*-(1-naphthyl)-ethylenediammonium dichloride. Nitrite production was determined by reading the absorbance at 540nm.

#### Analysis of nitrate uptake rates

Nitrate uptake rates were measured as previously described (Muños *et al.*, 2004). *Salicornia europaea* plants of ~30 d old irrigated with 1/2 Hoagland solution plus 200mM NaCl were transferred to 0.1mM CaSO_4_ for 1min and then to four solutions containing different NaCl and K^15^NO_3_ (99% atom excess ^15^N) concentrations, as follows: 0.1mM K^15^NO_3_, 0.1mM K^15^NO_3_+200mM NaCl, 10mM K^15^NO_3_, and 10mM K^15^NO_3_+200mM NaCl for 15min, and finally, to 0.1mM CaSO_4_ for 1min. Six roots for each treatment and three independent biological replications were dried at 70 °C for 48h and ground. The powder (2–3mg) was used for ^15^N determination by the Delta Plus-MS system (Thermo).

Thirty-day-old *S. europaea* plants irrigated with 1/2 Hoagland solution plus 200mM NaCl were pre-treated with 1mM of the non-specific Ca^2+^ channel blocker LaCl_3_ for 24h. The LaCl_3_-pre-treated and control plants were used for ^15^NO_3_^–^ uptake assay following the above-mentioned method.

#### Plasma membrane (PM) isolation

PMs were isolated as previously reported (Tang, 2012). Roots were homogenized in grinding buffer (pH 7.5) (25mM HEPES, 0.33M sucrose, 10% glycerol, 0.6% PVP, 5mM ascorbic acid, 5mM EDTA, 5mM DTT, and 1mM PMSF). The homogenate was filtered through Miracloth and centrifuged at 10 000 *g* for 15min. Total microsomal (TM) fractions were pelleted by centrifugation at 80 000 *g* for 1h and resuspended in suspension buffer [5mM KH_2_PO_4_/K_2_HPO_4_ buffer (pH 7.8), 0.33mM sucrose, 3mM KCl, 1mM DTT, and 1mM protease cocktail]. Two-phase partitioning was performed by using a solution containing 6.2% polyethylene glycol (PEG) 3350, 6.2% dextran T-500, 0.33M sucrose, 3mM KCl, and 5mM KH_2_PO_4_/K_2_HPO_4_ buffer (pH 7.8). The mixture was centrifuged at 1500 *g* for 10min. After partitioning, the upper phase fraction was diluted with 10 vols of dilution buffer (0.33M sucrose, 25mM HEPES, and 1mM DTT) and spun at 120 000 *g* for 1h to collect the PMs. Then, the PM vesicles were incubated with 0.02% Brij-58 detergent on ice for 10min to invert the vesicles and release the cytosolic contaminants. Samples were then diluted 20 times with double-distilled H_2_O and centrifuged at 120 000 *g* for 60min (Elmore *et al.*, 2012). The pellets were resuspended in 100 μl of suspension buffer (5mM KH_2_PO_4_/K_2_HPO_4_ buffer, pH 7.8, 3mM KCl, 1mM DTT, 0.1mM EDTA, and 1 μM protease cocktail). For each sample, five independent biological replicates were prepared.

#### Immunodetection assay and H^+^-ATPase hydrolytic activity assay

Immunoblot analysis was performed according to standard methods (Zhang *et al.*, 2011). Equal amounts of 5 μg of proteins from TM and PM fractions were separated by SDS–PAGE (Supplementary Fig. S1 availabe at *JXB* online) and transferred to nitrocellulose membranes (GE Amersham Biosciences, NY, USA) using a wet transblot system (Bio-Rad, Hercules, USA). The TM and PM proteins were probed with specific primary antibodies, and visualized using the enhanced chemiluminescence method. The primary antibodies used were anti-H^+^-ATPase (PM marker, Agrisera, Vännäs, Sweden), anti-Sar1 [secretion-associated and Ras-related protein 1, an endoplasmic reticulum (ER) marker, Agrisera], and anti-V-ATPase (tonoplast marker, Agrisera).

Hydrolytic activity of the PM H^+^-ATPase was measured according to the method of Nouri and Komastu (2010). The PM fraction was added to the reaction solution containing 30mM MES-TRIS (pH 6.5), 50mM KCl, 3mM MgSO_4_, and 3mM ATP in the presence or absence of the specific H^+^-ATPase inhibitor 0.1mM Na_3_VO_4_ and incubated at 30 °C for 15min. The reaction was stopped by the addition of 0.5% ammonium molybdate, 1% SDS, and 0.4M H_2_SO_4_. Ascorbate was then added at a final concentration of 0.3%, the mixture was placed at room temperature for 30min, and the absorbance was measured at 750nm.

#### Labelling of proteins with Cy dye

PM proteins were precipitated with the methanol/chloroform method as previously reported (Wessel and Flügge, 1984). The protein pellets were recovered in modified 2D-DIGE buffer (7M urea, 2M thiourea, 4% CHAPS, 25mM TRIS-HCl, and 1% *n*-dodecyl β-d-maltoside). The pH of protein samples was adjusted to 8.8 with HCl and NaOH, and the protein concentration was determined using the Bradford method. The internal standard was prepared by mixing equal amounts of all analysed samples. Protein samples were labelled using minimal fluorescent dyes (Cy3, Cy5, and Cy2) (GE Healthcare, Pittsburgh, PA, USA) following the manufacturer’s instruction.

#### 2D-DIGE and image scanning

The labelled protein samples were mixed with the internal standard (Supplementary Table S1 at *JXB* online), adjusted to a total volume of 450 μl with rehydration buffer [7M urea, 2M thiourea, 4% CHAPS, 1% DTT, and 1% immobilized pH gradient (IPG) buffer, pH 4–7], and used for isoelectric focusing (IEF). IEF was performed on an IPG strip holder with 24cm, linear gradient IPG strips with pH 4–7 (GE Healthcare) and then on an Ettan DALT System (GE Healthcare) according to the manufacturer’s instructions. The images were analysed using DeCyder 6.5 (GE Healthcare). Spots reproducible in 24 of 30 images were used to identify protein abundance change by two-way analysis of variance (ANOVA) (*P*<0.05). The gels used for 2D-DIGE analyses were stained with Coomassie Brilliant Blue (CBB) to observe highly abundant spots. Then, additional gels with 1mg of internal standard proteins were stained with CBB for spots that could not be determined from 2D-DIGE gels.

#### Protein identification and database searching

The 117 most abundant spots were digested in-gel with bovine trypsin (Roche Molecular Biochemicals, Indianapolis, IN, USA) as described previously (Wang *et al.*, 2007). The ultrafleXtreme matrix-assisted laser desorption ionization-time of flight/time of flight-mass spectrometer (MALDI-TOF/TOF-MS; Bruker Daltonics, Billerica, MA, USA) was used to reveal the MS/MS spectra. The peptides were suspended with 10 μl of 70% acetonitrile (ACN) containing 0.1% trifluoroacetic acid (TFA), and 1 μl was taken and spotted onto the AnchorChip™ MALDI target plate (Bruker Daltonics). Then 1 μl of matrix solution (1mg ml^–1^ α-cyano-4-hydroxycinnamic acid in 70% ACN containing 0.1% TFA) was spotted after the sample solution dried. All spectra were obtained in positive ion reflection mode under the control of FlexControl 3.3. The matrix suppression was set to deflection, 500Da. The spectra detection mass range was set at 700–4000 *m/z*. External calibration involved the use of the Bruker standard peptide calibration kit. For each spot, the top 30 strongest intensity peaks in the MS spectra and single-to-noise threshold >6 were automatically selected as precursor ions for MS/MS analysis. MS spectra were acquired with 400 laser shots per spectrum, whereas MS/MS spectra were obtained using 1500 laser shots per fragmentation spectrum. Then MS and MS/MS data were transferred to BioTools 3.2 (Bruker Daltonics) and searched against three databases with the Mascot engine 2.2.03 (http://www.matrixscience.com). The three databases were the NCBInr protein database (http://www.ncbi.nlm.nih.gov/; green plants, 1 669 695 sequences in NCBI 20131226) and two *S. europaea* transcriptome-translated protein databases, as follows: database 1 (162 969 sequences; Ma *et al.*, 2013) and database 2 (35 219 sequences; Fan *et al.*, 2013). Monoisotopic and [M+H]^+^ were selected for mass values. Peptide mass tolerance was set at 50 ppm, fragment mass tolerance was set as 0.5Da, and one missing cleavage was permitted. Carbamidomethyl (C) was set as fixed modification and Oxidation (M) was set as variable modification. All proteins were matched by Mascot with scores that exceeded their 95% confidence threshold and contained at least one peptide with a score at a significance threshold (*P*<0.05). If several database entries of homologous proteins matched these criteria, only the entry with the highest score is reported. For proteins having only one matching peptide with a score at a significant threshold (*P*<0.05), the MS/MS spectra were also presented (Supplementary Fig. S3; Supplementary Table S3 at *JXB* online).

#### Protein classification and bioinformatics analysis

The identified proteins were searched against the NCBI protein database (http://www.ncbi.nlm.nih.gov/) and Uniprot (http://www.uniprot.org/) and were divided into different groups based on their molecular and biological functions. The subcellular location of each protein was determined by SUBAIII (http://suba3.plantenergy.uwa.edu.au) and published information on the homologous proteins in *Arabidopsis*. Principal component analysis (PCA) was performed using the extended data analysis module within the DeCyder software (GE Healthcare). Heat map was performed using MultipleExperiment Viewer 4.8.1 software based on the Log_2_-transformed fold change.

#### RNA isolation and quantitative real-time PCR (qRT-PCR) analysis

Total RNA was extracted by Trizol reagent and treated with RNase-free DNase I. A 0.5 μg aliquot of RNA was used for the first-strand cDNA synthesis with the SuperScript III first-strand synthesis system (Invitrogen, Carlsbad, CA, USA). qRT-PCR was performed with an Mx3000P Real-Time PCR System (Agilent, USA) using SYBR qPCR Mix (Toyobo, Japan). The relative gene expression levels were calculated by the 2^–ΔΔCT^ method, and the α-tubulin gene was used as internal control (Lv *et al.*, 2012). qRT-PCR of each sample was repeated three times. The oligonucleotide primers used are listed in Supplementary Table S5 at *JXB* online.

#### Detection of [Ca^2+^]_*cyt*_

Thirty-day-old *S. europaea* plants irrigated with 1/2 Hoagland solution plus 200mM NaCl were used. In the 2h NaCl exposure experiment, the intact roots were incubated in loading solution containing 20 μΜ Fluo-4/AM, 50mM sorbitol, 0.2mM CaCl_2_, and 0.05% Pluronic F127 (pH 4.2) at 4 °C for 4h in the dark, followed by a 2h incubation with 0.2mM CaCl_2_ with or without 200mM NaCl at 20 °C in the dark. In the 24h, 3 d, and 30 d NaCl exposure experiments, the plants were first pre-treated with 200mM NaCl for 18h, 66h, and 30 d. Then, the intact roots of treated and control plants were incubated in loading solution with or without 200mM NaCl at 4 °C for 4h in the dark, after which the roots were incubated for 2h with 0.2mM CaCl_2_ with or without 200mM NaCl at 20 °C in the dark. After washing with buffer, [Ca^2+^]_cyt_ was determined under a laser scanning confocal microscope (Leica TCS SP5, Wetzlar, Germany) at excitation and emission wavelengths of 488nm and 505–530nm, respectively. Observations were focused on the primary root tips of plants. The same area of root tips of each sample was analysed using Image J software (National Institutes of Health, Bethesda, MD, USA) to determine the fluorescence intensity (mean pixel intensity).

#### Statistical analysis

Statistical analysis was carried out using SPSS 18.0 software. Data were evaluated by two-way ANOVA. To determine the type of interaction between saline and nitrogen treatment, taking the FW for example, the effect of saline–high nitrogen treatment (FW_HN+S_/FW_LN_) was compared with the effect of each factor when applied individually (i.e. FW_HN_/FW_LN_×FW_LN+S_/FW_LN_). Interaction was synergistic if FW_HN+S_/FW_LN_ was substantially more than FW_HN_/FW_LN_×FW_LN+S_/FW_LN_; and it was additive if FW_HN+S_/FW_LN_ was approximately equal to FW_HN_/FW_LN_×FW_LN+S_/FW_LN_, whereas it was antagonistic if FW_HN+S_/FW_LN_ was substantially less than FW_HN_/FW_LN_×FW_LN+S_/FW_LN_ (Striker *et al.*, 2015). For each experiment, at least three replications were used for analysis. The methods of significance testing are described in the figure legends.

## Results

### NaCl had a synergetic effect with nitrate on the growth of *S. europaea*

**Fig. 1.**
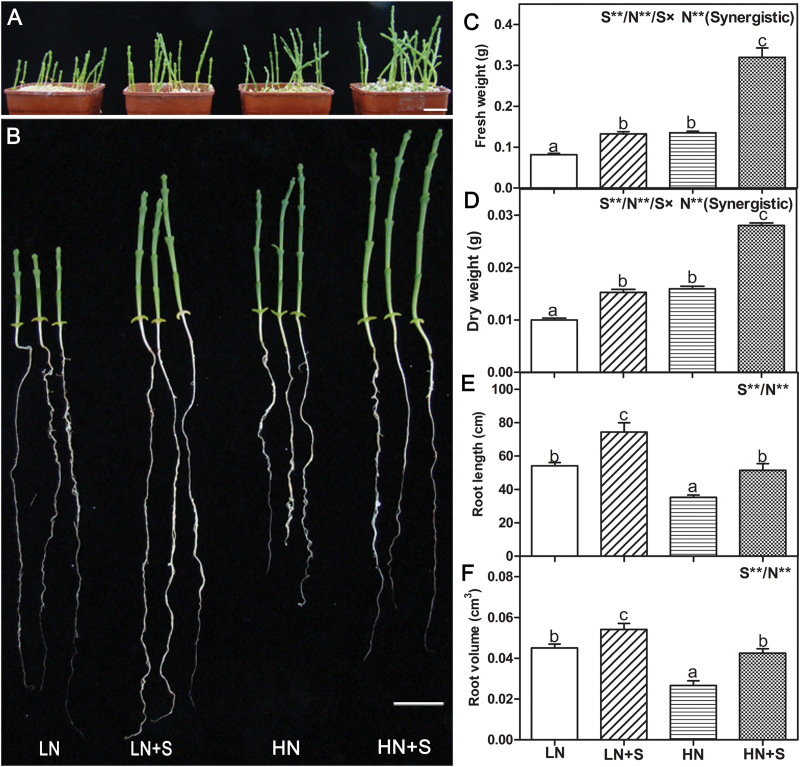
Phenotypic and physiological changes of *S. europaea* grown under different NaCl and NO_3_^–^ concentrations. *S. europaea* seedlings were treated with different nitrate and NaCl concentrations for 30 d, and the phenotypes (A and B), fresh weight (C), dry weight (D), root length (E), and root volume (F) were determined. Scale bars=2cm. Values were means ±SE (*n*=12). Different letters above the bars indicated significant differences (*P*<0.05). For C, D, E, and F, two-way ANOVA was performed to indicate significant S, N, and S×N effects. Double asterisks indicate significant differences at *P*<0.01. Combined effects are symbolized by a slash (/). Synergistic indicates the type of interaction.

In this study, the effect of NaCl and nitrate on the growth of *S. europaea* was first investigated. Seedlings were treated with four different solutions, LN, LN+S, HN, and HN+S. Generally, NaCl has a positive influence on the growth of euhalophytes, for which zero NaCl is an abnormal condition whereas 200mM NaCl is in the range of optimal condition (Flowers and Colmer, 2008). Consistently, in this study, it was found that *S. europaea* grows better in the presence of 200mM NaCl under both LN and HN conditions. In addition, high N also promotes the growth of *S. europaea* (Fig. 1A, B). The FW, DW, root length, and root volume were measured, and two-way ANOVA (*P*<0.05) was used to reveal the effects of S, N, and S×N on these parameters. All the four traits showed significant S and N effects. The FW and DW increased under NaCl and NO_3_^–^ treatments, and seedlings treated with HN+S exhibited the highest values, whereas LN showed the lowest (Fig. 1C, D). Moreover, a significant S×N interaction was observed for FW and DW, and the type of interaction was synergistic (Fig. 1C, D), suggesting that NaCl and nitrate had synergistic effects on the growth of *S. europaea*. NaCl facilitated the root growth of *S. europaea*, whereas high nitrate inhibited it. Both the root length and volume were largest under LN+S treatment, the values of which were 110.8% and 103.1% higher than those treated with HN, respectively (Fig. 1E, F).

### The effects of NaCl on nitrate uptake and assimilation

**Fig. 2.**
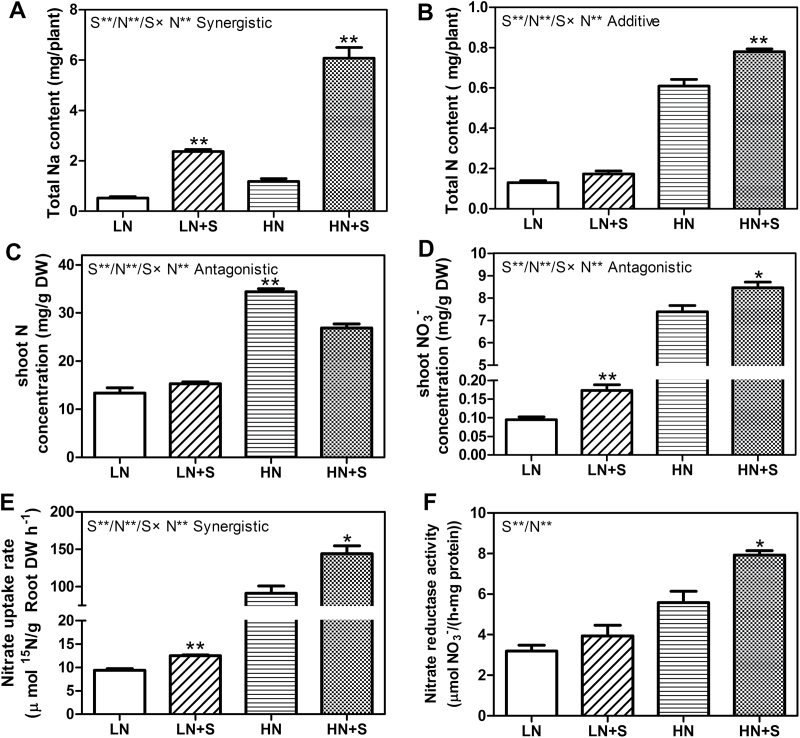
Total Na and N contents, shoot N and NO_3_^–^ concentrations, nitrate uptake rates, and NR activities of *S. europaea* grown under different NaCl and NO_3_^–^ concentrations. *S. europaea* seedlings were treated with different nitrate and NaCl concentrations for 30 d, total Na (A) and total N (B) content of the whole plant, shoot N (C) and NO_3_^–^ (D) concentration, and NR activity (F) were measured. (E) *S. europaea* seedlings were treated with four different NaCl and ^15^N labelled K^15^NO_3_ concentrations for 15min, and the content of ^15^N in roots was measured. Values were means ±SE (*n*=4 for A, B, C, D, and F; and *n*=3 for E). Asterisks indicate significant differences (*P*<0.05) between salt-treated and untreated samples. In addition, two-way ANOVA was performed to indicate significant S, N, and S×N effects. Double asterisks indicate significant differences at *P*<0.01, and combined effects are symbolized by a slash (/). Synergistic, additive, and antagonistic represent different types of interaction.

In order to investigate the effects of NaCl on the nitrate uptake and assimilation, the total Na and total N contents per plant, the N and NO_3_^–^ concentrations in shoot of *S. europaea*, the nitrate uptake rates, and nitrate reductase activities were measured (Fig. 2). All of the traits except NR activities showed significant S, N, and S×N effects. As expected, total Na contents were significantly increased under NaCl treatments, which were also promoted by N treatment (Fig. 2A). Under low N conditions, no significant effect of NaCl on total N content and shoot N concentrations was observed (Fig. 2B, C), but the shoot NO_3_^–^ concentrations and the nitrate uptake rates increased significantly by NaCl treatment. The values increased by 83.4% and 32.8% (Fig. 2D, E), respectively. Compared with the LN condition, NR activity under LN+S increased by 23.5%, but the values were very low and the difference was not statistically significant (Fig. 2F). Under high N conditions, the shoot N concentrations were significantly decreased by NaCl treatment (Fig. 2C), but the total N contents, shoot NO_3_^–^ concentration, the nitrate uptake rates, and the NR activities were all significantly increased by NaCl treatment. The values increased by 27.8, 14.6, 57.6, and 41.7%, respectively (Fig. 2B–F). Notably, NaCl and NO_3_^–^ also had a synergistic interaction effect on nitrate uptake rate. These results showed that NaCl could promote nitrate uptake and assimilation in *S. europaea*.

### Identification of NaCl- and nitrate-regulated PM proteins by 2D-DIGE

**Fig. 3.**
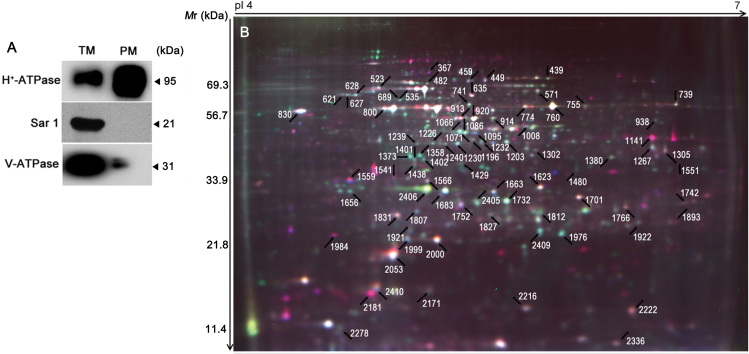
Purity assessment and a representative 2D-DIGE image. (A) Western blot analysis of extracted PM proteins with antibodies against proteins from different cell compartments. (B) PM proteins were separated by 2D-DIGE and detected with a Typhoon 9400 Scanner. The differentially accumulated protein spots under different NaCl and nitrate concentrations were determined using two-way ANOVA, and those positively identified by MALDI-TOF-TOF are marked by arrows.

To determine the proteins regulated by NaCl and nitrate in *S. europaea*, a comparative proteomic analysis of root PM proteins under the four treatments was conducted. Root PM fractions were purified by the two-phase partitioning method (Tang, 2012), and their purity was evaluated by western blot analysis. Compared with the TM fraction, H^+^-ATPase was strongly enriched in the PM fraction. Conversely, V-ATPase, a tonoplast membrane protein, was more strongly enriched in the TM fraction than in the PM fraction. Sar1, an integral ER membrane protein, was only detected in the TM fraction and was absent in the PM fraction (Fig. 3A). Furthermore, the hydrolytic activity of PM H^+^-ATPase in the presence or absence of 0.1mM of the specific PM H^+^-ATPase inhibitor Na_3_VO_4_ was determined, and the purity of PM vesicles was 84.76±2.80%_._ These results demonstrated that PM vesicles of relatively high purity were obtained.

**Fig. 4.**
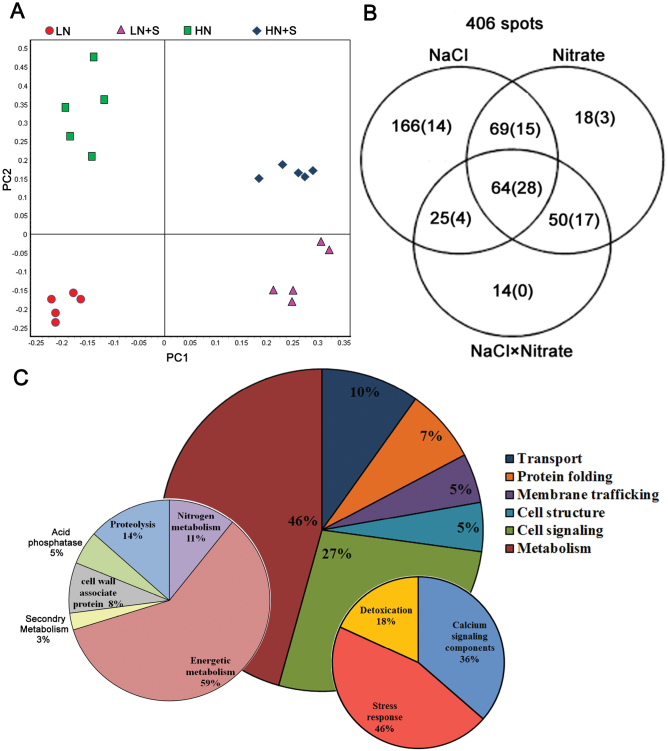
PCA, spot significant analysis, and functional classification of the identified proteins. (A) PCA of the different experimental groups. Samples plotted on the first two principal components (PCs) are shown. (B) Venn diagram showing the distribution of significant spots following two-way ANOVA. The numbers outside the parentheses are the total numbers of significant spots and at least one absolute variation was obtained above 1.5-fold by performing comparisons among the different treatments. The numbers in parentheses are the number of protein spots identified in the MALDI-TOF/TOF-MS. (C) Functional classification of 81 identified proteins. The percentage of proteins from each category is shown. The two most abundant categories, metabolism and cell signalling, were subsequently divided into subgroups.

2D-DIGE with pH 4–7 strips was used to separate root PM proteins, and ~2400 protein spots were found in each image (Fig. 3B; Supplementary Fig. S2 at *JXB* online). The 20 protein samples representing five biological repeats of each of the four treatments were assigned to 10 DIGE gels, which were labelled with Cy2, Cy3, and Cy5. A total of 30 images were acquired (Supplementary Table S1; Supplementary Fig. S2). After matching the 30 spot maps, 717 protein spots were found in 80% of them (Supplementary Table S2). PCA based on these 717 protein spots was performed to determine the distribution and similarities of different experimental groups. The five spot maps of each treatment were exactly clustered in the score plot (Fig. 4A), indicating high reproducibility among the replicate gels. Principal component 1 (PC1) explained 47.7% of the variance and separated the experimental groups according to NaCl treatment, whereas the axis PC2 accounted for 13.7% of the variance and separated the experimental groups according to nitrate treatment.

Two-way ANOVA (*P*<0.05) with false discovery rate (FDR) correction for multiple testing revealed that a total of 406 spots displayed significant S, N, and S×N effects and at least one absolute variation >1.5-fold by comparison within the different treatments. Overall, among the differentially accumulated proteins, 166, 18, and 14 spots displayed pure S, N, and S×N effects, respectively, showing that NaCl treatment had a greater impact on protein accumulation than nitrate. In addition, 69 spots displayed pure combined S and N effects, which indicated that both NaCl and nitrate affected the accumulation of these proteins, but the two factors were independent. Finally, 64 spots displayed significant S×N effect in combination with S and N effects, indicating that NaCl and nitrate had interactive effects on the accumulation of these proteins (Fig. 4B).

### Functional classification of NaCl- and nitrate-regulated proteins

After removing the dim spots and noise, the 117 most abundant spots displaying significant S, N, and/or S×N effects were manually excised from the gels, and further analysed by MS/MS spectra. Since there is a lack of information on the genome and comprehensive protein sequence *of S. europaea*, these spectra were searched against the NCBInr green plant database and two *S. europaea* transcriptome-translated protein databases (Fan *et al.*, 2013; Ma *et al.*, 2013). Most proteins blasted by the three databases were homologous proteins from different species, and the ones with the highest scores were finally selected for annotation (Supplementary Fig. S3; Supplementary Table S3 at *JXB* online). In total, 81 out of the 117 spots were annotated, among which 65 proteins have been previously shown or predicted to be associated with the PM. The localization information determined by the SUBAIII database and the literature is listed in Supplementary Table S3.

Furthermore, the identified proteins were grouped into six categories based on their molecular and biological functions, namely metabolism (46%), cell signalling (27%), transport (10%), protein folding (7%), membrane trafficking (5%), and cell structure (5%). The two major categories were further classified into subcategories. Specifically, proteins related to metabolism were divided into six subcategories, as follows: N metabolism, energetic metabolism, secondary metabolism, acid phosphates, cell wall-associated proteins, and proteolysis. Proteins involved in cell signalling were assigned to three subcategories, as follows: calcium signalling components, stress response, and detoxication (Fig. 4C; Supplementary Table S3 at *JXB* online).

### The accumulation pattern of the identified proteins

**Fig. 5.**
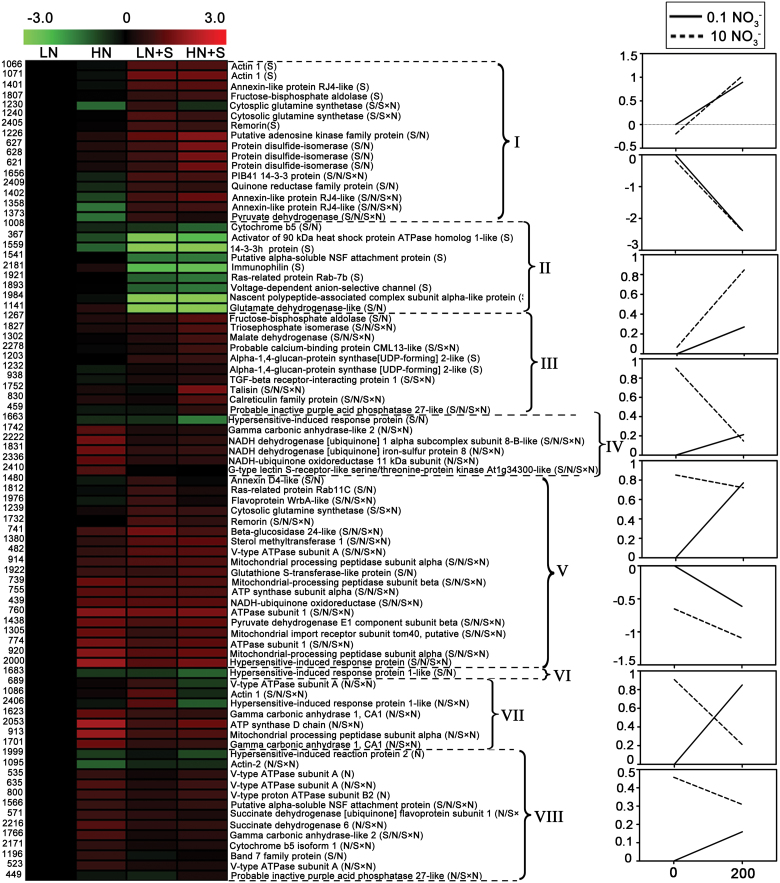
Clustering analysis of differentially accumulated proteins. The 81 differentially accumulated proteins were grouped into eight clusters according to their change patterns, which are illustrated by reaction norm diagrams consisting of a graphical representation highlighting the contribution of NaCl treatment to the observed protein abundance under nitrate treatment. Each row in the heat map indicates a single protein, whose expression values were generated using Log_2_ ($\bar{RV}$_treatments_/$\bar{RV}$_LN_). For each protein, the spot number and the protein name with its ANOVA effect (S, N, and/or S×N) are shown. Reaction norms were generated using the means of Log_2_ ($\bar{RV}$_treatments_/$\bar{RV}$_LN_) of spots for each defined cluster. $\bar{RV}$, means of normal volume/normal standard volume.

The 81 annotated, differentially accumulated proteins were further grouped into eight distinct clusters (Clusters I–VIII, Fig. 5; Supplementary Table S4 at *JXB* online) according to their change in abundance pattern. These clusters were illustrated by reaction norm diagrams consisting of a graphical representation highlighting the contribution of NaCl treatment (0mM and 200mM) to the observed protein abundance for different levels of nitrate treatments (0.1mM and 10mM). Cluster I contained 16 proteins whose accumulation increased significantly by NaCl treatment under both LN and HN conditions. Seven of these proteins were also significantly changed under nitrate treatments. Cluster II comprised nine proteins that were significantly repressed by the NaCl treatment under both LN and HN conditions. Four of these nine proteins changed significantly under nitrate treatments. Clusters III and IV comprised proteins whose abundance was increased or decreased significantly by NaCl treatment, but only under HN conditions. Cluster III included 10 proteins, three of which were significantly changed under nitrate treatments. Cluster IV contained six proteins, all of which were significantly changed under nitrate treatments. The largest cluster (Cluster V) comprised 19 proteins, which showed increased accumulation by NaCl treatment under LN conditions. Sixteen of these 19 proteins were also significantly changed under nitrate treatments. Only one protein in Cluster VI was repressed by NaCl treatment under LN conditions, and this protein was also down-regulated under nitrate supplement without NaCl. Cluster VII comprised seven proteins showing opposite responses to NaCl under LN and HN conditions. All of these proteins displayed a clear S×N interaction effect. Cluster VIII comprised 13 proteins whose abundance was not changed significantly by NaCl treatment whether under LN or HN conditions, but the abundance of such proteins changed significantly in response to nitrate.

### The involvement of Ca^2+^ signalling in NaCl-facilitated nitrate uptake in *S. europaea*

**Fig. 6.**
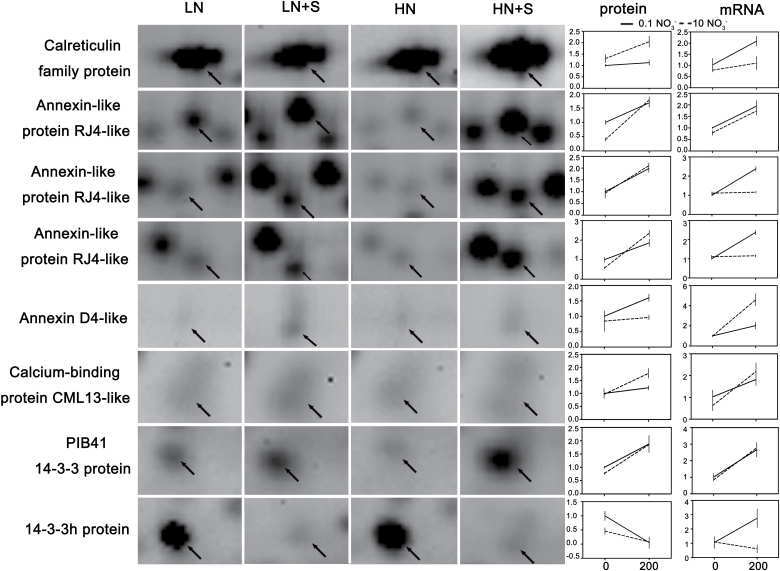
Expression profiles of calcium signalling components at protein and mRNA levels in response to NaCl and nitrate. The images on the left are magnified views of 2D-DIGE gel sections containing the spot of the calcium signalling components. The corresponding expression files at protein and mRNA levels are listed on the right. Expression levels of mRNAs were evaluated using qRT-PCR, *n*=3. The protein expression values were generated using ($\bar{RV}$_treatments_/$\bar{RV}$_LN_). $\bar{RV}$, means of normal volume/normal standard volume, *n*=5. Values are means ±SD.

Eight calcium signalling components were identified by 2D-DIGE, and seven of these components were up-regulated, whereas the other one was down-regulated by NaCl treatment under HN and/or LN conditions (Fig. 6). qRT-PCR was performed to examine whether the eight components were also regulated at the transcription level. As shown in Fig. 6, the mRNA transcripts of the eight proteins were all up-regulated by NaCl treatment under HN and/or LN conditions. Two of them, annexin (ANN)-like protein RJ4-like and PBI41 14-3-3, displayed consistency between mRNA and protein variation trends, whereas the other six proteins showed some discrepancies (Fig. 6).

**Fig. 7.**
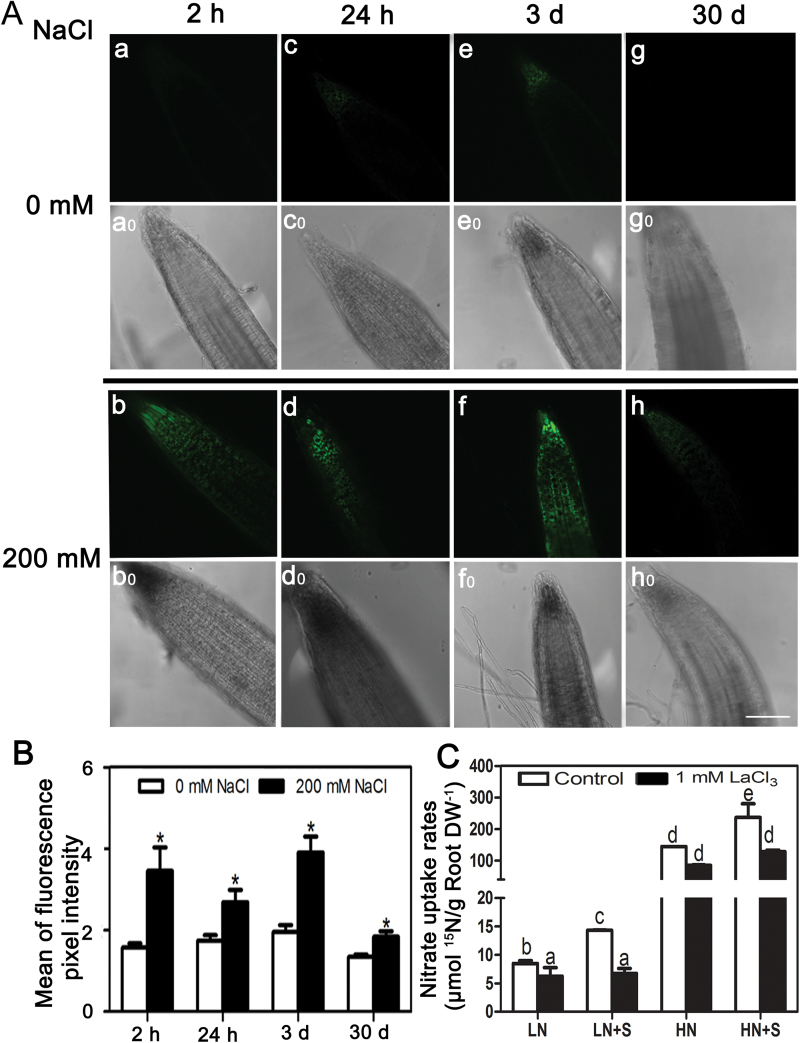
The involvement of Ca^2+^ signalling in NaCl-facilitated nitrate uptake in *S. europaea*. (A) The effects of NaCl on relative [Ca^2+^]_cyt_ in *S. europaea* root tips. *S. europaea* seedlings treated with 200mM NaCl for 2h, 24h, 3 d, and 30 d were incubated with Fluo-4/AM, and root tips were observed by confocal microscopy (b, d, f, and h). The root tips without NaCl treatments served as controls (a, c, e, and g), a0 to h0 are light field images of the corresponding root tip. Scale bar=100 μm. (B) Quantification of average fluorescence intensities of root tips. Values are means ±SE (*n*=10). Asterisks indicate significant differences (*P*<0.05). (C) The effect of LaCl_3_ on nitrate uptake rates of *S. europaea* roots. LaCl_3_-pre-treated *S. europaea* seedlings were immersed in modified 1/2 Hoagland solutions with different NaCl and ^15^N-labelled KNO_3_ concentrations for 15min, and the content of ^15^N in roots was measured. Values are means ±SE (*n*=3). Different letters above the bars indicate significant differences at *P*<0.05, which were calculated separately under LN and HN conditions.

Previous studies have shown that [Ca^2+^]_cyt_ fluctuations and the downstream calcium signalling proteins play pivotal roles in stress responses (Plieth, 2005). Thus, the dynamic changes of [Ca^2+^]_cyt_ under 200mM NaCl treatment were determined in *S. europaea* roots using the Ca^2+^-sensitive fluorescent probe, Fluo-4/AM. In contrast to weak fluorescence detected in the control root tips, strong fluorescence was observed in roots treated with NaCl for 2h, 24h, and 3 d (Fig. 7A, B). Although the fluorescence from 30 d NaCl-treated root tips was weak, it was still significantly higher than that of the control. Statistical analysis of the mean intensity of fluorescence showed that [Ca^2+^]_cyt_ was significantly elevated under NaCl treatments (Fig. 7B).

To investigate the effects of [Ca^2+^]_cyt_ on nitrate uptake under NaCl treatments in *S. europaea*, LaCl_3_ (a Ca^2+^ channel blocker) was applied to *S. europaea* roots, and the nitrate influx rates were detected. Pre-treatment with LaCl_3_ resulted in decreased [Ca^2+^]_cyt_ (Supplementary Fig. S4 at *JXB* online), and the nitrate uptake rates of roots were significantly reduced under the three treatments besides the HN conditions compared with the control. Moreover, the reduction was much more pronounced under NaCl treatments than without NaCl treatments. After LaCl_3_ pre-treatment, no significant difference in the nitrate uptake rate was observed between LN and LN+S, or between HN and HN+S (Fig. 7C). This result indicated that the decrease in [Ca^2+^]_cyt_ not only caused a decrease in nitrate uptake rate, but also attenuated the promotion effects of NaCl on this parameter.

## Discussion

### NaCl facilitates nitrate uptake in *S. europaea*

Generally, NaCl has inhibitory effects on nitrate assimilation in glycophytes. For example, in *Arabidopsis*, NO_3_^–^ concentrations and NR activity in both leaves and roots were markedly decreased by NaCl during the second week of treatment (Debouba *et al.*, 2013). However, in this study, it was found that NaCl can promote nitrate uptake in the euhalophyte *S. europaea*. The shoot NO_3_^–^ concentration and nitrate uptake rates of *S. europaea* were significantly promoted by NaCl under both low N and high N conditions (Fig. 2D, E). These results are consistent with the findings for the euhalophyte *S. physophora* which showed that NaCl application significantly increased the leaf NO_3_^–^ concentration under N-sufficient conditions (Yuan *et al.*, 2010). The current results and previous studies together show that the effect of NaCl on nitrate uptake and assimilation may be significantly different between glycophytes and halophytes. NaCl generally has a positive influence on the growth of euhalophytes, for which zero NaCl is an abnormal condition whereas 200mM NaCl is in the range of the optimal condition (Flowers and Colmer, 2008). Previous studies showed that the growth of *S. europaea* is stimulated by 200–400mM NaCl (Wang *et al.*, 2009; Lv *et al.*, 2012). Consistently, in this study, it was found that supply of 200mM NaCl improved the growth of *S. europaea* under both LN and HN conditions (Fig. 1A, B). Better growth of *S. europaea* under salt conditions increased the demand for N, which could at least partly explain the stimulation of nitrate uptake and increased plant N content by salt treatment (Fig. 2B, E). Furthermore, there might be an NaCl-facilitated nitrate uptake mechanism in *S. europaea*, which is directly supported by the findings that nitrate uptake rates and shoot NO_3_^–^ concentrations of *S. europaea* were significantly promoted by NaCl treatment under both high N and low N conditions (Fig. 2D, E). In addition, the NR activities were significantly increased by salt under the high N condition (Fig. 2F). Consistent with this study, a sodium-dependent nitrate transport at the PM of leaf and root cells was also reported in *Zostera marina*, which is a seagrass that grows submerged in seawater where the NaCl concentration is ~500mM (Rubio *et al.*, 2005).

### The possible roles of Ca^2+^ signalling proteins identified by comparative proteomics in *S. europaea*

Ca^2+^ plays vital roles in plant development and in response to environmental stimuli (DeFalco *et al.*, 2010). Developmental and environmental cues are perceived at the cell surface, thereby eliciting [Ca^2+^]_cyt_ changes through concerted action of channels, pumps, and carriers (Kudla *et al.*, 2010). Two protein families, ANN and calreticulin (CRT), involved in this process were identified in this proteomic study. ANN encodes unconventional calcium-permeable channels, which is a key component of root cell adaptation to salinity (Davies, 2014). Four ANN proteins (spots 1358, 1401, 1402, and 1480) were significantly increased in response to NaCl (Fig. 6), indicating that they may act as NaCl-regulated Ca^2+^-permeable channels in *S. europaea*. Interestingly, spots 1401 and 1402 represented the same protein, which have the same molecular mass but different pI values (Fig. 3B). It is speculated that the isoforms may be generated by phosphorylation of ANN. CRT is a highly conserved Ca^2+^-binding protein functioning in intracellular Ca^2+^ homeostasis and signalling and is predominantly located in the ER; CRT is reportedly associated with the extracellular membrane surface (Jia *et al.*, 2009). CRT is an NaCl-responsive protein in potato (Aghaei *et al.*, 2008), as well as in *Arabidopsis* (Jiang *et al.*, 2007). In *S. europaea*, the abundance of CRT (spot 830) was up-regulated by NaCl only under HN conditions, and it was also induced by HN under NaCl application (Fig. 6), indicating that CRT may act as an NaCl- and HN-responsive protein in *S. europaea* regulating calcium homeostasis and signalling transduction.

The spatio-temporal Ca^2+^ signals are then decoded and transmitted by Ca^2+^-binding proteins (DeFalco *et al.*, 2010). Calmodulin (CaM)-like (CML) and 14-3-3 proteins involved in this process were identified. CMLs are involved in Ca^2+^ signal transduction by associating with numerous downstream targets, such as protein kinase, phosphatases, transcription factors, transporters, and channels (Popescu *et al.*, 2007). CMLs have been implicated in plant development processes and abiotic stress responses (Magnan *et al.*, 2008). In this study, one CML13-like protein was up-regulated by NaCl under HN conditions (Fig. 6), and this protein may function in Ca^2+^ signalling as a part of the response of *S. europaea* to NaCl under N-sufficient conditions. In contrast, the accumulation of CML was significantly down-regulated after 48h salt treatment in roots of barely, which was accompanied by significant reduction of root Ca^2+^ content (Wu *et al.*, 2014). These different trends may reflect diverse mechanisms of CML regulation in glycophytes and halophytes. 14-3-3 proteins are also involved in Ca^2+^ signalling, which can interact with Ser/Thr-phosphorylated proteins (Pauly *et al.*, 2007). They have been reported to regulate plant N assimilation by tuning the activities of NRT2, NR, and glutamine synthase (GS). Two phosphorylation sites of NRT2.1 were identified as being regulated by NaCl treatment (Vialaret *et al.*, 2014), and sequence analysis of NRT2s in tobacco and *Arabidopsis* has identified some possible 14-3-3-binding sites (Guo *et al.*, 2011). The activities of cytosolic NR and GS, the key enzymes in nitrate assimilation, are regulated through phosphorylation by 14-3-3s, calcium-independent protein kinases (CIPKs), and calcium-dependent protein kinases (CDPKs) (Masclaux-Daubresse *et al.*, 2010). NR is the target of CPK17 and 14-3-3, which is regulated by salt stress (Lambeck *et al.*, 2010). Notably, the two 14-3-3 proteins identified in this study changed oppositely in response to NaCl treatment (Fig. 6). Similarly, in salt-tolerant rice strains, the abundance of two 14-3-3 proteins increased, whereas one 14-3-3 protein decreased under salt stress (Liu *et al.*, 2014). These findings indicate that NaCl may regulate 14-3-3 proteins through multiple mechanisms.

### Changes in [Ca^2+^]_cyt_ and downstream Ca^2+^ signalling are essential for NaCl-facilitated nitrate uptake in *S. europaea*

Salt stress is first perceived at the cell membrane level, and then an intracellular signalling cascade is triggered via secondary messengers (Kader and Lindberg, 2010). The present results suggested that Ca^2+^ acted as an essential second messenger in NaCl-facilitated nitrate uptake in *S. europaea* and functioned through downstream signalling components. First, [Ca^2+^]_cyt_ was significantly elevated in *S. europaea* under both short- and long-term NaCl treatment (Fig. 7A, B). Many studies have revealed an increase in [Ca^2+^]_cyt_ after salt stress (Kader *et al.*, 2007; D’Onofrio and Lindberg, 2009). NaCl induces a higher increase in [Ca^2+^]_cyt_ in salt-tolerant rice cultivars than in salt-sensitive cultivars (Kader *et al.*, 2007). However, in *Arabidopsis* root cells or corn root protoplast, NaCl induced a decrease in [Ca^2+^]_cyt_ within minutes (Lynch and Läuchli, 1988; Halperin *et al.*, 2003). Thus, the regulation of [Ca^2+^]_cyt_ by NaCl varies with the tested species, cells, and tissues. It is likely that the amplitude, frequency, and duration of changes in [Ca^2+^]_cyt_ are important for decoding the specific downstream responses for salt stress response in plants. Secondly, a decline in [Ca^2+^]_cyt_ not only caused a decrease in nitrate uptake rate, but also eliminated the promotion effects of NaCl on this parameter (Fig. 7C), suggesting that the NaCl-induced [Ca^2+^]_cyt_ elevation was essential for NaCl-facilitated nitrate uptake in *S. europaea*. Evidence on the direct relationship between [Ca^2+^]_cyt_ and nitrate uptake is still lacking, but some research has shown links between them. Application of Ca^2+^ could increase nitrate uptake under salt stress, and this effect may be ascribed to its involvement in preserving the structural and functional integrity of cell membranes or in increasing the activities of nitrate transporters (Mansour, 2000). Meanwhile, plants treated with EGTA (a specific Ca^2+^ chelator) or La^3+^ (a Ca^2+^ channel blocker) showed decreased induction of the key genes involved in the nitrate assimilatory pathway, such as NR and nitrite reductase (NiR) (Vidal *et al.*, 2010). Thirdly, eight calcium signalling proteins were identified in this proteomic study, seven of which were significantly up-regulated under NaCl treatments (Fig. 6); it is speculated that these may play important roles in NaCl-facilitated nitrate uptake in *S. europaea*. The important roles of Ca^2+^ signalling proteins in salt adaption have been reported in other plant species. In *Arabidopsis*, the phosphorylation levels of CPKs changed significantly under salt stress (Vialaret *et al.*, 2014). Mehlmer *et al.* (2010) also reported that CPK3-mediated Ca^2+^ signalling is required for salt stress acclimation, and 28 potential CPK3 targets were identified by their studies. Among these, remorin, CRT, VDAC (voltage-dependent anion channel), 14-3-3 proteins, and RAB GTPase, all of which play roles in cellular ion homeostasis and signal transduction under salt stress (Mehlmer *et al.*, 2010), were also identified in the present study. In addition, Latz *et al.* (2013) reported that the vacuolar two-pore K^+^ channel 1 (TPK1) was a target of CPK3 and 14-3-3 protein, and played an essential role in the regulation of the cytosolic K^+^/Na^+^ ratio under salt stress adaptations.

**Fig. 8.**
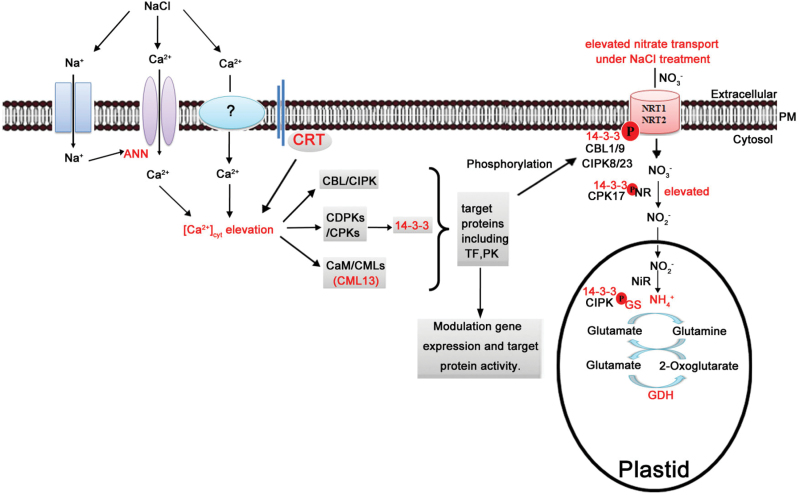
A putative regulatory network of NaCl-facilitated nitrate uptake in *S. europaea*. In *S. europaea* roots, the NaCl stimuli induced Ca^2+^ entry across the PM by activating ANN protein and some unknown Ca^2+^ channels, thereby causing [Ca^2+^]_cyt_ elevation. CRT is involved in cytosolic Ca^2+^ homeostasis. The [Ca^2+^]_cyt_ elevation is sensed by calcium signalling proteins including CaM/CML, CBL/CIPKs, and CDPKs/CPKs. 14-3-3 protein can be activated by CPK3 or some unknown Ca^2+^ signalling components. The calcium signalling proteins can transmit the signal into phosphorylation cascades capable of modulating gene expression and target protein activity, which may function in ion transport/homeostasis, membrane trafficking, redox homeostasis, and so on. CBL1/9 and CIPK8/23 are involved in the regulation of NRT1.1 by phosphorylation while 14-3-3 is involved in the regulation of NRT2s. NRs in the cytosol are targets of CPK17 and 14-3-3, which are also regulated by salt stress. The subsequent steps of N assimilation take place in plastids. GS participates in this process and can be regulated by CIPK and 14-3-3 through phosphorylation. The components in red indicate those identified in the present study.

Based on the present results and previous studies, a possible regulatory network of NaCl-facilitated nitrate uptake in *S. europaea* focusing on the involvement of Ca^2+^ signalling is proposed (Fig. 8). In *S. europaea* roots, the NaCl stimuli induced Ca^2+^ entry across the PM by activating ANN protein and some unknown Ca^2+^ channels (Davies, 2014), thereby causing [Ca^2+^]_cyt_ elevation. CRT is involved in cytosolic Ca^2+^ homeostasis (Jia *et al.*, 2009). The [Ca^2+^]_cyt_ elevation is sensed by calcium signalling proteins including CaM/CML, CBL/CIPKs, as well as CDPKs/CPKs (DeFalco *et al.*, 2010). 14-3-3 protein can be activated by CPK3 or some unknown Ca^2+^ signalling components (Mehlmer *et al.*, 2010). The calcium signalling proteins can transmit the signal into phosphorylation cascades capable of modulating gene expression, and target protein activity, which may function in ion transport/homeostasis, membrane trafficking, redox homeostasis, and so on (DeFalco *et al.*, 2010). Thus, the elevated nitrate uptake rates and NR activity under NaCl treatment in *S. europaea* may be ascribed to calcium-dependent phosphorylation of proteins such as those described in the following sentences. CBL1/9 and CIPK8/23 are involved in the regulation of NRT1.1 by phosphorylation (Ho *et al.*, 2009; Hu *et al.*, 2009), while 14-3-3 is involved in the regulation of NRT2s (Guo *et al.*, 2011). NRs in the cytosol are targets of CPK17 and 14-3-3, which are also regulated by salt stress (Lambeck *et al.*, 2010). The subsequent steps of N assimilation in roots take place in plastids. GS participates in this processs and can be regulated by CIPK and 14-3-3 through phosphorylation (Masclaux-Daubresse *et al.*, 2010).

## Conclusions

In this study, it was first found that, unlike in glycophytes, NaCl facilitates nitrate uptake in *S. europaea*, which may be a unique feature in halophytes. Further comparative proteomics of root PM proteins revealed 81 differentially accumulated proteins in response to salt and nitrate. Among them, there are eight calcium signalling components, and the accumulations of seven was increased in response to salinity. [Ca^2+^]_cyt_ was significantly elevated in *S. europaea* under both short- and long-term NaCl treatment. In addition, application of the Ca^2+^ channel blocker LaCl_3_ not only caused a decrease in nitrate uptake rate but also reduced the promotion effects of NaCl on the nitrate uptake rates. It is proposed that NaCl induced [Ca^2+^]_cyt_ elevation and the downstream calcium signalling are essential for NaCl-facilitated nitrate uptake in *S. europaea*. The calcium signalling proteins may function through regulating the expression or activity of nitrate transporters and some key enzymes in N assimilation, which can consequently affect nitrate assimilation.

## Supplementary data

Supplementary data are available at *JXB* online.

Figure S1. Coomassie blue-stained gel image of TM and PM samples.

Figure S2. Ten images of 2D-DIGE.

Figure S3. Scores and matched peptides of the identified proteins based on ultrafleXtreme MALDI-TOF/TOF-MS.

Figure S4. The effect of LaCl_3_ on the relative [Ca^2+^]_cyt_ in *S. europaea* root tips.

Table S1. Protein samples assigned for Cy dye label and DIGE.

Table S2. Expression profile data of the 717 spots present in at least 24 of the 30 images.

Table S3. The differentially accumulated *S. europaea* PM proteins identified by MALDI-TOF/TOF.

Table S4. Protein distribution in the eight clusters.

Table S5. Specific primers used for real-time PCR of genes encoding calcium signalling components of *S. europaea.*

Supplementary Data

## Abbreviations:

ANN: annexin
ANOVA: analysis of variance
[Ca^2+^]_cyt_: cytosolic Ca^2+^ concentration
CaM: calmodulin
CBL: calcineurin B-like
CDPK: calcium-dependent protein kinase
CIPK: calcium-independent protein kinase
CML: calmodulin-like
CRT: calreticulin
2D-DIGE: two-dimensional fluorescence difference gel electrophoresis
DW: dry weight
FW: fresh weight
GDH: glutamate dehydrogenase
GS: glutamine synthase
NiR: nitrite reductase
NR: nitrate reductase
NRT: nitrate transporter
PM: plasma membrane
TM: total microsomal
VDAC: voltage-dependent anion-selective channel.
