# Supplementary material for: Comparative proteomics of root plasma membrane proteins reveals the involvement of calcium signalling in NaCl-facilitated nitrate uptake in Salicornia europaea
Source: J Exp Bot. 2015 May 8;66(15):4497–510. doi: 10.1093/jxb/erv216 (PMC4507759; doi:10.1093/jxb/erv216)
Supplement: Supplementary Data [file supp_66_15_4497__index.html]

Comparative proteomics of root plasma membrane proteins reveals the involvement of calcium signalling in NaCl-facilitated nitrate uptake in Salicornia europaea — Comparative proteomics of root plasma membrane proteins reveals the involvement of calcium signalling in NaCl-facilitated nitrate uptake in Salicornia europaea — Supplementary Data 

# Comparative proteomics of root plasma membrane proteins reveals the involvement of calcium signalling in NaCl-facilitated nitrate uptake in *Salicornia europaea*

## Supplementary Data

Data files

**Files in this Data Supplement:**

- Supplementary Data - Supplementary Data
- Supplementary Data - Supplementary Data
- Supplementary Data - Supplementary Data
- Supplementary Data - Supplementary Data
- Supplementary Data - Supplementary Data
- Supplementary Data - Supplementary Data
