# Supplementary material for: Comparative proteomics of root plasma membrane proteins reveals the involvement of calcium signalling in NaCl-facilitated nitrate uptake in Salicornia europaea
Source: J Exp Bot. 2015 May 8;66(15):4497–510. doi: 10.1093/jxb/erv216 (PMC4507759; doi:10.1093/jxb/erv216)
Supplement: Supplementary Data [file supp_erv216_jexbot144980_file001.pdf]

Title: Comparative proteomics of root plasma membrane proteins reveals the involvement of calcium signaling in NaCl-facilitated nitrate uptake in *Salicornia europaea*

Lingling Nie[1]*, Juanjuan Feng[1]*, Pengxiang Fan[1,2]*, Xianyang Chen[1], Jie Guo[1], Sulian Lv[1], Hexigeduleng Bao[1,3], Weitao Jia[1], Fang Tai[1], Ping Jiang[1], Jinhui Wang[1], Yinxin Li[1†]

Supplementary Figure S1. Coomassie blue-stained gel image of TM and PM samples.

Supplementary Figure S2. Ten images of 2D-DIGE. Each image comprises the merged images of two samples (red and green) and one internal standard (blue) according to the design in Supplementary Table S1.

Supplementary Figure S3. Scores and matched peptides of the identified proteins based on ultrafleXtreme MALDI TOF/TOF MS. Proteins are listed in an order consistent with that in Supplementary Table S3. The numbers were consistent with those in 2D-DIGE gel. The matched peptides and individual peptide scores for the identified proteins and the search results from three databases are given. The three databases were NCBInr protein database (http://www.ncbi.nlm.nih.gov/; Green plants, 1,669,695 sequences in NCBI 20131226), and two *S. europaea* transcriptome-translated protein databases, as follows: database 1 (162,969 sequences) and database 2 (3, 5219 sequences). For proteins that had only one matching peptide with a score at a significance threshold ($P < 0.05$), the MS/MS spectra was also presented.

Supplementary Figure S4. The effect of $LaCl_3$ on the relative $[Ca^{2+}]_{cyt}$ in *S. europaea* root tips. Plants pretreated with 1 mM $LaCl_3$ for 24 h and control plants (no $LaCl_3$ pretreatment) were incubated with Fluo-4/AM and treated with 200 mM NaCl for 2 h. Root tips were observed by confocal microscope, and relative fluorescence intensity was quantified.

Figure S1

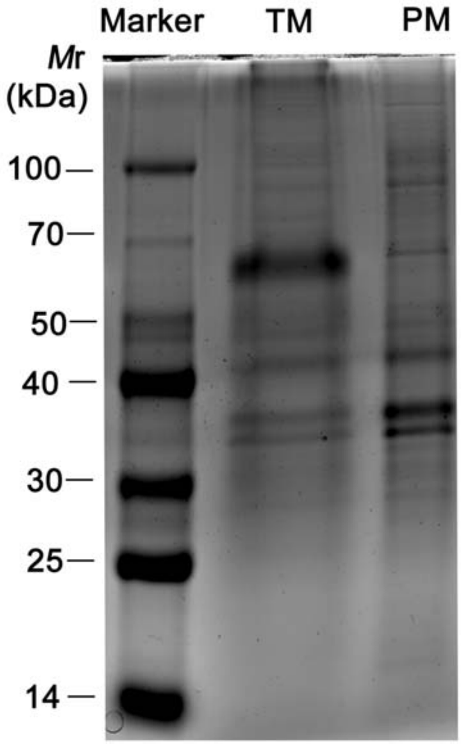

Figure S2

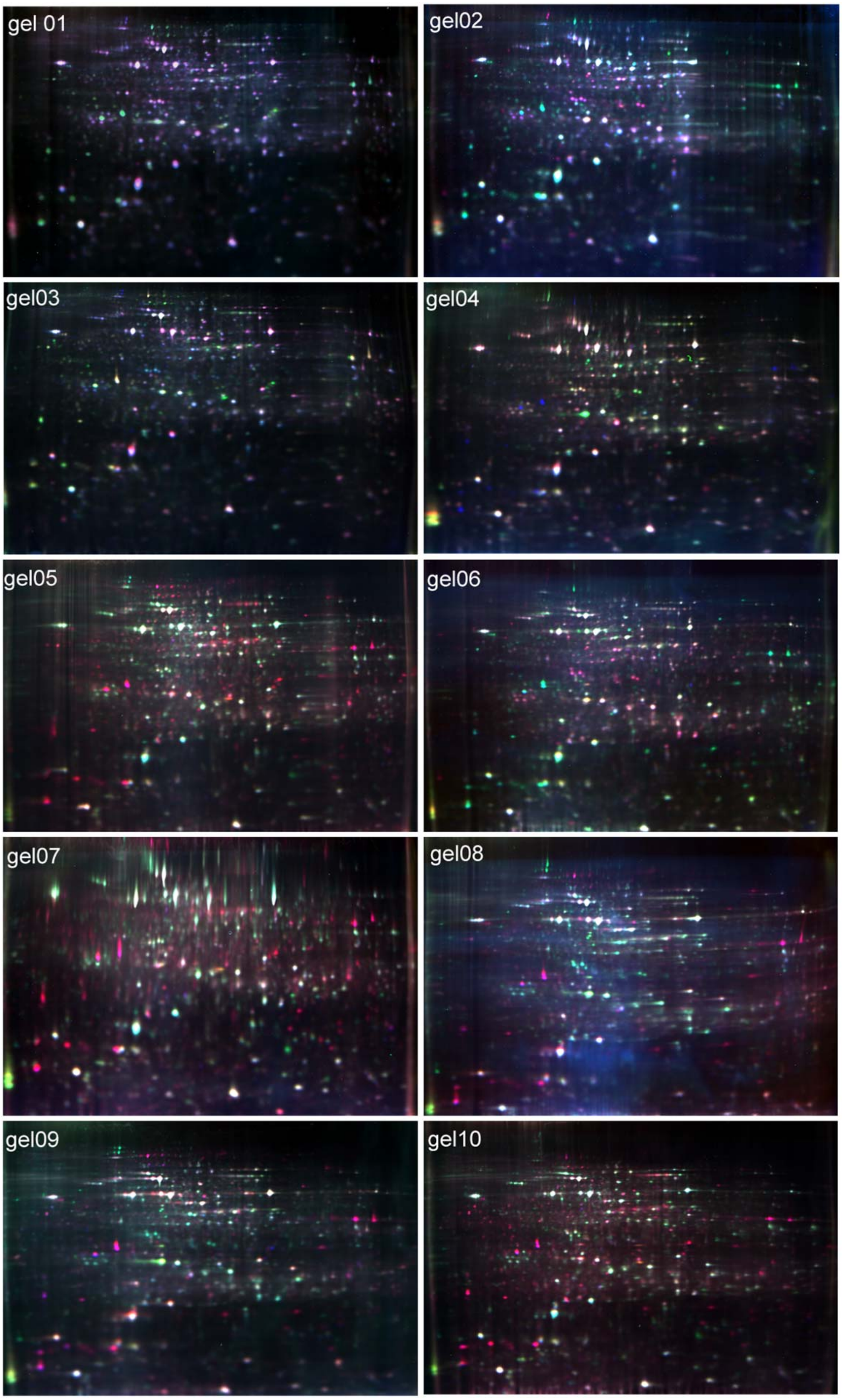
gel 01
gel02
gel03
gel04
gel05
gel06
gel07
gel08
gel09
gel10

Figure S3

Spot 482

NCBInr protein database

1. Match to: gi|336112678 Mass: 68845 Score: 344 Matches: 6(4) Sequences: 6(4)

V-type ATPase subunit A [*Salicornia europaea*]

Matched peptides shown in **bold red**.

1 MPSVYADRMT TFEDSEKESE YGYIRKVSGP VVVADGMAGA AMYELVRVGN

51 **DNLIGEIIR**L EGDSATIQVY EETGGLTVND PVLRTHKPLS VELGPGILGN

101 IFDGIQRPLK TIAIKSGDVY IPRGVSVPPL DKDALWDFQP NKLGEGDLLT

151 GGDLYAIVDE NTLMKHHVAL PPDAMGKITY IAPPGQYSIM DTVLELEFQG

201 VVKKFTMLQT WPVRTPRPVS SK**LAADTPLL TGQR**VLDALF PSVLGGTCAI

251 PGAFGCGKTV ISQALSK**YSN SDAVVYVGCG ER**GNEMAEVL MDFPQLTMTL

301 PDGREESVMK RTTLVANTSN MPVAAR**EASI YTGITIAEYF R**DMGNNVSMM

351 ADSTSRWAEA LREISGR**LAE MPADSGYPAY LAA**RLASFYE RAGKVKCLGG

401 PERNGSVTIV GAVSPPGGDF SDPVTSATLS IVQVFWGLDK KLAQRKHFPS

451 VNWLISYSKY STALESFYEK **FDPEFIDIR**T KAREVLQRED DLNEIVQLVG

501 KDALAETDKI TLDTAKLLRE DYLAQNAFTA YDKFCPFYKS VWMMRNIIHF

551 YNLANQAVER GAGSDGQKIT YSLIKHKLGD LFYRLVSQKF EDPAEGEDTL

601 VAKFKKLNDD LSASFRNLED ETR

Query	Observed	Mr(expt)	Mr(calc)	ppm	Miss	Score	Expect	Rank	Unique	Peptide
6	1151.6237	1150.6164	1150.5659	43.9	0	38	0.61	1	U	K.FDPEFIDIR.T
8	1255.7197	1254.7125	1254.6932	15.4	0	57	0.0051	1		K.LAADTPLLTGQR.V
11	1312.7426	1311.7353	1311.7146	15.8	0	39	0.32	1	U	R.VGNDNLIGEIIR.L
13	1675.7470	1674.7397	1674.7308	5.34	0	91	1.5e-06	1		K.YSNSDAVVYVGCGER.G
15	1733.8905	1732.8832	1732.8672	9.27	0	60	0.0024	1		R.EASIYTGITIAEYFR.D
22	1811.8694	1810.8622	1810.8559	3.43	0	60	0.0024	1		R.LAEMPADSGYPAYLAAR.L + Oxidation (M)

2. gi|2506211 Mass: 68923 Score: 268 Matches: 4(4) Sequences: 4(4)

RecName: Full=V-type proton ATPase catalytic subunit A; Short=V-ATPase subunit A; AltName: Full=V-ATPase 69 kDa subunit; AltName: Full=VAA3-1; AltName: Full=Vacuolar proton pump subunit alpha

Query	Observed	Mr(expt)	Mr(calc)	ppm	Miss	Score	Expect	Rank	Unique	Peptide
8	1255.7197	1254.7125	1254.6932	15.4	0	57	0.0051	1		K.LAADTPLLTGQR.V
13	1675.7470	1674.7397	1674.7308	5.34	0	91	1.5e-06	1		K.YSNSDAVVYVGCGER.G
15	1733.8905	1732.8832	1732.8672	9.27	0	60	0.0024	1		R.EASIYTGITLAEYFR.D
22	1811.8694	1810.8622	1810.8559	3.43	0	60	0.0024	1		R.LAEMPADSGYPAYLAAR.L + Oxidation (M)

Proteins matching the same set of peptides:

gi|66816974 Mass: 41510 Score: 268 Matches: 4(4) Sequences: 4(4)

vacuolar H+-ATPase subunit A [*Vigna unguiculata*]

gi|356521645 Mass: 69021 Score: 268 Matches: 4(4) Sequences: 4(4) ‘

PREDICTED: V-type proton ATPase catalytic subunit A-like [*Glycine max*]

gi|363806992 Mass: 69021 Score: 268 Matches: 4(4) Sequences: 4(4)

V-type proton ATPase catalytic subunit A-like [*Glycine max*]

3. gi|514709242 Mass: 68637 Score: 176 Matches: 3(3) Sequences: 3(3)

PREDICTED: V-type proton ATPase catalytic subunit A-like [*Setaria italica*]

Query	Observed	Mr(expt)	Mr(calc)	ppm	Miss	Score	Expect	Rank	Unique	Peptide
8	1255.7197	1254.7125	1254.6932	15.4	0	57	0.0051	1	U	K.IAADTPLLTGQR.V
15	1733.8905	1732.8832	1732.8672	9.27	0	60	0.0024	1		R.EASIYTGITIAEYFR.D
22	1811.8694	1810.8622	1810.8559	3.43	0	60	0.0024	1		

R.LAEMPADSGYPAYLAAR.L + Oxidation (M)

4. gi|255080478 Mass: 68480 Score: 150 Matches: 2(2) Sequences: 2(2)

H+-or Na+-translocating f-type, v-type and A-type ATPase superfamily [*Micromonas sp. RCC299*]

Query	Observed	Mr(expt)	Mr(calc)	ppm	Miss	Score	Expect	Rank	Unique	Peptide
13	1675.7470	1674.7397	1674.7308	5.34	0	91	1.5e-06	1	U	K.YSNSDGIVYVGCGER.G
15	1733.8905	1732.8832	1732.8672	9.27	0	60	0.0024	1		R.EASIYTGITLAEYFR.D

Database 1

Match to: Unigene18901_Se200S transcribed RNA sequence Mass: 23700 Score: 211 Matches: 3(3) Sequences: 3(3)

gi|222424801 AT1G78900 [*Arabidopsis thaliana*]

Matched sequence in Database 1:

Matched peptides shown in bold red.

TVISQALSKYSNSDAVVYVGCGERGNEMAEVLMDFPQLTMTLPDGREESVMKRTTLVANTSNMPVAAREASIYTGITIAEYF

RDMGNNVSMMADSTSRWAEALREISGRLAEMPADSGYPAYLAARLASFYERAGKVKCLGGPERNGSVTIVGAVSPPGGDFS

DPVTSATLSIVQVFWGLDKKLAQRKHFPSVNWLISYSKYSTALESFYEKFDPE

Query	Observed	Mr(expt)	Mr(calc)	ppm	Miss	Score	Expect	Rank	Unique	Peptide
13	1675.7470	1674.7397	1674.7308	5.34	0	91	2e-008	1	U	K.YSNSDAVVYVGCGER.G
15	1733.8905	1732.8832	1732.8672	9.27	0	60	5.1e-005	1	U	R.EASIYTGITIAEYFR.D
22	1811.8694	1810.8622	1810.8559	3.43	0	60	4.9e-005	1	U	

R.LAEMPADSGYPAYLAAR.L + Oxidation (M)

Proteins matching the same set of peptides:

Unigene45737_SeCKS transcribed RNA sequence Mass: 22692 Score: 211 Matches: 3(3) Sequences: 3(3)

gi|222424801 AT1G78900 [*Arabidopsis thaliana*]

2. Unigene12325_SeCKS transcribed RNA sequence Mass: 27816 Score: 96 Matches: 2(2) Sequences: 2(2)

gi|336112678 V-type ATPase subunit A [*Salicornia europaea*]

Query	Observed	Mr(expt)	Mr(calc)	ppm	Miss	Score	Expect	Rank	Unique	Peptide
8	1255.7197	1254.7125	1254.6932	15.4	0	57	7.7e-005	1	U	K.LAADTPLLTGQR.V
11	1312.7426	1311.7353	1311.7146	15.8	0	39	0.0053	1	U	R.VGNDNLIGEIIR.L

Proteins matching the same set of peptides:

Unigene47073_Se200S transcribed RNA sequence Mass: 28308 Score: 96 Matches: 2(2) Sequences: 2(2)

gi|336112678 V-type ATPase subunit A [*Salicornia europaea*]

Database 2

Match to: Unigene52395_SALfmcTARAAPEI-3 Mass: 29936 Score: 344 Matches: 7(5) Sequences: 7(5)

Score: 344

gi|336112678 V-type ATPase subunit A [*Salicornia europaea*]

Matched peptides shown in bold red.

PVSSKLAADTPLLTGQRVLDALFPSVLGGTCAIPGAFGCGKTVISQALSKYSNSDAVVYVGCGERGNEMAEVLMDFPQLTMTL

PDGREESVMKRTTLVANTSNMPVAAREASIYTGITIAEYFRDMGNNVSMMADSTSRWAEALREISGRLAEMPADSGYPAYLAA

RLASFYERAGKVKCLGGPERNGSVTIVGAVSPPGGDFSDPVTSATLSIVQVFWGLDKKLAQRKHFPSVNWLISY

Query	Observed	Mr(expt)	Mr(calc)	ppm	Miss	Score	Expect	Rank	Unique	Peptide
1	745.4543	744.4471	744.3918	74.2	0	28	0.063	1	U	R.WAEALR.E
4	885.5150	884.5077	884.4392	77.5	0	10	2.5	4	U	R.LASFYER.A
6	1151.6237	1150.6164	1150.5659	43.9	0	38	0.0043	1	U	K.FDPEFIDIR.T
8	1255.7197	1254.7125	1254.6932	15.4	0	57	4.6e-005	1	U	K.LAADTPLLTGQR.V
13	1675.7470	1674.7397	1674.7308	5.34	0	91	1.4e-008	1	U	K.YSNSDAVVYVGCGER.G
15	1733.8905	1732.8832	1732.8672	9.27	0	60	2.5e-005	1	U	R.EASIYTGITIAEYFR.D
22	1811.8694	1810.8622	1810.8559	3.43	0	60	2.1e-005	1	U	R.LAEMPADSGYPAYLAAR.L + Oxidation (M)

Spot 523

NCBInr protein database

Match to: gi|336112678 Mass: 68845 Score: 671 Matches: 13(8) Sequences: 11(6)

V-type ATPase subunit A [*Salicornia europaea*]

Matched peptides shown in bold red.

1 MPSVYADRMT TFEDSEKESE YGYIRKVSGP VVVADGMAGA AMYELVRVGN

51 DNLIGEIIRL EGDSATIQVY EETGGLTVND PVLRTHKPLS VELGPGILGN

101 IFDGIQRPLK TIAIKSGDVY IPRGVSVPPL DKDALWDFQP NKLGEGDLLT

151 GGDLYAIVDE NTLMKHHVAL PPDAMGKITY IAPPGQYSIM DTVLELEFQG

201 VVKKFTMLQT WPVRTPRPVS SKLAADTPLL TGQRVLDALF PSVLGGTCAI

251 PGAFGCGKTV ISQALSKYSN SDAVVYVGCG ERGNEMAEVL MDFPQLTMTL

301 PDGREESVMK RTTLVANTSN MPVAAREASI YTGITIAEYF RDMGNNVSMM

351 ADSTSRWAEA LREISGRLAE MPADSGYPAY LAARLASFYE RAGKVKCLGG

401 PERNGSVTIV GAVSPPGGDF SDPVTSATLS IVQVFWGLDK KLAQRKHFPS

451 VNWLISYSKY STALESFYEK FDPEFIDIRT KAREVLQRED DLNEIVQLVG

501 KDALAETDKI TLDTAKLLRE DYLAQNAFTA YDKFCPFYKS VWMMRNIIHF

551 YNLANQAVER GAGSDGQKIT YSLIKHKLGD LFYRLVSQKF EDPAEGEDTL

601 VAKFKKLNDD LSASFRNLED ETR

Query	Observed	Mr(expt)	Mr(calc)	ppm	Miss	Score	Expect	Rank	Unique	Peptide
11	1137.5515	1136.5442	1136.5462	-1.74	0	16	76	7	U	K.LNDDLSASFR.N
12	1151.5694	1150.5621	1150.5659	-3.23	0	43	0.19	1	U	K.FDPEFIDIR.T
14	1255.7007	1254.6935	1254.6932	0.21	0	63	0.0013	1		K.LAADTPLLTGQR.V
22	1312.7212	1311.7139	1311.7146	-0.55	0	41	0.24	1	U	R.VGNDNLIGEIIR.L
28	1545.8053	1544.7981	1544.7981	-0.00	0	20	30	2		R.TTLVANTSNMPVAAR.E
30	1675.7359	1674.7286	1674.7308	-1.30	0	94	7.4e-07	1		K.YSNSDAVVYVGCGER.G
31	1733.8814	1732.8741	1732.8672	3.98	0	82	1.4e-05	1		R.EASIYTGITIAEYFR.D
34	1795.8644	1794.8571	1794.8610	-2.16	0	80	2.2e-05	1		R.LAEMPADSGYPAYLAAR.L
36	1811.8661	1810.8588	1810.8559	1.58	0	(54)	0.0092	1		R.LAEMPADSGYPAYLAAR.L + Oxidation (M)
40	2076.0610	2075.0538	2075.0423	5.55	1	123	1.1e-09	1	U	R.LVSQKFEDPAEGEDTLVAK.F
41	2084.9146	2083.9073	2083.9044	1.38	1	(48)	0.025	1	U	R.MTTFEDSEKESEYGYIR.K

44	2100.9104	2099.9031	2099.8993	1.81	1	92	1.1e-06	1	U	R.MTTFEDSEKESEYGYIR.K + Oxidation (M)
54	2470.2173	2469.2100	2469.1740	14.6	1	19	17	3	U	K.YSTALESFYEKFDPEFIDIR.T

2. gi|2506211 Mass: 68923 Score: 339 Matches: 6(5) Sequences: 5(4)

RecName: Full=V-type proton ATPase catalytic subunit A; Short=V-ATPase subunit A; AltName: Full=V-ATPase 69 kDa subunit; AltName: Full=VAA3-1; AltName: Full=Vacuolar proton pump subunit alpha

Query	Observed	Mr(expt)	Mr(calc)	ppm	Miss	Score	Expect	Rank	Unique	Peptide
14	1255.7007	1254.6935	1254.6932	0.21	0	63	0.0013	1		K.LAADTPLLTGQR.V
28	1545.8053	1544.7981	1544.7981	-0.00	0	20	30	2		R.TTLVANTSNMPVAAR.E
30	1675.7359	1674.7286	1674.7308	-1.30	0	94	7.4e-07	1		K.YSNSDAVVYVGCGER.G
31	1733.8814	1732.8741	1732.8672	3.98	0	82	1.4e-05	1		R.EASIYTGITLAEYFR.D
34	1795.8644	1794.8571	1794.8610	-2.16	0	80	2.2e-05	1		R.LAEMPADSGYPAYLAAR.L
36	1811.8661	1810.8588	1810.8559	1.58	0	(54)	0.0092	1		R.LAEMPADSGYPAYLAAR.L + Oxidation (M)

Proteins matching the same set of peptides:

gi|66816974 Mass: 41510 Score: 339 Matches: 6(5) Sequences: 5(4)

vacuolar H+-ATPase subunit A [*Vigna unguiculata*]

gi|356521645 Mass: 69021 Score: 339 Matches: 6(5) Sequences: 5(4)

PREDICTED: V-type proton ATPase catalytic subunit A-like [*Glycine max*]

gi|363806992 Mass: 69021 Score: 339 Matches: 6(5) Sequences: 5(4)

V-type proton ATPase catalytic subunit A-like [*Glycine max*]

3. gi|147791359 Mass: 73274 Score: 333 Matches: 6(5) Sequences: 5(4)

hypothetical protein VITISV_008123 [*Vitis vinifera*]

Query	Observed	Mr(expt)	Mr(calc)	ppm	Miss	Score	Expect	Rank	Unique	Peptide
14	1255.7007	1254.6935	1254.6932	0.21	0	63	0.0013	1		K.LAADTPLLTGQR.V
28	1545.8053	1544.7981	1544.7981	-0.00	0	20	30	2		R.TTLVANTSNMPVAAR.E
31	1733.8814	1732.8741	1732.8672	3.98	0	82	1.4e-05	1		R.EASIYTGITIAEYFR.D
34	1795.8644	1794.8571	1794.8610	-2.16	0	80	2.2e-05	1		R.LAEMPADSGYPAYLAAR.L
36	1811.8661	1810.8588	1810.8559	1.58	0	(54)	0.0092	1		R.LAEMPADSGYPAYLAAR.L + Oxidation (M)
40	2076.0610	2075.0538	2075.0423	5.55	1	88	3.2e-06	2	U	R.LVSQKFEDPAEGEDALVTK.F

4. gi|514709242 Mass: 68637 Score: 245 Matches: 5(4) Sequences: 4(3)

PREDICTED: V-type proton ATPase catalytic subunit A-like [*Setaria italica*]

Query	Observed	Mr(expt)	Mr(calc)	ppm	Miss	Score	Expect	Rank	Unique	Peptide
14	1255.7007	1254.6935	1254.6932	0.21	0	63	0.0013	1	U	K.IAADTPLLTGQR.V
28	1545.8053	1544.7981	1544.7981	-0.00	0	20	30	2		R.TTLVANTSNMPVAAR.E
31	1733.8814	1732.8741	1732.8672	3.98	0	82	1.4e-05	1		R.EASIYTGITIAEYFR.D
34	1795.8644	1794.8571	1794.8610	-2.16	0	80	2.2e-05	1		R.LAEMPADSGYPAYLAAR.L
36	1811.8661	1810.8588	1810.8559	1.58	0	(54)	0.0092	1		R.LAEMPADSGYPAYLAAR.L + Oxidation (M)

5. gi|294460350 Mass: 68744 Score: 201 Matches: 5(3) Sequences: 4(2)

unknown [*Picea sitchensis*]

Query	Observed	Mr(expt)	Mr(calc)	ppm	Miss	Score	Expect	Rank	Unique	Peptide
28	1545.8053	1544.7981	1544.7981	-0.00	0	20	30	2		R.TTLVANTSNMPVAAR.E
31	1733.8814	1732.8741	1732.8672	3.98	0	82	1.4e-05	1		R.EASIYTGITLAEYFR.D
34	1795.8644	1794.8571	1794.8610	-2.16	0	80	2.2e-05	1		R.LAEMPADSGYPAYLAAR.L
36	1811.8661	1810.8588	1810.8559	1.58	0	(54)	0.0092	1		R.LAEMPADSGYPAYLAAR.L + Oxidation (M)
44	2100.9104	2099.9031	2099.8994	1.81	1	19	18	2	U	K.MTTFEDTEKESEYGYVR.K + Oxidation (M)

6. gi|255080478 Mass: 68480 Score: 174 Matches: 3(2) Sequences: 3(2)

H+-or Na+-translocating f-type, v-type and A-type ATPase superfamily [*Micromonas sp. RCC299*]

Query	Observed	Mr(expt)	Mr(calc)	ppm	Miss	Score	Expect	Rank	Unique	Peptide
28	1545.8053	1544.7981	1544.7981	-0.00	0	20	30	2		R.TTLVANTSNMPVAAR.E
30	1675.7359	1674.7286	1674.7308	-1.30	0	74	7.6e-05	2	U	K.YSNSDGIVYVGCGER.G
31	1733.8814	1732.8741	1732.8672	3.98	0	82	1.4e-05	1		R.EASIYTGITLAEYFR.D

Database 1

Match to: Unigene 18901_Se200S transcribed RNA sequence Mass: 23700 Score: 276 Matches: 5(4) Sequences: 4(3)

gi|222424801 AT1G78900 [*Arabidopsis thaliana*]

Matched peptides shown in bold red.

TVISQALSKYSNSDAVVYVGCGERGNEMAEVLMDFPQLTMTLPDGREESVMKRTTLVANTSNMPVAAREASIYTGITIAEYF
RDMGNNVSMMADSTSRWAEALREISGRLAEMPADSGYPAYLAARLASFYERAGKVKCLGGPERNGSVTIVGAVSPPGGDFS
DPVTSATLSIVQVFWGLDKKLAQRKHFPSVNWLISYSKYSTALESFYEKFDPE

Query	Observed	Mr(expt)	Mr(calc)	ppm	Miss	Score	Expect	Rank	Unique	Peptide
28	1545.8053	1544.7981	1544.7981	-0.00	0	20	0.59	1	U	R.TTLVANTSNMPVAAR.E
30	1675.7359	1674.7286	1674.7308	-1.30	0	94	8.7e-009	1	U	K.YSNSDAVVYVGCGER.G
31	1733.8814	1732.8741	1732.8672	3.98	0	82	3.1e-007	1	U	R.EASIYTGITIAEYFR.D
34	1795.8644	1794.8571	1794.8610	-2.16	0	80	4.8e-007	1	U	R.LAEMPADSGYPAYLAAR.L
36	1811.8661	1810.8588	1810.8559	1.58	0	(54)	0.00019	1	U	R.LAEMPADSGYPAYLAAR.L + Oxidation (M)

Proteins matching the same set of peptides:

Unigene45737_SeCKS transcribed RNA sequence Mass: 22692 Score: 276 Matches: 5(4) Sequences: 4(3)

gi|222424801 AT1G78900 [*Arabidopsis thaliana*]

2. Unigene12325_SeCKS transcribed RNA sequence Mass: 27816 Score: 205 Matches: 5(4) Sequences: 4(3)

gi|336112678 V-type ATPase subunit A [*Salicornia europaea*]

Query	Observed	Mr(expt)	Mr(calc)	ppm	Miss	Score	Expect	Rank	Unique	Peptide
9	1016.4677	1015.4605	1015.4610	-0.58	0	10	3.5	1	U	K.ESEYGYIR.K
14	1255.7007	1254.6935	1254.6932	0.21	0	63	2.1e-005	1	U	K.LAADTPLLTGQR.V
22	1312.7212	1311.7139	1311.7146	-0.55	0	41	0.0044	1	U	R.VGNDNLIGEIIR.L
41	2084.9146	2083.9073	2083.9044	1.38	1	(48)	0.00034	1	U	R.MTTFEDSEKESEYGYIR.K
44	2100.9104	2099.9031	2099.8993	1.81	1	92	1.3e-008	1	U	R.MTTFEDSEKESEYGYIR.K + Oxidation (M)

Proteins matching the same set of peptides:

Unigene47073_Se200S transcribed RNA sequence Mass: 28308 Score: 205 Matches: 5(4) Sequences: 4(3)

gi|336112678 V-type ATPase subunit A [*Salicornia europaea*]

3. Unigene46998_Se200S transcribed RNA sequence Mass: 18203 Score: 189 Matches: 4(2) Sequences: 4(2)

gi|336112678 V-type ATPase subunit A [*Salicornia europaea*]

Query	Observed	Mr(expt)	Mr(calc)	ppm	Miss	Score	Expect	Rank	Unique	Peptide
11	1137.5515	1136.5442	1136.5462	-1.74	0	16	1.4	1	U	K.LNDDLSASFR.N
12	1151.5694	1150.5622	1150.5659	-3.23	0	43	0.0027	1		K.FDPEFIDIR.T
15	1265.6476	1264.6403	1264.6411	-0.64	1	7	14	3	U	K.KLNDDLSASFR.N
40	2076.0610	2075.0538	2075.0423	5.55	1	123	2.3e-011	1		R.LVSQKFEDPAEGEDTLVAK.F

4. Unigene45081_SeCKS transcribed RNA sequence Mass: 16998 Score: 185 Matches: 3(2) Sequences: 3(2)

gi|336112678 V-type ATPase subunit A [*Salicornia europaea*]

Query	Observed	Mr(expt)	Mr(calc)	ppm	Miss	Score	Expect	Rank	Unique	Peptide
12	1151.5694	1150.5622	1150.5659	-3.23	0	43	0.0027	1		K.FDPEFIDIR.T
40	2076.0610	2075.0538	2075.0423	5.55	1	123	2.3e-011	1		R.LVSQKFEDPAEGEDTLVAK.F
54	2470.2173	2469.2100	2469.1740	14.6	1	19	0.41	1	U	K.YSTALESFYEKFDPEFIDIR.T

Database 2

Match to: Unigene52395_SALfmcTARAAPEI-3 Mass: 29936 Score: 293 Matches: 8(6) Sequences: 7(5)

gi|336112678 V-type ATPase subunit A [*Salicornia europaea*]

Matched peptides shown in bold red.

PVSSKLAADTPLLTGQRVLDALFPSVLGGTCAIPGAFGCGKTVISQALSKYSNSDAVVYVGCGERGNEMAEVLMDFPQLTMTL
PDGREESVMKRTTLVANTSNMPVAAREASIYTGITIAEYFRDMGNNVSMMADSTSRWAEALREISGRLAEMPADSGYPAYLAA
RLASFYERAGKVKCLGGPERNGSVTIVGAVSPPGGDFSDPVTSATLSIVQVFWGLDKKLAQRKHFPSVNWLISY

Query	Observed	Mr(expt)	Mr(calc)	ppm	Miss	Score	Expect	Rank	Unique	Peptide
12	1151.5694	1150.5622	1150.5659	-3.23	0	43	0.0014	1	U	K.FDPEFIDIR.T
14	1255.7007	1254.6935	1254.6932	0.21	0	63	1.2e-005	1	U	K.LAADTPLLTGQR.V
28	1545.8053	1544.7981	1544.7981	-0.00	0	20	0.29	1	U	R.TTLVANTSNMPVAAR.E
30	1675.7359	1674.7286	1674.7308	-1.30	0	94	6.9e-009	1	U	K.YSNSDAVVYVGCGER.G
31	1733.8814	1732.8741	1732.8672	3.98	0	82	1.5e-007	1	U	R.EASIYTGITIAEYFR.D
34	1795.8644	1794.8571	1794.8610	-2.16	0	80	2.1e-007	1	U	R.LAEMPADSGYPAYLAAR.L
36	1811.8661	1810.8588	1810.8559	1.58	0	(54)	8.3e-005	1	U	R.LAEMPADSGYPAYLAAR.L + Oxidation (M)
54	2470.2173	2469.2100	2469.1740	14.6	1	19	0.16	1	U	K.YSTALESFYEKFDPEFIDIR.T

Spot 535

NCBInr protein database

Match to: gi|336112678 Mass: 68845 Score: 437 Matches: 8(5) Sequences: 8(5)

V-type ATPase subunit A [*Salicornia europaea*]

Matched peptides shown in bold red.

1 MPSVYADRMT TFEDSEKESE YGYIRKVSGP VVVADGMAGA AMYELVRVGN

51 DNLIGEIIRL EGDSATIQVY EETGGLTVND PVLRTHKPLS VELGPGILGN

101 IFDGIQRPLK TIAIKSGDVY IPRGVSVPPL DKDALWDFQP NKLGEGDLLT

151 GGDLYAIVDE NTLMKHHVAL PPDAMGKITY IAPPGQYSIM DTVLELEFQG

201 VVKKFTMLQT WPVRTPRPVS SKLAADTPLL TGQRVLDALF PSVLGGTCAI

251 PGAFGCGKTV ISQALSKYSN SDAVVYVGCG ERGNEMAEVL MDFPQLTMTL

301 PDGREESVMK RTTLVANTSN MPVAAREASI YTGITIAEYF RDMGNNVSMM

351 ADSTSRWAEA LREISGRLAE MPADSGYPAY LAARLASFYE RAGKVKCLGG

401 PERNGSVTIV GAVSPPGGDF SDPVTSATLS IVQVFWGLDK KLAQRKHFPS

451 VNWLISYSKY STALESFYEK FDPEFIDIRT KAREVLQRED DLNEIVQLVG

501 KDALAETDKI TLDTAKLLRE DYLAQNAFTA YDKFCPFYKS VWMMRNIIHF

551 YNLANQAVER GAGSDGQKIT YSLIKHKLGD LFYRLVSQKF EDPAEGEDTL

601 VAKFKKLNDD LSASFRNLED ETR

Query	Observed	Mr(expt)	Mr(calc)	ppm	Miss	Score	Expect	Rank	Unique	Peptide
8	1016.4851	1015.4778	1015.4610	16.5	0	39	0.46	1		K.ESEYGYIR.K
14	1151.5876	1150.5803	1150.5659	12.6	0	41	0.28	1	U	K.FDPEFIDIR.T
17	1255.7117	1254.7044	1254.6932	8.94	0	59	0.0029	1		K.LAADTPLLTGQR.V
21	1312.7345	1311.7272	1311.7146	9.60	0	66	0.00062	1	U	R.VGNDNLIGEIIR.L
33	1675.7487	1674.7414	1674.7308	6.33	0	90	2e-06	1		K.YSNSDAVVYVGCGER.G
35	1733.8924	1732.8851	1732.8672	10.4	0	47	0.046	1		R.EASIYTGITIAEYFR.D
38	1801.9495	1800.9422	1800.9271	8.40	0	36	0.54	1		R.NIIHFYNLANQAVER.G
39	1811.8748	1810.8675	1810.8559	6.37	0	58	0.0034	1		R.LAEMPADSGYPAYLAAR.L + Oxidation (M)

2. gi|13235340 Mass: 69139 Score: 330 Matches: 6(4) Sequences: 6(4)

putative vacuolar ATP Synthase subunit A [*Mesembryanthemum crystallinum*]

Query	Observed	Mr(expt)	Mr(calc)	ppm	Miss	Score	Expect	Rank	Unique	Peptide
8	1016.4851	1015.4778	1015.4610	16.5	0	39	0.46	1		K.ESEYGYIR.K
17	1255.7117	1254.7044	1254.6932	8.94	0	59	0.0029	1		K.LAADTPLLTGQR.V
33	1675.7487	1674.7414	1674.7308	6.33	0	90	2e-06	1		K.YSNSDAVVYVGCGER.G
35	1733.8924	1732.8851	1732.8672	10.4	0	47	0.046	1		R.EASIYTGITIAEYFR.D
38	1801.9495	1800.9422	1800.9635	-11.80	1	36	0.54	1	U	R.NIIHFYNLANKAVER.G
39	1811.8748	1810.8675	1810.8559	6.37	0	58	0.0034	1		R.LAEMPADSGYPAYLAAR.L + Oxidation (M)

3. gi|2506211 Mass: 68923 Score: 291 Matches: 5(4) Sequences: 5(4)

RecName: Full=V-type proton ATPase catalytic subunit A; Short=V-ATPase subunit A; AltName: Full=V-ATPase 69 kDa subunit; AltName: Full=VAA3-1; AltName: Full=Vacuolar proton pump subunit alpha

Query	Observed	Mr(expt)	Mr(calc)	ppm	Miss	Score	Expect	Rank	Unique	Peptide
17	1255.7117	1254.7044	1254.6932	8.94	0	59	0.0029	1		K.LAADTPLLTGQR.V
33	1675.7487	1674.7414	1674.7308	6.33	0	90	2e-06	1		K.YSNSDAVVYVGCGER.G
35	1733.8924	1732.8851	1732.8672	10.4	0	47	0.046	1		R.EASIYTGITLAEYFR.D
38	1801.9495	1800.9422	1800.9271	8.40	0	36	0.54	1		R.NIIHFYNLANQAVER.G
39	1811.8748	1810.8675	1810.8559	6.37	0	58	0.0034	1		R.LAEMPADSGYPAYLAAR.L + Oxidation (M)

Proteins matching the same set of peptides:

gi|356521645 Mass: 69021 Score: 291 Matches: 5(4) Sequences: 5(4)

PREDICTED: V-type proton ATPase catalytic subunit A-like [*Glycine max*]

gi|363806992 Mass: 69021 Score: 291 Matches: 5(4) Sequences: 5(4)

V-type proton ATPase catalytic subunit A-like [*Glycine max*]

4. gi|350537129 Mass: 68812 Score: 239 Matches: 5(3) Sequences: 5(3)

vacuolar H+-ATPase A1 subunit isoform [*Solanum lycopersicum*]

Query	Observed	Mr(expt)	Mr(calc)	ppm	Miss	Score	Expect	Rank	Unique	Peptide
8	1016.4851	1015.4778	1015.4610	16.5	0	39	0.46	1	U	K.ESEYGYLR.K
17	1255.7117	1254.7044	1254.6932	8.94	0	59	0.0029	1		K.LAADTPLLTGQR.V
35	1733.8924	1732.8851	1732.8672	10.4	0	47	0.046	1		R.EASIYTGITIAEYFR.D
38	1801.9495	1800.9422	1800.9271	8.40	0	36	0.54	1		R.NIIHFYNLANQAVER.G
39	1811.8748	1810.8675	1810.8559	6.37	0	58	0.0034	1		R.LAEMPADSGYPAYLAAR.L + Oxidation (M)

5. gi|60592632 Mass: 68999 Score: 192 Matches: 4(3) Sequences: 4(3)

vacuolar H+-ATPase catalytic subunit [*Pyrus communis*]

Query	Observed	Mr(expt)	Mr(calc)	ppm	Miss	Score	Expect	Rank	Unique	Peptide
17	1255.7117	1254.7044	1254.6932	8.94	0	59	0.0029	1		K.LAADTPLLTGQR.V
35	1733.8924	1732.8851	1732.8672	10.4	0	47	0.046	1		R.EASIYTGITIAEYFR.D
38	1801.9495	1800.9422	1800.9271	8.40	0	28	3.7	4	U	R.NIIHYFNLANQAVER.A
39	1811.8748	1810.8675	1810.8559	6.37	0	58	0.0034	1		R.LAEMPADSGYPAYLAAR.L + Oxidation (M)

Proteins matching the same set of peptides:

gi|131573315 Mass: 69161 Score: 192 Matches: 4(3) Sequences: 4(3)

vacuolar H+-ATPase [Malus domestica]

6. gi|514709242 Mass: 68637 Score: 164 Matches: 3(3) Sequences: 3(3)

PREDICTED: V-type proton ATPase catalytic subunit A-like [*Setaria italica*]

Query	Observed	Mr(expt)	Mr(calc)	ppm	Miss	Score	Expect	Rank	Unique	Peptide
17	1255.7117	1254.7044	1254.6932	8.94	0	59	0.0029	1	U	K.IAADTPLLTGQR.V
35	1733.8924	1732.8851	1732.8672	10.4	0	47	0.046	1		R.EASIYTGITIAEYFR.D
39	1811.8748	1810.8675	1810.8559	6.37	0	58	0.0034	1		R.LAEMPADSGYPAYLAAR.L + Oxidation (M)

7. gi|255080478 Mass: 68480 Score: 116 Matches: 2(2) Sequences: 2(2)

H+-or Na+-translocating f-type, v-type and A-type ATPase superfamily [*Micromonas sp. RCC299*]

Query	Observed	Mr(expt)	Mr(calc)	ppm	Miss	Score	Expect	Rank	Unique	Peptide
33	1675.7487	1674.7414	1674.7308	6.33	0	71	0.00017	2	U	K.YSNSDGIVYVGCGER.G
35	1733.8924	1732.8851	1732.8672	10.4	0	47	0.046	1		R.EASIYTGITLAEYFR.D

8. gi|5869971 Mass: 67976 Score: 105 Matches: 2(2) Sequences: 2(2)

H(+)-transporting ATP synthase [*Scherffelia dubia*]

Query	Observed	Mr(expt)	Mr(calc)	ppm	Miss	Score	Expect	Rank	Unique	Peptide
17	1255.7117	1254.7044	1254.7296	-20.06	0	58	0.0041	3	U	K.LLGSTPLLTGQR.V
35	1733.8924	1732.8851	1732.8672	10.4	0	47	0.046	1		R.EASIYTGITLAEYFR.D

Database 1

Match to: Unigene 18901_Se200S transcribed RNA sequence Mass: 23700 Score: 195 Matches: 3(3) Sequences: 3(3)

Score: 195

gi|222424801 AT1G78900 [*Arabidopsis thaliana*]

Matched peptides shown in bold red.

TVISQALSK**YSNSDAVVYVGCGER**GNEMAEVLMDFPQLTMTLPDGREESVMKRTTLVANTSNMPVAAR**EASIYTGITIAEYF**
RDMGNNVSMMADSTSRWAEALREISGR**LAEMPADSGYPAYLAAR**LASFYERAGKVKCLGGPERNGSVTIVGAVSPPGGDFS
DPVTSATLSIVQVFWGLDKKLAQRKHFPSVNWLISYSKYSTALESFYEKFDPE

Query	Observed	Mr(expt)	Mr(calc)	ppm	Miss	Score	Expect	Rank	Unique	Peptide
33	1675.7487	1674.7414	1674.7308	6.33	0	90	2.6e-008	1	U	K.YSNSDAVVYVGCGER.G
35	1733.8924	1732.8851	1732.8672	10.4	0	47	0.001	1	U	R.EASIYTGITIAEYFR.D
39	1811.8748	1810.8675	1810.8559	6.37	0	58	7.3e-005	1	U	R.LAEMPADSGYPAYLAAR.L + Oxidation (M)

Proteins matching the same set of peptides:

Unigene45737_SeCKS transcribed RNA sequence Mass: 22692 Score: 195 Matches: 3(3) Sequences: 3(3)

gi|222424801 AT1G78900 [*Arabidopsis thaliana*]

2. Unigene12325_SeCKS transcribed RNA sequence Mass: 27816 Score: 164 Matches: 3(3) Sequences: 3(3)

gi|336112678 V-type ATPase subunit A [*Salicornia europaea*]

Query	Observed	Mr(expt)	Mr(calc)	ppm	Miss	Score	Expect	Rank	Unique	Peptide
8	1016.4851	1015.4778	1015.4610	16.5	0	39	0.0058	1	U	K.ESEYGYIR.K
17	1255.7117	1254.7044	1254.6932	8.94	0	59	4.4e-005	1	U	K.LAADTPLLTGQR.V
21	1312.7345	1311.7272	1311.7146	9.60	0	66	1.1e-005	1	U	R.VGNDNLIGEIIR.L

Proteins matching the same set of peptides:

Unigene47073_Se200S transcribed RNA sequence Mass: 28308 Score: 164 Matches: 3(3) Sequences: 3(3)

gi|336112678 V-type ATPase subunit A [*Salicornia europaea*]

3. Unigene45081_SeCKS transcribed RNA sequence Mass: 16998 Score: 78 Matches: 2(2) Sequences: 2(2)

gi|336112678 V-type ATPase subunit A [*Salicornia europaea*]

Query	Observed	Mr(expt)	Mr(calc)	ppm	Miss	Score	Expect	Rank	Unique	Peptide
14	1151.5876	1150.5803	1150.5659	12.6	0	41	0.0044	1	U	K.FDPEFIDIR.T
38	1801.9495	1800.9422	1800.9271	8.40	0	36	0.011	1	U	R.NIIHFYNLANQAVER.G

Proteins matching the same set of peptides:

Unigene46998_Se200S transcribed RNA sequence Mass: 18203 Score: 78 Matches: 2(2) Sequences: 2(2)

gi|336112678 V-type ATPase subunit A [*Salicornia europaea*]

Database 2

Match to: Unigene52395_SALfmcTARAAPEI-3 Mass: 29936 Score: 201 Matches: 5(5) Sequences: 5(5)

gi|336112678 V-type ATPase subunit A [*Salicornia europaea*]

Matched peptides shown in bold red.

PVSSK**LAADTPLLTGQR**VLDALFPSVLGGTCAIPGAFGCGKTVISQALSK**YSNSDAVVYVGCGER**GNEMAEVLMDFPQLTMTL
PDGREESVMKRTTLVANTSNMPVAAR**EASIYTGITIAEYFR**DMGNNVSMMADSTSRWAEALREISGR**LAEMPADSGYPAYLAA**
RLASFYERAGKVKCLGGPERNGSVTIVGAVSPPGGDFSDPVTSATLSIVQVFWGLDKKLAQRKHFPSVNWLISY

Query	Observed	Mr(expt)	Mr(calc)	ppm	Miss	Score	Expect	Rank	Unique	Peptide
14	1151.5876	1150.5803	1150.5659	12.6	0	41	0.002	1	U	K.FDPEFIDIR.T
17	1255.7117	1254.7044	1254.6932	8.94	0	59	2.7e-005	1	U	K.LAADTPLLTGQR.V
33	1675.7487	1674.7414	1674.7308	6.33	0	90	1.8e-008	1	U	K.YSNSDAVVYVGCGER.G
35	1733.8924	1732.8851	1732.8672	10.4	0	47	0.00048	1	U	R.EASIYTGITIAEYFR.D
39	1811.8748	1810.8675	1810.8559	6.37	0	58	3.1e-005	1	U	R.LAEMPADSGYPAYLAAR.L

+ Oxidation (M)

Spot 635

NCBInr protein database

Match to: gi|336112678 Mass: 68845 Score: 510 Matches: 9(6) Sequences: 8(5)

gi|336112678 V-type ATPase subunit A [*Salicornia europaea*]

Matched peptides shown in bold red.

1 MPSVYADRMT TFEDSEKESE YGYIRKVSGP VVVADGMAGA AMYELVRVGN

51 DNLIGEIIRL EGDSATIQVY EETGGLTVND PVLRTHKPLS VELGPGILGN

101 IFDGIQRPLK TIAIKSGDVY IPRGVSVPPL DKDALWDFQP NKLGEGDLLT

151 GGDLYAIVDE NTLMKHHVAL PPDAMGKITY IAPPGQYSIM DTVLELEFQG

201 VVKKFTMLQT WPVRTPRPVS SKLAADTPLL TGQRVLDALF PSVLGGTCAI

251 PGAFGCGKTV ISQALSKYSN SDAVVYVGCG ERGNEMAEVL MDFPQLTMTL

301 PDGREESVMK RTTLVANTSN MPVAAREASI YTGITIAEYF RDMGNNVSMM

351 ADSTSRWAEA LREISGRLAE MPADSGYPAY LAARLASFYE RAGKVKCLGG

401 PERNGSVTIV GAVSPPGGDF SDPVTSATLS IVQVFWGLDK KLAQRKHFPS

451 VNWLISYSKY STALESFYEK FDPEFIDIRT KAREVLQRED DLNEIVQLVG

501 KDALAETDKI TLDTAKLLRE DYLAQNAFTA YDKFCPFYKS VWMMRNIIHF

551 YNLANQAVER GAGSDGQKIT YSLIKHKLGD LFYRLVSQKF EDPAEGEDTL

601 VAKFKKLNDD LSASFRNLED ETR

Query	Observed	Mr(expt)	Mr(calc)	ppm	Miss	Score	Expect	Rank	Unique	Peptide
8	883.4708	882.4635	882.4599	4.08	0	34	1.1	1		K.LGDLFYR.L
12	1151.5796	1150.5723	1150.5659	5.61	0	46	0.094	1	U	K.FDPEFIDIR.T
14	1255.7113	1254.7040	1254.6932	8.61	0	50	0.024	1		K.LAADTPLLTGQR.V
25	1545.8110	1544.8038	1544.7981	3.68	0	12	1.6e+02	7		R.TTLVANTSNMPVAAR.E
29	1675.7532	1674.7459	1674.7308	9.01	0	102	1.4e-07	1		K.YSNSDAVVYVGCGER.G
30	1733.8986	1732.8913	1732.8672	13.9	0	67	0.00051	1		R.EASIYTGITIAEYFR.D
33	1795.8860	1794.8787	1794.8610	9.86	0	92	1.3e-06	1		R.LAEMPADSGYPAYLAAR.L
35	1811.8828	1810.8755	1810.8559	10.8	0	(57)	0.0044	1		R.LAEMPADSGYPAYLAAR.L + Oxidation (M)
36	2076.0780	2075.0707	2075.0423	13.7	1	109	2.7e-08	1	U	R.LVSQKFEDPAEGEDTLVAK.F

2. gi|147791359 Mass: 73274 Score: 330 Matches: 7(5) Sequences: 6(4)

hypothetical protein VITISV_008123 [*Vitis vinifera*]

Query	Observed	Mr(expt)	Mr(calc)	ppm	Miss	Score	Expect	Rank	Unique	Peptide
8	883.4708	882.4635	882.4599	4.08	0	34	1.1	1		R.LGDLFYR.L
14	1255.7113	1254.7040	1254.6932	8.61	0	50	0.024	1		K.LAADTPLLTGQR.V
25	1545.8110	1544.8038	1544.7981	3.68	0	12	1.6e+02	7		R.TTLVANTSNMPVAAR.E
30	1733.8986	1732.8913	1732.8672	13.9	0	67	0.00051	1		R.EASIYTGITIAEYFR.D
33	1795.8860	1794.8787	1794.8610	9.86	0	92	1.3e-06	1		R.LAEMPADSGYPAYLAAR.L
35	1811.8828	1810.8755	1810.8559	10.8	0	(57)	0.0044	1		R.LAEMPADSGYPAYLAAR.L + Oxidation (M)
36	2076.0780	2075.0707	2075.0423	13.7	1	75	6.2e-05	2	U	R.LVSQKFEDPAEGEDALVTK.F

3. gi|2506211 Mass: 68923 Score: 322 Matches: 6(5) Sequences: 5(4)

RecName: Full=V-type proton ATPase catalytic subunit A; Short=V-ATPase subunit A; AltName: Full=V-ATPase 69 kDa subunit; AltName: Full=VAA3-1; AltName: Full=Vacuolar proton pump subunit alpha

Query	Observed	Mr(expt)	Mr(calc)	ppm	Miss	Score	Expect	Rank	Unique	Peptide
14	1255.7113	1254.7040	1254.6932	8.61	0	50	0.024	1		K.LAADTPLLTGQR.V
25	1545.8110	1544.8038	1544.7981	3.68	0	12	1.6e+02	7		R.TTLVANTSNMPVAAR.E
29	1675.7532	1674.7459	1674.7308	9.01	0	102	1.4e-07	1		K.YSNSDAVVYVGCGER.G
30	1733.8986	1732.8913	1732.8672	13.9	0	65	0.00069	2		R.EASIYTGITLAEYFR.D
33	1795.8860	1794.8787	1794.8610	9.86	0	92	1.3e-06	1		R.LAEMPADSGYPAYLAAR.L
35	1811.8828	1810.8755	1810.8559	10.8	0	(57)	0.0044	1		R.LAEMPADSGYPAYLAAR.L + Oxidation (M)

Proteins matching the same set of peptides:

gi|66816974 Mass: 41510 Score: 322 Matches: 6(5) Sequences: 5(4)

vacuolar H+-ATPase subunit A [*Vigna unguiculata*]

gi|356521645 Mass: 69021 Score: 322 Matches: 6(5) Sequences: 5(4)

PREDICTED: V-type proton ATPase catalytic subunit A-like [*Glycine max*]

gi|363806992 Mass: 69021 Score: 322 Matches: 6(5) Sequences: 5(4)

V-type proton ATPase catalytic subunit A-like [*Glycine max*]

4. gi|514709242 Mass: 68637 Score: 255 Matches: 6(4) Sequences: 5(3)

PREDICTED: V-type proton ATPase catalytic subunit A-like [*Setaria italica*]

Query	Observed	Mr(expt)	Mr(calc)	ppm	Miss	Score	Expect	Rank	Unique	Peptide
8	883.4708	882.4635	882.4599	4.08	0	34	1.1	1		R.LGDLFYR.L
14	1255.7113	1254.7040	1254.6932	8.61	0	50	0.024	1	U	K.IAADTPLLTGQR.V
25	1545.8110	1544.8038	1544.7981	3.68	0	12	1.6e+02	7		R.TTLVANTSNMPVAAR.E
30	1733.8986	1732.8913	1732.8672	13.9	0	67	0.00051	1		R.EASIYTGITIAEYFR.D
33	1795.8860	1794.8787	1794.8610	9.86	0	92	1.3e-06	1		R.LAEMPADSGYPAYLAAR.L
35	1811.8828	1810.8755	1810.8559	10.8	0	(57)	0.0044	1		R.LAEMPADSGYPAYLAAR.L + Oxidation (M)

5. gi|449447990 Mass: 69062 Score: 254 Matches: 6(4) Sequences: 5(3)

PREDICTED: V-type proton ATPase catalytic subunit A-like [*Cucumis sativus*]

Query	Observed	Mr(expt)	Mr(calc)	ppm	Miss	Score	Expect	Rank	Unique	Peptide
8	883.4708	882.4635	882.4599	4.08	0	34	1.1	1		R.LGDLFYR.L
14	1255.7113	1254.7040	1254.6932	8.61	0	50	0.024	1		K.LAADTPLLTGQR.V
25	1545.8110	1544.8038	1544.7981	3.68	0	12	1.6e+02	7		R.TTLVANTSNMPVAAR.E
30	1733.8986	1732.8913	1732.8672	13.9	0	65	0.00069	2		R.EASIYTGITLAEYFR.D
33	1795.8860	1794.8787	1794.8610	9.86	0	92	1.3e-06	1		R.LAEMPADSGYPAYLAAR.L
35	1811.8828	1810.8755	1810.8559	10.8	0	(57)	0.0044	1		R.LAEMPADSGYPAYLAAR.L + Oxidation (M)

Proteins matching the same set of peptides:

gi|502149512 Mass: 68930 Score: 254 Matches: 6(4) Sequences: 5(3)

PREDICTED: V-type proton ATPase catalytic subunit A-like [*Cicer arietinum*]

6. gi|255080478 Score: 161 Matches: 3(2) Sequences: 3(2)

H+-or Na+-translocating f-type, v-type and A-type ATPase superfamily [*Micromonas sp. RCC299*]

Query	Observed	Mr(expt)	Mr(calc)	ppm	Miss	Score	Expect	Rank	Unique	Peptide
25	1545.8110	1544.8038	1544.7981	3.68	0	12	1.6e+02	7		R.TTLVANTSNMPVAAR.E

29	1675.7532	1674.7459	1674.7308	9.01	0	85	6.5e-06	2	U	K.YSNSDGIVYVGCGER.G
30	1733.8986	1732.8913	1732.8672	13.9	0	65	0.00069	2		R.EASIYTGITLAEYFR.D

7. gi|5869971 Score: 125 Matches: 3(2) Sequences: 3(2)

H(+)-transporting ATP synthase [*Scherffelia dubia*]

Query	Observed	Mr(expt)	Mr(calc)	ppm	Miss	Score	Expect	Rank	Unique	Peptide
14	1255.7113	1254.7040	1254.7296	-20.39	0	47	0.045	3	U	K.LLGSTPLLTGQR.V
25	1545.8110	1544.8038	1544.7981	3.68	0	12	1.6e+02	7		R.TTLVANTSNMPVAAR.E
30	1733.8986	1732.8913	1732.8672	13.9	0	65	0.00069	2		R.EASIYTGITLAEYFR.D

Database 1

Match to: Unigene 18901 Mass: 23700 Score: 273 Matches: 5(4) Sequences: 4(3)

gi|222424801 AT1G78900 [*Arabidopsis thaliana*]

Matched peptides shown in bold red.

TVISQALSKYSNSDAVVYVGCGERGNEMAEVLMDFPQLTMTLPDGREESVMKRTTLVANTSNMPVAAREASIYTGITIAEYF
RDMGNNVSMMADSTSRWAEALREISGRLAEMPADSGYPAYLAARLASFYERAGKVKCLGGPERNGSVTIVGAVSPPGGDFS
DPVTSATLSIVQVFWGLDKKLAQRKHFPSVNWLISYSKYSTALESFYEKFDPE

Query	Observed	Mr(expt)	Mr(calc)	ppm	Miss	Score	Expect	Rank	Unique	Peptide
25	1545.8110	1544.8038	1544.7981	3.68	0	12	3.1	2	U	R.TTLVANTSNMPVAAR.E
29	1675.7532	1674.7459	1674.7308	9.01	0	102	1.9e-009	1	U	K.YSNSDAVVYVGCGER.G
30	1733.8986	1732.8913	1732.8672	13.9	0	67	1.1e-005	1	U	R.EASIYTGITIAEYFR.D
33	1795.8860	1794.8787	1794.8610	9.86	0	92	2.9e-008	1	U	R.LAEMPADSGYPAYLAAR.L
35	1811.8828	1810.8755	1810.8559	10.8	0	(57)	9.8e-005	1	U	R.LAEMPADSGYPAYLAAR.L

+ Oxidation (M)

Proteins matching the same set of peptides:

Unigene45737_SeCKS transcribed RNA sequence Mass: 22692 Score: 273 Matches: 5(4) Sequences: 4(3)

gi|222424801 AT1G78900 [*Arabidopsis thaliana*]

2. Unigene45081_SeCKS transcribed RNA sequence Mass: 16998 Score: 188 Matches: 3(3) Sequences: 3(3)

gi|336112678 V-type ATPase subunit A [*Salicornia europaea*]

Query	Observed	Mr(expt)	Mr(calc)	ppm	Miss	Score	Expect	Rank	Unique	Peptide
8	883.4708	882.4635	882.4599	4.08	0	34	0.02	1	U	K.LGDLFYR.L
12	1151.5796	1150.5723	1150.5659	5.61	0	46	0.0014	1	U	K.FDPEFIDIR.T
36	2076.0780	2075.0707	2075.0423	13.7	1	109	5.4e-010	1	U	

R.LVSQKFEDPAEGEDTLVAK.F

Proteins matching the same set of peptides:

Unigene46998_Se200S transcribed RNA sequence Mass: 18203 Score: 188 Matches: 3(3) Sequences: 3(3)

gi|336112678 V-type ATPase subunit A [*Salicornia europaea*]

3. Unigene12325_SeCKS transcribed RNA sequence Mass: 27816 Score: 55 Matches: 2(1) Sequences: 2(1)

gi|336112678 V-type ATPase subunit A [*Salicornia europaea*]

Proteins matching the same set of peptides:

Unigene47073_Se200S transcribed RNA sequence Mass: 28308 Score: 55 Matches: 2(1) Sequences: 2(1)

gi|336112678 V-type ATPase subunit A [*Salicornia europaea*]

Database 2

Match to: Unigene52395_SALfmcTARAAPEI-3 Mass: 29936 Score: 289 Matches: 7(6) Sequences: 6(5)

gi|336112678 V-type ATPase subunit A [*Salicornia europaea*]

Matched peptides shown in bold red.

PVSSKLAADTPLLTGQRVLDALFPSVLGGTCAIPGAFGCGKTVISQALSKYSNSDAVVYVGCGERGNEMAEVLMDFPQLTMTL

PDGREESVMKRTTLVANTSNMPVAAREASIYTGITIAEYFRDMGNNVSMMADSTSRWAEALREISGRLAEMPADSGYPAYLAA

RLASFYERAGKVKCLGGPERNGSVTIVGAVSPPGGDFSDPVTSATLSIVQFVWGLDKKLAQRKHFPSVNWLISY

Query	Observed	Mr(expt)	Mr(calc)	ppm	Miss	Score	Expect	Rank	Unique	Peptide
12	1151.5796	1150.5723	1150.5659	5.61	0	46	0.00068	1	U	K.FDPEFIDIR.T
14	1255.7113	1254.7040	1254.6932	8.61	0	50	0.00022	1	U	K.LAADTPLLTGQR.V
25	1545.8110	1544.8038	1544.7981	3.68	0	12	1.5	2	U	R.TTLVANTSNMPVAAR.E
29	1675.7532	1674.7459	1674.7308	9.01	0	102	1.3e-009	1	U	K.YSNSDAVVYVGCGER.G
30	1733.8986	1732.8913	1732.8672	13.9	0	67	5.3e-006	1	U	R.EASIYTGITIAEYFR.D
33	1795.8860	1794.8787	1794.8610	9.86	0	92	1.2e-008	1	U	R.LAEMPADSGYPAYLAAR.L
35	1811.8828	1810.8755	1810.8559	10.8	0	(57)	4e-005	1	U	R.LAEMPADSGYPAYLAAR.L + Oxidation (M)

Spot 689

NCBInr protein database

Match to: gi|336112678 Mass: 68845 Score: 237 Matches: 6(2) Sequences: 6(2)

V-type ATPase subunit A [*Salicornia europaea*]

Matched peptides shown in bold red.

1 MPSVYADRMT TFEDSEKESE YGYIRKVSGP VVVADGMAGA AMYELVRVGN

51 DNLIGEIIRL EGDSATIQVY EETGGLTVND PVLRTHKPLS VELGPGILGN

101 IFDGIQRPLK TIAIKSGDVY IPRGVSVPPL DKDALWDFQP NKLGEGDLLT

151 GGDLYAIVDE NTLMKHHVAL PPDAMGKITY IAPPGQYSIM DTVLELEFQG

201 VVKKFTMLQT WPVRTPRPVS SKLAADTPLL TGQRVLDALF PSVLGGTCAI

251 PGAFGCGKTV ISQALSKYSN SDAVVYVGCG ERGNEMAEVL MDFPQLTMTL

301 PDGREESVMK RTTLVANTSN MPVAAREASI YTGITIAEYF RDMGNNVSMM

351 ADSTSRWAEA LREISGRLAE MPADSGYPAY LAARLASFYE RAGKVKCLGG

401 PERNGSVTIV GAVSPPGGDF SDPVTSATLS IVQFVWGLDK KLAQRKHFPS

451 VNWLISYSKY STALESFYEK FDPEFIDIRT KAREVLQRED DLNEIVQLVG

501 KDALAETDKI TLDTAKLLRE DYLAQNAFTA YDKFCPFYKS VWMMRNIIHF

551 YNLANQAVER GAGSDGQKIT YSLIKHKLGD LFYRLVSQKF EDPAEGEDTL

601 VAKFKKLNDD LSASFRNLED ETR

Query	Observed	Mr(expt)	Mr(calc)	ppm	Miss	Score	Expect	Rank	Unique	Peptide
5	1016.4827	1015.4754	1015.4610	14.2	0	15	1e+02	6		K.ESEYGYIR.K
10	1151.5790	1150.5718	1150.5659	5.12	0	24	15	1	U	K.FDPEFIDIR.T
16	1255.7069	1254.6997	1254.6932	5.15	0	46	0.057	1		K.LAADTPLLTGQR.V
24	1675.7443	1674.7371	1674.7308	3.75	0	57	0.0046	1		K.YSNSDAVVYVGCGER.G
26	1733.8864	1732.8792	1732.8672	6.92	0	27	5.2	1		R.EASIYTGITIAEYFR.D
29	1811.8707	1810.8634	1810.8559	4.13	0	68	0.00035	1		R.LAEMPADSGYPAYLAAR.L + Oxidation (M)

2. gi|2506211 Mass: 68923 Score: 198 Matches: 4(2) Sequences: 4(2)

RecName: Full=V-type proton ATPase catalytic subunit A; Short=V-ATPase subunit A; AltName: Full=V-ATPase 69 kDa subunit;

AltName: Full=VAA3-1; AltName: Full=Vacuolar proton pump subunit alpha

Query	Observed	Mr(expt)	Mr(calc)	ppm	Miss	Score	Expect	Rank	Unique	Peptide
16	1255.7069	1254.6997	1254.6932	5.15	0	46	0.057	1		K.LAADTPLLTGQR.V
24	1675.7443	1674.7371	1674.7308	3.75	0	57	0.0046	1		K.YSNSDAVVYVGCGER.G
26	1733.8864	1732.8792	1732.8672	6.92	0	27	5.2	1		R.EASIYTGITLAEYFR.D
29	1811.8707	1810.8634	1810.8559	4.13	0	68	0.00035	1		R.LAEMPADSGYPAYLAAR.L + Oxidation (M)

Proteins matching the same set of peptides:

gi|66816974 Mass: 41510 Score: 198 Matches: 4(2) Sequences: 4(2)

vacuolar H+-ATPase subunit A [Vigna unguiculata]

gi|356521645 Mass: 69021 Score: 198 Matches: 4(2) Sequences: 4(2)

PREDICTED: V-type proton ATPase catalytic subunit A-like [Glycine max]

gi|363806992 Mass: 69021 Score: 198 Matches: 4(2) Sequences: 4(2)

V-type proton ATPase catalytic subunit A-like [Glycine max]

3. gi|350537129 Mass: 68812 Score: 157 Matches: 4(1) Sequences: 4(1)

vacuolar H+-ATPase A1 subunit isoform [Solanum lycopersicum]

Query	Observed	Mr(expt)	Mr(calc)	ppm	Miss	Score	Expect	Rank	Unique	Peptide
5	1016.4827	1015.4754	1015.4610	14.2	0	16	83	2	U	K.ESEYGYLR.K
16	1255.7069	1254.6997	1254.6932	5.15	0	46	0.057	1		K.LAADTPLLTGQR.V
26	1733.8864	1732.8792	1732.8672	6.92	0	27	5.2	1		R.EASIYTGITIAEYFR.D
29	1811.8707	1810.8634	1810.8559	4.13	0	68	0.00035	1		R.LAEMPADSGYPAYLAAR.L + Oxidation (M)

4. gi|514709242 Mass: 68637 Score: 141 Matches: 3(1) Sequences: 3(1)

PREDICTED: V-type proton ATPase catalytic subunit A-like [Setaria italica]

Query	Observed	Mr(expt)	Mr(calc)	ppm	Miss	Score	Expect	Rank	Unique	Peptide
16	1255.7069	1254.6997	1254.6932	5.15	0	46	0.057	1	U	K.IAADTPLLTGQR.V
26	1733.8864	1732.8792	1732.8672	6.92	0	27	5.2	1		R.EASIYTGITIAEYFR.D
29	1811.8707	1810.8634	1810.8559	4.13	0	68	0.00035	1		R.LAEMPADSGYPAYLAAR.L + Oxidation (M)

Database 1

Match to: Unigene18901_Se200S transcribed RNA sequence Mass: 23700 Score: 151 Matches: 3(2) Sequences: 3(2)

gi|222424801 AT1G78900 [*Arabidopsis thaliana*]

Matched peptides shown in bold red.

TVISQALSKYSNSDAVVYVGCGERGNEMAEVLMDFPQLTMTLPDGREESVMKRTTLVANTSNMPVAAREASIYTGITIAEYFRDMGNNVSMMADSTSRWAEALREISGRLAEMPADSGYPAYLAARLASFYERAGKVKCLGGPERNGSVTIVGAVSPPGGDFSDPVTSATLSIVQVFWGLDKKLAQRKHFPSVNWLISYSKYSTALESFYEKFDPE

Query	Observed	Mr(expt)	Mr(calc)	ppm	Miss	Score	Expect	Rank	Unique	Peptide
24	1675.7443	1674.7371	1674.7308	3.75	0	57	5.7e-005	1	U	K.YSNSDAVVYVGCGER.G
26	1733.8864	1732.8792	1732.8672	6.92	0	27	0.11	1	U	R.EASIYTGITIAEYFR.D
29	1811.8707	1810.8634	1810.8559	4.13	0	68	7.4e-006	1	U	R.LAEMPADSGYPAYLAAR.L + Oxidation (M)

Proteins matching the same set of peptides:

Unigene45737_SeCKS transcribed RNA sequence Mass: 22692 Score: 151 Matches: 3(2) Sequences: 3(2)

gi|222424801 AT1G78900 [*Arabidopsis thaliana*]

Database 2

Match to: Unigene52395_SALfmcTARAAPEI-3 Mass: 29936 Score: 127 Matches: 5(4) Sequences: 5(4)

gi|336112678 V-type ATPase subunit A [*Salicornia europaea*]

Matched peptides shown in **bold red**.

PVSSK**LAADTPLLTGQR**VLDALFPSVLGGTCAIPGAFGCGKTVISQALSK**YSNSDAVVYVGCGER**GNEMAEVLMDFPQLTMTL

PDGREESVMKRTTLVANTSNMPVAAR**EASIYTGITIAEYFR**DMGNNVSMMADSTSRWAEALREISGR**LAEMPADSGYPAYLAA**

RLASFYERAGKVKCLGGPERNGSVTIVGAVSPPGGDFSDPVTSATLSIVQVFWGLDKKLAQRKHFPSVNWLISY

Query	Observed	Mr(expt)	Mr(calc)	ppm	Miss	Score	Expect	Rank	Unique	Peptide
10	1151.5790	1150.5718	1150.5659	5.12	0	24	0.11	1	U	K.FDPEFIDIR.T
16	1255.7069	1254.6997	1254.6932	5.15	0	46	0.00052	1	U	K.LAADTPLLTGQR.V
24	1675.7443	1674.7371	1674.7308	3.75	0	57	4.3e-005	1	U	K.YSNSDAVVYVGCGER.G
26	1733.8864	1732.8792	1732.8672	6.92	0	27	0.054	1	U	R.EASIYTGITIAEYFR.D
29	1811.8707	1810.8634	1810.8559	4.13	0	68	3.1e-006	1	U	R.LAEMPADSGYPAYLAAR.L + Oxidation (M)

Spot 800

NCBInr protein database

gi|118429132 Mass: 54168 Score: 765 Matches: 12(8) Sequences: 11(8)

vacuolar ATPase subunit B [*Kalidium foliatum*]

Matched peptides shown in **bold red**.

1 MGAQTILDVD DGNLEVGMEY RTVSGVAGPL VILEKVKGPK **FQEIVNIR**LG

51 DGSTRRGQVL EVDGEK**AVVQ VFEGTSGIDN KYTTVQFTGD VLK**TPVSQDM

101 LGRIFNGSGK PIDNGPPILP EAYLDISGSS INPSERTYPE EMIQTGISTI

151 DVMNSIARGQ K**IPLFSAAGL PHNEIAAQIC R**QAGLVKRLE KSENLLEDGG

201 TEDNFAIVFA AMGVNMETAQ FFKRDFEENG SMER**VTLFLN LANDPTIER**I

251 ITPRIALTTA EYLAYECGKH VLVILTDMSS YADALREVSA AREEVPGRR**G**

301 **YPGYMYTDLA TIYER**AGRIE GRSGSITQIP ILTMPNDDIT HPTPDLTGYI

351 TEGQIYIDRQ LHNR**QIYPPI NVLPSLSR**LM KSAIGEGMTR **RDHSDVSNQL**

401 **YANYAIGK**DV QAMKAVVGEE ALSSEDMLYL EFLDKFER**KF VAQGAYDTR**S

451 IFQSLDLAWT LLRIFPRELL HRIPAK**TLDS YYSR**DASN

Query	Observed	Mr(expt)	Mr(calc)	ppm	Miss	Score	Expect	Rank	Unique	Peptide
3	1004.4913	1003.4840	1003.4611	22.9	0	52	0.025	1	U	K.TLDSYYSR.D
4	1018.5915	1017.5842	1017.5607	23.1	0	56	0.0078	1	U	K.FQEIVNIR.L
7	1127.5741	1126.5668	1126.5407	23.1	0	60	0.0026	1	U	K.FVAQGAYDTR.S
17	1255.6686	1254.6613	1254.6357	20.4	1	88	4.5e-06	1	U	R.KFVAQGAYDTR.S
18	1371.7418	1370.7345	1370.7082	19.2	0	96	6.4e-07	1	U	K.YTTVQFTGDVLK.T
21	1563.8271	1562.8198	1562.7941	16.5	0	115	8.7e-09	1	U	K.AVVQVFEGTSGIDNK.Y
23	1596.9363	1595.9290	1595.9035	16.0	0	117	3.1e-09	1		R.QIYPPINVLPSLSR.L
27	1715.9640	1714.9567	1714.9254	18.3	0	92	1.3e-06	1		R.VTLFLNLANDPTIER.I
33	1912.9131	1911.9058	1911.8713	18.1	0	46	0.057	1		R.GYPGYMYTDLATIYER.A
35	1928.9066	1927.8993	1927.8662	17.2	0	(42)	0.13	1		R.GYPGYMYTDLATIYER.A + Oxidation (M)

36 2051.0196 2050.0123 2049.9868 12.5 1 12 1.3e+02 7 U

R.RDHSDVSNQLYANYAIGK.D

38 2178.1897 2177.1824 2177.1415 18.8 0 30 1.5 1

K.IPLFSAAGLPHNEIAAQICR.Q

2. gi|302807614 Mass: 53696 Score: 341 Matches: 6(3) Sequences: 5(3)

hypothetical protein SELMODRAFT_269014 [*Selaginella moellendorffii*]

Query	Observed	Mr(expt)	Mr(calc)	ppm	Miss	Score	Expect	Rank	Unique	Peptide
4	1018.5915	1017.5842	1017.5607	23.1	0	56	0.0078	1	U	K.FQEIVNLR.L
23	1596.9363	1595.9290	1595.9035	16.0	0	117	3.1e-09	1		R.QIYPPINVLPSLSR.L
27	1715.9640	1714.9567	1714.9254	18.3	0	92	1.3e-06	1		R.VTLFLNLANDPTIER.I
33	1912.9131	1911.9058	1911.8713	18.1	0	46	0.057	1		R.GYPGYMYTDLATIYER.A
35	1928.9066	1927.8993	1927.8662	17.2	0	(42)	0.13	1		R.GYPGYMYTDLATIYER.A + Oxidation (M)
38	2178.1897	2177.1824	2177.1415	18.8	0	30	1.5	1		K.IPLFSAAGLPHNEIAAQICR.Q

3. gi|412992647 Mass: 55259 Score: 282 Matches: 5(2) Sequences: 4(2)

predicted protein [*Bathycoccus prasinos*]

Query	Observed	Mr(expt)	Mr(calc)	ppm	Miss	Score	Expect	Rank	Unique	Peptide
23	1596.9363	1595.9290	1595.9035	16.0	0	117	3.1e-09	1		R.QIYPPINVLPSLSR.L
27	1715.9640	1714.9567	1714.9254	18.3	0	88	2.9e-06	2	U	K.TVLFLNLANDPTIER.I
33	1912.9131	1911.9058	1911.8713	18.1	0	46	0.057	1		R.GYPGYMYTDLATIYER.A
35	1928.9066	1927.8993	1927.8662	17.2	0	(42)	0.13	1		R.GYPGYMYTDLATIYER.A + Oxidation (M)
38	2178.1897	2177.1824	2177.1415	18.8	0	30	1.5	1		K.IPLFSAAGLPHNEIAAQICR.Q

Database 1

Match to: Unigene44877_SeCKS transcribed RNA sequence Mass: 27694 Score: 390 Matches: 5(5) Sequences: 5(5)

gi|134034502 vacuolar ATPase subunit B [*Halostachys caspica*]

STMGAQSILDVDDGNLEVGMEYRTVSGVAGPLVILEKVKGPKFQEIVNIRLGDGSTRRGQVLEVDGEKAVVQVFEGTSGIDNK
YTTVQFTGDVLKTPVSQDMLGRIFNGSGKPIDNGPPILPEAYLDISGSSINPSERTYPEEMIQTGISTIDVMNSIARGQKIPLFSAA
GLPHNEIAAQICRQAGLVKRLEKSDNLLEDSGVEDNFAIVFAAMGVNMETAQFFKRDFEENGSMERVTLFLNLANDPTIERIITP

Query	Observed	Mr(expt)	Mr(calc)	ppm	Miss	Score	Expect	Rank	Unique	Peptide
4	1018.5915	1017.5842	1017.5607	23.1	0	56	0.00012	1	U	K.FQEIVNIR.L
18	1371.7418	1370.7345	1370.7082	19.2	0	96	1.1e-008	1	U	K.YTTVQFTGDVLK.T
21	1563.8271	1562.8198	1562.7941	16.5	0	115	1.7e-010	1	U	K.AVVQVFEGTSGIDNK.Y
27	1715.9640	1714.9567	1714.9254	18.3	0	92	1.9e-008	1	U	R.VTLFLNLANDPTIER.I
38	2178.1897	2177.1824	2177.1415	18.8	0	30	0.025	1	U	K.IPLFSAAGLPHNEIAAQICR.Q

2. Unigene46881_Se200S transcribed RNA sequence Mass: 26025 Score: 375 Matches: 7(6) Sequences: 6(5)

gi|118429132 vacuolar ATPase subunit B [*Kalidium foliatum*]

Query	Observed	Mr(expt)	Mr(calc)	ppm	Miss	Score	Expect	Rank	Unique	Peptide
3	1004.4913	1003.4840	1003.4611	22.9	0	52	0.0004	1	U	K.TLDSYYSR.D
7	1127.5741	1126.5668	1126.5407	23.1	0	60	4.4e-005	1	U	K.FVAQGAYDTR.S
17	1255.6686	1254.6613	1254.6357	20.4	1	88	8.5e-008	1	U	R.KFVAQGAYDTR.S
23	1596.9363	1595.9290	1595.9035	16.0	0	117	3.6e-011	1	U	R.QIYPPINVLPSLSR.L

33	1912.9131	1911.9058	1911.8713	18.1	0	46	0.0011	1	U	R.GYPGYMYTDLATIYER.A
35	1928.9066	1927.8993	1927.8662	17.2	0	(42)	0.0021	1	U	R.GYPGYMYTDLATIYER.A + Oxidation (M)
36	2051.0196	2050.0123	2049.9868	12.5	1	12	2.9	1	U	R.RDHSDVSNQLYANYAIGK.D

Database 2

Match to: Unigene50262_SALfmcTARAAPEI-3 Mass: 16158 Score: 214 Matches: 3(3) Sequences: 3(3)

gi|134034502 vacuolar ATPase subunit B [*Halostachys caspica*]

VGMEYRTVSGVAGPLVILEKVKGPKFQEIVNIRLGDGSTRRGQVLEVDGEKAVVQVFEGTSGIDNKYTTVQFTGDVLKTPVSQ

DMLGRIFNGSGKPIDNGPPILPEAYLDISGSSINPSERTYPEEMIQTGISTIDVMNSIARGQKIPPF

Query	Observed	Mr(expt)	Mr(calc)	ppm	Miss	Score	Expect	Rank	Unique	Peptide
4	1018.5915	1017.5842	1017.5607	23.1	0	56	7.3e-005	1	U	K.FQEIVNIR.L
18	1371.7418	1370.7345	1370.7082	19.2	0	96	6e-009	1	U	K.YTTVQFTGDVLK.T
21	1563.8271	1562.8198	1562.7941	16.5	0	115	7.5e-011	1	U	K.AVVQVFEGTSGIDNK.Y

2. Unigene37150_SALfmcTARAAPEI-3 Mass: 10822 Score: 146 Matches: 3(3) Sequences: 3(3)

gi|134034502 vacuolar ATPase subunit B [*Halostachys caspica*]

Query	Observed	Mr(expt)	Mr(calc)	ppm	Miss	Score	Expect	Rank	Unique	Peptide
3	1004.4913	1003.4840	1003.4611	22.9	0	52	0.00023	1	U	K.TLDSYYSR.D
7	1127.5741	1126.5668	1126.5407	23.1	0	60	2.4e-005	1	U	K.FVAQGAYDTR.S
17	1255.6686	1254.6613	1254.6357	20.4	1	88	4.1e-008	1	U	R.KFVAQGAYDTR.S

Spot 1893

NCBInr protein database

Match to: gi|67848430 Mass: 29710 Score: 58 Matches: 1(1) Sequences: 1(1)

mitochrondrial voltage-dependent anion-selective channel [*Phaseolus coccineus*]

Matched peptides shown in bold red.

1 MAKGPGLYTD IGKKARDLLF KDYHSDQKFT VTTYSPTGVA ITSSGTRKGE

51 LFLADVNTQL KNKNITTDIK VDTDSNLFTT ITVNEPAPGL KAIFNFKVPD

101 QRSGKVELQY LHDYAGISTS VGLTANPIVN FSGVVGTNIL ALGADLSFDT

151 KIGELTKSNA GLSFTKDDLI ASLTLNDKGD ALNAAYYHVV NPLTNTAVGA

201 EVTHRFSTNE NTITLGTQHA LDPLTTLKAR VNNFGKSSAL IQHEWRPKSF

251 FTISGEVDTK AIEKSAKVGL SLVLKP

Query	Observed	Mr(expt)	Mr(calc)	ppm	Miss	Score	Expect	Rank	Unique	Peptide
13	2232.1329	2231.1256	2231.1322	-2.93	0	58	0.0027	1	U	K.VDTDSNLFTTITVNEPAPGLK.A

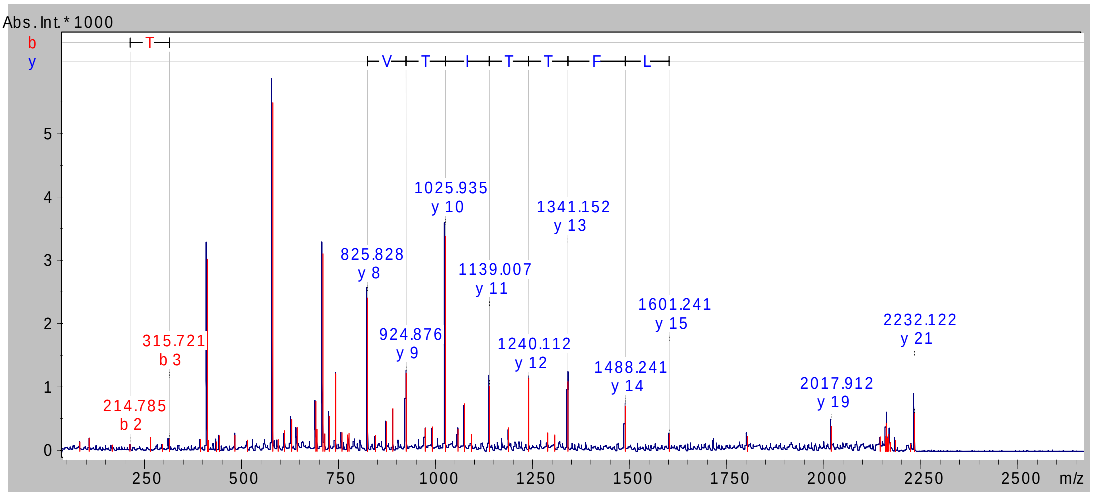

Proteins matching the same set of peptides:

gi|255639656 Mass: 29737 Score: 58 Matches: 1(1) Sequences: 1(1) unknown [*Glycine max*]

gi|255647426 Mass: 29798 Score: 58 Matches: 1(1) Sequences: 1(1) unknown [*Glycine max*]

gi|351725579 Mass: 29752 Score: 58 Matches: 1(1) Sequences: 1(1)

peroxisomal voltage-dependent anion-selective channel protein [*Glycine max*]

gi|356568620 Mass: 29768 Score: 58 Matches: 1(1) Sequences: 1(1)

PREDICTED: outer plastidial membrane protein porin-like isoform 1 [*Glycine max*]

gi|593332355 Mass: 29709 Score: 58 Matches: 1(1) Sequences: 1(1)

hypothetical protein PHAVU_008G043800g [*Phaseolus vulgaris*]

Spot 1305

Database 1

Match to: Unigene19624_Se200S transcribed RNA sequence Mass: 34760 Score: 253 Matches: 4(3) Sequences: 4(3)

gi|255544135 mitochondrial import receptor subunit tom40, putative [*Ricinus communis*]

Matched peptides shown in **bold red**.

FHSRMAATPMTAPAMGAQATELPK**TEEVVDYNNLPCPLPYEEIHR**ESLMSLKPEVFEGMRFDFNKALGPKFFLSHSVAMGPTE

VPSQSPEPIKIPTANYEFGANYLDPKLMLIGRVSHDGRLNARLKCDLTDDLILKGNASLTSEPHMSHAMGTFDYKGKDYRAQL

QLGSGTLVGANYIQSVTPHISLGGEIFWAGQHRK**SGVGYAAR**YSNDKTVATGQVASTGMVALSYVQKVSEKLSFATEFSYNHM

SKDVTGTVGYDAVLRQARVRGK**IDSNGCVTGFLEER**LNMGLTFILSAELDHKKKDYK**FGFGFTVGE**

Query	Observed	Mr(expt)	Mr(calc)	ppm	Miss	Score	Expect	Rank	Unique	Peptide
2	780.4056	779.3983	779.3926	7.30	0	19	0.87	1		K.SGVGYAAR.Y
6	960.4515	959.4442	959.4389	5.55	0	35	0.012	1		K.FGFGFTVGE.-
11	1596.7123	1595.7050	1595.7250	-12.53	0	96	7e-009	1		K.IDSNGCVTGFLEER.L
23	2587.2050	2586.1977	2586.2060	-3.20	0	103	1.4e-009	1	U	K.TEEVVDYNNLPCPLPYEEIHR.E

2. Unigene82324_SeCKS transcribed RNA sequence Mass: 14402 Score: 250 Matches: 4(3) Sequences: 4(3)

gi|629084296 hypothetical protein EUGRSUZ_J00343 [*Eucalyptus grandis*]

Query	Observed	Mr(expt)	Mr(calc)	ppm	Miss	Score	Expect	Rank	Unique	Peptide
2	780.4056	779.3983	779.3926	7.30	0	19	0.87	1		K.SGVGYAAR.Y
6	960.4515	959.4442	959.4389	5.55	0	35	0.012	1		K.FGFGFTVGE.-

9	1379.7125	1378.7052	1378.7093	-2.92	0	100	6e-009	1	U	K.DVTATVGYDAVLR.Q
11	1596.7123	1595.7050	1595.7250	-12.53	0	96	7e-009	1		K.IDSNGCVTGFLEER.L

Database 2

Match to: Unigene55273_SALfmcTARAAPEI-3 Mass: 34105 Score: 353 Matches: 5(4) Sequences: 5(4)

gi|255544135 mitochondrial import receptor subunit tom40, putative [*Ricinus communis*]

Matched peptides shown in **bold red**.

ATPMTAPDMGAQSTELPK**TEEVVDYNNLPCPLPYEEIHR**ESLMSLKPEVFEGMRFDFNKALGPKFFLSHSVAMGPTEVPSQSPE

PIKIPTANYEFGANYLDPKLMLIGRVSHDGRLNARLKCDLTDDLILKGNASLTSEPHMSHAMGTFDYKGKDYRAQLQLGSGTL

VGANYIQSVTPHISLGGEIFWAGQHRK**SGVGYAAR**YSNDKTVATGQVASTGMVALSYVQKVSEKLSFATEFSYNHMSK**DVTAT**

VGYDAVLRQARVRGK**IDSNGCVTGFLEER**LNMGLTFILSAELDHKKKDYKFG**FGFTVGE**

Query	Observed	Mr(expt)	Mr(calc)	ppm	Miss	Score	Expect	Rank	Unique	Peptide
2	780.4056	779.3983	779.3926	7.30	0	19	0.4	1	U	K.SGVGYAAR.Y
6	960.4515	959.4442	959.4389	5.55	0	35	0.011	1	U	K.FGFGFTVGE.-
9	1379.7125	1378.7052	1378.7093	-2.92	0	100	2.5e-009	1	U	K.DVTATVGYDAVLR.Q
11	1596.7123	1595.7050	1595.7250	-12.53	0	96	4.4e-009	1	U	K.IDSNGCVTGFLEER.L
23	2587.2050	2586.1977	2586.2060	-3.20	0	103	6.9e-010	1	U	K.TEEVVDYNNLPCPLPYEEIHR.E

Spot 830

NCBInr protein database

Match to: gi|566168859 Mass: 44022 Score: 244 Matches: 4(3) Sequences: 4(3)

calreticulin family protein [*Populus trichocarpa*]

Matched peptides shown in **bold red**.

1 MENRGRNPSF LSLLLLLSLF AIASAK**VFFE ER**FEDGWEKR WVKSEWKKDE

51 SMAGEWNYTS GKWNGDANDK **GIQTSEDYRF YAISAEFPEF SNK**DNTLVFQ

101 FSVKHEQKLD CGGGYMKLLS GEVDQKKFGG DTPYSIMFGP DICGYSTKKV

151 HAIFNYNDTN HLIKKDVPCE TDQLTHVYTF ILRPDATYSI LIDNQEKQTG

201 SLYTDWDLLP AKKIKDPEAK KPEDWDDKEF IPDPEDKKPE GYDDIPAEIT

251 DPEAKKPEDW DDEEDGEWTA PTIPNPEYKG PWKAKKIKNP NYKGKWKAPM

301 IDNPEFKDDP ELYVYPKLR**Y VGVELWQVK**S GTLFDNVLVC DDPEYAKQLA

351 EETWGKQKDA EKAAFEELEK KREEEETKDD PVESDAEDED EAEADDSDKD

401 DADKSDDKDD DQHDEL

Query	Observed	Mr(expt)	Mr(calc)	ppm	Miss	Score	Expect	Rank	Unique	Peptide
4	826.4305	825.4232	825.4021	25.6	0	18	36	1		K.VFFEER.F
8	1052.4665	1051.4593	1051.4359	22.2	0	47	0.045	1		R.FEDGWENR.W
16	1649.8097	1648.8024	1648.7773	15.2	0	71	0.00022	1		R.FYAISAEFPEFSNK.D
22	1968.8991	1967.8918	1967.8609	15.7	1	108	4.3e-08	1		K.WNGDANDKGIQTSEDYR.F

2. gi|11131631 Mass: 48334 Score: 221 Matches: 4(2) Sequences: 4(2)

RecName: Full=Calreticulin; Flags: Precursor

Query	Observed	Mr(expt)	Mr(calc)	ppm	Miss	Score	Expect	Rank	Unique	Peptide
4	826.4305	825.4232	825.4021	25.6	0	18	36	1		K.VFFEER.F
13	1220.6889	1219.6816	1219.6601	17.6	0	25	9.3	2	U	R.YVGVELWQVK.S
16	1649.8097	1648.8024	1648.7773	15.2	0	71	0.00022	1		R.FYAISAEFPEFSNK.D

22	1968.8991	1967.8918	1967.8609	15.7	1	108	4.3e-08	1		K.WNGDANDKGIQTSEDYR.F

3. gi|527204054 Mass: 38714 Score: 162 Matches: 3(2) Sequences: 3(2)

calreticulin, partial [*Genlisea aurea*]

Query	Observed	Mr(expt)	Mr(calc)	ppm	Miss	Score	Expect	Rank	Unique	Peptide
4	826.4305	825.4232	825.4021	25.6	0	18	36	1		K.VFFEER.F
16	1649.8097	1648.8024	1648.7773	15.2	0	71	0.00022	1		R.FYAISAEFPEFSNK.D
22	1968.8991	1967.8918	1967.8497	21.4	1	74	0.00011	3	U	K.WSGDPDDKGIQTSEDYR.F

Database 1

Match to: Unigene15150_SeCKS transcribed RNA Mass: 49380 Score: 475 Matches: 8(5) Sequences: 8(5)

gi|224122952 calreticulin family protein [*Populus trichocarpa*]

Matched peptides shown in bold red.

FLNLSLSSNLAMEFRKRNPSSFSLLILLSLFAIASAKVFFEERFEDGWENRWVKSDWKKDENMAGEWNYTSGKWNGDANDK

GIQTSEDYRFYAISAEFPEFSNKEKTLVFQFSVKHEQKLDCGGGYMKLLSGDVDQKKFGGDTPYSIMFGPDICGYSTKKVHAIL

TYNDTNHLIKKDVPCETDQLTHVYTFILRPDATYSILIDNEEKQTGSLYNDWDLLPPKKIKDPEAKKPEDWDDKEYISDPEDKK

PEGYDDIPKEIPDADAKKPEDWDDEEDGEWTAPTIPNPEYKGPWKPKKIKNPNYKGKWKAPMIDNPDFKDDPELYVFPNLKY

VGVELWQVKSGTLFDNVLVCDDPDYAKKLAEETWGKHKDAEKSAFEEAEKKREEEETKDDPVESDAEDEDDADDSEGDAD

DSDKSDAKDDDTHDEL

Query	Observed	Mr(expt)	Mr(calc)	ppm	Miss	Score	Expect	Rank	Unique	Peptide
4	826.4305	825.4232	825.4021	25.6	0	18	0.66	1	U	K.VFFEER.F
8	1052.4665	1051.4593	1051.4359	22.2	0	47	0.00054	1	U	R.FEDGWENR.W
10	1068.5287	1067.5214	1067.4883	31.0	0	10	5.6	1	U	K.GIQTSEDYR.F
13	1220.6889	1219.6816	1219.6601	17.6	0	25	0.14	1	U	K.YVGVELWQVK.S
16	1649.8097	1648.8024	1648.7773	15.2	0	71	4.4e-006	1	U	R.FYAISAEFPEFSNK.E
19	1906.9450	1905.9377	1905.9148	12.0	1	88	7.3e-008	1	U	R.FYAISAEFPEFSNKEK.T
22	1968.8991	1967.8918	1967.8609	15.7	1	108	5.6e-010	1	U	K.WNGDANDKGIQTSEDYR.F
24	2028.9472	2027.9399	2027.9146	12.4	0	109	4.2e-010	1	U	K.SGTLFDNVLVCDDPDYAK.K

Database 2

Match to: Unigene38027_SALfmcTARAAPEI-3 Mass: 13310 Score: 167 Matches: 3(2) Sequences: 3(2)

gi|15217459 calreticulin 1b [*Arabidopsis thaliana*]

NGDANDKGIQTSEDYRFYAISAEFPEFSNKEKTLVFQFSVKHEQKLDCGGGYMKLLSGDVDQKKFGGDTPYRUTIFLAFCEFW

ILLLLLFHNCLLVLIYNGWRCSIMFGPDIC

Query	Observed	Mr(expt)	Mr(calc)	ppm	Miss	Score	Expect	Rank	Unique	Peptide
10	1068.5287	1067.5214	1067.4883	31.0	0	10	2.8	1	U	K.GIQTSEDYR.F
16	1649.8097	1648.8024	1648.7773	15.2	0	71	1.9e-006	1	U	R.FYAISAEFPEFSNK.E
19	1906.9450	1905.9377	1905.9148	12.0	1	88	3.3e-008	1	U	R.FYAISAEFPEFSNKEK.T

Spot 1358

Database 2

Match to: Unigene24274_SALfmcTARAAPEI-3 Mass: 8296 Score: 30 Matches: 1(1) Sequences: 1(1)

gi|460406796 PREDICTED: annexin-like protein RJ4-like [*Solanum lycopersicum*]

Matched peptides shown in bold red.

GTDEKGIIQIIGHRTAPQRKLIKEAYEQLYGENLIKLFEKELSGNFEKAVYRWMLEPCDREAVLAHNALKE

Query	Observed	Mr(expt)	Mr(calc)	ppm	Miss	Score	Expect	Rank	Unique	Peptide
2	1006.6036	1005.5963	1005.6083	-11.97	0	30	0.026	1	U	K.GIIQIIGHR.T

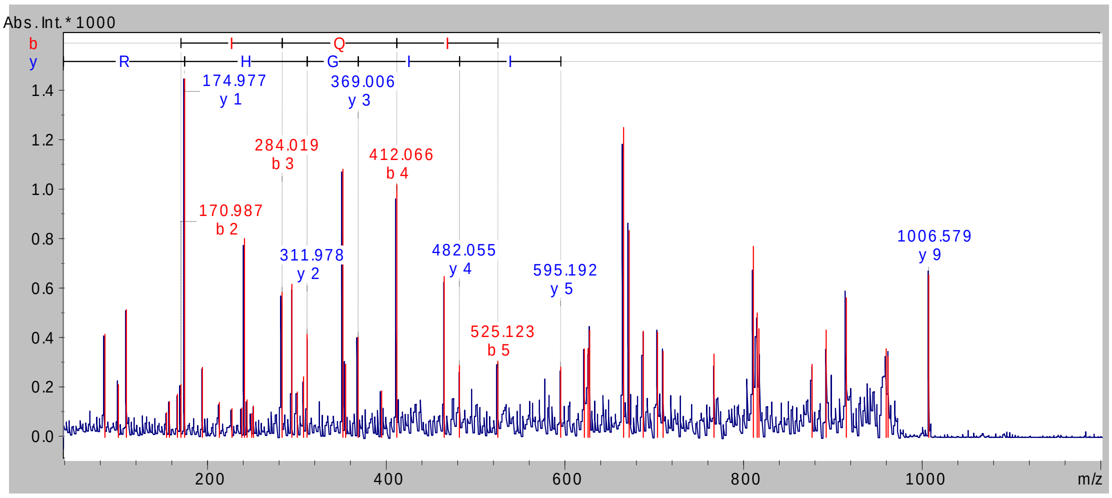

Spot 1401

Database 1

Match to: Unigene45291_SeCKS transcribed RNA sequence Mass: 15349 Score: 156 Matches: 3(3) Sequences: 3(3)

gi|460406796 PREDICTED: annexin-like protein RJ4-like [*Solanum lycopersicum*]

Matched peptides shown in bold red.

VIKVEFRYREREKKTQISVCKITMASLKAPEEFDPIADSEVLHEACHGWTTDEKAIIQIIGHRTAAQRKLLKEVYEQQYSEDLSK
LFEDKLHGHFEKAVYRWMLEPCDREAVLAHNALKEKEYQVIVEI

Query	Observed	Mr (expt)	Mr(calc)	ppm	Miss	Score	Expect	Rank	Unique	Peptide
3	1020.6337	1019.6264	1019.6240	2.42	0	36	0.0046	1	U	K.AIIQIIGHR.T
12	1499.7762	1498.7689	1498.7569	8.04	1	35	0.016	1	U	K.LFEDKLHGHFEK.A
16	1617.7416	1616.7343	1616.7206	8.52	0	85	1e-007	1	U	K.EVYEQQYSEDLSK.L

2. Unigene46801_Se200S transcribed RNA sequence Mass: 23202 Score: 135 Matches: 5(2) Sequences: 4(2)

gi|523712978 cotton annexin 4 [*Gossypium hirsutum*]

Query	Observed	Mr(expt)	Mr(calc)	ppm	Miss	Score	Expect	Rank	Unique	Peptide
7	1108.6220	1107.6147	1107.6077	6.34	0	19	0.5	1	U	K.AQLIPTFYR.Y
14	1529.7517	1528.7445	1528.7232	13.9	0	41	0.0039	1	U	K.DLGEIMELFYQR.H + Oxidation (M)
17	1841.9154	1840.9081	1840.9029	2.83	1	(22)	0.29	1	U	R.AEKDLGEIMELFYQR.H
18	1857.9220	1856.9147	1856.8978	9.09	1	51	0.00038	1	U	R.AEKDLGEIMELFYQR.H + Oxidation (M)
26	2221.9613	2220.9541	2220.9204	15.1	0	23	0.079	1	U	R.YNHSLEEDVACYFTGNMR.K + Oxidation (M)

Spot 1402

Database 1

Match to: Unigene45291_SeCKS transcribed RNA sequence Mass: 15349 Score: 63 Matches: 1(1) Sequences: 1(1)

gi|460406796 PREDICTED: annexin-like protein RJ4-like [*Solanum lycopersicum*]

Matched peptides shown in bold red.

VIKVEFRYREREKKTQISVCKITMASLKAPEEFDPIADSEVLHEACHGWTTDEKAIIQIIGHRTAAQRKLLKEVYEQQYSEDLSK
LFEDKLHGHFEKAVYRWMLEPCDREAVLAHNALKEKEYQVIVEI

Query	Observed	Mr(expt)	Mr(calc)	ppm	Miss	Score	Expect	Rank	Unique	Peptide

8	1617.7139	1616.7066	1616.7206	-8.63	0	63	1.4e-005	1	U	K.EVYEQQYSEDLSK.L

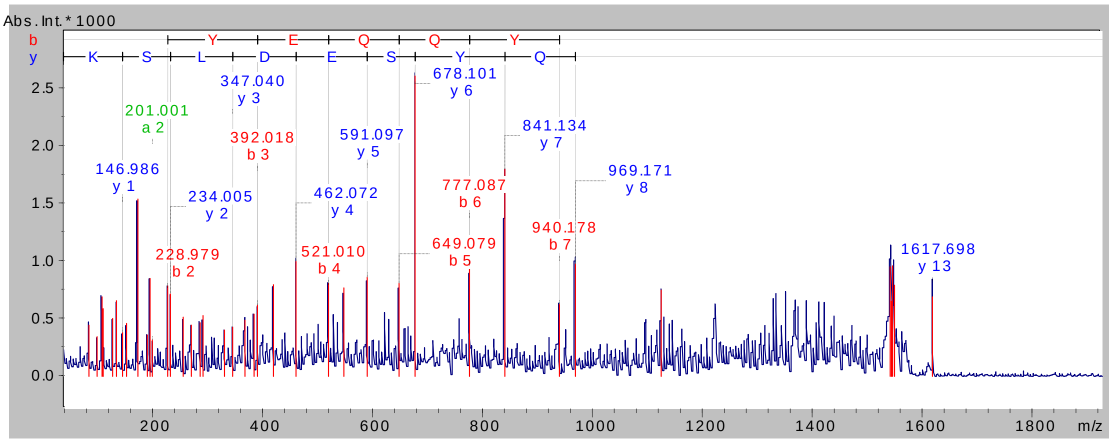

Proteins matching the same set of peptides:

Unigene46048_Se200S transcribed RNA sequence Mass: 15236 Score: 63 Matches: 1(1) Sequences: 1(1)

gi|658042335 annexin-like protein RJ4 [*Malus domestica*]

Spot 1480

Database 1

Match to: Unigene38480_SeCKS transcribed RNA sequence Mass: 30539 Score: 167 Matches: 4(3) Sequences: 4(3)

gi|359807257 Annexin D4-like [*Glycine max*]

Matched peptides shown in **bold red**.

VNLCSFLVQFIQLIEAALRSIMADVCYENEALTLTQAFSGLGVDEKALISIAGKLKSDSIR**KATPFLFSADER**HFEK**SNEQQVELIKHEALR**FKNAVVLWTMHPWERDARFFKEALTKGSKGYSLIIQIACARSSEELLGARKAYHSIFHSSVEEDVASYAHGHERKFLVALVSAYRYEGGRVRDETAKAEAEVLHDAIKK**TFRPIEDDEVVR**IFCTRSKLHLKAISRYYKELYGKDLDEELQGDLSLKEALQCLVVPEVHFSEVL

Query	Observed	Mr(expt)	Mr(calc)	ppm	Miss	Score	Expect	Rank	Unique	Peptide
5	1253.6396	1252.6323	1252.6088	18.8	0	29	0.07	1	U	K.ATPFLFSADER.H
6	1381.7335	1380.7263	1380.7038	16.3	1	36	0.013	1	U	R.KATPFLFSADER.H
8	1475.7732	1474.7659	1474.7416	16.5	0	45	0.0019	1	U	K.TFRPIEDDEVVR.I
11	1793.9654	1792.9581	1792.9431	8.37	1	58	6.8e-005	1	U	K.SNEQQVELIKHEALR.F

Proteins matching the same set of peptides:

Unigene38488_Se200S transcribed RNA sequence Mass: 30327 Score: 167 Matches: 4(3) Sequences: 4(3)

gi|359807257 annexin D4-like [*Glycine max*]

Database 2

Match to: Unigene55854_SALfmcTARAAPEI-3 Mass: 35257 Score: 167 Matches: 4(4) Sequences: 4(4)

gi|359807257 annexin D4-like [*Glycine max*]

Matched peptides shown in **bold red**.

VCYENEALTLTQAFSGLGVDEKALISIAGKLKSDSIR**KATPFLFSADER**HFEK**SNEQQVELIKHEALR**FKNAVVLWTMHPWERDARFFKEALTKGSKGYSLIIQIACARSSEELLGARKAYHSIFHSSVEEDVASYAHGHERKFLVALVSAYRYEGGRVRDETAKAEAEVLHDAIKK**TFRPIEDDEVVR**IFCTRSKLHLKAISRYYKELYGKDLDEELQGDLSLKEALQCLVVPEVHFSEVLDASMRKGADDFTQEALTQVIVSQADVNMKEIQAVYYEKFGVQLSQKIEETCLGNFKQFLLTLVARG

Query	Observed	Mr(expt)	Mr(calc)	ppm	Miss	Score	Expect	Rank	Unique	Peptide
5	1253.6396	1252.6323	1252.6088	18.8	0	29	0.033	1	U	K.ATPFLFSADER.H
6	1381.7335	1380.7263	1380.7038	16.3	1	36	0.0061	1	U	R.KATPFLFSADER.H
8	1475.7732	1474.7659	1474.7416	16.5	0	45	0.00078	1	U	K.TFRPIEDDEVVR.I

11	1793.9654	1792.9581	1792.9431	8.37	1	58	3.7e-005	1	U	K.SNEQQVELIKHEALR.F

Spot 2278

NCBInr protein database

Match to: gi|470125402 Mass: 16620 Score: 151 Matches: 2(1) Sequences: 2(1)

PREDICTED: probable calcium-binding protein CML13-like [*Fragaria vesca subsp. vesca*]

Matched peptides shown in **bold red**.

1 MGKDLSDDQV SSMK**EAFTLF DTDNDGK**IAP SELGILMRSL GGNPTQAQLK

51 SIVAEEKLTA PFDFKRFLEL MSKHMKPEPF DRQLRDAFK**V LDKDATGFVS**

101 **VSELR**HILTS IGEKLEPSEF DEWIREVDVG KDGKIKYDDF IARMVAK

Query	Observed	Mr(expt)	Mr(calc)	ppm	Miss	Score	Expect	Rank	Unique	Peptide
19	1472.6833	1471.6760	1471.6467	19.9	0	109	3.7e-08	1		K.EAFTLFDTDNDGK.I
23	1735.9515	1734.9442	1734.9152	16.7	1	42	0.13	1	U	K.VLDKDATGFVSVSELR.H

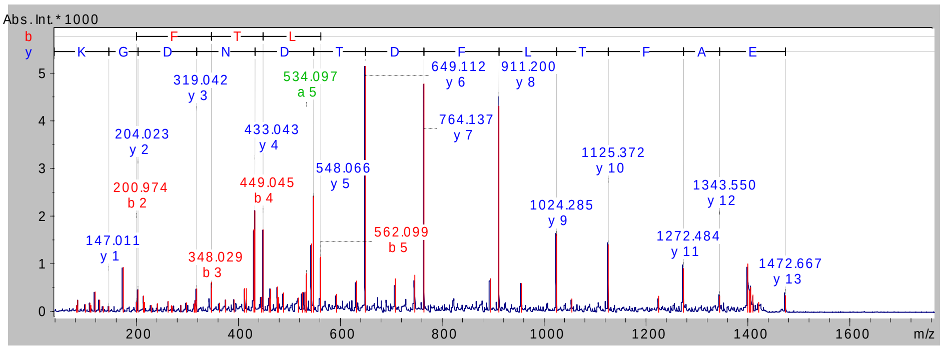

2. gi|595851241 Mass: 20091 Score: 146 Matches: 2(1) Sequences: 2(1)

hypothetical protein PRUPE_ppa019592mg, partial [Prunus persica]

Query	Observed	Mr(expt)	Mr(calc)	ppm	Miss	Score	Expect	Rank	Unique	Peptide
7	1154.6352	1153.6279	1153.6131	12.8	1	38	0.62	1	U	K.IKYEDFIAR.M
19	1472.6833	1471.6760	1471.6467	19.9	0	109	3.7e-08	1		K.EAFTLFDTDNDGK.I

3. gi|526117828 Mass: 16588 Score: 102 Matches: 2(1) Sequences: 2(1)

serine/threonine kinase-like [Vitis vinifera]

Query	Observed	Mr(expt)	Mr(calc)	ppm	Miss	Score	Expect	Rank	Unique	Peptide
15	1404.6945	1403.6872	1403.6721	10.7	0	65	0.001	1	U	K.LEPAEFDEWIR.E
23	1735.9515	1734.9442	1734.9152	16.7	1	37	0.39	2	U	K.VLDKESTGFVSVADLR.H

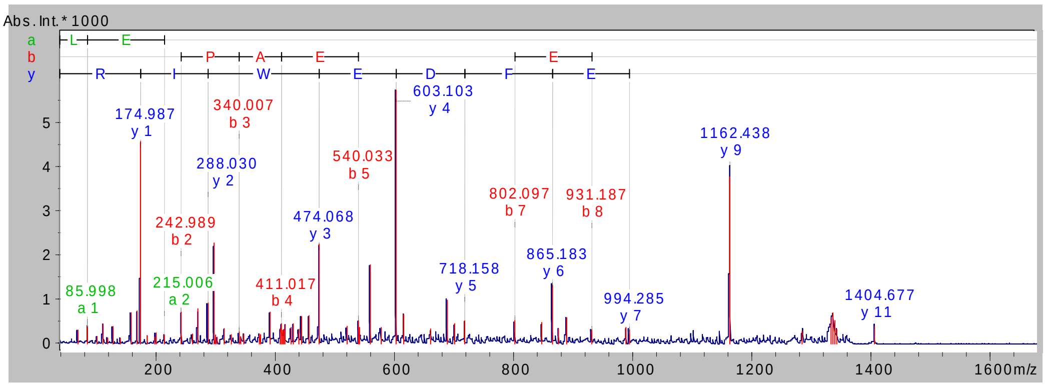

Database 1

Match to: Unigene18664_SeCKS transcribed RNA sequence Mass: 25466 Score: 526 Matches: 8(6) Sequences: 8(6)

gi|502160156 PREDICTED: probable calcium-binding protein CML13-like [*Cicer arietinum*]

Matched peptides shown in **bold red**.

KKREKKKNILPFKPKKTQNFKASGKREKSSLINKTAPSSPPLPTFKTTTITSTTEEEQQQKSTHLQNNTIHQINPKKMGKDLTDD QVSSMK**EAFTLFDTDNDGKIAPSELGILMR**SLGGNPTQAQLK**EIISQENLTTAFDFNR**FLDLMSK**YLKPEPFDR**QLRDAFKVLD K**EGTGYVAVTDLR**HILTSIGEK**LEPAEFDEWIR**EVEVGSDGR**IKYEDFIAR**MVAK

Query	Observed	Mr(expt)	Mr(calc)	ppm	Miss	Score	Expect	Rank	Unique	Peptide
7	1154.6352	1153.6279	1153.6131	12.8	1	38	0.0074	1	U	R.IKYEDFIAR.M
9	1164.6179	1163.6106	1163.5975	11.3	0	39	0.0075	1	U	K.YLKPEPFDR.Q
13	1215.6984	1214.6911	1214.6693	18.0	0	22	0.29	1	U	K.IAPSELGILMR.S + Oxidation (M)
14	1280.6700	1279.6627	1279.6408	17.1	0	28	0.085	1	U	K.EGTGYVAVTDLR.H
15	1404.6945	1403.6872	1403.6721	10.7	0	65	1.9e-005	1	U	K.LEPAEFDEWIR.E
19	1472.6833	1471.6760	1471.6467	19.9	0	109	4.9e-010	1	U	K.EAFTLFDTDNDGK.I
23	1735.9515	1734.9442	1734.9152	16.7	1	99	4.6e-009	1	U	K.VLDKEGTGYVAVTDLR.H
24	1897.9486	1896.9413	1896.9217	10.3	0	126	1e-011	1	U	K.EIISQENLTTAFDFNR.F

Database 2

Match to: Unigene39866_SALfmcTARAAPEI-3 Mass: 12190 Score: 264 Matches: 6(6) Sequences: 6(6)

gi|502160156 PREDICTED: probable calcium-binding protein CML13-like [*Cicer arietinum*]

Matched peptides shown in **bold red**.

NPTQAQLK**EIISQENLTTAFDFNR**FLDLMSK**YLKPEPFDR**QLRDAFK**VLDKEGTGYVAVTDLR**HILTSIGEK**LEPAEFDEWIR**EV EVGSDGR**IKYEDFIAR**MVAK

Query	Observed	Mr(expt)	Mr(calc)	ppm	Miss	Score	Expect	Rank	Unique	Peptide
7	1154.6352	1153.6279	1153.6131	12.8	1	38	0.0044	1	U	R.IKYEDFIAR.M
9	1164.6179	1163.6106	1163.5975	11.3	0	39	0.0036	1	U	K.YLKPEPFDR.Q
14	1280.6700	1279.6627	1279.6408	17.1	0	28	0.038	1	U	K.EGTGYVAVTDLR.H
15	1404.6945	1403.6872	1403.6721	10.7	0	65	8.8e-006	1	U	K.LEPAEFDEWIR.E
23	1735.9515	1734.9442	1734.9152	16.7	1	99	2.6e-009	1	U	K.VLDKEGTGYVAVTDLR.H
24	1897.9486	1896.9413	1896.9217	10.3	0	126	4.8e-012	1	U	K.EIISQENLTTAFDFNR.F

Spot 1656

Database 1

Match to: Unigene32065_SeCKS transcribed RNA sequence Mass: 19732 Score: 41 Matches: 2(1) Sequences: 2(1)

gi|481048335 PIB41 14-3-3 protein [*Petunia x hybrida*]

Matched peptides shown in **bold red**.

VIKEYRAKIETELTKICDGILKLLDTHLIPSATVAESKVFYLKMNGDYHRYLAEFKTAAGRKESAEHTLTAYK**SAQDIALAELP PTHPIR**LGLALNFSVFYYEILNSPDRACALAKQAFDEAISELDTLGEESYK**DSTLIMQLLR**DNLTLWTSDNADEVGDEIKEASK DDAAPGQQ

Query	Observed	Mr(expt)	Mr(calc)	ppm	Miss	Score	Expect	Rank	Unique	Peptide
18	1189.6895	1188.6823	1188.6536	24.1	0	7	8.4	6		K.DSTLIMQLLR.D
37	1829.0318	1828.0245	1827.9843	22.0	0	35	0.0086	1	U	K.SAQDIALAELPPTHPIR.L

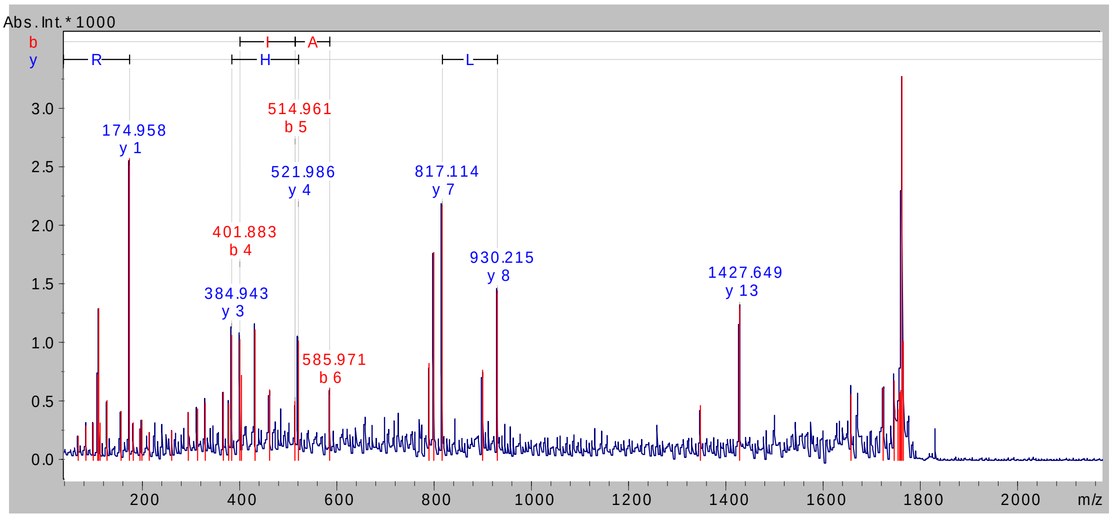

Proteins matching the same set of peptides:

Unigene47158_Se200S transcribed RNA sequence Mass: 22526 Score: 41 Matches: 2(1) Sequences: 2(1)

gi|481048335 PIB41 14-3-3 protein [*Petunia x hybrida*]

2. Unigene12859_Se200S transcribed RNA sequence Mass: 15809 Score: 38 Matches: 2(1) Sequences: 2(1)

gi|595793675 hypothetical protein PRUPE_ppa010141mg [Prunus persica]

Query	Observed	Mr(expt)	Mr(calc)	ppm	Miss	Score	Expect	Rank	Unique	Peptide
18	1189.6895	1188.6823	1188.6536	24.1	0	7	8.4	6		K.DSTLIMQLLR.D
36	1804.0014	1802.9941	1802.9275	37.0	0	31	0.027	1	U	K.SAQDIANAELAPTHPIR.L

Proteins matching the same set of peptides:

Unigene14137_SeCKS transcribed RNA sequence Mass: 15767 Score: 38 Matches: 2(1) Sequences: 2(1)

gi|255545792 14-3-3 protein, putative [Ricinus communis]

Spot 1559

NCBInr protein database

Match to: gi|302122830 Mass: 29401 Score: 150 Matches: 3(2) Sequences: 3(2)

14-3-3h protein [*Gossypium hirsutum*]

Matched peptides shown in **bold red**.

1 MDFSKERERF VYIAKLAEQA ERFDDMVTSM KKLAELDVEL TVEERNLLSV

51 GYKNVIGARR ASWRILSSIE QKEESKGNEQ NVKKIKTSRQ KVESELSNIC

101 KDIMGVINEH LIPSCSGGES TGFYYKMKGD YYRYLAEFKM GDERKEAADQ

151 SMKAYQSATT TAEAELPPTH PIRLGLALNF SVFYYEIMNS PERACHLAKQ

201 AFDEAISELD TLSEESYKDS TLIMQLLRDN LTLWTSDIPE DGEEAAKLGA

251 AKVGEGDE

Query	Observed	Mr(expt)	Mr(calc)	ppm	Miss	Score	Expect	Rank	Unique	Peptide
2	770.4148	769.4075	769.4010	8.43	0	38	0.41	1		R.YLAEFK.M
10	1515.8172	1514.8099	1514.7828	17.9	0	49	0.032	1	U	K.LAELDVELTVEER.N
11	1643.9268	1642.9195	1642.8777	25.4	1	63	0.0011	1	U	K.KLAELDVELTVEER.N

Database 1

Match to: Unigene44887_SeCKS transcribed RNA sequence Mass: 9673 Score: 138 Matches: 3(2) Sequences: 3(2)

gi|587906570 14-3-3-like protein B [*Morus notabilis*]

Matched peptides shown in **bold red**.

PFPMASTKERENFVYTAKLAEQAERYDEMVEAMRKLAELDVELTVEERNLLSVGYKNVVGARRASWRILSSIEQKEEGKGNE

QN

Query	Observed	Mr(expt)	Mr(calc)	ppm	Miss	Score	Expect	Rank	Unique	Peptide
6	1256.6340	1255.6268	1255.6197	5.65	1	26	0.16	1	U	K.ERENFVYTAK.L
10	1515.8172	1514.8099	1514.7828	17.9	0	49	0.00058	1	U	K.LAELDVELTVEER.N
11	1643.9268	1642.9195	1642.8777	25.4	1	63	1.5e-005	1	U	R.KLAELDVELTVEER.N

Proteins matching the same set of peptides:

Unigene46689_Se200S transcribed RNA sequence Mass: 8946 Score: 138 Matches: 3(2) Sequences: 3(2)

gi|645237605 PREDICTED: 14-3-3-like protein C [*Prunus mume*]

Database 2

Match to: Unigene20754_SALfmcTARAAPEI-3 Mass: 7647 Score: 38 Matches: 1(1) Sequences: 1(1)

gi|90856221 14-3-3 protein [*Camellia sinensis*]

Matched peptides shown in bold red.

ICDGILKLLDTKLVPAASVGDPKVFYLKMKGDYHRYLAEFKTGSERKESAESTLNAYKSAQDIANAELA

Query	Observed	Mr(expt)	Mr(calc)	ppm	Miss	Score	Expect	Rank	Unique	Peptide
2	770.4148	769.4075	769.4010	8.43	0	38	0.0036	1	U	R.YLAEFK.T

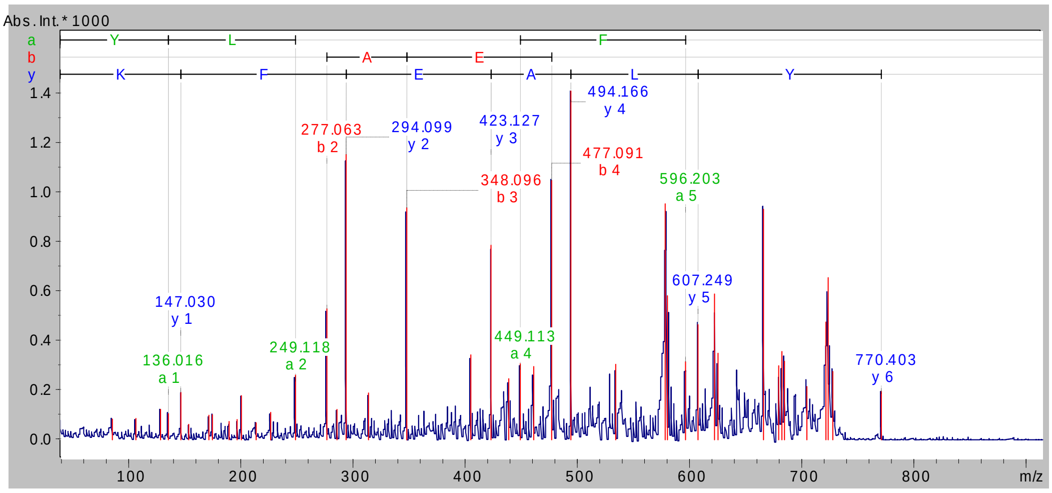

Proteins matching the same set of peptides:

Unigene30258_SALfmcTARAAPEI-3 Mass: 7930 Score: 38 Matches: 1(1) Sequences: 1(1)

gi|334192417 14-3-3 protein [*Pyrus pyrifolia*]

Unigene50562_SALfmcTARAAPEI-3 Mass: 25802 Score: 38 Matches: 1(1) Sequences: 1(1)

gi|672241334 14-3-3d [*Morus alba var. atropurpurea*]

Unigene51720_SALfmcTARAAPEI-3 Mass: 25858 Score: 38 Matches: 1(1) Sequences: 1(1)

gi|351724655 14-3-3-like protein D [*Glycine max*]

Spot 1196

Database 2

Match to: Unigene54833_SALfmcTARAAPEI-3 Mass: 30334 Score: 132 Matches: 2(2) Sequences: 2(2)

gi|297799222

band 7 family protein [*Arabidopsis lyrata subsp. lyrata*] >gi|297313331|gb|EFH43754.1| band 7 family protein [*Arabidopsis lyrata subsp. lyrata*]

RIAYIHSLKEQTIEIPEQPAVTTDNVSIRVNGVLFVKVVDAEKASYGNDNVMFAVTQLAQTVMRSAIGKMPLDKTFMERDTLNA
YIVDSINDVASQWGLVCRRYEIRDIIPPPGIRESMEMQAEAERKKRAEIIEAEGKKQAVILTSDAARMDLINRATGEAEAIRIVSE
ALHRDGGQAAAALKLAHDYVQAFNNMAKESTTMLLPSSVGDASSMVASALAIYKNTILNTPGKKTLHKPSDPTSSARQLVEE
DDEEDSALEDDDKPVFSLQSSKNT

Query	Observed	Mr(expt)	Mr(calc)	ppm	Miss	Score	Expect	Rank	Unique	Peptide
5	977.5863	976.5790	976.5706	8.69	0	27	0.047	1	U	R.DIIPPPGIR.E
28	2240.1549	2239.1476	2239.1332	6.45	0	105	5.7e-010	1	U	K.EQTIEIPEQPAVTTDNVSIR.V

Spot 1683

NCBInr protein database

Match to: gi|351722551 Mass: 27432 Score: 195 Matches: 4(2) Sequences: 4(2)

uncharacterized protein LOC100306559 [*Glycine max*]

Matched peptides shown in bold red.

1 MGQALGCYQV DQSNVAIKEH FGKFDDVLEP GCHCLPWCLG YQIAGSLSLR

51 VQQLDVRCET KTKDNVFVTV VASVQYRAVS EKASDAFYRL TNTREQIQSY

101 VFDVIRASVP KLELDSVFEQ KNDIAKAVEE ELEKAMSTYG FEIVQTLIVD

151 IEPDVNVKRA MNEINAAARL RLAANEKAEA EKILQIKKAE GEAESKYLSG

201 LGIARQRQAI VDGLRDSALA FSENVPGTTA KDVMDMVLVT HTLTP

Query	Observed	Mr(expt)	Mr(calc)	ppm	Miss	Score	Expect	Rank	Unique	Peptide
9	1076.5481	1075.5408	1075.5080	30.5	0	21	29	1		R.AMNEINAAAR.L

+ Oxidation (M)

Query	Observed	Mr(expt)	Mr(calc)	ppm	Miss	Score	Expect	Rank	Unique	Peptide
12	1207.6532	1206.6459	1206.6132	27.1	0	51	0.025	1	U	K.LELDSVFEQK.N
25	1689.9465	1688.9393	1688.8985	24.1	1	40	0.21	1	U	R.ASVPKLELDSVFEQK.N
26	1826.0271	1825.0199	1824.9734	25.4	1	83	9.4e-06	1		K.TKDNVFVTVVASVQYR.A

Proteins matching the same set of peptides:

gi|388514777 Mass: 31693 Score: 195 Matches: 4(2) Sequences: 4(2)

unknown [*Lotus japonicus*]

gi|558695702 Mass: 27523 Score: 195 Matches: 4(2) Sequences: 4(2)

hypersensitive-induced response protein [*Phaseolus vulgaris*]

gi|571495746 Mass: 31816 Score: 195 Matches: 4(2) Sequences: 4(2)

PREDICTED: uncharacterized protein LOC100306559 isoform X1 [*Glycine max*]

gi|593701555 Mass: 31842 Score: 195 Matches: 4(2) Sequences: 4(2)

hypothetical protein PHAVU_004G026100g [*Phaseolus vulgaris*]

2. gi|527198851 Mass: 31717 Score: 182 Matches: 3(2) Sequences: 3(2)

hypothetical protein M569_07929, partial [*Genlisea aurea*]

Query	Observed	Mr(expt)	Mr(calc)	ppm	Miss	Score	Expect	Rank	Unique	Peptide
9	1076.5481	1075.5408	1075.5080	30.5	0	21	29	1		R.AMNEINAAAR.L + Oxidation (M)
19	1394.7895	1393.7822	1393.7466	25.5	0	101	1.7e-07	2	U	K.SSAVFIPHGPGAVR.D
22	1480.8178	1479.8105	1479.7722	25.9	0	59	0.0031	1		R.EQIQAYVFDVIR.A

3. gi|94983903 Mass: 15264 Score: 163 Matches: 3(2) Sequences: 3(2)

salinity-induced protein [*Alternanthera philoxeroides*]

Query	Observed	Mr(expt)	Mr(calc)	ppm	Miss	Score	Expect	Rank	Unique	Peptide
9	1076.5481	1075.5408	1075.5080	30.5	0	21	29	1		R.AMNEINAAAR.M + Oxidation (M)
22	1480.8178	1479.8105	1479.7722	25.9	0	59	0.0031	1		R.EQIQAYVFDVIR.A
26	1826.0271	1825.0199	1824.9734	25.4	1	83	9.4e-06	1		K.TKDNVFVTVVASVQYR.A

Database 1

Match to: Unigene14982_SeCKS transcribed RNA sequence Mass: 32486 Score: 546 Matches: 13(7) Sequences: 12(7)

gi| 525507222 hypersensitive-induced response protein 1-like [*Cucumis sativus*]

Matched peptides shown in bold red.

DKRTRIMGQALGCVQVGQSTVAISERFGKFNDVLNPGCHCVPWCLGSQVAGYLTLRVQQLDVRCETKTKDNVFVTVVASVQY
RAVSDNATDAFYKLSNTREQIQAYVFDVIRASVPKLELDSVFEQKNDIAKSVEQELEKAMSAYGYEIVQTLIVDIEPDVNVKRA
MNEINAAARMRVAANEKAEAEKILQIKKAEGEAESKYLGGMGIARQRQAIVDGLRDSVLQFSENVPGTSAKDVMDMILITQY
FDTMKEIGASSKASSVFIPHGPGAVRDIGNQIRDGLLQAQTMER

Query	Observed	Mr(expt)	Mr(calc)	ppm	Miss	Score	Expect	Rank	Unique	Peptide
4	815.4616	814.4543	814.4297	30.2	0	22	0.41	2	U	R.DIGNQIR.D
5	857.5049	856.4977	856.4767	24.5	0	19	0.79	1	U	R.VQQLDVR.C
6	871.5244	870.5171	870.4923	28.5	0	16	1.3	1	U	R.QAIVDGLR.D
7	937.5176	936.5104	936.4851	27.0	0	(19)	0.78	1	U	K.YLGGMGIAR.Q
8	953.5151	952.5079	952.4800	29.2	0	20	0.58	1	U	K.YLGGMGIAR.Q + Oxidation (M)
9	1076.5481	1075.5408	1075.5080	30.5	0	21	0.58	1	U	R.AMNEINAAAR.M + Oxidation (M)
12	1207.6532	1206.6459	1206.6132	27.1	0	51	0.00048	1	U	K.LELDSVFEQK.N
17	1301.6411	1300.6338	1300.5935	31.0	0	31	0.039	1	U	R.AVSDNATDAFYK.L
19	1394.7895	1393.7822	1393.7466	25.5	0	104	1.3e-009	1	U	K.ASSVFIPHGPGAVR.D
22	1480.8178	1479.8105	1479.7722	25.9	0	59	4.5e-005	1	U	R.EQIQAYVFDVIR.A
24	1678.8706	1677.8633	1677.8210	25.2	0	79	5.7e-007	1	U	R.DSVLQFSENVPGTSAK.D
25	1689.9465	1688.9393	1688.8985	24.1	1	40	0.0032	1	U	R.ASVPKLELDSVFEQK.N
26	1826.0271	1825.0199	1824.9734	25.4	1	83	1.3e-007	1	U	K.TKDNVFVTVVASVQYR.A

Proteins matching the same set of peptides:

Unigene14983_SeCKS transcribed RNA sequence Mass: 32486 Score: 546 Matches: 13(7) Sequences: 12(7)

gi|659113223 hypersensitive-induced response protein 2 [*Cucumis melo*]

Unigene16990_Se200S transcribed RNA sequence Mass: 32486 Score: 546 Matches: 13(7) Sequences: 12(7)

gi|659113223 hypersensitive-induced response protein 2 [*Cucumis melo*]

Database 2

Match to: Unigene48873_SALfmcTARAAPEI-3 Mass: 19474 Score: 304 Matches: 7(5) Sequences: 7(5)

gi| 525507222 hypersensitive-induced response protein 1-like [*Cucumis sativus*]

Matched peptides shown in bold red.

MGQALGCVQVGQSTVAISERFGKFNDVLNPGCHCVPWCLGSQVAGYLTLRVQQLDVRCETKTKDNVFVTVVASVQYRAVS
DNATDAFYKLSNTREQIQAYVFDVIRASVPKLELDSVFEQKNDIAKSVEQELEKAMSAYGYEIVQTLIVDIEPDVNVKRAMNEI
NAAARMRVA

Query	Observed	Mr(expt)	Mr(calc)	ppm	Miss	Score	Expect	Rank	Unique	Peptide
5	857.5049	856.4977	856.4767	24.5	0	19	0.34	1	U	R.VQQLDVR.C
9	1076.5481	1075.5408	1075.5080	30.5	0	21	0.28	1	U	R.AMNEINAAAR.M + Oxidation (M)
12	1207.6532	1206.6459	1206.6132	27.1	0	51	0.00024	1	U	K.LELDSVFEQK.N
17	1301.6411	1300.6338	1300.5935	31.0	0	31	0.02	1	U	R.AVSDNATDAFYK.L
22	1480.8178	1479.8105	1479.7722	25.9	0	59	2.6e-005	1	U	R.EQIQAYVFDVIR.A
25	1689.9465	1688.9393	1688.8985	24.1	1	40	0.002	1	U	R.ASVPKLELDSVFEQK.N
26	1826.0271	1825.0199	1824.9734	25.4	1	83	8.2e-008	1	U	K.TKDNVFVTVVASVQYR.A

2. Unigene47573_SALfmcTARAAPEI-3 Mass: 7694 Score: 206 Matches: 3(2) Sequences: 3(2)

gi|23345044 hypersensitive-induced reaction protein 2 [*Hordeum vulgare subsp. vulgare*]

Query	Observed	Mr(expt)	Mr(calc)	ppm	Miss	Score	Expect	Rank	Unique	Peptide
4	815.4616	814.4543	814.4297	30.2	0	22	0.2	2	U	R.DIGNQIR.D

19	1394.7895	1393.7822	1393.7466	25.5	0	104	9.2e-010	1	U	K.ASSVFIPHGPGAVR.D
24	1678.8706	1677.8633	1677.8210	25.2	0	79	2.5e-007	1	U	R.DSVLQFSENVPGTSAK.D

Spot 1999

Database 2

Match to: Unigene47573_SALfmcTARAAPEI-3 Mass: 7694 Score: 29 Matches: 1(1) Sequences: 1(1)

gi|23345044 hypersensitive-induced reaction protein 2 [*Hordeum vulgare subsp. vulgare*]

Matched peptides shown in bold red.

LRDSVLQFSENVPGTSAKDVMDMILITQYFDTMKEIGASSK**ASSVFIPHGPGAVR**DIGNQIRDGLLQAQTM

Query	Observed	Mr(expt)	Mr(calc)	ppm	Miss	Score	Expect	Rank	Unique	Peptide
8	1394.7238	1393.7165	1393.7466	-21.62	0	29	0.038	1	U	K.ASSVFIPHGPGAVR.D

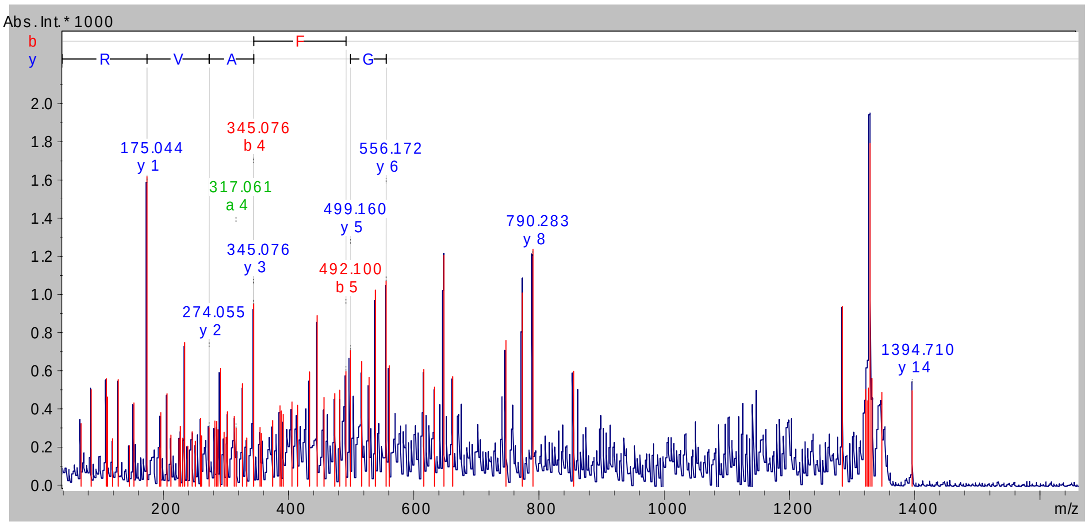

Spot 2000

Database 1

Match to: Unigene19093_Se200S transcribed RNA sequence Mass: 19288 Score: 56 Matches: 1(1) Sequences: 1(1)

gi| 151347473 hypersensitive-induced response protein [*Carica papaya*]

Matched peptides shown in bold red.

QAYVFDVIRATVPRSNVDDVFEQKDEIARAVAAELEKAMSHYGYEIVQTLIVDIEPDERVKRAMNEINAASRMRVAANDKAEA
EKILQIKKAEGEAEAKYLSGLGIARQRQAIVDGLRDSVLGFSVNVPGTNAKDVMDMILVTQYFDTMKEIGASSKASTVFIPHGP
GAVRDIATQ

Query	Observed	Mr(expt)	Mr(calc)	ppm	Miss	Score	Expect	Rank	Unique	Peptide
30	1764.8961	1763.8889	1763.8326	31.9	1	56	0.0001	1	U	R.SNVDDVFEQKDEIAR.A

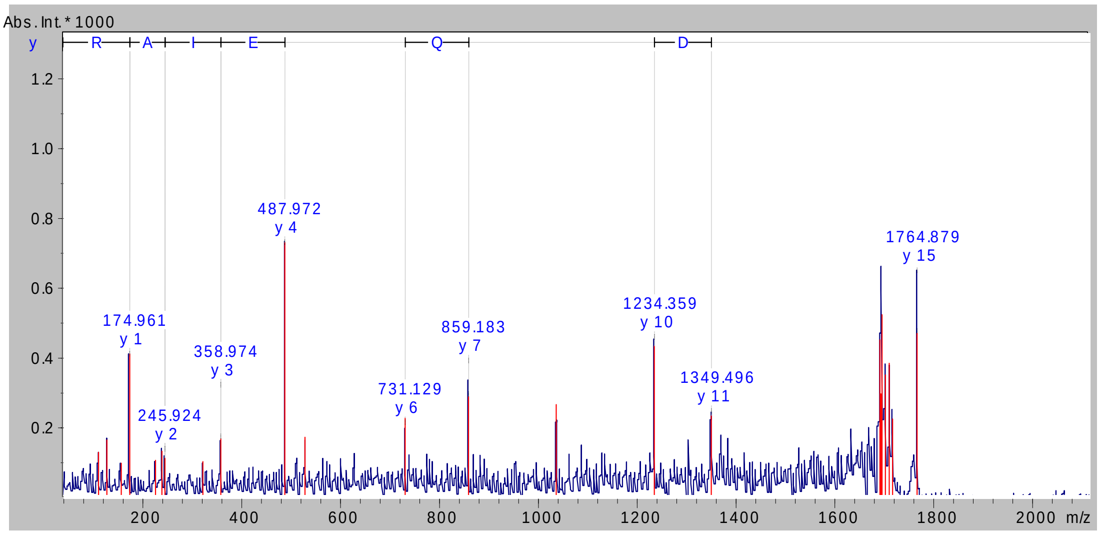

Proteins matching the same set of peptides:

Unigene38011_SeCKS transcribed RNA sequence Mass: 19332 Score: 56 Matches: 1(1) Sequences: 1(1)

gi|151347473 hypersensitive-induced response protein [*Carica papaya*]

Database 2

Match to: Unigene45044_SALfmcTARAAPEI-3 Mass: 17154 Score: 56 Matches: 1(1) Sequences: 1(1)

gi|151347473 hypersensitive-induced response protein [*Carica papaya*]

MGNLFCCFQVDQSTVAIKETFGKYDDVLEPGCHCVPWFIGQQIAGKLTLRVQQLDVRCETKTKDNVFVTVVASIQYRALADK

AGDAFYKLSNTRSQIQAYVFDVIRATVPRSNVDDVFEQKDEIARAVAAELEKAMSHYGYEIVQTLIVD

Query	Observed	Mr(expt)	Mr(calc)	ppm	Miss	Score	Expect	Rank	Unique	Peptide
30	1764.8961	1763.8889	1763.8326	31.9	1	56	5.1e-005	1	U	R.SNVDDVFEQKDEIAR.A

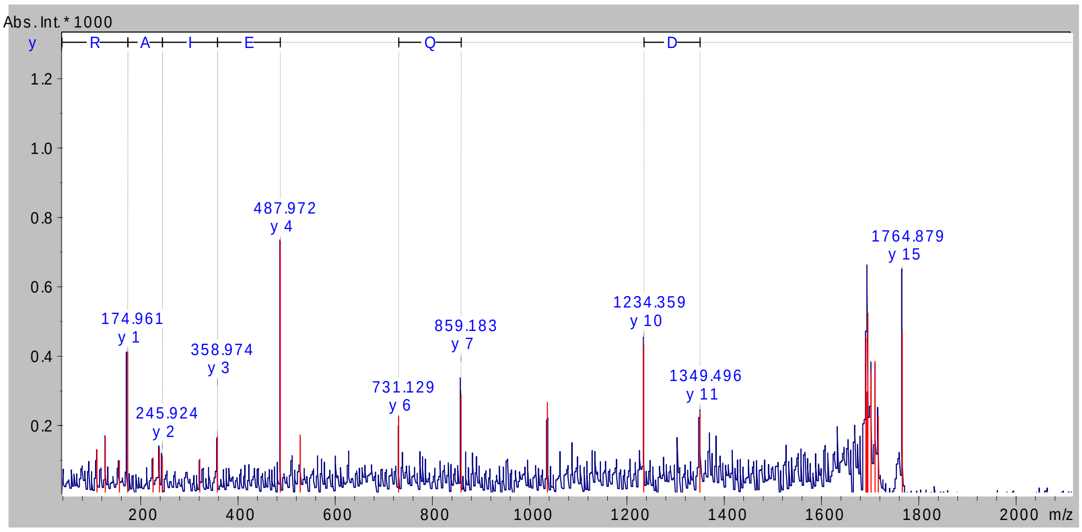

Spot 2406

NCBInr protein database

gi|527198851 Mass: 31717 Score: 217 Matches: 4(2) Sequences: 4(2)

hypothetical protein M569_07929, partial [*Genlisea aurea*]

Matched peptides shown in **bold red**.

1 LLVSKMGGVC CCVQVDQSTV AMKEQFGKFN EVLEPGCHCL PWCFGFQISG

51 SLSLRVQQID VRCETKTKDN VFVTVVASIQ YRALANKAAD AFYKLSNTR**E**

101 QIQAYVFDVI RASVPRLELD AAFEQKNDIA KAVEDELEKA MSAYGYEIVQ

151 TLIVDIEPDA QVKRAMNEIN AAARLRVAAN EKAEAEKILQ IKRAEGEAES

201 KYLSGLGIAR QRQAIVDGLR DSVLAFSENV PGTSAKDVMD MVLITQYFDT

251 MKEIGSTSKS SAVFIPHGPG AVRDIASQIR DGLLQ

Query	Observed	Mr(expt)	Mr(calc)	ppm	Miss	Score	Expect	Rank	Unique	Peptide
4	871.5125	870.5053	870.4923	14.9	0	34	1	1		R.QAIVDGLR.D
8	1076.5430	1075.5357	1075.5080	25.8	0	29	5	1		R.AMNEINAAAR.L + Oxidation (M)
17	1394.7708	1393.7635	1393.7466	12.1	0	80	2.4e-05	2	U	K.SSAVFIPHGPGAVR.D
18	1480.7955	1479.7883	1479.7722	10.9	0	74	0.00011	1		R.EQIQAYVFDVIR.A

Database 1

Match to: Unigene14982_SeCKS transcribed RNA sequence Mass: 32486 Score: 470 Matches: 12(5) Sequences: 11(5)

gi|525507222 hypersensitive-induced response protein 1-like [*Cucumis sativus*]

Matched peptides shown in bold red.

DKRTRIMGQALGCVQVGQSTVAISERFGKFNDVLNPGCHCVPWCLGSQVAGYLTLRVQQLDVRCETKTKDNVFVTVVASVQY

RAVSDNATDAFYKLSNTREQIQAYVFDVIRASVPKLELDSVFEQKNDIAKSVEQELEKAMSAYGYEIVQTLIVDIEPDVNVKRA

MNEINAAARMRVAANEKAEAEKILQIKKAEGEAESKYLGGMGIARQRQAIVDGLRDSVLQFSENVPGTSAKDVMDMILITQY

FDTMKEIGASSKASSVFIPHGPGAVRDIGNQIRDGLLQAQTMER

Query	Observed	Mr(expt)	Mr(calc)	ppm	Miss	Score	Expect	Rank	Unique	Peptide
2	815.4528	814.4455	814.4297	19.4	0	27	0.13	1	U	R.DIGNQIR.D
3	857.4948	856.4875	856.4767	12.6	0	22	0.39	2	U	R.VQQLDVR.C
4	871.5125	870.5053	870.4923	14.9	0	34	0.017	1	U	R.QAIVDGLR.D
5	937.4974	936.4902	936.4851	5.40	0	(12)	4.5	5	U	K.YLGGMGIAR.Q
6	953.4933	952.4861	952.4800	6.35	0	13	2.6	2	U	K.YLGGMGIAR.Q + Oxidation (M)
8	1076.5430	1075.5357	1075.5080	25.8	0	29	0.1	1	U	R.AMNEINAAAR.M + Oxidation (M)
10	1207.6381	1206.6309	1206.6132	14.6	0	63	3.2e-005	1	U	K.LELDSVFEQK.N
14	1277.6315	1276.6242	1276.6081	12.6	0	25	0.2	1	U	R.DGLLQAQTMER.- + Oxidation (M)
17	1394.7708	1393.7635	1393.7466	12.1	0	83	2.3e-007	1	U	K.ASSVFIPHGPGAVR.D
18	1480.7955	1479.7883	1479.7722	10.9	0	74	1.9e-006	1	U	R.EQIQAYVFDVIR.A
19	1678.8401	1677.8328	1677.8210	7.07	0	78	8.2e-007	1	U	R.DSVLQFSENVPGTSAK.D
20	1825.9937	1824.9865	1824.9734	7.15	1	22	0.22	1	U	K.TKDNVFVTVVASVQYR.A

Proteins matching the same set of peptides:

Unigene14983_SeCKS transcribed RNA sequence Mass: 32486 Score: 470 Matches: 12(5) Sequences: 11(5)

gi|659113223 hypersensitive-induced response protein 2 [*Cucumis melo*]

Unigene16990_Se200S transcribed RNA sequence Mass: 32486 Score: 470 Matches: 12(5) Sequences: 11(5)

gi|659113223 hypersensitive-induced response protein 2 [*Cucumis melo*]

Database 2

Match to: Unigene48873_SALfmcTARAAPEI-3 Mass: 19474 Score: 210 Matches: 5(3) Sequences: 5(3)

gi|525507222 hypersensitive-induced response protein 1-like [*Cucumis sativus*]

MGQALGCVQVGQSTVAISERFGKFNDVLNPGCHCVPWCLGSQVAGYLTLRVQQLDVRCETKTKDNVFVTVVASVQYRAVSD

NATDAFYKLSNTREQIQAYVFDVIRASVPKLELDSVFEQKNDIAKSVEQELEKAMSAYGYEIVQTLIVDIEPDVNVKRAMNEIN

AAARMRVA

Query	Observed	Mr(expt)	Mr(calc)	ppm	Miss	Score	Expect	Rank	Unique	Peptide

3	857.4948	856.4875	856.4767	12.6	0	22	0.18	2	U	R.VQQLDVR.C
8	1076.5430	1075.5357	1075.5080	25.8	0	29	0.049	1	U	R.AMNEINAAAR.M + Oxidation (M)
10	1207.6381	1206.6309	1206.6132	14.6	0	63	1.5e-005	1	U	K.LELDSVFEQK.N
18	1480.7955	1479.7883	1479.7722	10.9	0	74	9.2e-007	1	U	R.EQIQAYVFDVIR.A
20	1825.9937	1824.9865	1824.9734	7.15	1	22	0.12	1	U	K.TKDNVFVTVVASVQYR.A

2. Unigene47573_SALfmcTARAAPEI-3 Mass: 7694 Score: 188 Matches: 3(2) Sequences: 3(2)

gi|23345044 hypersensitive-induced reaction protein 2 [*Hordeum vulgare subsp. vulgare*]

Query	Observed	Mr(expt)	Mr(calc)	ppm	Miss	Score	Expect	Rank	Unique	Peptide
2	815.4528	814.4455	814.4297	19.4	0	27	0.068	1	U	R.DIGNQIR.D
17	1394.7708	1393.7635	1393.7466	12.1	0	83	1.5e-007	1	U	K.ASSVFIPHGPGAVR.D
19	1678.8401	1677.8328	1677.8210	7.07	0	78	3.4e-007	1	U	R.DSVLQFSENVPGTSAK.D

Spot 1663

NCBInr protein database

Match to: gi|23345046 Mass: 31806 Score: 205 Matches: 3(2) Sequences: 3(2)

hypersensitive-induced reaction protein 3 [*Hordeum vulgare subsp. vulgare*]

Matched peptides shown in bold red.

1 MGNLCCCVQV DQSTVAIREQ FGKFDSVLQP GCHCLPWIFG KRVVGHLTLR

51 LQQLDVRCET KTKDNVFVTV VASIQYRPLA GKESDAYYKL TNTRSQIQAY

101 VFDVIRASVP KLNLDDAFVQ KNDIAKAVED ELEKAMSAYG FEIVQTLIVD

151 IEPDAHVKQA MNEINAAARM RVAANEKAEA EKIVQIKRAE GEAEAKYLSG

201 LGIARQRQAI VDGLRDSVLG FAVNVPGTTA KDVMDMVLIT QYFDTMKEIG

251 ASSKSSAVFI PHGPGAVRDI ATQIRDGLLQ GQSASDN

Query	Observed	Mr(expt)	Mr(calc)	ppm	Miss	Score	Expect	Rank	Unique	Peptide
8	949.5544	948.5471	948.5392	8.29	0	63	0.0016	1		K.YLSGLGIAR.Q
14	1438.7829	1437.7756	1437.7616	9.73	0	102	1.8e-07	1	U	R.SQIQAYVFDVIR.A
19	1997.9051	1996.8978	1996.8832	7.29	0	40	0.2	3	U	K.DVMDMVLITQYFDTMK.E + 3 Oxidation (M)

Proteins matching the same set of peptides:

gi|115465785 Mass: 31886 Score: 205 Matches: 3(2) Sequences: 3(2)

Os05g0591900 [*Oryza sativa Japonica Group*]

gi|162462908 Mass: 31876 Score: 205 Matches: 3(2) Sequences: 3(2)

hypersensitive induced reaction3 [*Zea mays*]

gi|195625408 Mass: 31885 Score: 205 Matches: 3(2) Sequences: 3(2)

hypersensitive-induced response protein [*Zea mays*]

gi|208293677 Mass: 31823 Score: 205 Matches: 3(2) Sequences: 3(2)

hypersensitive induced response protein 3 [*Triticum aestivum*]

gi|223469622 Mass: 31743 Score: 205 Matches: 3(2) Sequences: 3(2)

hypersensitive induced response protein 3 [*Triticum aestivum*]

gi|357153791 Mass: 39541 Score: 205 Matches: 3(2) Sequences: 3(2)

PREDICTED: hypersensitive-induced response protein 1-like [*Brachypodium distachyon*]

gi|380447730 Mass: 31779 Score: 205 Matches: 3(2) Sequences: 3(2)

hypersensitive induced reaction protein 3 [*Triticum aestivum*]

gi|413946722 Mass: 36737 Score: 205 Matches: 3(2) Sequences: 3(2)

hypersensitive-induced response protein [*Zea mays*]

gi|514746035 Mass: 31854 Score: 205 Matches: 3(2) Sequences: 3(2)

PREDICTED: hypersensitive-induced response protein 1-like isoform X1 [*Setaria italica*]

gi|573945051 Mass: 31725 Score: 205 Matches: 3(2) Sequences: 3(2)

PREDICTED: hypersensitive-induced response protein 1-like isoform X1 [*Oryza brachyantha*]

Database 1

Match to: Unigene19093_Se200S transcribed RNA sequence Mass: 19288 Score: 568 Matches: 7(6) Sequences: 7(6)

gi|151347473 hypersensitive-induced response protein [*Carica papaya*]

Matched peptides shown in bold red.

QAYVFDVIRATVPRSNVDDVFEQKDEIARAVAAELEKAMSHYGYEIVQTLIVDIEPDERVKRAMNEINAASRMRVAANDKAEA

EKILQIKKAEGEAEAKYLSGLGIARQRQAIVDGLRDSVLGFSVNVPGTNAKDVMDMILVTQYFDTMKEIGASSKASTVFIPHGP

GAVRDIATQ

Query	Observed	Mr(expt)	Mr(calc)	ppm	Miss	Score	Expect	Rank	Unique	Peptide
8	949.5544	948.5471	948.5392	8.29	0	63	2.6e-005	1	U	K.YLSGLGIAR.Q
9	1092.5169	1091.5096	1091.5029	6.16	0	30	0.061	1	U	R.AMNEINAASR.M + Oxidation (M)
11	1180.5626	1179.5554	1179.5408	12.3	0	57	9.7e-005	1	U	R.SNVDDVFEQK.D
13	1408.7860	1407.7788	1407.7623	11.7	0	91	3e-008	1	U	K.ASTVFIPHGPGAVR.D
15	1604.8498	1603.8425	1603.8206	13.6	0	129	5.2e-012	1	U	R.DSVLGFSVNVPGTNAK.D
17	1764.8525	1763.8452	1763.8326	7.13	1	128	7.7e-012	1	U	R.SNVDDVFEQKDEIAR.A
19	1997.9051	1996.8978	1996.8832	7.29	0	70	3.1e-006	1	U	K.DVMDMILVTQYFDTMK.E + 3 Oxidation (M)

Database 2

Match to: Unigene15527_SALfmcTARAAPEI-3 Mass: 9070 Score: 290 Matches: 3(3) Sequences: 3(3)

gi|195625408 hypersensitive-induced response protein [*Zea mays*]

Matched peptides shown in bold red.

LSGLGIARQRQAIVDGLRDSVLGFSVNVPGTNAKDVMDMILVTQYFDTMKEIGASSKASTVFIPHGPGAVRDIATQIRDGLLQA

SS

Query	Observed	Mr(expt)	Mr(calc)	ppm	Miss	Score	Expect	Rank	Unique	Peptide
13	1408.7860	1407.7788	1407.7623	11.7	0	91	1.9e-008	1	U	K.ASTVFIPHGPGAVR.D
15	1604.8498	1603.8425	1603.8206	13.6	0	129	2.5e-012	1	U	R.DSVLGFSVNVPGTNAK.D
19	1997.9051	1996.8978	1996.8832	7.29	0	70	1.8e-006	1		K.DVMDMILVTQYFDTMK.E + 3 Oxidation (M)

2. Unigene45044_SALfmcTARAAPEI-3 Mass: 17154 Score: 287 Matches: 3(3) Sequences: 3(3)

gi|151347473 hypersensitive-induced response protein [*Carica papaya*]

Query	Observed	Mr(expt)	Mr(calc)	ppm	Miss	Score	Expect	Rank	Unique	Peptide
11	1180.5626	1179.5554	1179.5408	12.3	0	57	5.2e-005	1	U	R.SNVDDVFEQK.D
14	1438.7829	1437.7756	1437.7616	9.73	0	102	1.6e-009	1	U	R.SQIQAYVFDVIR.A
17	1764.8525	1763.8452	1763.8326	7.13	1	128	4e-012	1	U	R.SNVDDVFEQKDEIAR.A

3. Unigene48696_SALfmcTARAAPEI-3 Mass: 14690 Score: 100 Matches: 2(2) Sequences: 2(2)

gi|151347473 hypersensitive-induced response protein [*Carica papaya*]

Query	Observed	Mr(expt)	Mr(calc)	ppm	Miss	Score	Expect	Rank	Unique	Peptide
9	1092.5169	1091.5096	1091.5029	6.16	0	30	0.034	1	U	R.AMNEINAASR.M + Oxidation (M)

19 1997.9051 1996.8978 1996.8832 7.29 0 70 1.8e-006 1 K.DVMDMILVTQYFDTMK.E + 3 Oxidation (M)

Spot 1732

Database 1

Match to: Unigene14877_Se200S transcribed RNA sequence Mass: 25044 Score: 295 Matches: 6(4) Sequences: 6(4)

gi|255541538 Remorin, putative [*Ricinus communis*]

Matched peptides shown in bold red.

FFFFPFYSTNYIFCDHFPPKKKINNMGEEETKKVEEEKTPTAEESPKDVADEKRLVPVSDHKDSDFKPSPADEKAIVPVMTPPPA AETEKKPSGGSIDRDVVLAKVEQDKKLALVSAWEDSEKSKAENKACKKLSDVDAWETSRKATIEADLRAFEEKLEKKKAEYA ETMKNKVAEVHKAAQEKRAMIEAVKGEELLKAEECAAKFRATGHIPKKCFGCF

Matched peptides shown in bold red.

Query	Observed	Mr(expt)	Mr(calc)	ppm	Miss	Score	Expect	Rank	Unique	Peptide
2	818.3511	817.3438	817.3251	22.9	1	18	0.25	1	U	K.KCFGCF.-
5	1016.5878	1015.5805	1015.5662	14.1	1	28	0.09	1	U	R.KATIEADLR.A
12	1278.6114	1277.6041	1277.5888	12.0	0	51	0.00043	1	U	K.LSDVDAWETSR.K
13	1347.6933	1346.6860	1346.6718	10.6	0	73	3.5e-006	1	U	K.LALVSAWEDSEK.S
16	1406.6999	1405.6926	1405.6837	6.28	1	77	1.2e-006	1	U	K.KLSDVDAWETSR.K
19	1666.8818	1665.8746	1665.8647	5.89	0	49	0.00057	1	U	K.AIVPVMTPPPAAETEK.K + Oxidation (M)

Spot 2405

Database 1

Match to: Unigene483_SeCKS transcribed RNA sequence Mass: 23630 Score: 183 Matches: 4(3) Sequences: 4(3)

gi|255541538 Remorin, putative [*Ricinus communis*]

Matched peptides shown in bold red.

FFCDHFPPKKKINNMGEEETKKVEEEKTPTAEESPKDVADEKRLVPVSDHKDSDFKPSPADEKAIVPVMTPPPAAETEKKPSGG SIDRDVVLAKVEQDKKLALVSAWEDSEKSKAENKACKKLSDVDAWETSRKANIEADLRAFEEKLEKKKAEYAETMKNKVAE VHKAAQEKRAMIEAVKGEELLKAEECAAKFRATGHIPKKCFGCF

Query	Observed	Mr(expt)	Mr(calc)	ppm	Miss	Score	Expect	Rank	Unique	Peptide
8	1278.6219	1277.6146	1277.5888	20.2	0	48	0.00092	1	U	K.LSDVDAWETSR.K
9	1347.7064	1346.6992	1346.6718	20.3	0	43	0.0031	1	U	K.LALVSAWEDSEK.S
11	1406.7125	1405.7053	1405.6837	15.3	1	66	1.6e-005	1	U	K.KLSDVDAWETSR.K
15	1666.8914	1665.8841	1665.8647	11.6	0	28	0.08	1	U	K.AIVPVMTPPPAAETEK.K + Oxidation (M)

Proteins matching the same set of peptides:

Unigene14877_Se200S transcribed RNA sequence Mass: 25044 Score: 183 Matches: 4(3) Sequences: 4(3)

gi|255541538 Remorin, putative [*Ricinus communis*]

Spot 938

Database 1

Match to: Unigene12393_Se200S transcribed RNA sequence Mass: 37002 Score: 176 Matches: 7(2) Sequences: 7(2)

gi|566183896 TGF-beta receptor-interacting protein 1 [*Populus trichocarpa*]

Matched peptides shown in bold red.

NWRMRPILMKGHERPLTFLKYNRDGDLLFSCAKDHNPTVWFGDNGERLGTYRGHNGAVWSCDVSWDSSRLLTGSADTTVKL
WDVKKGEQLFTFKFDAPARAVEFSVGEKLAVMTTEPFMEKTSAIHVKRIAADIEDQDNESALVIKGSTARFYRAVWGPLNKTII
SCGEDALIRVWDPVTGQEIMSTQDLENGHTKAITSLCMSADKSHFLTGSIDKSVKLWDTRTLTLIKTYVTEQPVNAVALSPLLD
HIVLGGGQEASAVTTTDRRAGKFDAKFYDKVLQEEIGSVRGHFGPINALAFNPDGKSFVSGGEDGYVRLHHFDQDYFNIKL

Query	Observed	Mr(expt)	Mr(calc)	ppm	Miss	Score	Expect	Rank	Unique	Peptide
5	965.5088	964.5015	964.4866	15.5	0	18	1.1	1	U	R.AVEFSVGEK.L
7	1097.6228	1096.6155	1096.5917	21.7	1	2	23	2	U	K.KGEQLFTFK.F
8	1129.6521	1128.6448	1128.6139	27.4	0	27	0.09	1	U	K.VLQEEIGSVR.G
11	1197.7059	1196.6986	1196.6666	26.8	0	2	17	5	U	K.GHERPLTFLK.Y
14	1347.7401	1346.7328	1346.6864	34.5	0	35	0.016	1	U	K.TIISCGEDALIR.V
18	1643.7552	1642.7479	1642.7124	21.6	0	68	5.4e-006	1	U	K.DHNPTVWFGDNGER.L
21	1682.9197	1681.9125	1681.8675	26.7	1	23	0.18	1	U	K.FYDKVLQEEIGSVR.G

Database 2

Unigene56025_SALfmcTARAAPEI-3 Mass: 36572 Score: 176 Matches: 7(3) Sequences: 7(3)

gi|566183896 TGF-beta receptor-interacting protein 1 [*Populus trichocarpa*]

Matched peptides shown in bold red.

MRPILMKGHERPLTFLKYNRDGDLLFSCAKDHNPTVWFGDNGERLGTYRGHNGAVWSCDVSWDSSRLLTGSADTTVKLWD
VKKGEQLFTFKFDAPARAVEFSVGEKLAVMTTEPFMEKTSAIHVKRIAADIEDQDNESALVIKGSTARFYRAVWGPLNKTIISCG
EDALIRVWDPVTGQEIMSTQDLENGHTKAITSLCMSADKSHFLTGSIDKSVKLWDTRTLTLIKTYVTEQPVNAVALSPLLDHIVL
GGGQEAANVTTTDRRAGKFDAKFYDKVLQEEIGSVRGHFGPINALAFNPDGKSFVSGGEDGYVRLHHFDQDYFNIKL

Query	Observed	Mr(expt)	Mr(calc)	ppm	Miss	Score	Expect	Rank	Unique	Peptide
5	965.5088	964.5015	964.4866	15.5	0	18	0.48	1	U	R.AVEFSVGEK.L
7	1097.6228	1096.6155	1096.5917	21.7	1	2	14	3	U	K.KGEQLFTFK.F
8	1129.6521	1128.6448	1128.6139	27.4	0	27	0.044	1	U	K.VLQEEIGSVR.G
11	1197.7059	1196.6986	1196.6666	26.8	0	2	10	7	U	K.GHERPLTFLK.Y
14	1347.7401	1346.7328	1346.6864	34.5	0	35	0.0084	1	U	K.TIISCGEDALIR.V
18	1643.7552	1642.7479	1642.7124	21.6	0	68	3e-006	1	U	K.DHNPTVWFGDNGER.L
21	1682.9197	1681.9125	1681.8675	26.7	1	23	0.1	1	U	K.FYDKVLQEEIGSVR.G

Spot 2410

Database 2

Match to: Unigene13089_SALfmcTARAAPEI-3 Mass: 42178 Score: 34 Matches: 1(1) Sequences: 1(1)

gi|225424428 PREDICTED: G-type lectin S-receptor-like serine/threonine-protein kinase At1g34300-like [*Vitis vinifera*]

Matched peptides shown in bold red.

SFSFSNSSWHPSQNLTLVSPNSNFSAGFHPSPISPNLFIFSIWYSNYISDSSNHTIVWSANWGSPLQSSASLFISSNGDLQLINGTSS
GLTPNFWPDPPTQSTNSTRLLLRDDGNLVFGSUQSFDYPTDTFLPTQNITSSTVLRSRNGKFNFTGSKDLVFNGSDTYWSSSNGF
LSLESDGRVIRDSGDPTYSSDFGSVRTRRLTLGDDGNLRILSYDPDKKEWVVVWVALLEVCQVHGLCGPGYICYSDGTTVSGY
ECVCPPGLMKSSKSGSCVPKNPIIKYERSKFIRLDYVNYSXXXXXXXXXQTNDETIRFADCRSKCMKNQKCLGFGFKFDGSGY
CVLQINNLLNGYWSQDSEMSFYLRVDQSESDPSNF

Query	Observed	Mr(expt)	Mr(calc)	ppm	Miss	Score	Expect	Rank	Unique	Peptide
3	1036.6020	1035.5947	1035.5019	89.6	1	34	0.01	1	U	K.SSKSGSCVPK.N

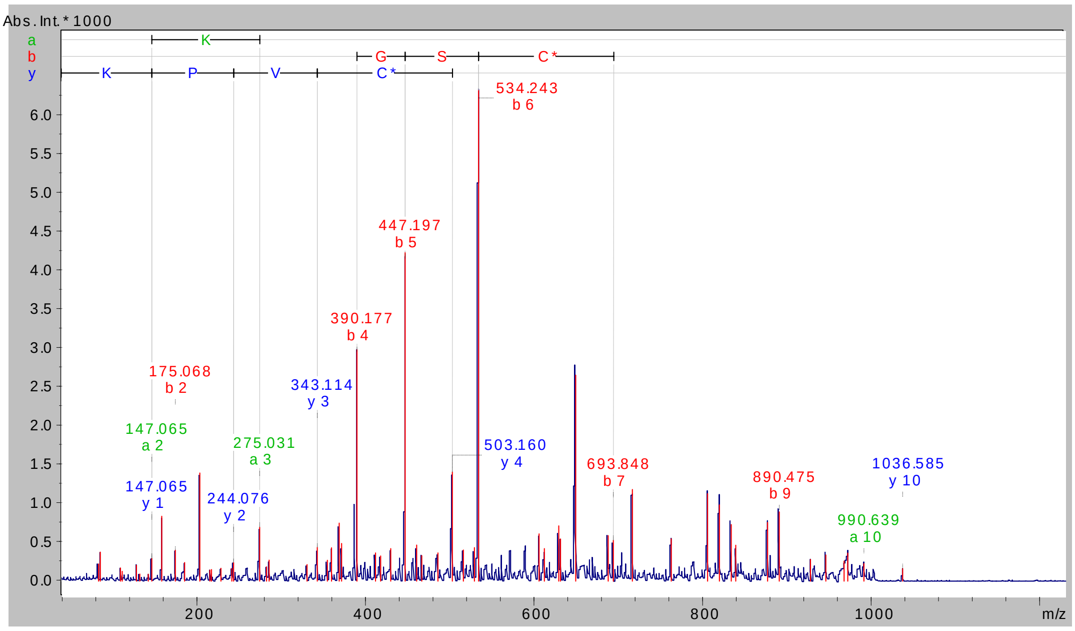

Spot 1922

NCBInr protein database

Match to: 1. gi|417093 Mass: 24641 Score: 52 Matches: 1(1) Sequences: 1(1)

RecName: Full=Glutathione S-transferase; AltName: Full=GST class-phi [*Silene vulgaris*]

Matched peptides shown in bold red.

1 MTIKVHGNPR STATQRVLVA LYEKHLEFEF VPIDMGAGGH KQPSYLALNP

51 FGQVPALEDG EIKLFESRAI TKYLAYTHDH QNEGTSLIHK EKHEMAAQLV

101 WEEVEAHQFD PVASKLAWEL VFKGIFGMQT DTTVVEENEA KLAK**VLDVYE**

151 **AR**LTESEYLG ANDSFTLVDL HHLPLLGYLM GTQVKKLFEE RAHVSAWCKK

201 ILARPSWEKT LALQKQA

Query	Observed	Mr(expt)	Mr(calc)	ppm	Miss	Score	Expect	Rank	Unique	Peptide
4	964.5165	963.5093	963.5025	6.98	0	52	0.021	1	U	K.VLDVYEAR.L

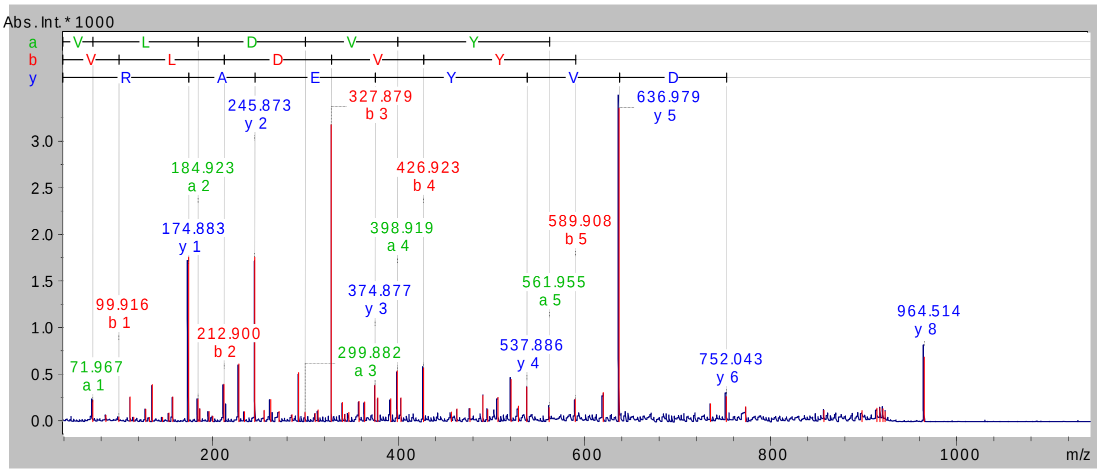

Proteins matching the same set of peptides:

gi|444341 Mass: 24657 Score: 52 Matches: 1(1) Sequences: 1(1)

glutathione S-transferase

gi|1402898 Mass: 23746 Score: 52 Matches: 1(1) Sequences: 1(1)

gst6 [*Arabidopsis thaliana*]

gi|2554769 Mass: 23983 Score: 52 Matches: 1(1) Sequences: 1(1)

Chain A, Structure Of Glutathione S-Transferase

gi|3193295 Mass: 11019 Score: 52 Matches: 1(1) Sequences: 1(1)

T14P8.11 [*Arabidopsis thaliana*]

gi|14532562 Mass: 19786 Score: 52 Matches: 1(1) Sequences: 1(1)

At2g47730/F17A22.12 [*Arabidopsis thaliana*]

gi|15227063 Mass: 24106 Score: 52 Matches: 1(1) Sequences: 1(1)

glutathione S-transferase F3 [*Arabidopsis thaliana*]

gi|15235401 Mass: 24114 Score: 52 Matches: 1(1) Sequences: 1(1)

glutathione S-transferase F2 [*Arabidopsis thaliana*]

gi|20197312 Mass: 24119 Score: 52 Matches: 1(1) Sequences: 1(1)

glutathione S-transferase (GST6) [*Arabidopsis thaliana*]

gi|21555418 Mass: 24036 Score: 52 Matches: 1(1) Sequences: 1(1)

Atpm24.1 glutathione S transferase [*Arabidopsis thaliana*]

gi|30690772 Mass: 29270 Score: 52 Matches: 1(1) Sequences: 1(1)

glutathione S-transferase phi 8 [*Arabidopsis thaliana*]

gi|31790097 Mass: 24068 Score: 52 Matches: 1(1) Sequences: 1(1)

glutathione S-transferase 3 [*Brassica juncea*]

gi|31790099 Mass: 24238 Score: 52 Matches: 1(1) Sequences: 1(1)

glutathione S-transferase 4 [*Brassica juncea*]

gi|31790103 Mass: 23907 Score: 52 Matches: 1(1) Sequences: 1(1)

glutathione S-transferase 5 [*Brassica juncea*]

gi|31790105 Mass: 23942 Score: 52 Matches: 1(1) Sequences: 1(1)

glutathione S-transferase 6 [*Brassica juncea*]

gi|110738331 Mass: 24134 Score: 52 Matches: 1(1) Sequences: 1(1)

putative glutathione S-transferase [*Arabidopsis thaliana*]

gi|117067088 Mass: 23889 Score: 52 Matches: 1(1) Sequences: 1(1)

glutathione-S-transferase [*Acanthus ebracteatus*]

gi|118406886 Mass: 23774 Score: 52 Matches: 1(1) Sequences: 1(1)

glutathione S-transferase [*Vitis vinifera*]

gi|121296516 Mass: 23955 Score: 52 Matches: 1(1) Sequences: 1(1)

glutathione S-transferase [*Brassica juncea*]

gi|225436695 Mass: 24295 Score: 52 Matches: 1(1) Sequences: 1(1)

PREDICTED: glutathione S-transferase PARB [*Vitis vinifera*]

gi|225436697 Mass: 24884 Score: 52 Matches: 1(1) Sequences: 1(1)

PREDICTED: glutathione S-transferase F13 [*Vitis vinifera*]

gi|225436699 Mass: 24857 Score: 52 Matches: 1(1) Sequences: 1(1)

PREDICTED: glutathione S-transferase F13 [*Vitis vinifera*]

gi|225437284 Mass: 23838 Score: 52 Matches: 1(1) Sequences: 1(1)

PREDICTED: glutathione S-transferase [*Vitis vinifera*]

gi|225437288 Mass: 23977 Score: 52 Matches: 1(1) Sequences: 1(1)

PREDICTED: glutathione S-transferase [*Vitis vinifera*]

gi|227206270 Mass: 12875 Score: 52 Matches: 1(1) Sequences: 1(1)

AT4G02520 [Arabidopsis thaliana]

gi|242061904 Mass: 25151 Score: 52 Matches: 1(1) Sequences: 1(1)

hypothetical protein SORBIDRAFT_04g022250 [*Sorghum bicolor*]

gi|242077196 Mass: 25784 Score: 52 Matches: 1(1) Sequences: 1(1)

hypothetical protein SORBIDRAFT_06g028630 [*Sorghum bicolor*]

gi|284520980 Mass: 24183 Score: 52 Matches: 1(1) Sequences: 1(1)

putative glutathione-s-transferase theta [*Jatropha curcas*]

gi|296086586 Mass: 48132 Score: 52 Matches: 1(1) Sequences: 1(1)

unnamed protein product [*Vitis vinifera*]

gi|297809899 Mass: 24045 Score: 52 Matches: 1(1) Sequences: 1(1)

hypothetical protein ARALYDRAFT_490313 [*Arabidopsis lyrata subsp. lyrata*]

gi|297817934 Mass: 24116 Score: 52 Matches: 1(1) Sequences: 1(1)

glutathione S-transferase 16 [*Arabidopsis lyrata subsp. lyrata*]

gi|315308160 Mass: 20449 Score: 52 Matches: 1(1) Sequences: 1(1)

glutathione S-transferase [*Ananas comosus*]

gi|326532130 Mass: 25054 Score: 52 Matches: 1(1) Sequences: 1(1)

predicted protein [*Hordeum vulgare subsp. vulgare*]

gi|357132149 Mass: 25028 Score: 52 Matches: 1(1) Sequences: 1(1)

PREDICTED: probable glutathione S-transferase GSTF1-like [*Brachypodium distachyon*]

gi|357134671 Mass: 24583 Score: 52 Matches: 1(1) Sequences: 1(1)

PREDICTED: glutathione S-transferase 4-like [*Brachypodium distachyon*]

gi|400530648 Mass: 24257 Score: 52 Matches: 1(1) Sequences: 1(1)

glutathione-S-transferase, partial [*Brassica rapa subsp. chinensis*]

gi|410994532 Mass: 16830 Score: 52 Matches: 1(1) Sequences: 1(1)

glutathion-S-transferase phi, partial [*Brassica oleracea*]

gi|460789893 Mass: 23914 Score: 52 Matches: 1(1) Sequences: 1(1)

glutathione S-transferase [*Musa acuminata AAA Group*]

gi|473847765 Mass: 25975 Score: 52 Matches: 1(1) Sequences: 1(1)

Glutathione S-transferase 4 [*Triticum urartu*]

gi|473847766 Mass: 17756 Score: 52 Matches: 1(1) Sequences: 1(1)

Glutathione S-transferase 4 [*Triticum urartu*]

gi|474329342 Mass: 22926 Score: 52 Matches: 1(1) Sequences: 1(1)

putative glutathione S-transferase GSTF1 [*Triticum urartu*]

gi|475505579 Mass: 24215 Score: 52 Matches: 1(1) Sequences: 1(1)

hypothetical protein F775_29002 [*Aegilops tauschii*]

gi|475577974 Mass: 28919 Score: 52 Matches: 1(1) Sequences: 1(1)

hypothetical protein F775_52443 [*Aegilops tauschii*]

gi|527203165 Mass: 23485 Score: 52 Matches: 1(1) Sequences: 1(1)

hypothetical protein M569_05047 [*Genlisea aurea*]

gi|557749550 Mass: 23926 Score: 52 Matches: 1(1) Sequences: 1(1)

glutathione S-transferase 6 protein [*Brassica oleracea var. italica*]

gi|565461296 Mass: 24254 Score: 52 Matches: 1(1) Sequences: 1(1)

hypothetical protein CARUB_v10001952mg [*Capsella rubella*]

gi|566159172 Mass: 23198 Score: 52 Matches: 1(1) Sequences: 1(1)

glutathione S-transferase family protein [*Populus trichocarpa*]

gi|566255736 Mass: 15863 Score: 52 Matches: 1(1) Sequences: 1(1)

hypothetical protein POPTR_0483s00200g, partial [*Populus trichocarpa*]

gi|566255740 Mass: 23182 Score: 52 Matches: 1(1) Sequences: 1(1)

hypothetical protein POPTR_0483s00220g [*Populus trichocarpa*]

gi|567161446 Mass: 24103 Score: 52 Matches: 1(1) Sequences: 1(1)

hypothetical protein EUTSA_v10028957mg [*Eutrema salsugineum*]

gi|567903568 Mass: 23981 Score: 52 Matches: 1(1) Sequences: 1(1)

hypothetical protein CICLE_v10024356mg, partial [*Citrus clementina*]

gi|568852904 Mass: 62853 Score: 52 Matches: 1(1) Sequences: 1(1)

PREDICTED: dentin sialophosphoprotein-like [*Citrus sinensis*]

gi|590718601 Mass: 24742 Score: 52 Matches: 1(1) Sequences: 1(1)

Glutathione S-transferase phi 8 [*Theobroma cacao*]

Database 1

Match to: Unigene15741_Se200S transcribed RNA sequence Mass: 24392 Score: 223 Matches: 4(4) Sequences: 3(3)

GI:390979559 glutathione S-transferase-like protein [*Dianthus caryophyllus*]

Matched peptides shown in bold red.

KIMGIKIHGTPESTNVLRVLAVAHEKELDYELVNVDMKSGAHKQEPFISINPFGQVPGFEDGDAKLFESRAITRYLAYTYENKGT

PLISKSGKEMADLAVWMEVEAHQFDPVSSKLAWELVYKGMFGLETDNAAVEENEAKLVKVLDVYEARLSKSKYLAGDSFTL

ADLHHLPNLHFLMGSKVKKLFDERPHVSAWCKDILARPSWEKTVALLKKD

Query	Observed	Mr(expt)	Mr(calc)	ppm	Miss	Score	Expect	Rank	Unique	Peptide
4	964.5165	963.5093	963.5025	6.98	0	52	0.0004	1	U	K.VLDVYEAR.L
10	1164.5748	1163.5675	1163.5499	15.2	0	64	2.6e-005	1	U	R.YLAYTYENK.G
26	1924.8869	1923.8796	1923.8520	14.4	0	(75)	1.1e-006	1	U	K.GMFGLETDNAAVEENEAK.L
27	1940.8888	1939.8815	1939.8469	17.8	0	107	6.9e-010	1	U	K.GMFGLETDNAAVEENEAK.L

\+ Oxidation (M)

Spot 1976

NCBInr protein database

Match to: gi|460405798 Mass: 21655 Score: 127 Matches: 2(1) Sequences: 2(1)

PREDICTED: flavoprotein WrbA-like [*Solanum lycopersicum*]

Matched peptides shown in bold red.

1 MATKIYIVYY STYGHVEKLA EEIKEGAASV EGVEAKLWQV PETLSEEVLA

51 KMSAPPKSDV PIITPQELAE ADGFVFGFPT RFGMMSAQFK AFLDATGGLW

101 RTQQLAGKPA GIFYSTGSQG GGQETTPLTA ITQLVHHGMI FVPIGYTFGA

151 GMFEMENVKG GSPYGAGTFA GDGSRQPSEL ELQQAHHQGK YIAAIAKKLK

201 GAA

Query	Observed	Mr(expt)	Mr(calc)	ppm	Miss	Score	Expect	Rank	Unique	Peptide
3	1062.4448	1061.4375	1061.4674	-28.16	0	11	1.4e+02	4	U	R.FGMMSAQFK.A + Oxidation (M)
8	1456.6045	1455.5972	1455.6379	-27.95	0	116	4.4e-09	1		K.GGSPYGAGTFAGDGSR.Q

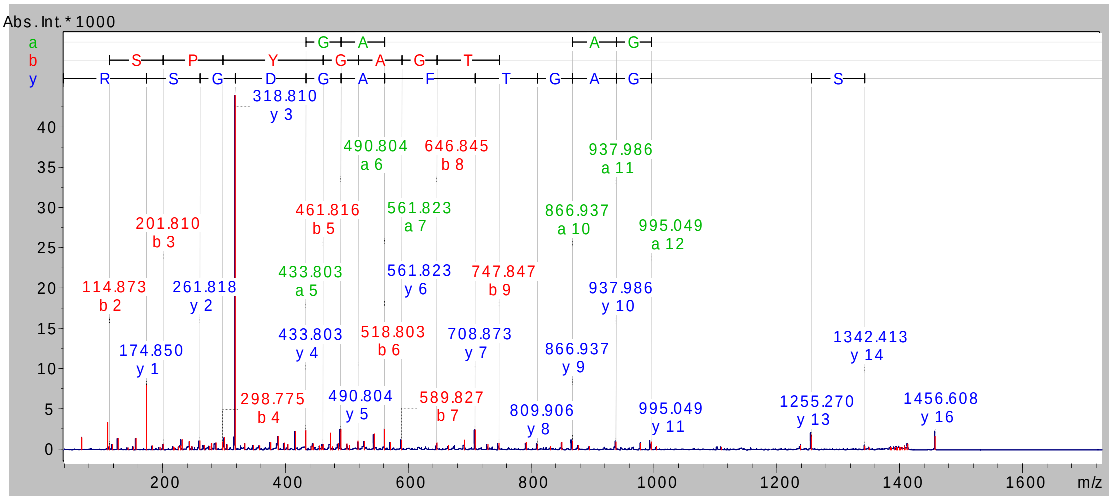

Proteins matching the same set of peptides:

gi|565394552 Mass: 21710 Score: 127 Matches: 2(1) Sequences: 2(1)

PREDICTED: minor allergen Alt a 7-like [*Solanum tuberosum*]

2. gi|3269288 Mass: 22355 Score: 126 Matches: 2(1) Sequences: 2(1)

putative protein [*Arabidopsis thaliana*]

Query	Observed	Mr(expt)	Mr(calc)	ppm	Miss	Score	Expect	Rank	Unique	Peptide
3	1062.4448	1061.4375	1061.4674	-28.16	0	10	1.8e+02	7	U	R.FGMMAAQFK.A + 2 Oxidation (M)
8	1456.6045	1455.5972	1455.6379	-27.95	0	116	4.4e-09	1		K.GGSPYGAGTFAGDGSR.Q

Proteins matching the same set of peptides:

gi|15239652 Mass: 21782 Score: 126 Matches: 2(1) Sequences: 2(1)

flavodoxin-like quinone reductase 1 [*Arabidopsis thaliana*]

gi|30687535 Mass: 21778 Score: 126 Matches: 2(1) Sequences: 2(1)

Quinone reductase family protein [*Arabidopsis thaliana*]

gi|37724581 Mass: 17612 Score: 126 Matches: 2(1) Sequences: 2(1)

putative quinone reductase, partial [*Vitis vinifera*]

gi|90811717 Mass: 21848 Score: 126 Matches: 2(1) Sequences: 2(1)

quinone-oxidoreductase QR2 [*Striga asiatica*]

gi|118485421 Mass: 21624 Score: 126 Matches: 2(1) Sequences: 2(1)

unknown [*Populus trichocarpa*]

gi|224115142 Mass: 21659 Score: 126 Matches: 2(1) Sequences: 2(1)

FLAVODOXIN-LIKE QUINONE REDUCTASE 1 family protein [*Populus trichocarpa*]

gi|225461209 Mass: 21726 Score: 126 Matches: 2(1) Sequences: 2(1)

PREDICTED: flavoprotein wrbA isoform 1 [*Vitis vinifera*]

gi|255555109 Mass: 21582 Score: 126 Matches: 2(1) Sequences: 2(1)

Flavoprotein wrbA, putative [*Ricinus communis*]

gi|297792905 Mass: 21872 Score: 126 Matches: 2(1) Sequences: 2(1)

hypothetical protein ARALYDRAFT_495531 [*Arabidopsis lyrata subsp. lyrata*]

gi|297799264 Mass: 21881 Score: 126 Matches: 2(1) Sequences: 2(1)

quinone reductase family protein [*Arabidopsis lyrata subsp. lyrata*]

gi|359493919 Mass: 20379 Score: 126 Matches: 2(1) Sequences: 2(1)

PREDICTED: flavoprotein wrbA isoform 2 [*Vitis vinifera*]

gi|449466103 Mass: 21735 Score: 126 Matches: 2(1) Sequences: 2(1)

PREDICTED: flavoprotein WrbA-like [*Cucumis sativus*]

gi|470116285 Mass: 21745 Score: 126 Matches: 2(1) Sequences: 2(1)

PREDICTED: flavoprotein WrbA-like [*Fragaria vesca subsp. vesca*]

gi|565434925 Mass: 21832 Score: 126 Matches: 2(1) Sequences: 2(1)

hypothetical protein CARUB_v10027151mg [*Capsella rubella*]

gi|565434927 Mass: 20513 Score: 126 Matches: 2(1) Sequences: 2(1)

hypothetical protein CARUB_v10027151mg [*Capsella rubella*]

gi|565445887 Mass: 22614 Score: 126 Matches: 2(1) Sequences: 2(1)

hypothetical protein CARUB_v10005724mg, partial [*Capsella rubella*]

gi|566154710 Mass: 21594 Score: 126 Matches: 2(1) Sequences: 2(1)

FLAVODOXIN-LIKE QUINONE REDUCTASE 1 family protein [*Populus trichocarpa*]

gi|567179332 Mass: 21846 Score: 126 Matches: 2(1) Sequences: 2(1)

hypothetical protein EUTSA_v10014706mg [*Eutrema salsugineum*]

gi|567218934 Mass: 20604 Score: 126 Matches: 2(1) Sequences: 2(1)

hypothetical protein EUTSA_v10026307mg [*Eutrema salsugineum*]

gi|567913699 Mass: 15767 Score: 126 Matches: 2(1) Sequences: 2(1)

hypothetical protein CICLE_v10016752mg [*Citrus clementina*]

gi|567913701 Mass: 21405 Score: 126 Matches: 2(1) Sequences: 2(1)

hypothetical protein CICLE_v10016752mg [*Citrus clementina*]

gi|567920206 Mass: 22278 Score: 126 Matches: 2(1) Sequences: 2(1)

hypothetical protein CICLE_v10009497mg [*Citrus clementina*]

gi|567920208 Mass: 21659 Score: 126 Matches: 2(1) Sequences: 2(1)

hypothetical protein CICLE_v10009497mg [*Citrus clementina*]

gi|567920210 Mass: 17601 Score: 126 Matches: 2(1) Sequences: 2(1)

hypothetical protein CICLE_v10009497mg [*Citrus clementina*]

gi|568827169 Mass: 21435 Score: 126 Matches: 2(1) Sequences: 2(1)

PREDICTED: minor allergen Alt a 7-like [*Citrus sinensis*]

gi|590625444 Mass: 22282 Score: 126 Matches: 2(1) Sequences: 2(1)

Quinone reductase family protein [*Theobroma cacao*]

gi|593698382 Mass: 21736 Score: 126 Matches: 2(1) Sequences: 2(1)

hypothetical protein PHAVU_005G088900g [*Phaseolus vulgaris*]

gi|593698384 Mass: 20419 Score: 126 Matches: 2(1) Sequences: 2(1)

hypothetical protein PHAVU_005G088900g [*Phaseolus vulgaris*]

gi|593698386 Mass: 16031 Score: 126 Matches: 2(1) Sequences: 2(1)

hypothetical protein PHAVU_005G088900g [*Phaseolus vulgaris*]

gi|595873985 Mass: 21589 Score: 126 Matches: 2(1) Sequences: 2(1)

hypothetical protein PRUPE_ppa011617mg [*Prunus persica*]

Database 1

Match to: Unigene19621_Se200S transcribed RNA sequence Mass: 21769 Score: 172 Matches: 4(2) Sequences: 4(2)

gi| 470116285 PREDICTED: flavoprotein WrbA-like [*Fragaria vesca subsp. vesca*]

Matched peptides shown in bold red.

GALMATKVYIVYYSMYGHVHTLAEEIQKGAASVEGVDAKLWQVPETLSDDALGKMSAPPKSDSPIISPTELADADGFIFGFPT

RFGMMAAQFKAFLDATGGLWRTQQLAGKPAGLFFSTGSQGGGQETTALTAITQLTHHGMIFVPIGYTFGAGMFEMENVKGGS
PYGAGTFAGDGSRQPTELELKQAFHQGQYIATITKKLKGGA

Query	Observed	Mr(expt)	Mr(calc)	ppm	Miss	Score	Expect	Rank	Unique	Peptide
3	1062.4448	1061.4375	1061.4674	-28.16	0	10	1.3	1	U	R.FGMMAAQFK.A + 2 Oxidation (M)
8	1456.6045	1455.5972	1455.6379	-27.95	0	116	3.3e-011	1	U	K.GGSPYGAGTFAGDGSR.Q
10	1605.8093	1604.8021	1604.8311	-18.09	0	32	0.032	1	U	K.QAFHQGQYIATITK.K
18	2553.2952	2552.2879	2552.2435	17.4	0	14	1.4	1	U	K.SDSPIISPTELADADGFIFGFPTR.F

Database 2

Match to: Unigene29794_SALfmcTARAAPEI-3 Mass: 4878 Score: 148 Matches: 2(2) Sequences: 2(2)

gi|15239652 flavodoxin-like quinone reductase 1 [*Arabidopsis thaliana*]

Matched peptides shown in bold red.

ENVKGGSPYGAGTFAGDGSRQPTELELKQAFHQGQYIATITKKLKG

Query	Observed	Mr(expt)	Mr(calc)	ppm	Miss	Score	Expect	Rank	Unique	Peptide
8	1456.6045	1455.5972	1455.6379	-27.95	0	116	3.4e-011	1	U	K.GGSPYGAGTFAGDGSR.Q
10	1605.8093	1604.8021	1604.8311	-18.09	0	32	0.014	1	U	K.QAFHQGQYIATITK.K

Spot 2409

NCBInr protein database

Match to: gi|37724581 Mass: 17612 Score: 220 Matches: 2(2) Sequences: 2(2)

putative quinone reductase, partial [*Vitis vinifera*]

Matched peptides shown in bold red.

1 WQVPETLPEE VLGKMSAPPK SDTPIITPTD LAEADGFVFG FPTRFGMMAA

51 QFKAFLDATG GLWRTQQLAG KPAGIFYSTG SQGGGQETTA LTAITQLVHH

101 GMIFVPIGYT FGAGMFEMEK VKGGSPYGAG TFAGDGSRQP SELELEQAFH

151 QGKYIAGITK KLKEAA

Query	Observed	Mr(expt)	Mr(calc)	ppm	Miss	Score	Expect	Rank	Unique	Peptide
12	1456.6677	1455.6604	1455.6379	0.0015	0	126	3.7e-09	1		K.GGSPYGAGTFAGDGSR.Q
18	1740.8667	1739.8594	1739.8478	0.0007	0	93	8e-06	1	U	R.QPSELELEQAFHQGK.Y

Proteins matching the same set of peptides:

gi|225461209 Mass: 21726 Score: 220 Matches: 2(2) Sequences: 2(2)

PREDICTED: flavoprotein wrbA isoform 1 [*Vitis vinifera*]

gi|359493919 Mass: 20379 Score: 220 Matches: 2(2) Sequences: 2(2)

PREDICTED: flavoprotein wrbA isoform 2 [*Vitis vinifera*]

gi|593698382 Mass: 21736 Score: 220 Matches: 2(2) Sequences: 2(2)

hypothetical protein PHAVU_005G088900g [*Phaseolus vulgaris*]

gi|593698384 Mass: 20419 Score: 220 Matches: 2(2) Sequences: 2(2)

hypothetical protein PHAVU_005G088900g [*Phaseolus vulgaris*]

gi|593698386 Mass: 16031 Score: 220 Matches: 2(2) Sequences: 2(2)

hypothetical protein PHAVU_005G088900g [*Phaseolus vulgaris*]

Database 1

Match to: Unigene32174_SeCKS transcribed RNA sequence Mass: 21739 Score: 383 Matches: 5(3) Sequences: 4(3)

gi| 297799264 quinone reductase family protein [*Arabidopsis lyrata subsp.lyrata*]

Matched peptides shown in bold red.

MATKVYIVYYSMYGHVEKLAEEIQKGAASVEGVEAKLWQVPETLSDDVLAKMSAPPKSDVSIITPNELPEADGLLFGFPTRFG

MMAAQFKAFMDATGGLWRTQQLAGKPAGIFYSTGSQGGGQETTPLTAITQLTHHGLIFVPIGYTFGAGMFEMEKVKGGSPYG

AGTFAGDGSRQPSELELEQAFHQGKYFAAIAKKLKATP

Query	Observed	Mr(expt)	Mr(calc)	ppm	Miss	Score	Expect	Rank	Unique	Peptide
4	1224.5987	1223.5915	1223.5757	12.9	0	(16)	1.4	1	U	K.AFMDATGGLWR.T
5	1240.5978	1239.5905	1239.5706	16.0	0	22	0.31	1	U	K.AFMDATGGLWR.T + Oxidation (M)
12	1456.6677	1455.6604	1455.6379	15.5	0	126	7.6e-012	1	U	K.GGSPYGAGTFAGDGSR.Q
18	1740.8667	1739.8594	1739.8478	6.64	0	93	2.4e-008	1	U	R.QPSELELEQAFHQGK.Y
25	2588.3383	2587.3311	2587.3170	5.44	0	141	2.1e-013	1	U	K.SDVSIITPNELPEADGLLFGFPTR.F

Database 2

Match to: Unigene49122_SALfmcTARAAPEI-3 Mass: 10303 Score: 220 Matches: 2(2) Sequences: 2(2)

gi| 460405798 flavoprotein WrbA-like [*Solanum lycopersicum*]

Matched peptides shown in bold red.

QQLAGKPAGIFYSTGSQGGGQETTPLTAITQLTHHGLIFVPIGYTFGAGMFEMEKVKGGSPYGAGTFAGDGSRQPSELELEQAF

HQGKYFAAIAKKLK

Query	Observed	Mr(expt)	Mr(calc)	ppm	Miss	Score	Expect	Rank	Unique	Peptide
12	1456.6677	1455.6604	1455.6379	15.5	0	126	4.8e-012	1	U	K.GGSPYGAGTFAGDGSR.Q
18	1740.8667	1739.8594	1739.8478	6.64	0	93	9.8e-009	1	U	R.QPSELELEQAFHQGK.Y

Spot 1226

NCBInr protein database

Match to: gi| 41350585 Mass: 25215 Score: 89 Matches: 2(2) Sequences: 1(1)

putative adenosine kinase [*Populus tremula x Populus alba*]

Matched peptides shown in bold red.

1 RXAQWMLQIP GATSYMGSIG KDKFGEEMKK NSTEAGVNVH YYEDEAAPTG

51 TCAVCVVGGE RSLIANLSAA NCYKSEHLKR PENWKLVEKA KYFYIAGFFL

101 TVSPESIMLV AEHAAANNKV FMMNLSAPFI CEFFKDVQEK ALPYMDYVFG

151 NETEARTFAK VHGWETENVE EIALKISQWP KASGAHKRIT VITQGSDPVV

201 VAEDGKVKLF PVILLPKEKL VDTNG

Query	Observed	Mr(expt)	Mr(calc)	ppm	Miss	Score	Expect	Rank	Unique	Peptide
20	1875.8695	1874.8622	1874.8509	6.06	0	(60)	0.0023	1	U	K.ALPYMDYVFGNETEAR.T
21	1891.8636	1890.8563	1890.8458	5.57	0	89	2.9e-06	1	U	K.ALPYMDYVFGNETEAR.T + Oxidation (M)

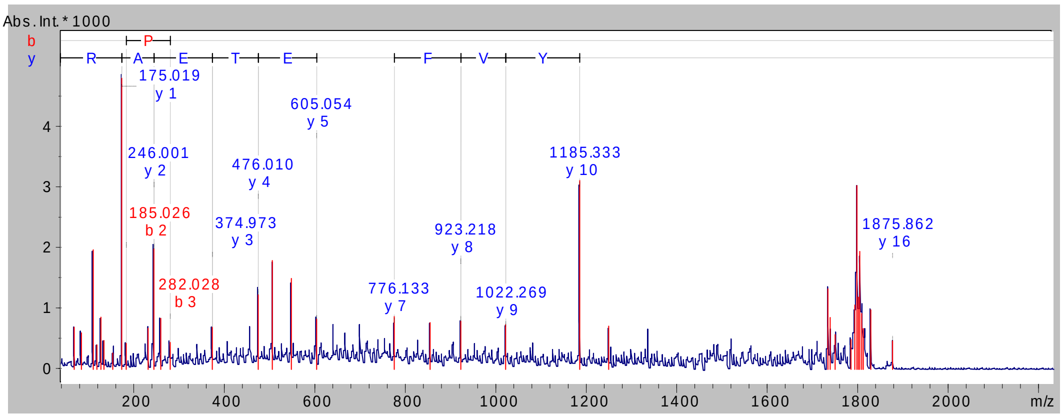

Proteins matching the same set of peptides:

gi|118484212 Mass: 37941 Score: 89 Matches: 2(2) Sequences: 1(1)

unknown [*Populus trichocarpa*]

gi|147812626 Mass: 27598 Score: 89 Matches: 2(2) Sequences: 1(1)

hypothetical protein VITISV_007608 [*Vitis vinifera*]

gi|217073276 Mass: 38022 Score: 89 Matches: 2(2) Sequences: 1(1)

unknown [*Medicago truncatula*]

gi|224109788 Mass: 37979 Score: 89 Matches: 2(2) Sequences: 1(1)

putative adenosine kinase family protein [*Populus trichocarpa*]

gi|225449016 Mass: 38300 Score: 89 Matches: 2(2) Sequences: 1(1)

PREDICTED: adenosine kinase 2 [*Vitis vinifera*]

gi|225449018 Mass: 38228 Score: 89 Matches: 2(2) Sequences: 1(1)

PREDICTED: adenosine kinase 2 [*Vitis vinifera*]

gi|296086001 Mass: 37353 Score: 89 Matches: 2(2) Sequences: 1(1)

unnamed protein product [*Vitis vinifera*]

gi|296086003 Mass: 37281 Score: 89 Matches: 2(2) Sequences: 1(1)

unnamed protein product [*Vitis vinifera*]

gi|357440379 Mass: 38082 Score: 89 Matches: 2(2) Sequences: 1(1)

Adenosine kinase [*Medicago truncatula*]

gi|357440381 Mass: 35497 Score: 89 Matches: 2(2) Sequences: 1(1)

Adenosine kinase [*Medicago truncatula*]

gi|388514649 Mass: 38110 Score: 89 Matches: 2(2) Sequences: 1(1)

unknown [*Medicago truncatula*]

gi|449464370 Mass: 37884 Score: 89 Matches: 2(2) Sequences: 1(1)

PREDICTED: adenosine kinase 2-like [*Cucumis sativus*]

gi|449530670 Mass: 35256 Score: 89 Matches: 2(2) Sequences: 1(1)

PREDICTED: LOW QUALITY PROTEIN: adenosine kinase 2-like, partial [*Cucumis sativus*]

gi|470137378 Mass: 37709 Score: 89 Matches: 2(2) Sequences: 1(1)

PREDICTED: adenosine kinase 2-like [*Fragaria vesca subsp. vesca*]

gi|567887850 Mass: 37943 Score: 89 Matches: 2(2) Sequences: 1(1)

hypothetical protein CICLE_v10032021mg [*Citrus clementina*]

gi|590565172 Mass: 38160 Score: 89 Matches: 2(2) Sequences: 1(1)

Adenosine kinase 2 [*Theobroma cacao*]

gi|596003580 Mass: 37952 Score: 89 Matches: 2(2) Sequences: 1(1)

hypothetical protein PRUPE_ppa008210mg [*Prunus persica*]

Database 1

Match to: Unigene15604_SeCKS transcribed RNA sequence Mass: 41740 Score: 155 Matches: 6(3) Sequences: 5(2)

gi| 224100781 putative adenosine kinase family protein [*Populus trichocarpa*]

Matched peptides shown in **bold red**.

HPCFRSESVLTLLLPFSYILPPSSSSFILPFSLIMETDGILLGMGNPLLDISAHVDNEFLQKYDIKLNNAILAEDKHLPMYGEMSEK
FKVDFIAGGATQNSIRVAQWMLQKPGATSFIGCIGKDKFGEEMKRDASNAGLNAQYYEHESSPTGTCAVCVVDGER**SLVANLS
AANCYK**VDHLKKPENWALVEKAKFYYIAGFFLTVSPETIQLVAEHAAANDKCFMMNLSAPFICEFFKEPQEK**ALPYMDYVFG
NETEAR**AFSKAHGWETENVEEIAIKISRWPKASGTHKRITVITQGADPVVVAEDGKVTTFPVAVLPKEKLVDTNGAGDAFVGG
FLSQLLEGKAIEDCVK**AGNYAANVVIQR**SGCTYPEKPDFK

Query	Observed	Mr(expt)	Mr(calc)	ppm	Miss	Score	Expect	Rank	Unique	Peptide
8	1275.6839	1274.6767	1274.6731	2.79	0	42	0.0039	1	U	K.AGNYAANVVIQR.S
10	1410.6901	1409.6828	1409.6973	-10.24	0	15	1.7	1	U	R.SLVANLSAANCYK.V
12	1448.7529	1447.7456	1447.7419	2.54	0	5	20	10	U	K.VDFIAGGATQNSIR.V
18	1723.9144	1722.9071	1722.9053	1.06	1	5	14	5	U	K.FKVDFIAGGATQNSIR.V
20	1875.8695	1874.8622	1874.8509	6.06	0	(60)	3.6e-005	1	U	K.ALPYMDYVFGNETEAR.A
21	1891.8636	1890.8563	1890.8458	5.57	0	89	4.3e-008	1	U	K.ALPYMDYVFGNETEAR.A

+ Oxidation (M)

Proteins matching the same set of peptides:

Unigene19479_Se200S transcribed RNA sequence Mass: 42193 Score: 155 Matches: 6(3) Sequences: 5(2)

gi|224100781 putative adenosine kinase family protein [Populus trichocarpa]

Spot 1812

Database 1

Match to: Unigene18365_SeCKS transcribed RNA sequence Mass: 24399 Score: 61 Matches: 3(0) Sequences: 3(0)

gi| 226491888 ras-related protein Rab11C [*Zea mays*]

Matched peptides shown in **bold red**.

PKKKMTR**KPEEEYDYLFK**VVLIGDSGVGKSNLLSRFTRNEFCLESK**STIGVEFATR**TLQVEGRTVK**AQIWDTAGQER**YRAITSA
YYRGALGALLVYDVTKPTTFDNVSRWLKELRDHADANIVIMLIGNKTDLKHLRAVATEDAQSFAEKEGLSFIETSALDATNVE
KAFQTILSEIYRIISKKSLSSNEAPAAPLGGGQTIDVGATTENNGKKSCCSSS

Query	Observed	Mr(expt)	Mr(calc)	ppm	Miss	Score	Expect	Rank	Unique	Peptide
3	1080.5721	1079.5648	1079.5611	3.43	0	7	12	4	U	K.STIGVEFATR.T
9	1274.6221	1273.6148	1273.6051	7.64	0	27	0.11	1	U	K.AQIWDTAGQER.Y
11	1460.7002	1459.6930	1459.6871	4.04	0	27	0.11	1	U	R.KPEEEYDYLFK.V

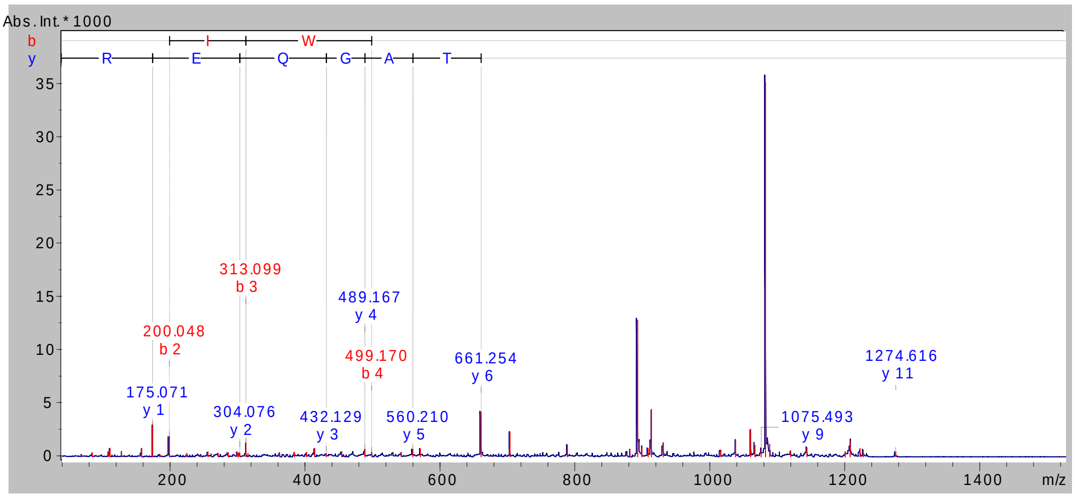

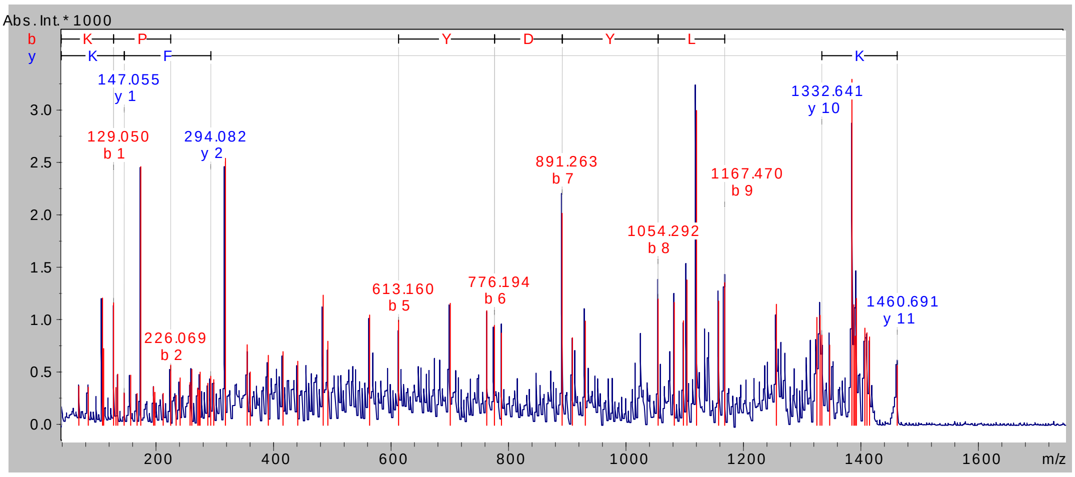

Proteins matching the same set of peptides:

Unigene78016_Se200S transcribed RNA sequence Mass: 24413 Score: 61 Matches: 3(0) Sequences: 3(0)

gi| 226491888 ras-related protein Rab11C [*Zea mays*]

Database 2

Match to: Unigene51906_SALfmcTARAAPEI-3 Mass: 23887 Score: 61 Matches: 3(1) Sequences: 3(1)

gi| 226491888 ras-related protein Rab11C [*Zea mays*]

Matched peptides shown in **bold red**.

MTR**KPEEEYDYLFK**VVLIGDSGVGKSNLLSRFTRNEFCLESK**STIGVEFATR**TLQVEGRTVK**AQIWDTAGQER**YRAITSAYYRG

ALGALLVYDVTKPTTFDNVSRWLKELRDHADANIVIMLIGNKTDLKHLRAVATEDAQSFAEKEGLSFIETSALDATNVEKAFQT

ILSEIYRIISKKSLSSNEAPAAPLGGGQTIDVGAATENNGKKSCCSSS

Query	Observed	Mr(expt)	Mr(calc)	ppm	Miss	Score	Expect	Rank	Unique	Peptide
3	1080.5721	1079.5648	1079.5611	3.43	0	7	5.6	5	U	K.STIGVEFATR.T
9	1274.6221	1273.6148	1273.6051	7.64	0	27	0.055	1	U	K.AQIWDTAGQER.Y
11	1460.7002	1459.6930	1459.6871	4.04	0	27	0.056	1	U	R.KPEEEYDYLFK.V

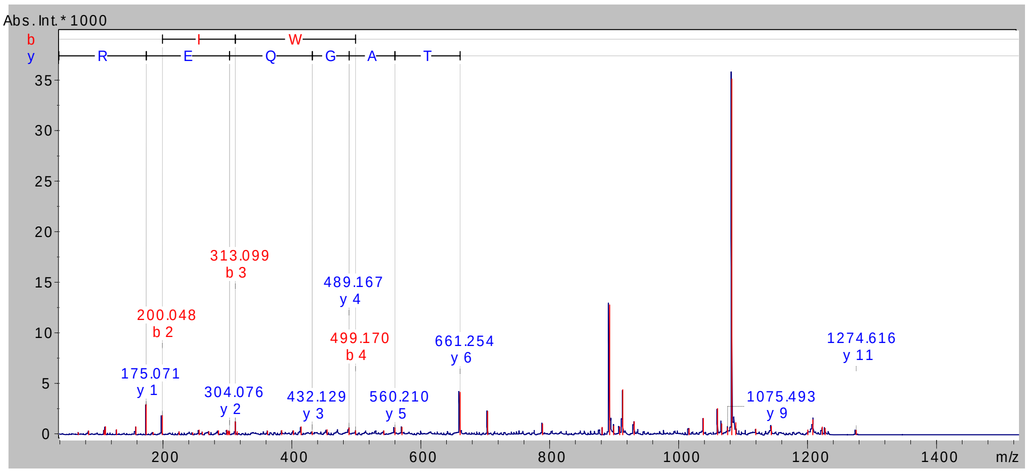

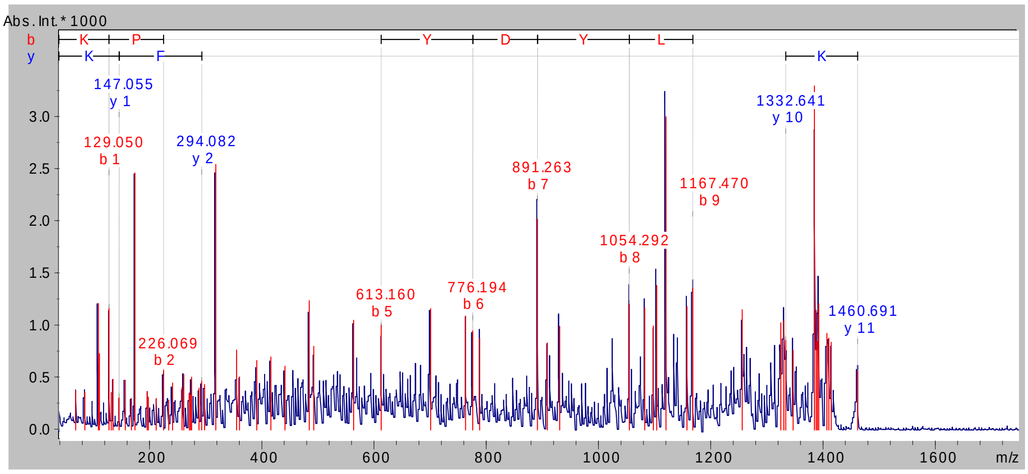

Spot 1921

NCBInr protein database

Match to: gi|224103431 Mass: 23354 Score: 108 Matches: 3(1) Sequences: 3(1)

RAS-related GTP-binding family protein [*Populus trichocarpa*]

Matched peptides shown in **bold red**.

1 MPSRRRTLLK VIILGDSGVG KTSLMNQYVN KKFSNQYKAT IGADFLTKEV

51 QFEDRLFTLQ IWDTAGQER**F QSLGVAFYR**G ADCCVLVYDV NSMKSFDNLN

101 NWREEFLIQA SPSDPENFPF VVLGNKVDVD GGNSRVVSEK KARAWCASK**G**

151 **NIPYFETSAK EGINVEEAFQ CIAK**NALKSG EEEEIYLPDT IDVGTSSQPR

201 STGCEC

Query	Observed	Mr(expt)	Mr(calc)	ppm	Miss	Score	Expect	Rank	Unique	Peptide
5	1187.6316	1186.6243	1186.6135	9.13	0	25	16	2		R.FQSLGVAFYR.G
8	1226.6135	1225.6063	1225.5979	6.84	0	17	57	1		K.GNIPYFETSAK.E
11	1607.7853	1606.7781	1606.7661	7.46	0	66	0.00057	1	U	K.EGINVEEAFQCIAK.N

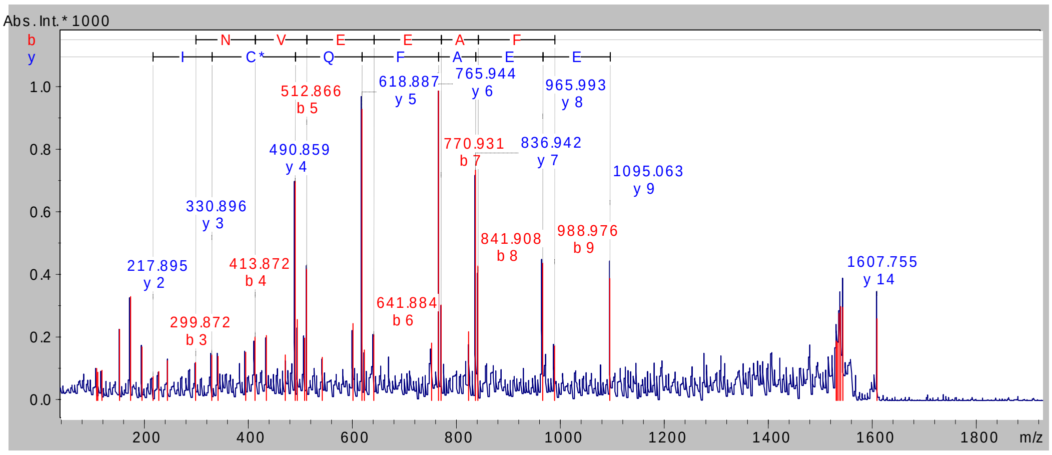

Proteins matching the same set of peptides:

gi|255536817 Mass: 23354 Score: 108 Matches: 3(1) Sequences: 3(1)

protein with unknown function [*Ricinus communis*]

gi|297741446 Mass: 28657 Score: 108 Matches: 3(1) Sequences: 3(1)

unnamed protein product [*Vitis vinifera*]

gi|351726788 Mass: 23380 Score: 108 Matches: 3(1) Sequences: 3(1)

uncharacterized protein LOC100305642 [*Glycine max*]

gi|359475302 Mass: 23370 Score: 108 Matches: 3(1) Sequences: 3(1)

PREDICTED: ras-related protein Rab7-like [*Vitis vinifera*]

gi|388507798 Mass: 23425 Score: 108 Matches: 3(1) Sequences: 3(1)

unknown [*Lotus japonicus*]

gi|388509354 Mass: 23325 Score: 108 Matches: 3(1) Sequences: 3(1)

unknown [*Lotus japonicus*]

gi|408784807 Mass: 23384 Score: 108 Matches: 3(1) Sequences: 3(1)

small GTP binding protein [*Arachis hypogaea*]

gi|532691792 Mass: 23366 Score: 108 Matches: 3(1) Sequences: 3(1)

ras-related protein Rab-7b [*Cucumis sativus*]

gi|567861184 Mass: 23353 Score: 108 Matches: 3(1) Sequences: 3(1)

hypothetical protein CICLE_v10029292mg [*Citrus clementina*]

gi|587874442 Mass: 22409 Score: 108 Matches: 3(1) Sequences: 3(1)

Ras-related protein Rab7 [*Morus notabilis*]

Database 1

Match to: Unigene2495_Se200S transcribed RNA sequence Mass: 24454 Score: 138 Matches: 4(1) Sequences: 4(1)

gi| 532691792 ras-related protein Rab-7b [*Cucumis sativus*]

Matched peptides shown in **bold red**.

IAEFQHSSKMSSRRRTLLKVIILGDSGVGKTSLMNQYVNKKFSNQYKATIGADFLTK**EVQFDDR**LFTLQIWDTAGQER**FQSLGVAFYR**GADCCVLVYDVNSMKSFDNLNIWREEFLIQASPSDPENFPFVVIGNKIDVDGGNSRVVPEKKARAWCASK**GNIPYFETSAKEGLNVEEAFQCIAK**NALKSSEEEEIYLPDTIDVANNNQQTSTGCDC

Query	Observed	Mr(expt)	Mr(calc)	ppm	Miss	Score	Expect	Rank	Unique	Peptide
3	908.4260	907.4187	907.4036	16.7	0	30	0.058	1	U	K.EVQFDDR.L
5	1187.6316	1186.6243	1186.6135	9.13	0	25	0.21	1	U	R.FQSLGVAFYR.G
8	1226.6135	1225.6063	1225.5979	6.84	0	17	1.1	1	U	K.GNIPYFETSAK.E

11	1607.7853	1606.7781	1606.7661	7.46	0	66	1.2e-005	1	U	K.EGLNVEEAFQCIAK.N

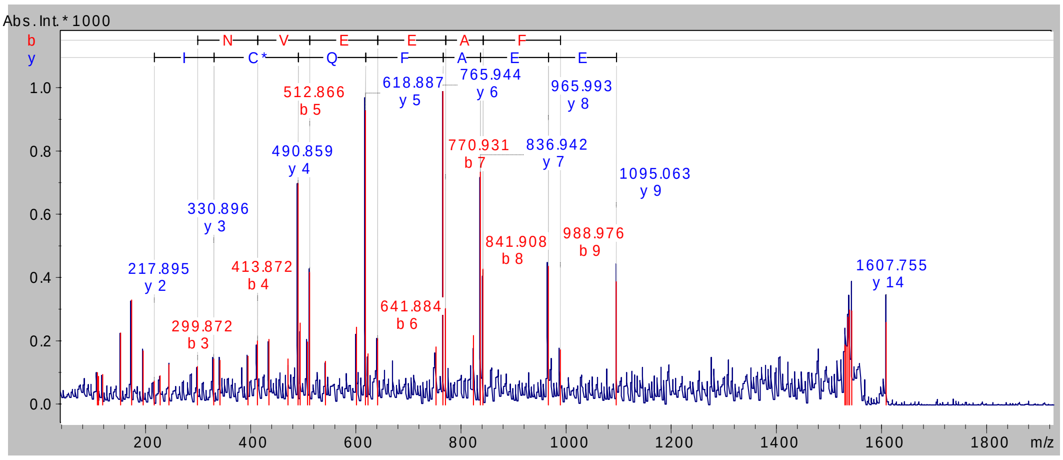

Proteins matching the same set of peptides:

Unigene11869_SeCKS transcribed RNA sequence Mass: 22662 Score: 138 Matches: 4(1) Sequences: 4(1)

gi|224103431 RAS-related GTP-binding family protein [Populus trichocarpa]

Database 2

Match to: Unigene52218_SALfmcTARAAPEI-3 Mass: 23397 Score: 138 Matches: 4(2) Sequences: 4(2)

gi| 532691792 ras-related protein Rab-7b [*Cucumis sativus*]

Matched peptides shown in **bold red**.

MSSRRRTLLKVIILGDSGVGKTSLMNQYVNKKFSNQYKATIGADFLTK**EVQFDDR**LFTLQIWDTAGQER**FQSLGVAFYR**GADC
CVLVYDVNSMKSFDNLNNWREEFLIQASPSDPENFPFVVIGNKIDVDGGNSRVVPEKKARAWCASK**GNIPYFETSAKEGLNVE**
EAFQCIAKNALKSGEEEEIYLPDTIDVANNNQQTSTGCDC

Query	Observed	Mr(expt)	Mr(calc)	ppm	Miss	Score	Expect	Rank	Unique	Peptide
3	908.4260	907.4187	907.4036	16.7	0	30	0.032	1	U	K.EVQFDDR.L
5	1187.6316	1186.6243	1186.6135	9.13	0	25	0.1	1	U	R.FQSLGVAFYR.G
8	1226.6135	1225.6063	1225.5979	6.84	0	17	0.5	1	U	K.GNIPYFETSAK.E
11	1607.7853	1606.7781	1606.7661	7.46	0	66	5.8e-006	1	U	K.EGLNVEEAFQCIAK.N

Spot 1541

Database 1

Match to: Unigene11714_Se200S transcribed RNA sequence Mass: 22745 Score: 157 Matches: 4(3) Sequences: 4(3)

gi| 38564727 putative alpha-soluble NSF attachment protein [*Helianthus annuus*]

Matched peptides shown in **bold red**.

REQGRENTLQRKMSDNIARGDEFVKKADKKLSGWGLFGNKHEDAAELFEKASNCYKLAKSWDKAGETYLKLADCYVKVDS
K**HEAANAFAEAGHAYK**KTSSK**EAVSCLEQSVNSFLEIGR**LNMGARYCKEIAEIFETDQNLDNAIVWHEKAGDLFQSEDVTTSA
NQCRQK**VAQYAAQLEQYPKAIEIYEDIAR**QSLNNTL

Query	Observed	Mr(expt)	Mr(calc)	ppm	Miss	Score	Expect	Rank	Unique	Peptide
2	1192.6142	1191.6069	1191.6135	-5.53	0	45	0.0022	1	U	K.AIEIYEDIAR.Q
10	1508.7646	1507.7573	1507.7671	-6.49	0	45	0.0018	1	U	K.VAQYAAQLEQYPK.A
12	1586.7226	1585.7153	1585.7273	-7.58	0	57	7e-005	1	U	K.HEAANAFAEAGHAYK.K
17	2037.9657	2036.9584	2036.9837	-12.39	0	11	3	1	U	

K.EAVSCLEQSVNSFLEIGR.L

Proteins matching the same set of peptides:

Unigene18948_SeCKS transcribed RNA sequence Mass: 24907 Score: 157 Matches: 4(3) Sequences: 4(3)

gi|38564727 putative alpha-soluble NSF attachment protein [*Helianthus annuus*]

Database 2

Match to: Unigene43231_SALfmcTARAAPEI-3 Mass: 16015 Score: 208 Matches: 3(3) Sequences: 3(3)

gi| 38564727 putative alpha-soluble NSF attachment protein [*Helianthus annuus*]

Matched peptides shown in bold red.

QNLDNAIAWHEKAADLFQSEDVTTSANQCRQKVAQYAAELGQYPKAIEIYEDIARQSLNNTLLKYGVKGHLLNAGLCHLCKG

DVVSITNALEKYQDLDPTFAGTREYRLLSDLAAAIDEQDVGKFTEVVKEFDSMTPLDAWK

Query	Observed	Mr(expt)	Mr(calc)	ppm	Miss	Score	Expect	Rank	Unique	Peptide
2	1192.6142	1191.6069	1191.6135	-5.53	0	45	0.00099	1	U	K.AIEIYEDIAR.Q
6	1383.6492	1382.6419	1382.6467	-3.43	0	53	9.5e-005	1	U	K.YQDLDPTFAGTR.E
16	2012.8730	2011.8657	2011.8905	-12.33	0	110	1.5e-010	1	U	K.AADLFQSEDVTTSANQCR.Q

Spot 1566

NCBInr protein database

Match to: gi|527205599 Mass: 32849 Score: 61 Matches: 1(1) Sequences: 1(1)

hypothetical protein M569_03331 [*Genlisea aurea*]

Matched peptides shown in bold red.

1 MGDHVARGEE FEKKAEKKLS GWGIFGSKYE DAADLFDKAA NSYKLGKSWN

51 QAGAVYVRLA DCHLKLDSKH EAASAFADAA HCYKKSNLKE SISCLEQSVN

101 IFLDIGRLNM SARYYKEIGE LYEQEQNLEQ AIAYFEKAAD LFQSEEVTTS

151 ANQCRQKVAQ FAAQLEQYQK AIEIYEDIAR QSLSNNLLKY GVKGHLLNAG

201 ICQLCRGDVV AITNALEKYQ DLDPTFSGTR EYKLLSDIAS AVEEEDVAKF

251 TSAVKEYDSM TQLDAWRTTL LLRVKEALKA KEMEDDDLT

Query	Observed	Mr(expt)	Mr(calc)	ppm	Miss	Score	Expect	Rank	Unique	Peptide
7	1192.6579	1191.6506	1191.6135	31.1	0	61	0.0023	1	U	K.AIEIYEDIAR.Q

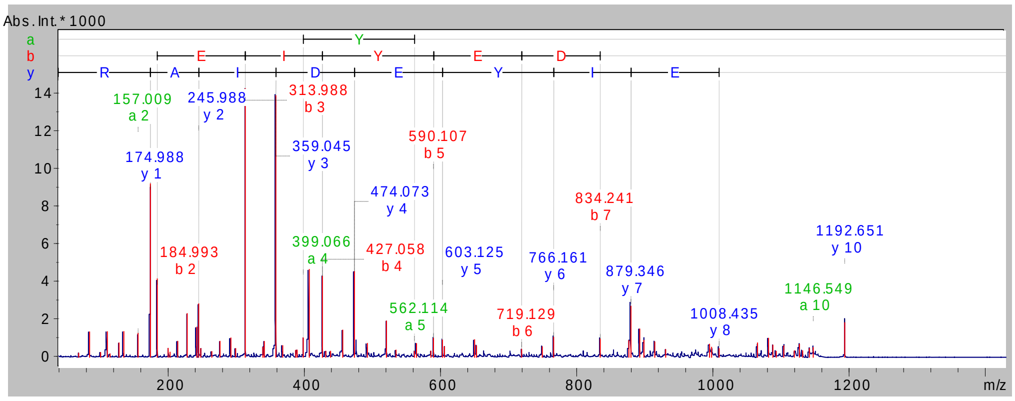

Database 1

Match to: Unigene11714_Se200S transcribed RNA sequence Mass: 22745 Score: 207 Matches: 4(4) Sequences: 4(4)

gi| 38564727 putative alpha-soluble NSF attachment protein [*Helianthus annuus*]

Matched peptides shown in bold red.

REQGRENTLQRKMSDNIARGDEFVKKADKKLSGWGLFGNKHEDAAELFEKASNCYKLAKSWDKAGETYLKLADCYVKVDS

KHEAANAFAEAGHAYKKTSSKEAVSCLEQSVNSFLEIGRLNMGARYCKEIAEIFETDQNLDNAIVWHEKAGDLFQSEDVTTSA

NQCRQKVAQYAAQLEQYPKAIEIYEDIARQSLNNTL

Query	Observed	Mr(expt)	Mr(calc)	ppm	Miss	Score	Expect	Rank	Unique	Peptide
7	1192.6579	1191.6506	1191.6135	31.1	0	61	4e-005	1	U	K.AIEIYEDIAR.Q
10	1297.6861	1296.6788	1296.6350	33.8	1	48	0.00072	1	U	K.SWDKAGETYLK.L
19	1586.7876	1585.7803	1585.7273	33.4	0	61	3.7e-005	1	U	K.HEAANAFAEAGHAYK.K
25	2038.0481	2037.0409	2036.9837	28.1	0	37	0.0073	1	U	K.EAVSCLEQSVNSFLEIGR.L

Database 2

Match to: Unigene43231_SALfmcTARAAPEI-3 Mass: 16015 Score: 378 Matches: 4(4) Sequences: 4(4)

gi| 38564727 putative alpha-soluble NSF attachment protein [*Helianthus annuus*]

Matched peptides shown in **bold red**.

QNLDNAIAWHEKAADLFQSEDVTTSANQCRQKVAQYAAELGQYPKAIEIYEDIARQSLNNTLLKYGVKGHLLNAGLCHLCKG

DVVSITNALEKYQDLDPTFAGTREYRLLSDLAAAIDEQDVGKFTEVVKEFDSMTPLDAWK

Query	Observed	Mr(expt)	Mr(calc)	ppm	Miss	Score	Expect	Rank	Unique	Peptide
7	1192.6579	1191.6506	1191.6135	31.1	0	61	2e-005	1	U	K.AIEIYEDIAR.Q
13	1383.6924	1382.6851	1382.6467	27.8	0	81	2e-007	1	U	K.YQDLDPTFAGTR.E
14	1437.7682	1436.7609	1436.7299	21.5	0	83	1.1e-007	1	U	K.VAQYAAELGQYPK.A
22	2012.9510	2011.9437	2011.8905	26.4	0	153	8.7e-015	1	U	K.AADLFQSEDVTTSANQCR.Q

2. Unigene43515_SALfmcTARAAPEI-3 Mass: 13932 Score: 145 Matches: 3(3) Sequences: 3(3)

gi| 38564727 putative alpha-soluble NSF attachment protein [*Helianthus annuus*]

Query	Observed	Mr(expt)	Mr(calc)	ppm	Miss	Score	Expect	Rank	Unique	Peptide
10	1297.6861	1296.6788	1296.6350	33.8	1	48	0.00035	1	U	K.SWDKAGETYLK.L
19	1586.7876	1585.7803	1585.7273	33.4	0	61	1.8e-005	1	U	K.HEAANAFAEAGHAYK.K
25	2038.0481	2037.0409	2036.9837	28.1	0	37	0.0033	1	U	K.EAVSCLEQSVNSFLEIGR.L

Spot 2181

NCBInr protein database

Match to: gi|1272406 Mass: 15793 Score: 54 Matches: 1(1) Sequences: 1(1)

immunophilin [*Arabidopsis thaliana*]

Matched peptides shown in **bold red**.

1 MKAVGFLLLL TILTLAYAKK SGDVTELQIG VKYKPQKCDL QAHKGDKIKV

51 HYRGKLTDGT VFDSSFERGD PIEFELGTGQ VIPGWDQGLL GACVGEKRKL

101 KIPSKLGYGD NGSPPKIPGG ATLIFDTELV AVNGEPSSEA KSKNEL

Query	Observed	Mr(expt)	Mr(calc)	ppm	Miss	Score	Expect	Rank	Unique	Peptide
10	1658.7175	1657.7103	1657.7948	-50.98	1	54	0.007	1	U	R.GKLTDGTVFDSSFER.G

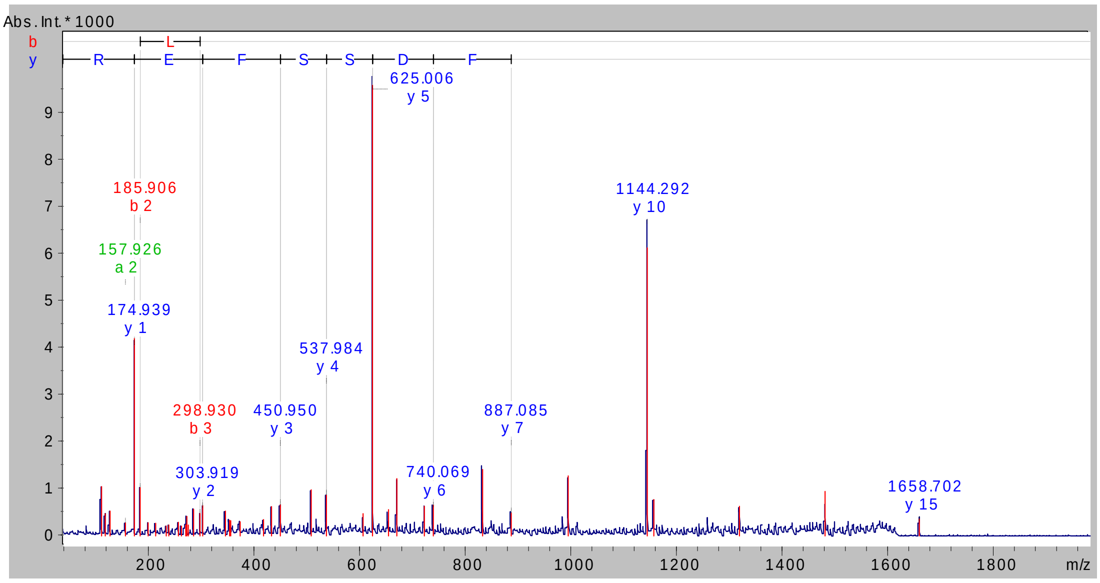

Proteins matching the same set of peptides:

gi|1272408 Mass: 17790 Score: 54 Matches: 1(1) Sequences: 1(1)

immunophilin [*Arabidopsis thaliana*]

gi|15239019 Mass: 17761 Score: 54 Matches: 1(1) Sequences: 1(1)

peptidyl-prolyl cis-trans isomerase FKBP15-2 [*Arabidopsis thaliana*]

gi|18404471 Mass: 16459 Score: 54 Matches: 1(1) Sequences: 1(1)

peptidyl-prolyl cis-trans isomerase FKBP15-1 [*Arabidopsis thaliana*]

gi|23396588 Mass: 16328 Score: 54 Matches: 1(1) Sequences: 1(1)

RecName: Full=FK506-binding protein 2; AltName: Full=15 kDa FKBP; AltName: Full=FKBP-15; AltName: Full=Peptidyl-prolyl cis-trans isomerase; Short=PPIase; AltName: Full=Rotamase; Flags: Precursor

gi|118489502 Mass: 16152 Score: 54 Matches: 1(1) Sequences: 1(1)

unknown [*Populus trichocarpa x Populus deltoides*]

gi|147828014 Mass: 15501 Score: 54 Matches: 1(1) Sequences: 1(1)

hypothetical protein VITISV_016372 [*Vitis vinifera*]

gi|217071518 Mass: 16552 Score: 54 Matches: 1(1) Sequences: 1(1)

unknown [*Medicago truncatula*]

gi|224065419 Mass: 16207 Score: 54 Matches: 1(1) Sequences: 1(1)

hypothetical protein POPTR_0002s24960g [*Populus trichocarpa*]

gi|225469328 Mass: 15817 Score: 54 Matches: 1(1) Sequences: 1(1)

PREDICTED: FK506-binding protein 2-1 [*Vitis vinifera*]

gi|255587693 Mass: 16019 Score: 54 Matches: 1(1) Sequences: 1(1)

fk506-binding protein, putative [*Ricinus communis*]

gi|297790286 Mass: 17691 Score: 54 Matches: 1(1) Sequences: 1(1)

FK506-binding protein 15 kD-2 [*Arabidopsis lyrata subsp. lyrata*]

gi|297831418 Mass: 16220 Score: 54 Matches: 1(1) Sequences: 1(1)

FK506-binding protein 15 kD-1 [*Arabidopsis lyrata subsp. lyrata*]

gi|357513317 Mass: 16568 Score: 54 Matches: 1(1) Sequences: 1(1)

FK506-binding protein [*Medicago truncatula*]

gi|388504402 Mass: 16852 Score: 54 Matches: 1(1) Sequences: 1(1)

unknown [*Lotus japonicus*]

gi|449463206 Mass: 16117 Score: 54 Matches: 1(1) Sequences: 1(1)

PREDICTED: peptidyl-prolyl cis-trans isomerase FKBP15-2-like [*Cucumis sativus*]

gi|470107658 Mass: 15953 Score: 54 Matches: 1(1) Sequences: 1(1)

PREDICTED: peptidyl-prolyl cis-trans isomerase FKBP15-2-like [*Fragaria vesca subsp. vesca*]

gi|502155917 Mass: 15984 Score: 54 Matches: 1(1) Sequences: 1(1)

PREDICTED: FK506-binding protein 2-like [*Cicer arietinum*]

gi|527184863 Mass: 15647 Score: 54 Matches: 1(1) Sequences: 1(1)

hypothetical protein M569_16257, partial [*Genlisea aurea*]

gi|565430598 Mass: 17937 Score: 54 Matches: 1(1) Sequences: 1(1)

hypothetical protein CARUB_v10027265mg [*Capsella rubella*]

gi|565482188 Mass: 16323 Score: 54 Matches: 1(1) Sequences: 1(1)

hypothetical protein CARUB_v10014832mg [*Capsella rubella*]

gi|567140760 Mass: 19136 Score: 54 Matches: 1(1) Sequences: 1(1)

hypothetical protein EUTSA_v10005016mg [*Eutrema salsugineum*]

gi|567158115 Mass: 20302 Score: 54 Matches: 1(1) Sequences: 1(1)

hypothetical protein EUTSA_v10002679mg [*Eutrema salsugineum*]

gi|567889448 Mass: 16411 Score: 54 Matches: 1(1) Sequences: 1(1)

hypothetical protein CICLE_v10032978mg [*Citrus clementina*]

gi|573958393 Mass: 15784 Score: 54 Matches: 1(1) Sequences: 1(1)

PREDICTED: peptidyl-prolyl cis-trans isomerase FKBP15-1-like [*Oryza brachyantha*]

gi|590713454 Mass: 16360 Score: 54 Matches: 1(1) Sequences: 1(1)

FK506- and rapamycin-binding protein 15 kD-2 [*Theobroma cacao*]

gi|595793507 Mass: 16496 Score: 54 Matches: 1(1) Sequences: 1(1)

hypothetical protein PRUPE_ppa012894mg [*Prunus persica*]

gi|351720801 Mass: 16874 Score: 53 Matches: 1(1) Sequences: 1(1)

uncharacterized protein LOC100499966 precursor [*Glycine max*]

gi|351722867 Mass: 15994 Score: 53 Matches: 1(1) Sequences: 1(1)

uncharacterized protein LOC100305479 precursor [*Glycine max*]

Spot 367

Database 1

Match to: Unigene26380_SALfmcTARAAPEI-3 Mass: 61038 Score: 34 Matches: 1(1) Sequences: 1(1)

gi| 357148034 PREDICTED: activator of 90 kDa heat shock protein ATPase homolog 1-like [*Brachypodium distachyon*]

Matched peptides shown in bold red.

NWPDGVFSTVKLTFEEPESGITVVKLVQTDVPEEDSFGNETVVENTERGWR**DLIFQR**IRAIFGFGL

Query	Observed	Mr(expt)	Mr(calc)	ppm	Miss	Score	Expect	Rank	Unique	Peptide
1	791.4416	790.4343	790.4371	-3.52	1	34	0.017	1	U	R.DLLMKR.R + Oxidation (M)

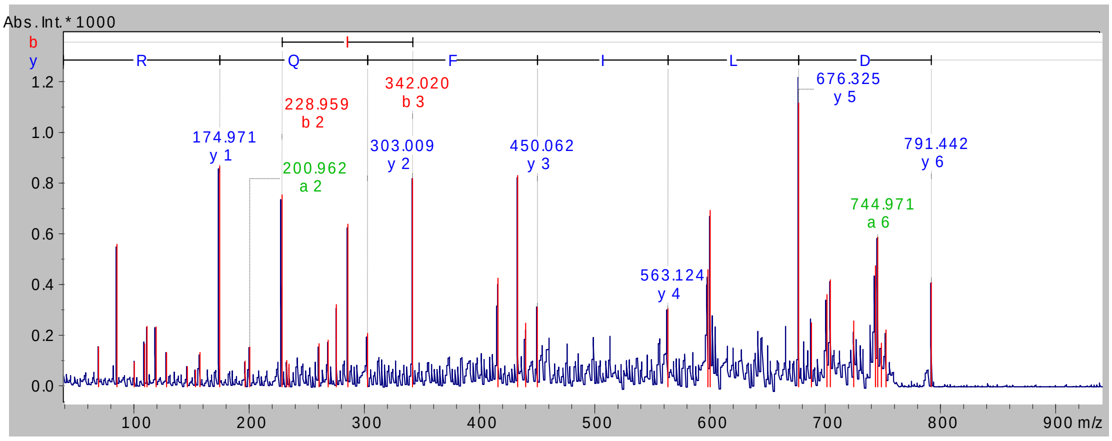

Spot 1984

Database 1

Match to: Unigene45684_SeCKS transcribed RNA sequence Mass: 22675 Score: 44 Matches: 1(1) Sequences: 1(1)

gi|587920345 Nascent polypeptide-associated complex subunit alpha-like protein [*Morus notabilis*]

Matched peptides shown in bold red.

GFCEYRPYLSRCLSSTVTSTAMTAQTQEELLAAHLDQQKIDDDEPIVEDDEDDDDDDDDDDKDDDELEGGQDGDASGRSKQS

RSEKKSRKAMLK**LGMKPISGVSRVTVK**KSKNILFVISKPDVFKSPTSDTYVIFGEAKIEDLSSQLQTQAAEQFKAPDLTNVISKP

ESSAMAQDDEDVDETGVEPKDIELVMTQAGVSRGKAVKA

Query	Observed	Mr(expt)	Mr(calc)	ppm	Miss	Score	Expect	Rank	Unique	Peptide
7	1571.9033	1570.8961	1570.9229	-17.07	1	44	0.001	1	U	K.LGMKPISGVSRVTVK.K

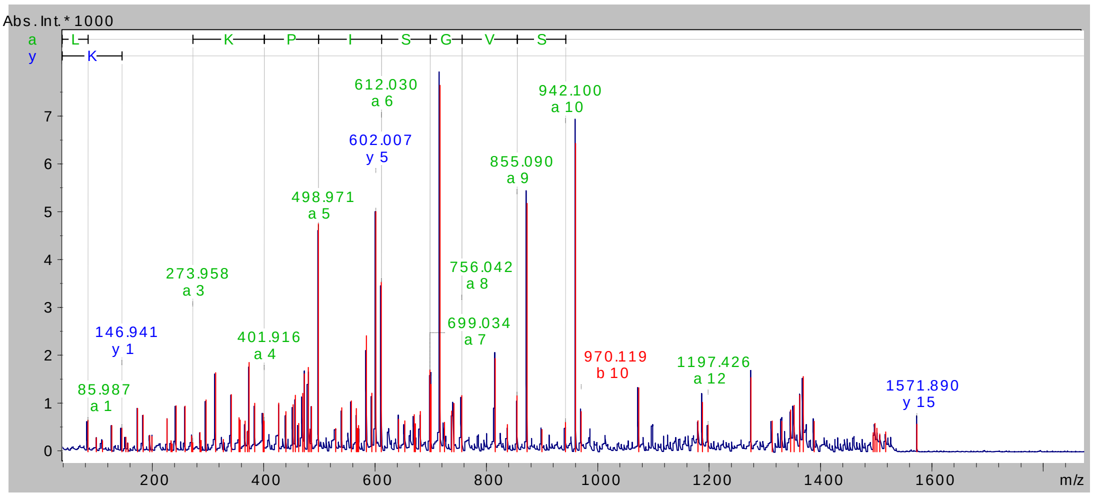

Proteins matching the same set of peptides:

Unigene47153_Se200S transcribed RNA sequence Mass: 24417 Score: 44 Matches: 1(1) Sequences: 1(1)

gi|672183023 PREDICTED: nascent polypeptide-associated complex subunit alpha-like protein 1 [*Phoenix dactylifera*]

Spot 621

Database 1

Match to: Unigene 12223_SeCKS transcribed RNA sequence Mass: 26947 Score: 182 Matches: 4(2) Sequences: 4(2)

gi| 225459587 PREDICTED: protein disulfide-isomerase [*Vitis vinifera*]

Matched peptides shown in bold red.

FFNSPNAKALLFMNFTIDFDSYKSKYEQAAGEYKSEGISFLLGDLDASQGALQYFGLKEEQVPVLIIQTNDGKKYVKDKVEPD

QIAAWIKEYNEGKVPAYRKSEPIPESNNEPVKVIVADTIEDIVFKSAKNVLLEFYAPWCGHCQKLAPILDEVAVSFENDPDVVIA

KFDATANDLPGDSFDVKGYPTLYFRSASGNIISYDGDRTKEDMIEFIQKNRDPAIQQTTTKDEQPSAKDEL

Query	Observed	Mr(expt)	Mr(calc)	ppm	Miss	Score	Expect	Rank	Unique	Peptide
2	924.4922	923.4849	923.4501	37.7	0	28	0.087	1	U	-.FFNSPNAK.A
3	1016.5625	1015.5552	1015.5127	41.9	0	24	0.33	1	U	K.GYPTLYFR.S
7	1325.6816	1324.6743	1324.6411	25.0	1	40	0.0054	1	U	K.EYNEGKVPAYR.K
10	1711.8554	1710.8481	1710.7737	43.5	0	90	4.9e-008	1	U	K.FDATANDLPGDSFDVK.G

Database 2

Match to: Unigene40965_SALfmcTARAAPEI-3 Mass: 14359 Score: 154 Matches: 3(2) Sequences: 3(2)

Matched peptides shown in **bold red**.

gi| 304557199 protein disulfide isomerase [*Citrus limon*]

NDGKKYVKDKVEPDQIAAWIKEYNEGKVPAYRKSEPIPESNNEPVKVIVADTIEDIVFKSAKNVLLEFYAPWCGHCQKLAPILD

EVAVSFENDPDVVIAKFDATANDLPGDSFDVKGYPTLYFRSASG

Query	Observed	Mr(expt)	Mr(calc)	ppm	Miss	Score	Expect	Rank	Unique	Peptide
3	1016.5625	1015.5552	1015.5127	41.9	0	24	0.14	1	U	K.GYPTLYFR.S
7	1325.6816	1324.6743	1324.6411	25.0	1	40	0.0024	1	U	K.EYNEGKVPAYR.K
10	1711.8554	1710.8481	1710.7737	43.5	0	90	2.1e-008	1	U	K.FDATANDLPGDSFDVK.G

Spot 627

Database 1

Match to: Unigene 12223_SeCKS transcribed RNA sequence Mass: 26947 Score: 80 Matches: 3(1) Sequences: 3(1)

gi| 225459587 PREDICTED: protein disulfide-isomerase [*Vitis vinifera*]

Matched peptides shown in **bold red**.

FFNSPNAKALLFMNFTIDFDSYKSKYEQAAGEYKSEGISFLLGDLDASQGALQYFGLKEEQVPVLIIQTNDGKKYVKDKVEPD

QIAAWIKEYNEGKVPAYRKSEPIPESNNEPVKVIVADTIEDIVFKSAKNVLLEFYAPWCGHCQKLAPILDEVAVSFENDPDVVIA

KFDATANDLPGDSFDVKGYPTLYFRSASGNIISYDGDRTKEDMIEFIQKNRDPAIQQTTTKDEQPSAKDEL

Query	Observed	Mr(expt)	Mr(calc)	ppm	Miss	Score	Expect	Rank	Unique	Peptide
2	924.4753	923.4680	923.4501	19.4	0	12	4	1	U	-.FFNSPNAK.A
3	1016.5374	1015.5301	1015.5127	17.2	0	21	0.59	1	U	K.GYPTLYFR.S
12	1711.7943	1710.7870	1710.7737	7.76	0	48	0.00065	1	U	K.FDATANDLPGDSFDVK.G

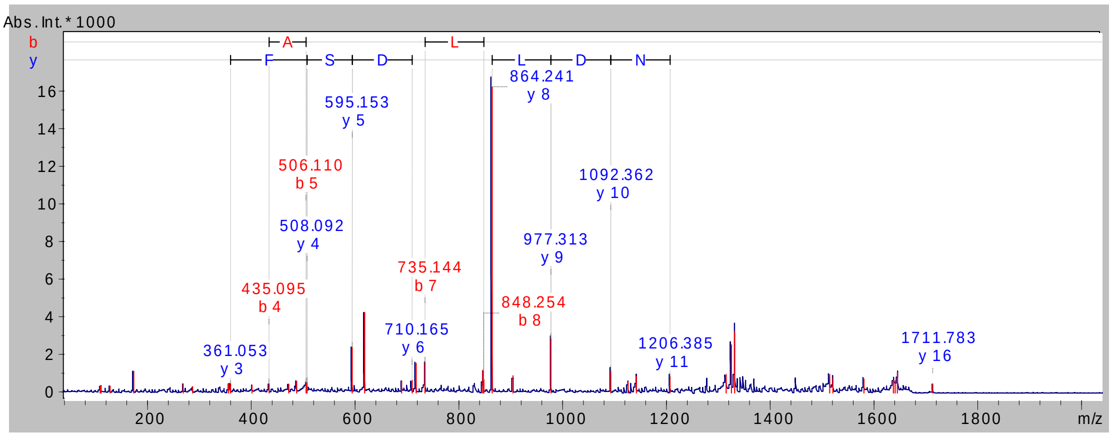

Database 2

Match to: Unigene40965_SALfmcTARAAPEI-3 Mass: 14359 Score: 69 Matches: 2(1) Sequences: 2(1)

gi| 304557199 protein disulfide isomerase [*Citrus limon*]

Matched peptides shown in bold red.

NDGKKYVKDKVEPDQIAAWIKEYNEGKVPAYRKSEPIPESNNEPVKVIVADTIEDIVFKSAKNVLLEFYAPWCGHCQKLAPILD
EVAVSFENDPDVVIAK**FDATANDLPGDSFDVKGYPTLYFR**SASG

Query	Observed	Mr(expt)	Mr(calc)	ppm	Miss	Score	Expect	Rank	Unique	Peptide
3	1016.5374	1015.5301	1015.5127	17.2	0	21	0.28	1	U	K.GYPTLYFR.S
12	1711.7943	1710.7870	1710.7737	7.76	0	48	0.00035	1	U	K.FDATANDLPGDSFDVK.G

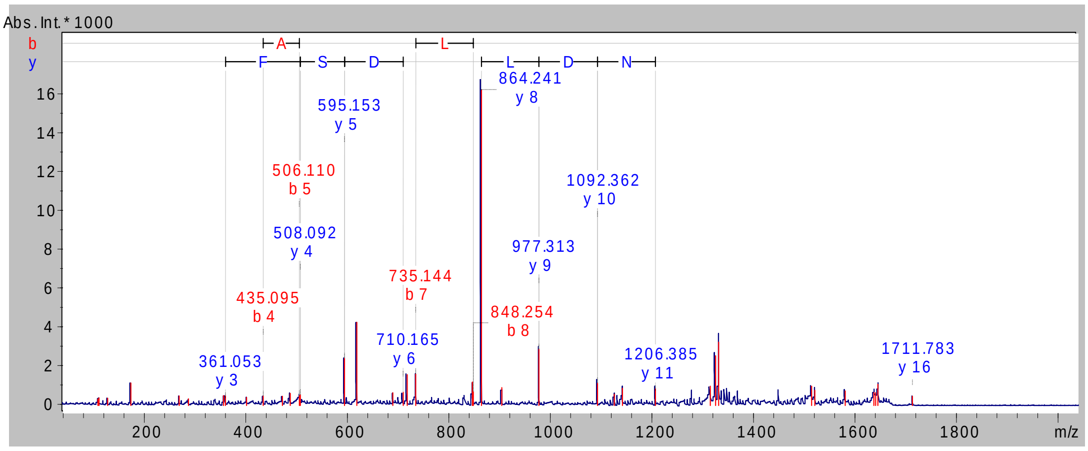

Spot 628

Database 1

Match to: Unigene 12223_SeCKS transcribed RNA sequence Mass: 26947 Score: 97 Matches: 3(1) Sequences: 3(1)

gi| 225459587 PREDICTED: protein disulfide-isomerase [*Vitis vinifera*]

Matched peptides shown in bold red.

FFNSPNAKALLFMNFTIDFDSYKSKYEQAAGEYKSEGISFLLGDLDASQGALQYFGLKEEQVPVLIIQTNDGKKYVK**DKVEPD
QIAAWIK**EYNEGKVPAYRKSEPIPESNNEPVKVIVADTIEDIVFKSAKNVLLEFYAPWCGHCQKLAPILDEVAVSFENDPDVVIA
KFDATANDLPGDSFDVK**GYPTLYFR**SASGNIISYDGDRTKEDMIEFIQKNRDPAIQQTTTKDEQPSAKDEL

Query	Observed	Mr(expt)	Mr(calc)	ppm	Miss	Score	Expect	Rank	Unique	Peptide
3	1016.5461	1015.5389	1015.5127	25.8	0	26	0.18	1	U	K.GYPTLYFR.S
12	1512.8426	1511.8353	1511.7984	24.4	1	25	0.12	1	U	K.DKVEPDQIAAWIK.E
16	1711.8282	1710.8209	1710.7737	27.6	0	46	0.0012	1	U	K.FDATANDLPGDSFDVK.G

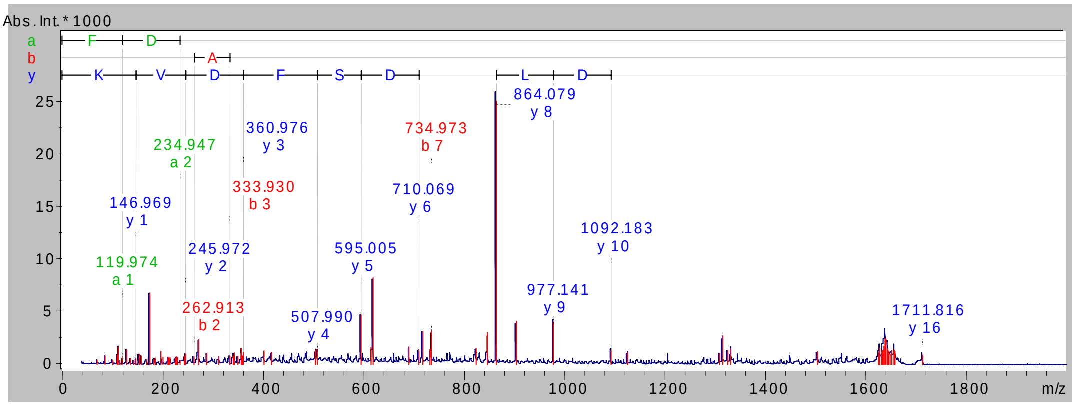

Database 2

Match to: Unigene40965_SALfmcTARAAPEI-3 Mass: 14359 Score: 97 Matches: 3(1) Sequences: 3(1)

gi| 304557199 protein disulfide isomerase [*Citrus limon*]

Matched peptides shown in bold red.

NDGKKYVKDKVEPDQIAAWIKEYNEGKVPAYRKSEPIPESNNEPVKVIVADTIEDIVFKSAKNVLLEFYAPWCGHCQKLAPILD

EVAVSFENDPDVVIAKFDATANDLPGDSFDVKGYPTLYFRSASG

Query	Observed	Mr(expt)	Mr(calc)	ppm	Miss	Score	Expect	Rank	Unique	Peptide
3	1016.5461	1015.5389	1015.5127	25.8	0	26	0.083	1	U	K.GYPTLYFR.S
12	1512.8426	1511.8353	1511.7984	24.4	1	25	0.067	1	U	K.DKVEPDQIAAWIK.E
16	1711.8282	1710.8209	1710.7737	27.6	0	46	0.00054	1	U	K.FDATANDLPGDSFDVK.G

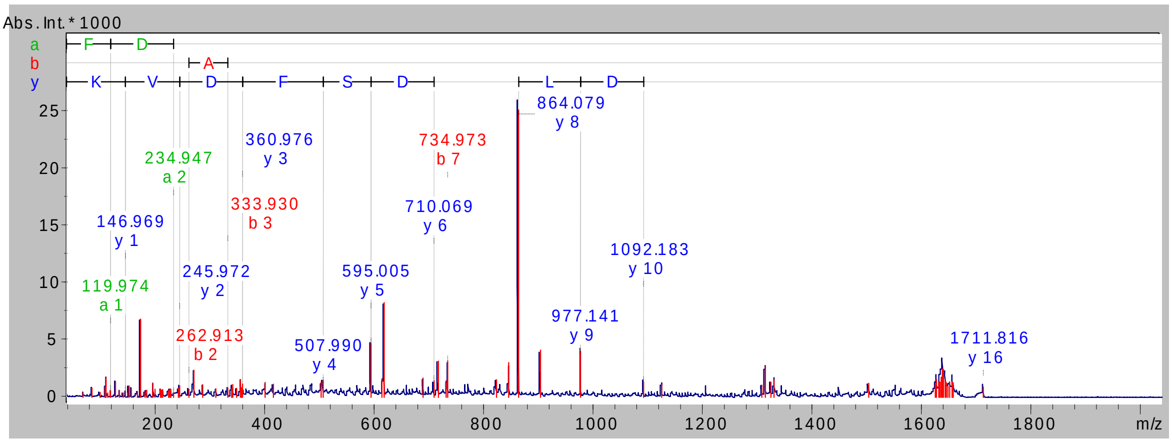

Spot 1066

NCBInr protein database

Match to: gi|322422113 Mass: 41941 Score: 209 Matches: 4(3) Sequences: 4(3)

actin 1 [*Celosia argentea*]

Matched peptides shown in bold red

1 MADAEEIQPL VCDNGTGMVK AGFAGDDAPR AVFPSIVGRP RHTGVMVGMG

51 QKDAYVGDEA QSKRGILTLK YPIEHGIVSN WDDMEKIWHH TFYNELRVAP

101 EEHPVLLTEA PLNPKANREK MTQIMFETFN VPAMYVAIQA VLSLYASGRT

151 TGIVLDSGDG VSHTVPIYEG YALPHAILRL DLAGRDLTDY LMKILTERGY

201 MFTTSAEREI VRDIKEKLAY VALDFEQESE TAKSSSAIEK NYELPDGQVI

251 TIGAERFRCP EVLFQPSLIG MEAAGIHETT YNSIMKCDVD IRKDLYGNIV

301 LSGGTTMFPG IADRMSKEIT ALAPSSMKIK VVAPPERKYS VWIGGSILAS

351 LSTFQQMWIS KGEYDESGPS IVHRKCF

Query	Observed	Mr(expt)	Mr(calc)	ppm	Miss	Score	Expect	Rank	Unique	Peptide
4	976.4460	975.4387	975.4410	-2.33	0	21	27	2		K.AGFAGDDAPR.A
11	1445.6539	1444.6467	1444.6583	-8.02	0	67	0.00054	1	U	K.GEYDESGPSIVHR.K
19	1813.8543	1812.8470	1812.8781	-17.18	0	52	0.016	1	U	K.LAYVALDFEQESETAK.S
22	1954.0285	1953.0212	1953.0571	-18.36	0	70	0.00024	1	U	

R.VAPEEHPVLLTEAPLNPK.A

Database 1

Match to: Unigene44960_SeCKS transcribed RNA sequence Mass: 39806 Score: 151 Matches: 4(2) Sequences: 4(2)

gi| 322422113 actin 1 [*Celosia argentea*]

IKMADAEEIQPLVCDNGTGMVKAGFAGDDAPRAVFPSIVGRPRHTGVMVGMGQKDAYVGDEAQSKRGILTLKYPIEHGIVSN

WDDMEKIWHHTFYNELRVAPEEHPVLLTEAPLNPKANREKMTQIMFETFNVPAMYVAIQAVLSLYASGRTTGIVLDSGDGVSH

TVPIYEGYALPHAILRLDLAGRDLTDYLMKILTERGYMFTTSAEREIVRDIKEKLAYVALDFEQESETAKSSSAIEKNYELPDGQ

VITIGAERFRCPEVLFQPSLIGMEAAGIHETTYNSIMKCDVDIRKDLYGNIVLSGGTTMFPGIADRMSKEITALAPSSMKIKVVAP

PERKYSVWIGGSILASLSTFQQM

Query	Observed	Mr(expt)	Mr(calc)	ppm	Miss	Score	Expect	Rank	Unique	Peptide
4	976.4460	975.4387	975.4410	-2.33	0	21	0.36	1	U	K.AGFAGDDAPR.A
8	1178.5246	1177.5173	1177.5074	8.46	0	9	4.4	2	U	R.GYMFTTSAER.E + Oxidation (M)
19	1813.8543	1812.8470	1812.8781	-17.18	0	52	0.00025	1	U	K.LAYVALDFEQESETAK.S
22	1954.0285	1953.0212	1953.0571	-18.36	0	70	4.5e-006	1	U	R.VAPEEHPVLLTEAPLNPK.A

Proteins matching the same set of peptides:

Unigene47152_Se200S transcribed RNA sequence Mass: 41001 Score: 151 Matches: 4(2) Sequences: 4(2)

gi|322422113 actin 1 [*Celosia argentea*]

Database 2

Match to: Unigene54790_SALfmcTARAAPEI-3 Mass: 33967 Score: 145 Matches: 3(2) Sequences: 3(2)

gi| 322422113 actin 1 [*Celosia argentea*]

GIVSNWDDMEKIWHHTFYNELRVAPEEHPVLLTEAPLNPKANREKMTQIMFETFNVPAMYVAIQAVLSLYASGRTTGIVLDSGD

GVSHTVPIYEGYALPHAILRLDLAGRDLTDYLMKILTERGYMFTTSAEREIVRDIKEKLAYVALDYEQESETAKSSSVIEKNYEL

PDGQVITIGAERFRCPEVLFQPSLVGMESAGIHETTYNSIMKCDVDIRKDLYGNIVLSGGSTMFPGIADRMSKEITALAPSSMKIK

VVAPPERKYSVWIGGSILASLSTFQQMWISKGEYDESGPSIVHRKCF

Query	Observed	Mr(expt)	Mr(calc)	ppm	Miss	Score	Expect	Rank	Unique	Peptide
8	1178.5246	1177.5173	1177.5074	8.46	0	9	3.1	2	U	R.GYMFTTSAER.E + Oxidation (M)
11	1445.6539	1444.6467	1444.6583	-8.02	0	67	4.7e-006	1	U	K.GEYDESGPSIVHR.K
22	1954.0285	1953.0212	1953.0571	-18.36	0	70	2e-006	1	U	R.VAPEEHPVLLTEAPLNPK.A

Spot 1071

NCBInr protein database

Match to: gi|322422113 Mass: 41941 Score: 736 Matches: 13(6) Sequences: 12(6)

actin 1 [*Celosia argentea*]

Matched peptides shown in bold red.

1 MADAEEIQPLVCDNGTGMVKAGFAGDDAPRAVFPSIVGRPRHTGVMVGMG

51 QKDAYVGDEAQSKRGILTLKYPIEHGIVSNWDDMEKIWHHTFYNELRVAP

101 EEHPVLLTEAPLNPKANREKMTQIMFETFN VPAMYVAIQAVLSLYASGRT

151 TGIVLDSGDGVSHTVPIYEGYALPHAILRLDLAGRDLTDYLMKILTERGY

201 MFTTSAEREIVRDIKEKLAYVALDFEQESETAKSSSAIEK NYELPDGQVI

251 TIGAERFRCPEVLFQPSLIGMEAAGIHETTYNSIMKCDVDIRKDLYGNIV

301 LSGGTTMFPGIADRMSKEITALAPSSMKIKVVAPPERKYSVWIGGSILAS

351 LSTFQQMWISKGEYDESGPSIVHRKCF

Query	Observed	Mr(expt)	Mr(calc)	ppm	Miss	Score	Expect	Rank	Unique	Peptide
1	767.4515	766.4442	766.4337	13.6	0	24	9.7	9		K.VVAPPER.K
3	945.5678	944.5606	944.5444	17.2	0	15	1.2e+02	6		R.AVFPSIVGR.P
4	976.4596	975.4524	975.4410	11.6	0	39	0.41	1		K.AGFAGDDAPR.A
6	1162.5314	1161.5241	1161.5125	10.0	0	41	0.21	1		R.GYMFTTSAER.E
7	1178.5322	1177.5249	1177.5074	14.9	0	(29)	3.7	1		R.GYMFTTSAER.E + Oxidation (M)
8	1198.7164	1197.7091	1197.6982	9.09	1	51	0.017	1		R.AVFPSIVGRPR.H
9	1338.6415	1337.6343	1337.6211	9.81	1	20	30	1		K.DAYVGDEAQSKR.G

10 1445.6800 1444.6727 1444.6583 10.00 0 88 4e-06 1 U K.GEYDESGPSIVHR.K

19 1813.8898 1812.8826 1812.8781 2.45 0 107 4.9e-08 1 U K.LAYVALDFEQESETAK.S

24 1954.0721 1953.0649 1953.0571 3.98 0 95 6.3e-07 1 R.VAPEEHPVLLTEAPLNPK.A

31 2341.1868 2340.1795 2340.1784 0.47 1 22 11 1

R.KDLYGNIVLSGGTTMFPGIADR.M + Oxidation (M)

33 2477.2565 2476.2492 2476.2445 1.89 1 117 2.8e-09 1 U

K.SSSAIEKNYELPDGQVITIGAER.F

35 3151.7245 3150.7172 3150.6350 26.1 0 118 1.5e-09 1

R.TTGIVLDSGDGVSHTVPIYEGYALPHAILR.L

2. gi|1531672 Mass: 41945 Score: 485 Matches: 9(4) Sequences: 9(4)

actin [*Striga asiatica*]

Query Observed Mr(expt) Mr(calc) ppm Miss Score Expect Rank Unique Peptide

1 767.4515 766.4442 766.4337 13.6 0 24 9.7 9 K.VVAPPER.K

3 945.5678 944.5606 944.5444 17.2 0 15 1.2e+02 6 R.AVFPSIVGR.P

4 976.4596 975.4524 975.4410 11.6 0 39 0.41 1 K.AGFAGDDAPR.A

8 1198.7164 1197.7091 1197.6982 9.09 1 51 0.017 1 R.AVFPSIVGRPR.H

9 1338.6415 1337.6343 1337.6211 9.81 1 20 30 1 K.DAYVGDEAQSKR.G

24 1954.0721 1953.0649 1953.0571 3.98 0 95 6.3e-07 1 R.VAPEEHPVLLTEAPLNPK.A

31 2341.1868 2340.1795 2340.1784 0.47 1 22 11 1

R.KDLYGNIVLSGGTTMFPGIADR.M + Oxidation (M)

33 2477.2565 2476.2492 2476.2445 1.89 1 103 7.6e-08 2

K.TSSAVEKNYELPDGQVITIGAER.F

35 3151.7245 3150.7172 3150.6350 26.1 0 118 1.5e-09 1

R.TTGIVLDSGDGVSHTVPIYEGYALPHAILR.L

3. gi|388506274 Mass: 42004 Score: 408 Matches: 9(3) Sequences: 9(3)

unknown [*Medicago truncatula*]

Query Observed Mr(expt) Mr(calc) ppm Miss Score Expect Rank Unique Peptide

1 767.4515 766.4442 766.4337 13.6 0 24 9.7 9 K.VVAPPER.K

3 945.5678 944.5606 944.5444 17.2 0 15 1.2e+02 6 R.AVFPSIVGR.P

4 976.4596 975.4524 975.4410 11.6 0 39 0.41 1 K.AGFAGDDAPR.A

8 1198.7164 1197.7091 1197.6982 9.09 1 51 0.017 1 R.AVFPSIVGRPR.H

9 1338.6415 1337.6343 1337.6211 9.81 1 20 30 1 K.DAYVGDEAQSKR.G

11 1473.7184 1472.7111 1472.6895 14.6 0 25 8.7 1 K.AEYEESGPSIVHR.K

24 1954.0721 1953.0649 1953.0571 3.98 0 95 6.3e-07 1 R.VAPEEHPVLLTEAPLNPK.A

31 2341.1868 2340.1795 2340.1784 0.47 1 22 11 1 R.KDLYGNIVLSGGTTMFPGIADR.M +

Oxidation (M)

35 3151.7245 3150.7172 3150.6350 26.1 0 118 1.5e-09 1

R.TTGIVLDSGDGVSHTVPIYEGYALPHAILR.L

4. gi|590636982 Mass: 41913 Score: 406 Matches: 8(3) Sequences: 8(3)

Actin-11 [*Theobroma cacao*]

Query Observed Mr(expt) Mr(calc) ppm Miss Score Expect Rank Unique Peptide

1 767.4515 766.4442 766.4337 13.6 0 24 9.7 9 K.VVAPPER.K

3 945.5678 944.5606 944.5444 17.2 0 15 1.2e+02 6 R.AVFPSIVGR.P

4 976.4596 975.4524 975.4410 11.6 0 39 0.41 1 K.AGFAGDDAPR.A

8	1198.7164	1197.7091	1197.6982	9.09	1	51	0.017	1		R.AVFPSIVGRPR.H
9	1338.6415	1337.6343	1337.6211	9.81	1	20	30	1		K.DAYVGDEAQSKR.G
24	1954.0721	1953.0649	1953.0571	3.98	0	95	6.3e-07	1		R.VAPEEHPVLLTEAPLNPK.A
26	2105.9504	2104.9432	2104.9405	1.25	0	45	0.057	1	U	M.AENEDIQPLVCDNGTGMVK.A + Oxidation (M)
35	3151.7245	3150.7172	3150.6350	26.1	0	118	1.5e-09	1		R.TTGIVLDSGDGVSHTVPIYEGYALPHAILR.L

5. gi|345546675 Mass: 19115 Score: 373 Matches: 7(3) Sequences: 7(3)

actin [*Lolium perenne*]

Query	Observed	Mr(expt)	Mr(calc)	ppm	Miss	Score	Expect	Rank	Unique	Peptide
3	945.5678	944.5606	944.5444	17.2	0	15	1.2e+02	6		R.AVFPSIVGR.P
4	976.4596	975.4524	975.4410	11.6	0	39	0.41	1		K.AGFAGDDAPR.A
8	1198.7164	1197.7091	1197.6982	9.09	1	51	0.017	1		R.AVFPSIVGRPR.H
9	1338.6415	1337.6343	1337.6211	9.81	1	20	30	1		K.DAYVGDEAQSKR.G
24	1954.0721	1953.0649	1953.0571	3.98	0	95	6.3e-07	1	U	R.VAPEEHPVLLTEAPLNPK.X
25	1992.0210	1991.0137	1991.0476	-17.02	0	35	0.6	1	U	R.VAPEEHPHLLTEAPLNPK.X
35	3151.7245	3150.7172	3150.6350	26.1	0	118	1.5e-09	1		R.TTGIVLDSGDGVSHTVPIYEGYALPHAILR.L

6. gi|449438155 Mass: 41923 Score: 364 Matches: 8(3) Sequences: 8(3)

PREDICTED: actin-like [*Cucumis sativus*]

Query	Observed	Mr(expt)	Mr(calc)	ppm	Miss	Score	Expect	Rank	Unique	Peptide
1	767.4515	766.4442	766.4337	13.6	0	24	9.7	9		K.VVAPPER.K
3	945.5678	944.5606	944.5444	17.2	0	15	1.2e+02	6		R.AVFPSIVGR.P
4	976.4596	975.4524	975.4410	11.6	0	39	0.41	1		K.AGFAGDDAPR.A
8	1198.7164	1197.7091	1197.6982	9.09	1	51	0.017	1		R.AVFPSIVGRPR.H
9	1338.6415	1337.6343	1337.6211	9.81	1	20	30	1		K.DAYVGDEAQSKR.G
24	1954.0721	1953.0649	1953.0571	3.98	0	95	6.3e-07	1		R.VAPEEHPVLLTEAPLNPK.A
31	2341.1868	2340.1795	2340.1784	0.47	1	22	11	1		R.KDLYGNIVLSGGTTMFPGIADR.M + Oxidation (M)
35	3151.7245	3150.7172	3150.6350	26.1	0	98	1.4e-07	2	U	R.TTGIVLDSGDGVSHTVPIYEGFSLPHAILR.L

7. gi|3219771 Mass: 37234 Score: 352 Matches: 8(3) Sequences: 8(3)

RecName: Full=Actin-41

Query	Observed	Mr(expt)	Mr(calc)	ppm	Miss	Score	Expect	Rank	Unique	Peptide
1	767.4515	766.4442	766.4337	13.6	0	24	9.7	9		K.VVAPPER.K
3	945.5678	944.5606	944.5444	17.2	0	15	1.2e+02	6		R.AVFPSIVGR.P
4	976.4596	975.4524	975.4410	11.6	0	39	0.41	1		-.AGFAGDDAPR.A
8	1198.7164	1197.7091	1197.6982	9.09	1	51	0.017	1		R.AVFPSIVGRPR.H
9	1338.6415	1337.6343	1337.6211	9.81	1	20	30	1		K.DAYVGDEAQSKR.G
24	1954.0721	1953.0649	1953.0571	3.98	0	95	6.3e-07	1		R.VAPEEHPVLLTEAPLNPK.A
31	2341.1868	2340.1795	2340.1784	0.47	1	22	11	1		R.KDLYGNIVLSGGTTMFPGIADR.M + Oxidation (M)
35	3151.7245	3150.7172	3150.6350	26.1	0	87	2.1e-06	3	U	R.STGIVLDSGDGLSHTVPIYEGYALPHAILR.L

8. gi|527203529 Mass: 41989 Score: 298 Matches: 8(2) Sequences: 8(2)

actin [*Genlisea aurea*]

Query	Observed	Mr(expt)	Mr(calc)	ppm	Miss	Score	Expect	Rank	Unique	Peptide
1	767.4515	766.4442	766.4337	13.6	0	24	9.7	9		K.VVAPPER.K
3	945.5678	944.5606	944.5444	17.2	0	15	1.2e+02	6		R.AVFPSIVGR.P
4	976.4596	975.4524	975.4410	11.6	0	39	0.41	1		K.AGFAGDDAPR.A
8	1198.7164	1197.7091	1197.6982	9.09	1	51	0.017	1		R.AVFPSIVGRPR.H
9	1338.6415	1337.6343	1337.6211	9.81	1	20	30	1		K.DAYVGDEAQSKR.G
11	1473.7184	1472.7111	1472.6896	14.6	0	10	2.9e+02	8	U	K.AEYDETGPSIVHR.K
31	2341.1868	2340.1795	2340.1784	0.47	1	22	11	1		R.KDLYGNIVLSGGTTMFPGIADR.M + Oxidation (M)
35	3151.7245	3150.7172	3150.6350	26.1	0	118	1.5e-09	1		R.TTGIVLDSGDGVSHTVPIYEGYALPHAILR.L

9. gi|9408605 Mass: 41850 Score: 285 Matches: 5(2) Sequences: 5(2)

actin [*Magnolia denudata*]

Query	Observed	Mr(expt)	Mr(calc)	ppm	Miss	Score	Expect	Rank	Unique	Peptide
1	767.4515	766.4442	766.4337	13.6	0	24	9.7	9		K.VVAPPER.K
8	1198.7164	1197.7091	1197.6982	9.09	1	28	3.1	5	U	R.AVSPIFVGRPR.H
9	1338.6415	1337.6343	1337.6211	9.81	1	20	30	1		K.DAYVGDEAQSKR.G
24	1954.0721	1953.0649	1953.0571	3.98	0	95	6.3e-07	1		R.VAPEEHPVLLTEAPLNPK.A
35	3151.7245	3150.7172	3150.6350	26.1	0	118	1.5e-09	1		R.TTGIVLDSGDGVSHTVPIYEGYALPHAILR.L

10. gi|379054856 Mass: 14509 Score: 197 Matches: 4(2) Sequences: 4(2)

actin-like protein, partial [*Miscanthus sinensis*]

Query	Observed	Mr(expt)	Mr(calc)	ppm	Miss	Score	Expect	Rank	Unique	Peptide
3	945.5678	944.5606	944.5444	17.2	0	15	1.2e+02	6		R.AVFPSIVGR.P
4	976.4596	975.4524	975.4410	11.6	0	39	0.41	1		K.AGFAGDDAPR.A
8	1198.7164	1197.7091	1197.6982	9.09	1	51	0.017	1		R.AVFPSIVGRPR.H
24	1954.0721	1953.0649	1953.0571	3.98	0	92	1.3e-06	6	U	R.VAPEEHPVLLTEAPINPK.S

Proteins matching the same set of peptides:

gi|589098163 Mass: 41823 Score: 197 Matches: 4(2) Sequences: 4(2)

hypothetical protein TRIREDRAFT_44504 [*Trichoderma reesei QM6a*]

11. gi|383506517 Mass: 20026 Score: 168 Matches: 2(2) Sequences: 2(2)

actin 2, partial [*Nicotiana tabacum*]

Query	Observed	Mr(expt)	Mr(calc)	ppm	Miss	Score	Expect	Rank	Unique	Peptide
19	1813.8898	1812.8826	1812.9145	-17.62	0	49	0.03	2	U	K.LAYVALDYEQTLDTAK.S
35	3151.7245	3150.7172	3150.6350	26.1	0	118	1.5e-09	1		R.TTGIVLDSGDGVSHTVPIYEGYALPHAILR.L

12. gi|148909436 Mass: 42060 Score: 148 Matches: 5(1) Sequences: 5(1)

unknown [*Picea sitchensis*]

Query	Observed	Mr(expt)	Mr(calc)	ppm	Miss	Score	Expect	Rank	Unique	Peptide
1	767.4515	766.4442	766.4337	13.6	0	24	9.7	9		K.VVAPPER.K

3	945.5678	944.5606	944.5444	17.2	0	15	1.2e+02	6	U	R.AVFPSLVGR.P
4	976.4596	975.4524	975.4410	11.6	0	39	0.41	1		K.AGFAGDDAPR.A
8	1198.7164	1197.7091	1197.6982	9.09	1	51	0.017	1	U	R.AVFPSLVGRPR.H
9	1338.6415	1337.6343	1337.6211	9.81	1	20	30	1		K.DAYVGDEAQSKR.G

13. gi|323371358 Mass: 17809 Score: 144 Matches: 3(1) Sequences: 2(1)

ACTIN [*Plantago coronopus*]

Query	Observed	Mr(expt)	Mr(calc)	ppm	Miss	Score	Expect	Rank	Unique	Peptide
6	1162.5314	1161.5241	1161.5125	10.0	0	41	0.21	1		R.GYMFTTSAER.E
7	1178.5322	1177.5249	1177.5074	14.9	0	(29)	3.7	1		R.GYMFTTSAER.E + Oxidation (M)
33	2477.2565	2476.2492	2476.2445	1.89	1	103	7.6e-08	2		R.TSSAVEKNYELPDGQVITIGAER.F

Database 1

Match to: Unigene44960_SeCKS transcribed RNA sequenceMass: 39806 Score: 638 Matches: 12(8) Sequences: 11(7)

gi| 322422113 actin 1 [*Celosia argentea*]

Matched peptides shown in bold red.

IKMADAEEIQPLVCDNGTGMVKAGFAGDDAPRAVFPSIVGRPRHTGVMVGMGQKDAYVGDEAQSKRGILTLKYPIEHGIVSN
WDDMEKIWHHTFYNELRVAPEEHPVLLTEAPLNPKANREKMTQIMFETFNVPAMYVAIQAVLSLYASGRTTGIVLDSGDGVSH
TVPIYEGYALPHAILRLDLAGRDLTDYLMKILTERGYMFTTSAEREIVRDIKEKLAYVALDFEQESETAKSSSAIEKNYELPDGQ
VITIGAERFRCPEVLFQPSLIGMEAAGIHETTYNSIMKCDVDIRKDLYGNIVLSGGTTMFPGIADRMSKEITALAPSSMKIKVVAP
PERKYSVWIGGSILASLSTFQQM

Query	Observed	Mr(expt)	Mr(calc)	ppm	Miss	Score	Expect	Rank	Unique	Peptide
1	767.4515	766.4442	766.4337	13.6	0	24	0.13	1	U	K.VVAPPER.K
4	976.4596	975.4524	975.4410	11.6	0	39	0.0061	1	U	K.AGFAGDDAPR.A
6	1162.5314	1161.5241	1161.5125	10.0	0	41	0.0031	1	U	R.GYMFTTSAER.E
7	1178.5322	1177.5249	1177.5074	14.9	0	(29)	0.052	1	U	R.GYMFTTSAER.E + Oxidation (M)
8	1198.7164	1197.7091	1197.6982	9.09	0	51	0.00025	1	U	R.AVFPSIVGRPR.H
9	1338.6415	1337.6343	1337.6211	9.81	1	20	0.56	1	U	K.DAYVGDEAQSKR.G
13	1515.7561	1514.7489	1514.7419	4.62	0	4	24	5	U	K.IWHHTFYNELR.V
19	1813.8898	1812.8826	1812.8781	2.45	0	107	8.3e-010	1	U	K.LAYVALDFEQESETAK.S
24	1954.0721	1953.0649	1953.0571	3.98	0	95	9.6e-009	1	U	R.VAPEEHPVLLTEAPLNPK.A
31	2341.1868	2340.1795	2340.1784	0.47	1	22	0.25	1	U	R.KDLYGNIVLSGGTTMFPGIADR.M + Oxidation (M)
33	2477.2565	2476.2492	2476.2445	1.89	1	117	5.7e-011	1	U	K.SSSAIEKNYELPDGQVITIGAER.F
35	3151.7245	3150.7172	3150.6350	26.1	0	118	2.3e-011	1	U	R.TTGIVLDSGDGVSHTVPIYEGYALPHAILR.L

Proteins matching the same set of peptides:

Unigene47152_Se200S transcribed RNA sequence Mass: 41001 Score: 638 Matches: 12(8) Sequences: 11(7)

gi|322422113 actin 1 [*Celosia argentea*]

Database 2

Match to: Unigene54790_SALfmcTARAAPEI-3 Mass: 33967 Score: 370 Matches: 7(5) Sequences: 6(4)

gi| 322422113 actin 1 [*Celosia argentea*]

Matched peptides shown in bold red.

GIVSNWDDMEKIWHHTFYNELRVAPEEHPVLLTEAPLNPKANREKMTQIMFETFNVPAMYVAIQAVLSLYASGRTTGIVLDSGD

GVSHTVPIYEGYALPHAILRLDLAGRDLTDYLMKILTER**GYMFTTSAER**EIVRDIKEKLAYVALDYEQESETAKSSSVIEKNYEL
PDGQVITIGAERFRCPEVLFQPSLVGMESAGIHETTYNSIMKCDVDIRKDLYGNIVLSGGSTMFPGIADRMSKEITALAPSSMKIK
VVAPPERKYSVWIGGSILASLSTFQQMWISK**GEYDESGPSIVHR**KCF

Query	Observed	Mr(expt)	Mr(calc)	ppm	Miss	Score	Expect	Rank	Unique	Peptide
1	767.4515	766.4442	766.4337	13.6	0	24	0.082	1		K.VVAPPER.K
6	1162.5314	1161.5241	1161.5125	10.0	0	41	0.0021	1	U	R.GYMFTTSAER.E
7	1178.5322	1177.5249	1177.5074	14.9	0	(29)	0.035	1	U	R.GYMFTTSAER.E + Oxidation (M)
10	1445.6800	1444.6727	1444.6583	10.00	0	88	3.6e-008	1	U	K.GEYDESGPSIVHR.K
13	1515.7561	1514.7489	1514.7419	4.62	0	4	11	5		K.IWHHTFYNELR.V
24	1954.0721	1953.0649	1953.0571	3.98	0	95	5.2e-009	1		R.VAPEEHPVLLTEAPLNPK.A
35	3151.7245	3150.7172	3150.6350	26.1	0	118	1.5e-011	1		R.TTGIVLDSGDGVSHTVPIYEGYALPHAILR.L

2. Unigene219_SALfmcTARAAPEI-3 Mass: 39335 Score: 333 Matches: 7(3) Sequences: 7(3)

gi|514814400 actin-1-like isoform X2 [Setaria italica]

Query	Observed	Mr(expt)	Mr(calc)	ppm	Miss	Score	Expect	Rank	Unique	Peptide
1	767.4515	766.4442	766.4337	13.6	0	24	0.082	1		K.VVAPPER.K
8	1198.7164	1197.7091	1197.6982	9.09	0	51	0.00016	1		R.AVFPSIVGRPR.H
9	1338.6415	1337.6343	1337.6211	9.81	1	20	0.29	1		K.DAYVGDEAQSKR.G
13	1515.7561	1514.7489	1514.7419	4.62	0	4	11	5		K.IWHHTFYNELR.V
24	1954.0721	1953.0649	1953.0571	3.98	0	95	5.2e-009	1		R.VAPEEHPVLLTEAPLNPK.A
31	2341.1868	2340.1795	2340.1784	0.47	1	22	0.098	1	U	R.KDLYGNIVLSGGTTMFPGIADR.M + Oxidation (M)
35	3151.7245	3150.7172	3150.6350	26.1	0	118	1.5e-011	1		R.TTGIVLDSGDGVSHTVPIYEGYALPHAILR.L

3. Unigene9679_SALfmcTARAAPEI-3 Mass: 9992 Score: 113 Matches: 4(2) Sequences: 4(2)

gi|402578741 actin-2, partial [*Wuchereria bancrofti*]

Query	Observed	Mr(expt)	Mr(calc)	ppm	Miss	Score	Expect	Rank	Unique	Peptide
4	976.4596	975.4524	975.4410	11.6	0	39	0.0037	1		-.AGFAGDDAPR.A
8	1198.7164	1197.7091	1197.6982	9.09	0	51	0.00016	1		R.AVFPSIVGRPR.H
9	1338.6415	1337.6343	1337.6211	9.81	1	20	0.29	1		K.DAYVGDEAQSKR.G
13	1515.7561	1514.7489	1514.7419	4.62	0	4	11	5		K.IWHHTFYNELR.V

4. Unigene25242_SALfmcTARAAPEI-3 Mass: 6633 Score: 108 Matches: 3(2) Sequences: 3(2)

gi|575488799 beta actin, partial [Paramoeba pemaquidensis]

Query	Observed	Mr(expt)	Mr(calc)	ppm	Miss	Score	Expect	Rank	Unique	Peptide
4	976.4596	975.4524	975.4410	11.6	0	39	0.0037	1		K.AGFAGDDAPR.A
8	1198.7164	1197.7091	1197.6982	9.09	0	51	0.00016	1		R.AVFPSIVGRPR.H
9	1338.6415	1337.6343	1337.6212	9.80	1	18	0.47	2	U	K.DSFVGDEAQSKR.G

Spot 1086

NCBInr protein database

Match to: gi|322422113 Mass: 41941 Score: 122 Matches: 3(2) Sequences: 3(2)

actin 1 [*Celosia argentea*]

Matched peptides shown in **bold red**.

1 MADAEEIQPL VCDNGTGMVK AGFAGDDAPR AVFPSIVGRP RHTGVMVGMG

51 QKDAYVGDEA QSKRGILTLK YPIEHGIVSN WDDMEKIWHH TFYNELRVAP

101 EEHPVLLTEA PLNPKANREK MTQIMFETFN VPAMYVAIQA VLSLYASGRT

151 TGIVLDSGDG VSHTVPIYEG YALPHAILRL DLAGRDLTDY LMKILTERGY

201 MFTTSAEREI VRDIKEKLAY VALDFEQESE TAKSSSAIEK NYELPDGQVI

251 TIGAERFRCP EVLFQPSLIG MEAAGIHETT YNSIMKCDVDIRKDLYGNIV

301 LSGGTTMFPG IADRMSKEIT ALAPSSMKIKVVAPPERKYS VWIGGSILAS

351 LSTFQQMWIS KGEYDESGPS IVHRKCF

Query	Observed	Mr(expt)	Mr(calc)	ppm	Miss	Score	Expect	Rank	Unique	Peptide
26	1445.6339	1444.6266	1444.6583	-21.90	0	47	0.051	1	U	K.GEYDESGPSIVHR.K
40	1813.8455	1812.8382	1812.8781	-22.01	0	22	16	1	U	K.LAYVALDFEQESETAK.S
43	1954.0253	1953.0180	1953.0571	-20.00	0	54	0.0098	1	U	R.VAPEEHPVLLTEAPLNPK.A

Database 1

Match to: Unigene44960_SeCKS transcribed RNA sequence Mass: 39806 Score: 83 Matches: 3(1) Sequences: 3(1)

gi| 322422113 actin 1 [*Celosia argentea*]

Matched peptides shown in bold red.

IKMADAEEIQPLVCDNGTGMVKAGFAGDDAPRAVFPSIVGRPRHTGVMVGMGQKDAYVGDEAQSKRGILTLKYPIEHGIVSN

WDDMEKIWHHTFYNELRVAPEEHPVLLTEAPLNPKANREKMTQIMFETFNVPAMYVAIQAVLSLYASGRTTGIVLDSGDGVSH

TVPIYEGYALPHAILRLDLAGRDLTDYLMKILTERGYMFTTSAEREIVRDIKEKLAYVALDFEQESETAKSSSAIEKNYELPDGQ

VITIGAERFRCPEVLFQPSLIGMEAAGIHETTYNSIMKCDVDIRKDLYGNIVLSGGTTMFPGIADRMSKEITALAPSSMKIKVVAP

PERKYSVWIGGSILASLSTFQQM

Query	Observed	Mr(expt)	Mr(calc)	ppm	Miss	Score	Expect	Rank	Unique	Peptide
15	1178.5026	1177.4953	1177.5074	-10.26	0	8	3.4	2	U	R.GYMFTTSAER.E + Oxidation (M)
40	1813.8455	1812.8382	1812.8781	-22.01	0	22	0.23	1	U	K.LAYVALDFEQESETAK.S
43	1954.0253	1953.0180	1953.0571	-20.00	0	54	0.00018	1	U	R.VAPEEHPVLLTEAPLNPK.A

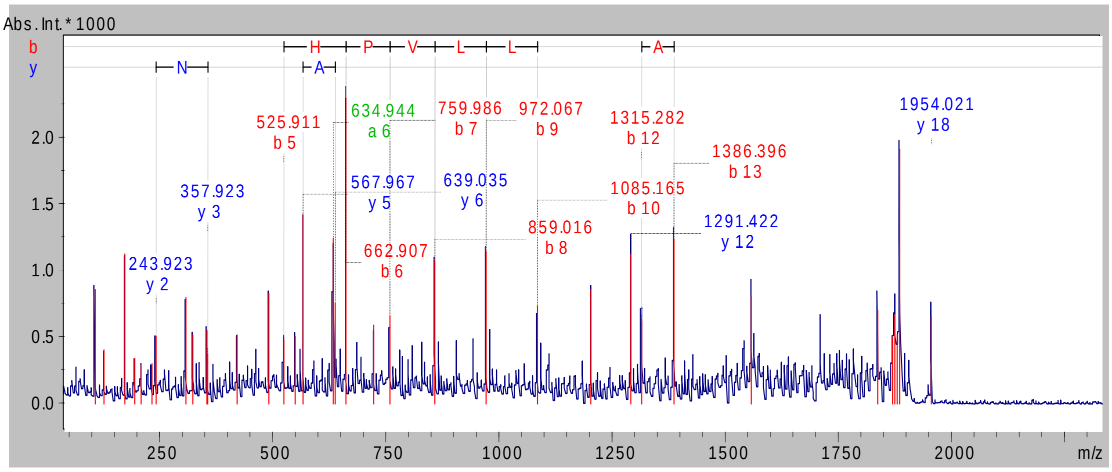

Proteins matching the same set of peptides:

Unigene47152_Se200S transcribed RNA sequence Mass: 41001 Score: 83 Matches: 3(1) Sequences: 3(1)

gi| 322422113 actin 1 [*Celosia argentea*]

Database 2

Match to: Unigene54790_SALfmcTARAAPEI-3 Mass: 33967 Score: 79 Matches: 3(2) Sequences: 3(2)

gi| 322422113 actin 1 [*Celosia argentea*]

Matched peptides shown in **bold red**.

GIVSNWDDMEKIWHHTFYNELR**VAPEEHPVLLTEAPLNPK**ANREKMTQIMFETFNVPAMYVAIQAVLSLYASGRTTGIVLDSGD

GVSHTVPIYEGYALPHAILRLDLAGRDLTDYLMKILTER**GYMFTTSAER**EIVRDIKEKLAYVALDYEQESETAKSSSVIEKNYEL

PDGQVITIGAERFRCPEVLFQPSLVGMESAGIHETTYNSIMKCDVDIRKDLYGNIVLSGGSTMFPGIADRMSKEITALAPSSMKIK

VVAPPERKYSVWIGGSILASLSTFQQMWISK**GEYDESGPSIVHR**KCF

Query	Observed	Mr(expt)	Mr(calc)	ppm	Miss	Score	Expect	Rank	Unique	Peptide
15	1178.5026	1177.4953	1177.5074	-10.26	0	8	3.5	1	U	R.GYMFTTSAER.E + Oxidation (M)
26	1445.6339	1444.6266	1444.6583	-21.90	0	47	0.00044	1	U	K.GEYDESGPSIVHR.K
43	1954.0253	1953.0180	1953.0571	-20.00	0	54	8e-005	1	U	R.VAPEEHPVLLTEAPLNPK.A

Spot 1095

NCBInr protein database

Match to: gi|113232 Mass: 42112 Score: 83 Matches: 3(1) Sequences: 3(1)

RecName: Full=Actin-2 [*Daucus carota*]

Matched peptides shown in bold red.

1 MADGGEDIQP LVCDNGTGMV K**AGFAGDDAP R**AVFPSIVVG RPRHTGVMVG

51 MGQKDAYVGD EAQSKRGILT LKYPIEHGIV SNWDDMEKIS HHTFYNELRV

101 **APEEHPVLLT EAPLNPK**ANR EKMTQIMFET FNVPAMYVLS RLRSCLSLYA

151 SGRTTGIVLD SGDGVSHTVP IYEGYALPHA ILRLDLAGRD LTDGLMKILT

201 ERGYMFTTTA TGMSYMKEKL AYVALVMSKS WRLPRARLLV EKNYELPDGQ

251 VITIGAVRGS GCPEVLFQPS MIGMESAGIH ETTYNSIMKC DVDIRKDLYG

301 NIVLSGGSTM FPGSCYASMS KEITALAPSS MKIKVVAPPE RKYSVWIGGS

351 ILASLSTFQQ MWISK**GEYDE SGPSIVHR**KCF

Query	Observed	Mr(expt)	Mr(calc)	ppm	Miss	Score	Expect	Rank	Unique	Peptide
3	976.4723	975.4650	975.4410	24.6	0	21	31	2		K.AGFAGDDAPR.A
20	1445.7088	1444.7015	1444.6583	29.9	0	13	1.4e+02	1	U	K.GEYDESGPSIVHR.K
29	1954.1057	1953.0984	1953.0571	21.2	0	49	0.023	1	U	R.VAPEEHPVLLTEAPLNPK.A

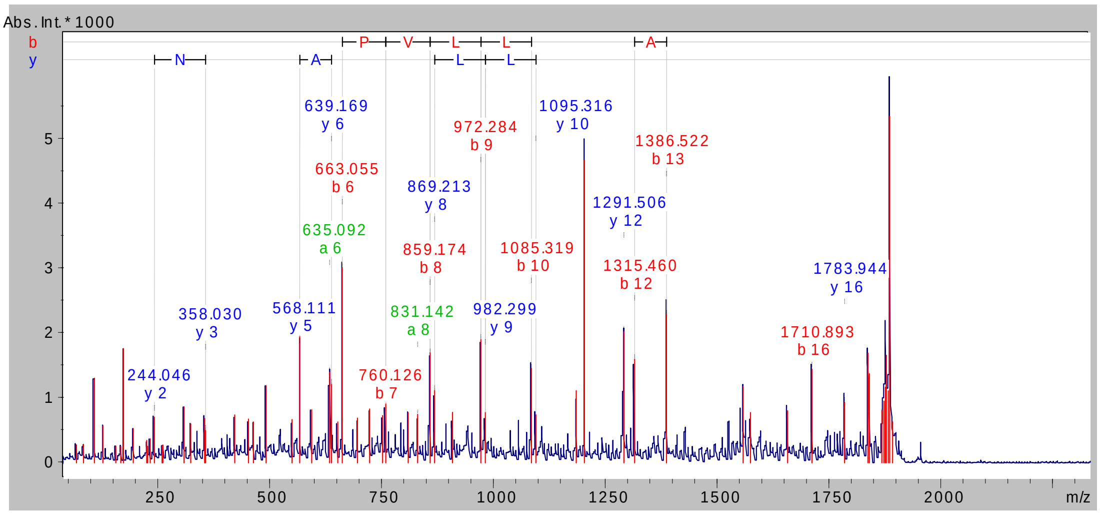

Proteins matching the same set of peptides:

gi|4139264 Mass: 41888 Score: 83 Matches: 3(1) Sequences: 3(1)

actin [*Brassica napus*]

gi|5230841 Mass: 41968 Score: 83 Matches: 3(1) Sequences: 3(1)

actin [*Malva pusilla*]

gi|6683504 Mass: 41931 Score: 83 Matches: 3(1) Sequences: 3(1)

actin isoform B [*Mimosa pudica*]

gi|9082317 Mass: 41934 Score: 83 Matches: 3(1) Sequences: 3(1)

actin [*Helianthus annuus*]

gi|23955912 Mass: 41943 Score: 83 Matches: 3(1) Sequences: 3(1)

actin [*Stevia rebaudiana*]

gi|32186890 Mass: 41917 Score: 83 Matches: 3(1) Sequences: 3(1)

actin [*Gossypium hirsutum*]

gi|32186892 Mass: 41926 Score: 83 Matches: 3(1) Sequences: 3(1)

actin [*Gossypium hirsutum*]

gi|32186894 Mass: 41899 Score: 83 Matches: 3(1) Sequences: 3(1)

actin [*Gossypium hirsutum*]

gi|32186896 Mass: 41935 Score: 83 Matches: 3(1) Sequences: 3(1)

actin [*Gossypium hirsutum*]

gi|32186898 Mass: 41989 Score: 83 Matches: 3(1) Sequences: 3(1)

actin [*Gossypium hirsutum*]

gi|32186900 Mass: 41902 Score: 83 Matches: 3(1) Sequences: 3(1)

actin [*Gossypium hirsutum*]

gi|32186902 Mass: 42057 Score: 83 Matches: 3(1) Sequences: 3(1)

actin [*Gossypium hirsutum*]

gi|32186904 Mass: 42106 Score: 83 Matches: 3(1) Sequences: 3(1)

actin [*Gossypium hirsutum*]

gi|32186906 Mass: 41913 Score: 83 Matches: 3(1) Sequences: 3(1)

actin [*Gossypium hirsutum*]

gi|32186908 Mass: 42015 Score: 83 Matches: 3(1) Sequences: 3(1)

actin [*Gossypium hirsutum*]

gi|32186910 Mass: 41876 Score: 83 Matches: 3(1) Sequences: 3(1)

actin [*Gossypium hirsutum*]

gi|32186912 Mass: 41936 Score: 83 Matches: 3(1) Sequences: 3(1)

actin [*Gossypium hirsutum*]

gi|32186916 Mass: 41929 Score: 83 Matches: 3(1) Sequences: 3(1)

actin [*Gossypium hirsutum*]

gi|34541966 Mass: 41885 Score: 83 Matches: 3(1) Sequences: 3(1)

actin [*Trifolium pratense*]

gi|38259662 Mass: 41869 Score: 83 Matches: 3(1) Sequences: 3(1)

actin [*Ricinus communis*]

gi|50058115 Mass: 41885 Score: 83 Matches: 3(1) Sequences: 3(1)

actin [*Nicotiana tabacum*]

gi|55820068 Mass: 41948 Score: 83 Matches: 3(1) Sequences: 3(1)

stem cambial region actin protein [*Eucommia ulmoides*]

gi|56181504 Mass: 40336 Score: 83 Matches: 3(1) Sequences: 3(1)

putative actin 1 [*Chorispora bungeana*]

gi|58013197 Mass: 42020 Score: 83 Matches: 3(1) Sequences: 3(1)

actin [*Isatis tinctoria*]

gi|149938964 Mass: 41865 Score: 83 Matches: 3(1) Sequences: 3(1)

ACT1 [*Actinidia deliciosa*]

gi|158529884 Mass: 41827 Score: 83 Matches: 3(1) Sequences: 3(1)

actin [*Glycyrrhiza uralensis*]

gi|182409985 Mass: 41885 Score: 83 Matches: 3(1) Sequences: 3(1)

actin [*Betula platyphylla*]

gi|209979576 Mass: 41880 Score: 83 Matches: 3(1) Sequences: 3(1)

actin [*Sedum alfredii*]

gi|209979578 Mass: 41839 Score: 83 Matches: 3(1) Sequences: 3(1)

actin [*Sedum alfredii*]

gi|220978696 Mass: 41922 Score: 83 Matches: 3(1) Sequences: 3(1)

actin 1 [*Gossypium hirsutum*]

gi|220978698 Mass: 41938 Score: 83 Matches: 3(1) Sequences: 3(1)

actin 2 [*Gossypium hirsutum*]

gi|224055984 Mass: 41897 Score: 83 Matches: 3(1) Sequences: 3(1)

actin family protein [*Populus trichocarpa*]

gi|224552368 Mass: 41883 Score: 83 Matches: 3(1) Sequences: 3(1)

actin [*Carica papaya*]

gi|225431585 Mass: 41927 Score: 83 Matches: 3(1) Sequences: 3(1)

PREDICTED: actin-7 [*Vitis vinifera*]

gi|225441455 Mass: 41918 Score: 83 Matches: 3(1) Sequences: 3(1)

PREDICTED: actin [*Vitis vinifera*]

gi|255031691 Mass: 41510 Score: 83 Matches: 3(1) Sequences: 3(1)

actin [*Prunus avium*]

gi|255115691 Mass: 41867 Score: 83 Matches: 3(1) Sequences: 3(1)

actin 1 [*Boehmeria nivea*]

gi|268376311 Mass: 41861 Score: 83 Matches: 3(1) Sequences: 3(1)

actin 7-like protein [*Pelargonium x hortorum*]

gi|281485191 Mass: 41854 Score: 83 Matches: 3(1) Sequences: 3(1)

actin [*Persea americana*]

gi|294718134 Mass: 41941 Score: 83 Matches: 3(1) Sequences: 3(1)

actin 2 [*Pyrus x bretschneideri*]

gi|296088608 Mass: 45585 Score: 83 Matches: 3(1) Sequences: 3(1)

unnamed protein product [*Vitis vinifera*]

gi|296881978 Mass: 41973 Score: 83 Matches: 3(1) Sequences: 3(1)

actin [*Jatropha curcas*]

gi|297739832 Mass: 31826 Score: 83 Matches: 3(1) Sequences: 3(1)

unnamed protein product [*Vitis vinifera*]

gi|315613890 Mass: 41897 Score: 83 Matches: 3(1) Sequences: 3(1)

actin 1 [*Morus alba*]

gi|316953632 Mass: 41810 Score: 83 Matches: 3(1) Sequences: 3(1)

actin [*Morus alba*]

gi|317160666 Mass: 41924 Score: 83 Matches: 3(1) Sequences: 3(1)

beta-actin [*Camellia sinensis*]

gi|319998245 Mass: 41931 Score: 83 Matches: 3(1) Sequences: 3(1)

actin [*Ananas comosus*]

gi|322422113 Mass: 41941 Score: 83 Matches: 3(1) Sequences: 3(1)

actin 1 [*Celosia argentea*]

gi|323650491 Mass: 41924 Score: 83 Matches: 3(1) Sequences: 3(1)

actin 9 [*Mangifera indica*]

gi|324984003 Mass: 41921 Score: 83 Matches: 3(1) Sequences: 3(1)

actin [Gossypium herbaceum subsp. africanum]

gi|340784744 Mass: 41813 Score: 83 Matches: 3(1) Sequences: 3(1)

actin [*Paeonia suffruticosa*]

gi|340784746 Mass: 41929 Score: 83 Matches: 3(1) Sequences: 3(1)

actin [*Paeonia lactiflora*]

gi|342837524 Mass: 41869 Score: 83 Matches: 3(1) Sequences: 3(1)

actin1 [*Morella rubra*]

gi|344939558 Mass: 41891 Score: 83 Matches: 3(1) Sequences: 3(1)

actin 7 [*Brassica rapa subsp. pekinensis*]

gi|345103851 Mass: 41883 Score: 83 Matches: 3(1) Sequences: 3(1)

actin 1 [*Gossypium thurberi*]

gi|345103865 Mass: 41901 Score: 83 Matches: 3(1) Sequences: 3(1)

actin 1 [*Gossypium darwinii*]

gi|345103875 Mass: 41885 Score: 83 Matches: 3(1) Sequences: 3(1)

actin 1 [*Gossypium barbadense var. peruvianum*]

gi|346683577 Mass: 41863 Score: 83 Matches: 3(1) Sequences: 3(1)

actin 1 [*Mangifera indica*]

gi|347949168 Mass: 41929 Score: 83 Matches: 3(1) Sequences: 3(1)

actin [*Fragaria x ananassa*]

gi|355329944 Mass: 40369 Score: 83 Matches: 3(1) Sequences: 3(1)

actin, partial [*Malus domestica*]

gi|356509003 Mass: 41917 Score: 83 Matches: 3(1) Sequences: 3(1)

PREDICTED: actin-7-like isoform X1 [*Glycine max*]

gi|356512803 Mass: 41940 Score: 83 Matches: 3(1) Sequences: 3(1)

PREDICTED: actin-7-like [*Glycine max*]

gi|357464527 Mass: 42514 Score: 83 Matches: 3(1) Sequences: 3(1)

Actin [*Medicago truncatula*]

gi|357464529 Mass: 40126 Score: 83 Matches: 3(1) Sequences: 3(1)

Actin [*Medicago truncatula*]

gi|378407816 Mass: 41913 Score: 83 Matches: 3(1) Sequences: 3(1)

actin 2 [*Medicago sativa*]

gi|388495758 Mass: 41814 Score: 83 Matches: 3(1) Sequences: 3(1)

unknown [*Medicago truncatula*]

gi|391324436 Mass: 41848 Score: 83 Matches: 3(1) Sequences: 3(1)

actin [*Sophora viciifolia*]

gi|399525600 Mass: 41913 Score: 83 Matches: 3(1) Sequences: 3(1)

actin [*Lycoris longituba*]

gi|449459238 Mass: 41911 Score: 83 Matches: 3(1) Sequences: 3(1)

PREDICTED: actin-7-like [*Cucumis sativus*]

gi|449533733 Mass: 41476 Score: 83 Matches: 3(1) Sequences: 3(1)

PREDICTED: actin-7-like, partial [*Cucumis sativus*]

gi|460378623 Mass: 41959 Score: 83 Matches: 3(1) Sequences: 3(1)

PREDICTED: actin-7-like [*Solanum lycopersicum*]

gi|460408874 Mass: 41986 Score: 83 Matches: 3(1) Sequences: 3(1)

PREDICTED: actin-7-like [*Solanum lycopersicum*]

gi|470141751 Mass: 41959 Score: 83 Matches: 3(1) Sequences: 3(1)

PREDICTED: actin-7-like [*Fragaria vesca subsp. vesca*]

gi|471271028 Mass: 41923 Score: 83 Matches: 3(1) Sequences: 3(1)

actin 1 [*Dionaea muscipula*]

gi|475347358 Mass: 41822 Score: 83 Matches: 3(1) Sequences: 3(1)

Actin-3 [*Aegilops tauschii*]

gi|525314449 Mass: 41883 Score: 83 Matches: 3(1) Sequences: 3(1)

uncharacterized protein LOC101503086 [*Cicer arietinum*]

gi|557159425 Mass: 41872 Score: 83 Matches: 3(1) Sequences: 3(1)

actin 7, partial [*Litsea cubeba*]

gi|564586964 Mass: 41890 Score: 83 Matches: 3(1) Sequences: 3(1)

actin-2 [*Sedum alfredii*]

gi|565459906 Mass: 41907 Score: 83 Matches: 3(1) Sequences: 3(1)

hypothetical protein CARUB_v10001188mg [*Capsella rubella*]

gi|566225310 Mass: 41913 Score: 83 Matches: 3(1) Sequences: 3(1)

hypothetical protein POPTR_0019s02630g [*Populus trichocarpa*]

gi|567870467 Mass: 41911 Score: 83 Matches: 3(1) Sequences: 3(1)

hypothetical protein CICLE_v10025866mg [*Citrus clementina*]

gi|569533237 Mass: 41917 Score: 83 Matches: 3(1) Sequences: 3(1)

actin [*Lycium chinense*]

gi|590708352 Mass: 57488 Score: 83 Matches: 3(1) Sequences: 3(1)

Actin 7 isoform 1 [*Theobroma cacao*]

Database 1

Match to: Unigene10014_Se200S transcribed RNA sequence Mass: 16803 Score: 70 Matches: 2(1) Sequences: 2(1)

GI:106879659 actin 1 [*Plantago major*]

Matched peptides shown in bold red.

MVKAGFAGDDAPRAVFPSIVGRPRHTGVMVGMGQKDAYVGDEAQSKRGILTLKYPIEHGIVSNWDDMEKIWHHTFYNELRV

APEEHPVLLTEAPLNPKANREKMTQIMFETFNTPAMYVAIQAVLSLYASGRTTGIVLDSGDGVSHTVPIYE

Query	Observed	Mr(expt)	Mr(calc)	ppm	Miss	Score	Expect	Rank	Unique	Peptide
3	976.4723	975.4650	975.4410	24.6	0	21	0.51	1	U	K.AGFAGDDAPR.A
29	1954.1057	1953.0984	1953.0571	21.2	0	49	0.00027	1	U	

R.VAPEEHPVLLTEAPLNPK.A

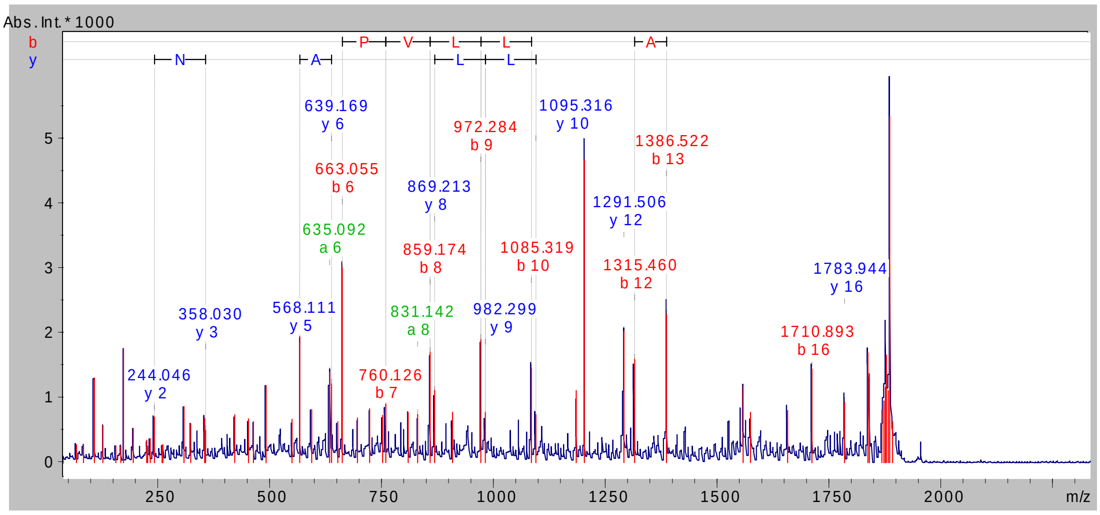

Proteins matching the same set of peptides:

Unigene47152_Se200S transcribed RNA sequence Mass: 34860 Score: 70 Matches: 2(1) Sequences: 2(1)

gi|322422113 actin 1 [*Celosia argentea*]

Unigene44960_SeCKS transcribed RNA sequence Mass: 39806 Score: 70 Matches: 2(1) Sequences: 2(1)

gi|322422113 actin 1 [*Celosia argentea*]

Unigene55357_SeCKS transcribed RNA sequence Mass: 14992 Score: 70 Matches: 2(1) Sequences: 2(1)

gi|254802952 actin, partial [*Nicotiana tabacum*]

Database 2

Match to: Unigene54790_SALfmcTARAAPEI-3 Mass: 33967 Score: 62 Matches: 2(1) Sequences: 2(1)

gi| 322422113 actin 1 [*Celosia argentea*]

Matched peptides shown in **bold red**.

GIVSNWDDMEKIWHHTFYNELR**VAPEEHPVLLTEAPLNPK**ANREKMTQIMFETFNVPAMYVAIQAVLSLYASGRTTGIVLDSGD
GVSHTVPIYEGYALPHAILRLDLAGRDLTDYLMKILTERGYMFTTSAEREIVRDIKEKLAYVALDYEQESETAKSSSVIEKNYEL
PDGQVITIGAERFRCPEVLFQPSLVGMESAGIHETTYNSIMKCDVDIRKDLYGNIVLSGGSTMFPGIADRMSKEITALAPSSMKIK
VVAPPERKYSVWIGGSILASLSTFQQMWISK**GEYDESGPSIVHR**KCF

Query	Observed	Mr(expt)	Mr(calc)	ppm	Miss	Score	Expect	Rank	Unique	Peptide
20	1445.7088	1444.7015	1444.6583	29.9	0	13	1.2	1	U	K.GEYDESGPSIVHR.K
29	1954.1057	1953.0984	1953.0571	21.2	0	49	0.00018	1	U	

R.VAPEEHPVLLTEAPLNPK.A

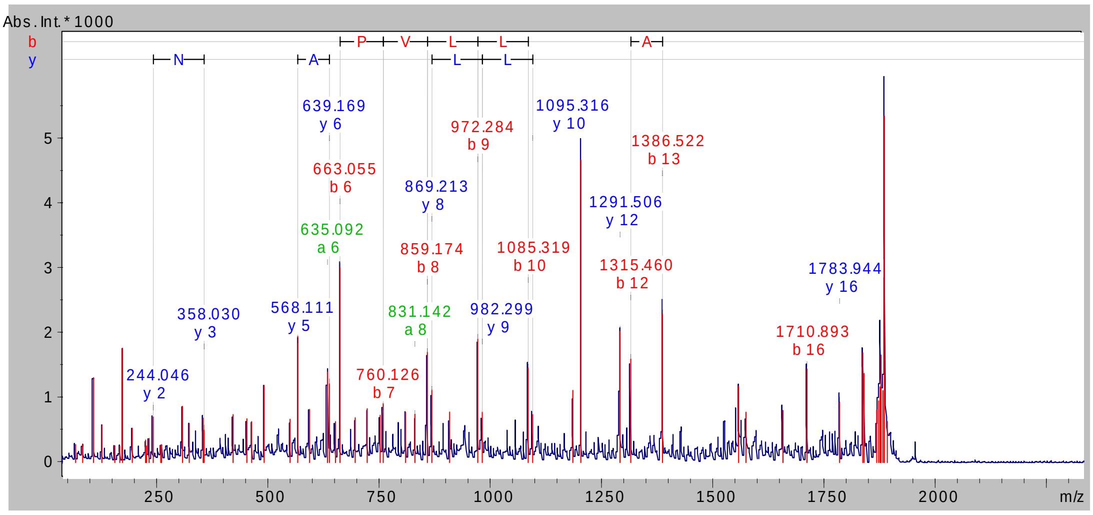

Spot 1141

Database 1

Match to: Unigene53689_SeCKS transcribed RNA sequence Mass: 32953 Score: 62 Matches: 2(1) Sequences: 2(1)

gi| 565393170 glutamate dehydrogenase-like [*Solanum tuberosum*]

Matched peptides shown in **bold red**.

ILRTMNALAATNRNFKLASRLLGLDSKLERSLLIPFREIKVECTIPK**DDGSIASFVGFR**IQHDNARGPMKGGIRYHPEVDPDEVN

ALAQLMTWKTAVANIPYGGAKGGIGCNPSELSATELERLTRVFTQRIHDLIGIHTDVPAPDMGTNSQTMAWMLDEYSKFHGHS

PAVVTGKPIDLGGSLGRDAATGRGVLFATEALLNDHGKSISGQRFVIQGFGNVGSWAARLISELGGK**VVAVSDISGAIR**NKNGL

DIDSLLKHVKENRGVKGFHDADAMDSNSILVEDCDVLIPAALGGVINKDNANEV

Query	Observed	Mr(expt)	Mr(calc)	ppm	Miss	Score	Expect	Rank	Unique	Peptide
25	1186.6991	1185.6918	1185.6717	16.9	0	26	0.097	1	U	K.VVAVSDISGAIR.N
28	1270.6440	1269.6367	1269.5990	29.7	0	36	0.012	1	U	K.DDGSIASFVGFR.I

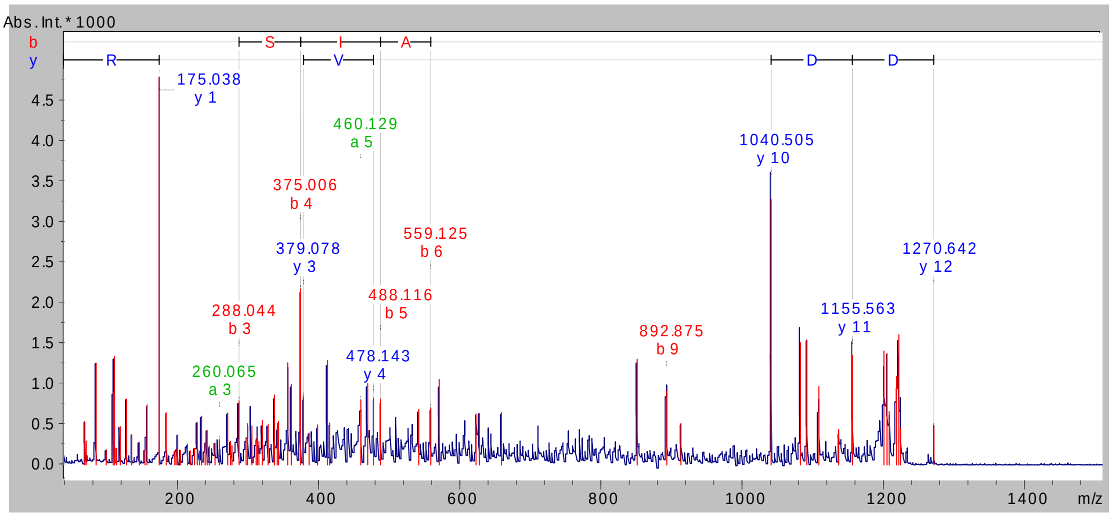

Database 2

Match to: Unigene54041_SALfmcTARAAPEI-3 Mass: 28831 Score: 36 Matches: 2(1) Sequences: 2(1)

gi| 12643806 RecName: Full=Glutamate dehydrogenase B; Short=GDH B [*Nicotiana plumbaginifolia*]

Matched peptides shown in **bold red**.

MNALAATNRNFKLASRLLGLDSKLERSLLIPFREIKVECTIPK**DDGSIASFVGFR**IQHDNARGPMKGGIRYHPEVDPDEVNALA

QLMTWKTAVANIPYGGAKGGIGCNPSELSATELERLTRVFTQRIHDLIGIHTDVPAPDMGTNSQTMAWMLDEYSKFHGHSPAVV

TGKPIDLGGSLGRDAATGRGVLFATEALLNDHGKSISGQRFVIQGFGNVGSWAARLISELGGKVVAVSDISGAIRNKNGLDIDSL

LKHVKENRGVKGFH

Query	Observed	Mr(expt)	Mr(calc)	ppm	Miss	Score	Expect	Rank	Unique	Peptide
25	1186.6991	1185.6918	1185.6717	16.9	0	26	0.06	1	U	K.VVAVSDISGAIR.N
28	1270.6440	1269.6367	1269.5990	29.7	0	36	0.0065	1	U	K.DDGSIASFVGFR.I

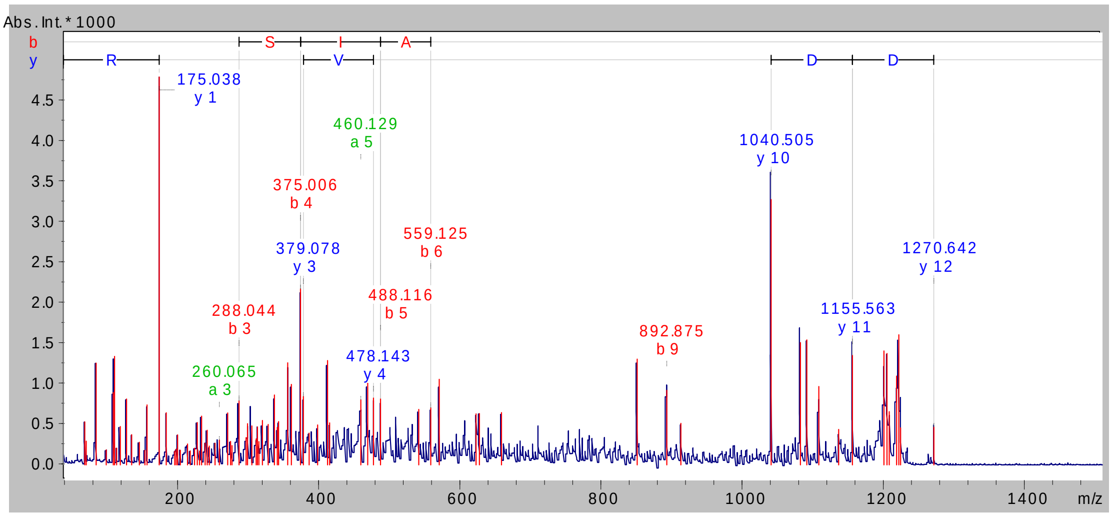

Proteins matching the same set of peptides:

Unigene56548_Se200S transcribed RNA sequence Mass: 33081 Score: 62 Matches: 2(1) Sequences: 2(1)

gi| 565393170 glutamate dehydrogenase-like [*Solanum tuberosum*]

Spot 1230

NCBInr protein database

Match to: gi|13173419 Mass: 39240 Score: 215 Matches: 4(2) Sequences: 4(2)

cytosolic glutamine synthetase [*Beta vulgaris*]

Matched peptides shown in **bold red**.

1 MALLNDLINL NLSETSDKII AEYIWIGGSG LDMRSKARTL TGPISDPAKL

51 PKWNYDGSST NQAPGEDSEV ILYPQAIFKD PFRRGDNILV MCDAYTPAGE

101 PIPTNKRYNA EKIFSHPDVV AEEPWYGIEQ EYTLLQKDIN WPLGWPTGGF

151 PGPQGPYYCG VGADKSFGRD IVDAHYKACI YAGVNISGIN GEVMPGQWEF

201 QVGPTVGISS GDQVWVARYI LERIAEIAGV VVSFDPKPVK GDWNGAGAHT

251 NYSTKSMRED GGINVIKAAI EKLSLRHKEH IAAYGEGNER RLTGRHETAD

301 ITTFSWGVAN RGASVRVGRD TEKDGKGYFE DRRPASNMDP YVVTSMIAET

351 TILGKP

Query	Observed	Mr(expt)	Mr(calc)	ppm	Miss	Score	Expect	Rank	Unique	Peptide
1	786.3691	785.3618	785.3344	34.9	0	16	48	4		K.GYFEDR.R
17	1345.6523	1344.6450	1344.6058	29.1	0	44	0.12	1	U	K.EHIAAYGEGNER.R
22	1610.7993	1609.7921	1609.7597	20.1	1	111	1.9e-08	1	U	R.HKEHIAAYGEGNER.R
36	2532.2658	2531.2585	2531.2148	17.2	1	46	0.041	1	U	

R.RGDNILVMCDAYTPAGEPIPTNK.R

Database 1

Match to: Unigene86984_SeCKS transcribed RNA sequence Mass: 27229 Score: 282 Matches: 4(3) Sequences: 4(3)

gi| 13173419 cytosolic glutamine synthetase [*Beta vulgaris*]

Matched peptides shown in **bold red**.

EKIFNHPDVAAEEPWYGIEQEYTLLQKDINWPLGWPVGGFPGPQGPYYCGVGADKSFGRDIVDAHYKACLYAGINISGINGEV

MPGQWEFQVGPSVGISSGDQVWVARYILERIAEIAGVVVTFDPKPVKGDWNGAGAHTNYSTKSMRNDGGIEVIKKAIEKLGL

R**HKEHIAAYGEGNER**RLTGR**HETADISTFSWGVANR**GASVRVGRDTEKDGK**GYFEDR**RPASNMDPYVVTSMIAETTILWKP

Query	Observed	Mr(expt)	Mr(calc)	ppm	Miss	Score	Expect	Rank	Unique	Peptide
1	786.3691	785.3618	785.3344	34.9	0	16	0.57	1	U	K.GYFEDR.R
17	1345.6523	1344.6450	1344.6058	29.1	0	44	0.0022	1	U	K.EHIAAYGEGNER.R
22	1610.7993	1609.7921	1609.7597	20.1	1	111	4.4e-010	1	U	R.HKEHIAAYGEGNER.R
26	1790.8721	1789.8648	1789.8384	14.8	0	110	4.5e-010	1	U	R.HETADISTFSWGVANR.G

Proteins matching the same set of peptides:

Unigene89771_Se200S transcribed RNA sequence Mass: 27229 Score: 282 Matches: 4(3) Sequences: 4(3)

gi|13173419 cytosolic glutamine synthetase [*Beta vulgaris*]

Spot 1239

NCBInr protein database

Match to: gi|113171384 Mass: 39338 Score: 217 Matches: 5(2) Sequences: 4(2)

cytosolic glutamine synthetase [*Cucumis melo*]

Matched peptides shown in **bold red**.

1 MSLLSDLINL NLSDSTEKII AEYIWIGGSG MDLRSKARTL SGPVSDPPKL

51 PKWNYDGSST GQAPGEDSEV ILYPQAIFRD PFRRGNNTLV ICDAYTPAGE

101 PIPTNKRHAA AKIFSHPDVV AEVPWYGIEQ EYTLLQKDVK WPIGWPIGGF

151 PGPQGPYYCG VGVDKAFGRD IVDAHYKACL YAGVNISGIN GEVMPGQWEF

201 QVGPSVGISA GDELWVARYI LERITEIAGV VLSFDPKPIQ GDWNGAGAHT

251 NYSTK**SMREE GGYEVIK**KAI EKLKLR**HKEH IAAYGEGNER** RLTGRHETAD

301 INTFSWGVAN RGASVRVGRD TEKEGK**GYFE DR**RPASNMGP YVVTSMVAET

351 TILWKP

Query	Observed	Mr(expt)	Mr(calc)	ppm	Miss	Score	Expect	Rank	Unique	Peptide
1	786.3517	785.3444	785.3344	12.7	0	19	18	2		K.GYFEDR.R
5	1345.6321	1344.6249	1344.6058	14.2	0	65	0.00083	1	U	K.EHIAAYGEGNER.R
6	1397.6919	1396.6846	1396.6656	13.6	1	20	29	5	U	K.SMREEGGYEVIK.K
7	1413.6843	1412.6770	1412.6606	11.6	1	(16)	83	4	U	K.SMREEGGYEVIK.K + Oxidation (M)
10	1610.7769	1609.7696	1609.7597	6.19	1	112	1.4e-08	1	U	R.HKEHIAAYGEGNER.R

Proteins matching the same set of peptides:

gi|356520651 Mass: 39295 Score: 217 Matches: 5(2) Sequences: 4(2)

PREDICTED: glutamine synthetase PR-2 [*Glycine max*]

gi|525507210 Mass: 39423 Score: 217 Matches: 5(2) Sequences: 4(2)

glutamine synthetase cytosolic isozyme-like [*Cucumis sativus*]

gi|565485767 Mass: 39421 Score: 217 Matches: 5(2) Sequences: 4(2)

hypothetical protein CARUB_v10020543mg [*Capsella rubella*]

Database 1

Match to: Unigene5820_SeCKS transcribed RNA sequence Mass: 41411 Score: 293 Matches: 6(3) Sequences: 5(3)

gi| 156072358 cytosolic glutamine synthetase [*Spinacia oleracea*]

Matched peptides shown in **bold red**.

TLDFNSIDPKLLPSSNKSNMSLLSDLVNLDLSDTTEKVIAEYIWIGGSGMDMRSKARTLNGPVSDPKELPKWNYDGSSTGQAP

GEDSEVILYPQAIFKDPFRRGNNILVMCDAYTPQGEPIPTNNRHNAEKIFSHPEVSAEVPWYGIEQEYTLLQKDVNWPLGWPIG
GYPGPQGPYYCGAGADKAFGRDIVDSHYKACLYAGINISGINGEVMPGQWEFQVGPSVGISAGDELWVARYILERITEVAGVSL
SFDPKPIPGDWNGAGAHTNYSTKSMREEGGYEVIKAAIEKLGLKHKEHIAAYGEGNERRLTGKHETASISNFLWGVANRGASV
RVGRDTEKEGKGYFEDRRPASNMDPYVVTSMIAETTIIWKP

Query	Observed	Mr(expt)	Mr(calc)	ppm	Miss	Score	Expect	Rank	Unique	Peptide
1	786.3517	785.3444	785.3344	12.7	0	19	0.2	1	U	K.GYFEDR.R
5	1345.6321	1344.6249	1344.6058	14.2	0	65	1.3e-005	1	U	K.EHIAAYGEGNER.R
6	1397.6919	1396.6846	1396.6656	13.6	1	20	0.59	1	U	K.SMREEGGYEVIK.A
7	1413.6843	1412.6770	1412.6606	11.6	1	(16)	1.3	1	U	K.SMREEGGYEVIK.A + Oxidation (M)
10	1610.7769	1609.7696	1609.7597	6.19	1	112	3.2e-010	1	U	K.HKEHIAAYGEGNER.R
20	2590.2057	2589.1984	2589.1952	1.26	0	76	6.3e-007	1	U	R.GNNILVMCDAYTPQGEPIPTNNR.H + Oxidation (M)

Proteins matching the same set of peptides:

Unigene12389_Se200S transcribed RNA sequence Mass: 41411 Score: 293 Matches: 6(3) Sequences: 5(3) cytosolic gi|156072358 glutamine synthetase [*Spinacia oleracea*]

Spot 1240

NCBInr protein database

Match to: gi|31980481 Mass: 12544 Score: 146 Matches: 3(1) Sequences: 3(1)

cytosolic glutamine synthetase [*Drosera tokaiensis*]

Matched peptides shown in bold red.

1 MPGQWEFQVG PSVGISAGNE LWVARYILER ITEIAGVVLS LDPKPIEGDW

51 NGAGAHTNNS TKSMREEGGY EVIKKAIEKL GLRHKEHIAA YGEGNERRLT

101 GHHETANINT FLW

Query	Observed	Mr(expt)	Mr(calc)	ppm	Miss	Score	Expect	Rank	Unique	Peptide
6	1345.6211	1344.6138	1344.6058	5.95	0	38	0.42	1	U	K.EHIAAYGEGNER.R
9	1413.6699	1412.6626	1412.6606	1.48	1	12	1.9e+02	10	U	K.SMREEGGYEVIK.K + Oxidation (M)
12	1610.7670	1609.7597	1609.7597	0.01	1	96	5.6e-07	1	U	R.HKEHIAAYGEGNER.R

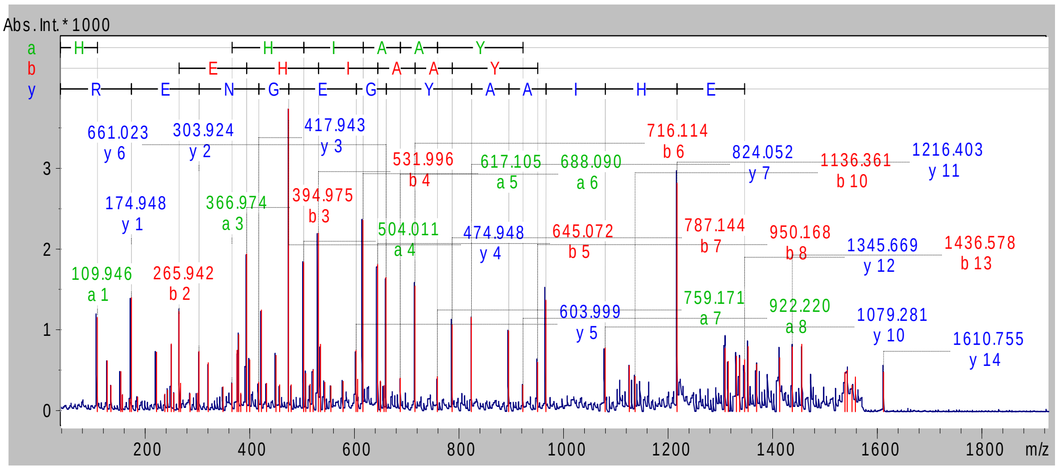

Proteins matching the same set of peptides:

gi|113171384 Mass: 39338 Score: 146 Matches: 3(1) Sequences: 3(1)

cytosolic glutamine synthetase [*Cucumis melo*]

gi|356520651 Mass: 39295 Score: 146 Matches: 3(1) Sequences: 3(1)

PREDICTED: glutamine synthetase PR-2 [*Glycine max*]

gi|525507210 Mass: 39423 Score: 146 Matches: 3(1) Sequences: 3(1)

glutamine synthetase cytosolic isozyme-like [*Cucumis sativus*]

gi|565485767 Mass: 39421 Score: 146 Matches: 3(1) Sequences: 3(1)

hypothetical protein CARUB_v10020543mg [*Capsella rubella*]

Database 1

Match to: Unigene5820_SeCKS transcribed RNA sequence Mass: 41411 Score: 242 Matches: 5(3) Sequences: 5(3)

gi| 156072358 cytosolic glutamine synthetase [*Spinacia oleracea*]

Matched peptides shown in bold red.

TLDFNSIDPKLLPSSNKSNMSLLSDLVNLDLSDTTEKVIAEYIWIGGSGMDMRSKARTLNGPVSDPKELPKWNYDGSSTGQAP

GEDSEVILYPQAIFKDPFRRGNNILVMCDAYTPQGEPIPTNNRHNAEKIFSHPEVSAEVPWYGIEQEYTLLQKDVNWPLGWPIG

GYPGPQGPYYCGAGADKAFGRDIVDSHYKACLYAGINISGINGEVMPGQWEFQVGPSVGISAGDELWVARYILERITEVAGVSL

SFDPKPIPGDWNGAGAHTNYSTKSMREEGGYEVIKAAIEKLGLKHKEHIAAYGEGNERRLTGKHETASISNFLWGVANRGASV

RVGRDTEKEGKGYFEDRRPASNMDPYVVTSMIAETTIIWKP

Query	Observed	Mr(expt)	Mr(calc)	ppm	Miss	Score	Expect	Rank	Unique	Peptide
3	786.3387	785.3314	785.3344	-3.84	0	11	0.97	1	U	K.GYFEDR.R
6	1345.6211	1344.6138	1344.6058	5.95	0	38	0.006	1	U	K.EHIAAYGEGNER.R
9	1413.6699	1412.6626	1412.6606	1.48	1	12	2.6	2	U	K.SMREEGGYEVIK.A + Oxidation (M)
12	1610.7670	1609.7597	1609.7597	0.01	1	96	1.2e-008	1	U	K.HKEHIAAYGEGNER.R
21	2590.1894	2589.1821	2589.1952	-5.04	0	85	9e-008	1	U	R.GNNILVMCDAYTPQGEPIPTNNR.H + Oxidation (M)

Proteins matching the same set of peptides:

Unigene12389_Se200S transcribed RNA sequence Mass: 41411 Score: 242 Matches: 5(3) Sequences: 5(3)

gi|156072358 glutamine synthetase [*Spinacia oleracea*]

Spot 1302

NCBInr protein database

Match to: gi|116782128 Mass: 35854 Score: 218 Matches: 3(2) Sequences: 3(2)

unknown [*Picea sitchensis*]

Matched peptides shown in bold red.

1 MAKEPARVLV TGAAGQIGYA LVPMIARGIM LGLDQPVILH MLDIPPAAEA

51 LNGVKMELID AAFPLLKGIV ATTDPVEACS GVNIAVMVGG FPRKEGMERK

101 DVMSKNVSIY KSQASALEQH AAPNCKVLVV ANPANTNALI LKEFAPSIPE

151 KNITCLTRLD HNRALGQISE RLQVPVSDVK NVIIWGNHSS SQYPDVYHAS

201 VVTGAGEKAV RQLVADDAWL DGEFITTVQQ RGAAIIKARK LSSALSAASS

251 ACDHIRDWVL GTPKGTWVSM GVYSDGSYDV PPGIIYSYPV TCENGSWSIV

301 QGLPINEFSR KKLDATANEL VEEKALAYSC LI

Query	Observed	Mr(expt)	Mr(calc)	ppm	Miss	Score	Expect	Rank	Unique	Peptide
2	873.4959	872.4887	872.4716	19.6	0	26	10	3		R.ALGQISER.L
13	1611.7779	1610.7707	1610.7471	14.6	0	72	0.00015	1	U	K.SQASALEQHAAPNCK.V
15	1875.9495	1874.9422	1874.9560	-7.36	1	119	2.7e-09	1		K.EFAPSIPEKNITCLTR.L

Database 1

Match to: Unigene1938_SeCKS transcribed RNA sequence Mass: 40614 Score: 265 Matches: 4(3) Sequences: 4(3)

gi| 11133601 Malate dehydrogenase, cytoplasmic [*Beta vulgaris*]

Matched peptides shown in **bold red**.

SPLINTVSIVLSHLHKTSHFYSHSTSLIFSLSQYSPQINHRKMAKEPLRVLVTGAAGQIGYALVPMIARGVMLGVDQPVILHMLDI
PPAAEALNGVKMELVDAAFPLLKGVVATTDAAEACKGVNVAVMVGGFPRKEGMERKDVMAKNVSIYK**SQASALEQHAAPN**
CKVLVVANPANTNALILK**EFAPSIPEQNITCLTR**LDHNR**ALGQISER**LNVQVCDVKNVIIWGNHSSSQYPDVNHASVKTESGDK
SVRELVADDAWLNGEFISTVQQRGAAIIKARKLSSALSAASSACDHIRDWVLGTPEGTWVSMGVYSDGSYNVPAGVIYSFPVT
CKDGEWK**IVQGLPIDDLSR**QKMDATGAELVDEKALAYSCLA

Query	Observed	Mr(expt)	Mr(calc)	ppm	Miss	Score	Expect	Rank	Unique	Peptide
2	873.4959	872.4887	872.4716	19.6	0	26	0.21	1	U	R.ALGQISER.L
9	1325.7661	1324.7588	1324.7351	18.0	0	48	0.00049	1	U	K.IVQGLPIDDLSR.Q
13	1611.7779	1610.7707	1610.7471	14.6	0	72	2.8e-006	1	U	K.SQASALEQHAAPNCK.V
15	1875.9495	1874.9422	1874.9196	12.0	0	119	5.5e-011	1	U	K.EFAPSIPEQNITCLTR.L

Proteins matching the same set of peptides:

Unigene12501_Se200S transcribed RNA sequence Mass: 38774 Score: 265 Matches: 4(3) Sequences: 4(3)

gi|11133601 Malate dehydrogenase, cytoplasmic [*Beta vulgaris*]

Unigene19765_Se200S transcribed RNA sequence Mass: 38774 Score: 265 Matches: 4(3) Sequences: 4(3)

gi|11133601 Malate dehydrogenase, cytoplasmic [*Beta vulgaris*]

Spot 1373

Database 1

Match to: Unigene56443_Se200S transcribed RNA sequence Mass: 36955 Score: 315 Matches: 6(4) Sequences: 5(3)

gi| 255543140 pyruvate dehydrogenase, putative [*Ricinus communis*]

Matched peptides shown in **bold red**.

LIIATTLVTTTVFLLLKKKQTDSEESEFFTNSPSSSSSSCKYKGNMMGILRQKLASGGFSQSLQHIRPAVSALRSYSSSGKEMTVR
DALNSALDEEMAADPK**VFMIGEEIGEYQGAYK**ITRGLLDKYGPQRVVDTPITESGFTGMGVGAAYYGLKPIVEFMTFNFAMQ
AIDHIINSAAKTCYMSAGQISVPIVFRGPNGAAAGVGAQHSQCYAPWYGACPGLK**VLSPYSSEDSR**GLLKAAIRDPDPVIFLEN
ELLYGESFPVSDEVLDPNFCLPIGKAKIEREGKDLTIVAFSKMVGHSLKAADILAEEGISVEVVNLR**SIRPLDRATINDSVR**KTSR
L

Query	Observed	Mr(expt)	Mr(calc)	ppm	Miss	Score	Expect	Rank	Unique	Peptide
2	856.4862	855.4789	855.4926	-16.07	0	11	3.1	7	U	R.SIRPLDR.A
3	875.4506	874.4433	874.4508	-8.58	0	5	26	4	U	R.ATINDSVR.K
11	1239.5804	1238.5731	1238.5779	-3.88	0	54	0.00017	1	U	K.VLSPYSSEDSR.G
21	1833.8569	1832.8496	1832.8655	-8.63	0	(32)	0.025	1	U	K.VFMIGEEIGEYQGAYK.I
23	1849.8496	1848.8424	1848.8604	-9.75	0	76	8.2e-007	1	U	K.VFMIGEEIGEYQGAYK.I + Oxidation (M)
24	1898.0124	1897.0051	1897.0156	-5.55	0	169	5e-016	1	U	K.AADILAEEGISVEVVNLR.S

Proteins matching the same set of peptides:

gi|116787572 Mass: 35869 Score: 218 Matches: 3(2) Sequences: 3(2)

unknown [*Picea sitchensis*]

gi|116788082 Mass: 41695 Score: 218 Matches: 3(2) Sequences: 3(2)

unknown [*Picea sitchensis*]

gi|224284403 Mass: 35838 Score: 218 Matches: 3(2) Sequences: 3(2)

unknown [*Picea sitchensis*]

2. gi|460404529 Mass: 35703 Score: 218 Matches: 3(2) Sequences: 3(2)

PREDICTED: malate dehydrogenase, cytoplasmic-like [*Solanum lycopersicum*]

Query	Observed	Mr(expt)	Mr(calc)	ppm	Miss	Score	Expect	Rank	Unique	Peptide
2	873.4959	872.4887	872.4716	19.6	0	26	10	3		R.ALGQISER.L
13	1611.7779	1610.7707	1610.7834	-7.94	1	72	0.00015	1	U	K.SQASALEKHAAPNCK.V
15	1875.9495	1874.9422	1874.9560	-7.36	1	119	2.7e-09	1		K.EFAPSIPEKNITCLTR.L

Proteins matching the same set of peptides:

gi|568214531 Mass: 35805 Score: 218 Matches: 3(2) Sequences: 3(2)

malate dehydrogenase-like protein [*Solanum tuberosum*]

Database 2

Match to: Unigene52396_SALfmcTARAAPEI-3 Mass: 29441 Score: 256 Matches: 7(4) Sequences: 6(3)

gi| 502100181 PREDICTED: pyruvate dehydrogenase E1 component subunit beta, mitochondrial-like [*Cicer arietinum*]

Matched peptides shown in **bold red**.

VRDALNSALDEEMAADPK**VFMIGEEIGEYQGAYK**ITRGLLDKYGPQRVVDTPITESGFTGMGVGAAYYGLKPIVEFMTFNFA

MQAIDHIINSAAKTCYMSAGQISVPIVFRGPNGAAAGVGAQHSQCFAPWYGACPGLK**VLSPYSSEDSR**GLLKAAIRDPDPVIFL

ENELLYGESFPVSDEVLDPNFCLPIGKAKIEREGK**DLTIVAFSR**MVGHALK**AADILAEEGISVEVVNLRSIRPLDRATINDSVR**KT

SRLVTVEEGFPQHGVGAEI

Query	Observed	Mr(expt)	Mr(calc)	ppm	Miss	Score	Expect	Rank	Unique	Peptide
2	856.4862	855.4789	855.4926	-16.07	0	11	1.8	3	U	R.SIRPLDR.A
3	875.4506	874.4433	874.4508	-8.58	0	5	13	5	U	R.ATINDSVR.K
6	1021.5629	1020.5556	1020.5604	-4.71	0	20	0.29	1	U	K.DLTIVAFSR.M
11	1239.5804	1238.5731	1238.5779	-3.88	0	54	9.1e-005	1	U	K.VLSPYSSEDSR.G
21	1833.8569	1832.8496	1832.8655	-8.63	0	(32)	0.014	1	U	K.VFMIGEEIGEYQGAYK.I
23	1849.8496	1848.8424	1848.8604	-9.75	0	76	4.7e-007	1	U	K.VFMIGEEIGEYQGAYK.I + Oxidation (M)
24	1898.0124	1897.0051	1897.0156	-5.55	0	169	2.7e-016	1	U	K.AADILAEEGISVEVVNLR.S

Spot 1438

NCBInr protein database

Match to: gi|565402905 Mass: 40724 Score: 267 Matches: 6(5) Sequences: 4(3)

PREDICTED: pyruvate dehydrogenase E1 component subunit beta-1, mitochondrial-like [*Solanum tuberosum*]

Matched peptides shown in **bold red**.

1 MSGIINRKMATRGISALNMNQFLLGSRVFASRNFSSATKEMTVRDALNSA

51 LDEEMSADPK**VFIMGEEVGEYQGAYK**ITKGLLNKYGPQRVLDTPITEAGF

101 TGMGVGAAYH GLKPVIEFMTFNFSMQAIDHIINSAAK**SNYMSAGQISVPI**

151 **VFR**GPNGAAAGVGAQHSQCY AAWFGACPGL K**VLAPYSSEDAR**GLLKAAIR

201 DPDPVVFLENELLYGESFPVSAEVLDSSFSLPIGKAKIEREGKDITITAF

251 SKMVGYALKA AEILAKEGISAEVINLRSIRPLDRSTINASVRKTNRLLTV

301 EEGFPQHGVGAEICASVVEESFEYLDAPVERISGADVPMP YAANLER**MAV**

351 **PQIEDIVR**GAKRACYRAAKSVPMAATA

Query	Observed	Mr(expt)	Mr(calc)	ppm	Miss	Score	Expect	Rank	Unique	Peptide
6	1207.6226	1206.6153	1206.5880	22.6	0	32	2	2		K.VLAPYSSEDAR.G
9	1270.7157	1269.7084	1269.6751	26.2	0	(55)	0.0073	1		R.MAVPQIEDIVR.G
11	1286.7303	1285.7230	1285.6700	41.2	0	56	0.0073	1		R.MAVPQIEDIVR.G +

Oxidation (M)

21 1784.9417 1783.9344 1783.8927 23.4 0 61 0.0019 1 K.SNYMSAGQISVPIVFR.G +

Oxidation (M)

22 1819.8952 1818.8880 1818.8498 21.0 0 (67) 0.00047 1 U K.VFIMGEEVGEYQGAYK.I

23 1835.8845 1834.8772 1834.8447 17.7 0 118 3.2e-09 1 U K.VFIMGEEVGEYQGAYK.I +

Oxidation (M)

2. gi|225425166 Mass: 39749 Score: 235 Matches: 5(5) Sequences: 3(3)

PREDICTED: pyruvate dehydrogenase E1 component subunit beta, mitochondrial-like isoform 1 [*Vitis vinifera]*

Query Observed Mr(expt) Mr(calc) ppm Miss Score Expect Rank Unique Peptide

9 1270.7157 1269.7084 1269.6751 26.2 0 (55) 0.0072 1 R.MAVPQIEDIVR.A

11 1286.7303 1285.7230 1285.6700 41.2 0 56 0.0073 1 R.MAVPQIEDIVR.A + Oxidation (M)

21 1784.9417 1783.9344 1783.8927 23.4 0 61 0.0019 1 K.SNYMSAGQISVPIVFR.G +

Oxidation (M)

22 1819.8952 1818.8880 1818.8498 21.0 0 (67) 0.00047 1 K.VFLMGEEVGEYQGAYK.I

23 1835.8845 1834.8772 1834.8447 17.7 0 118 3.2e-09 1 K.VFLMGEEVGEYQGAYK.I +

Oxidation (M)

Proteins matching the same set of peptides:

gi|296088722 Mass: 46703 Score: 235 Matches: 5(5) Sequences: 3(3)

unnamed protein product [*Vitis vinifera*]

gi|593331338 Mass: 39319 Score: 235 Matches: 5(5) Sequences: 3(3)

hypothetical protein PHAVU_008G000800g [*Phaseolus vulgaris*]

3. gi|224053535 Mass: 38846 Score: 211 Matches: 4(3) Sequences: 3(2)

pyruvate dehydrogenase family protein [Populus trichocarpa]

Query Observed Mr(expt) Mr(calc) ppm Miss Score Expect Rank Unique Peptide

6 1207.6226 1206.6153 1206.5880 22.6 0 32 2 2 K.VLAPYSSEDAR.G

21 1784.9417 1783.9344 1783.8927 23.4 0 61 0.0019 1 K.SNYMSAGQISVPIVFR.G +

Oxidation (M)

22 1819.8952 1818.8880 1818.8498 21.0 0 (67) 0.00047 1 K.VFLMGEEVGEYQGAYK.I

23 1835.8845 1834.8772 1834.8447 17.7 0 118 3.2e-09 1 K.VFLMGEEVGEYQGAYK.I +

Oxidation (M)

Proteins matching the same set of peptides:

gi|502100181 Mass: 39106 Score: 211 Matches: 4(3) Sequences: 3(2)

PREDICTED: pyruvate dehydrogenase E1 component subunit beta, mitochondrial-like [*Cicer arietinum*]

4. gi|566163022 Mass: 40230 Score: 181 Matches: 4(2) Sequences: 3(1)

pyruvate dehydrogenase family protein [*Populus trichocarpa*]

Query Observed Mr(expt) Mr(calc) ppm Miss Score Expect Rank Unique Peptide

6 1207.6226 1206.6153 1206.5880 22.6 0 32 2 2 K.VLAPYSSEDAR.G

21 1784.9417 1783.9344 1783.8927 23.4 0 30 2 2 U K.SNYMSSGQISVPIVFR.G

22 1819.8952 1818.8880 1818.8498 21.0 0 (67) 0.00047 1 K.VFLMGEEVGEYQGAYK.I

23 1835.8845 1834.8772 1834.8447 17.7 0 118 3.2e-09 1 K.VFLMGEEVGEYQGAYK.I +

Oxidation (M)

5. gi|565395264 Mass: 40643 Score: 147 Matches: 4(2) Sequences: 2(1)

PREDICTED: pyruvate dehydrogenase E1 component subunit beta-1, mitochondrial-like [*Solanum tuberosum*]

Query	Observed	Mr(expt)	Mr(calc)	ppm	Miss	Score	Expect	Rank	Unique	Peptide
9	1270.7157	1269.7084	1269.6751	26.2	0	(25)	7.1	2	U	R.MALPQVEDIVR.A
11	1286.7303	1285.7230	1285.6700	41.2	0	30	2.8	2	U	R.MALPQVEDIVR.A + Oxidation (M)
22	1819.8952	1818.8880	1818.8498	21.0	0	(67)	0.00047	1		R.VFLMGEEVGEYQGAYK.V
23	1835.8845	1834.8772	1834.8447	17.7	0	118	3.2e-09	1		R.VFLMGEEVGEYQGAYK.V + Oxidation (M)

Database 1

1. Match to: Unigene1913_Se200S transcribed RNA sequence Mass: 17310 Score: 376 Matches: 6(5) Sequences: 5(4)

gi| 297792391pyruvate dehydrogenase E1 component beta subunit, mitochondrial [*Arabidopsis lyrata subsp. lyrata*]

Matched peptides shown in bold red.

FDRVVVVEVEEERETMSWLVKQVVNSGRLQSSSRLRVLQLAARAYATSGKEMTVRDALNSAIDEEMGADRKVFLMGEEVGEY
QGAYKITKGLLEKYGPERVIDTPITEAGFAGIGVGAAYKDLKPIIEFMTFNFSMQAIDHIINSAAKSNYMSAGQIS

Query	Observed	Mr(expt)	Mr(calc)	ppm	Miss	Score	Expect	Rank	Unique	Peptide
14	1622.7624	1621.7551	1621.6890	40.8	0	53	0.0002	1	U	R.DALNSAIDEEMGADR.K + Oxidation (M)
19	1750.8626	1749.8553	1749.7839	40.8	1	24	0.2	1	U	R.DALNSAIDEEMGADRK.V + Oxidation (M)
22	1819.8952	1818.8880	1818.8498	21.0	0	(67)	9.8e-006	1	U	K.VFLMGEEVGEYQGAYK.I
23	1835.8845	1834.8772	1834.8447	17.7	0	118	5.9e-011	1	U	K.VFLMGEEVGEYQGAYK.I + Oxidation (M)
27	1963.9808	1962.9735	1962.9397	17.2	1	49	0.00059	1	U	R.KVFLMGEEVGEYQGAYK.I + Oxidation (M)
28	2050.1080	2049.1007	2049.0783	11.0	0	131	2.2e-012	1	U	R.VIDTPITEAGFAGIGVGAAYK.D

2. Unigene5467_Se200S transcribed RNA sequence Mass: 15863 Score: 299 Matches: 7(4) Sequences: 5(3)

gi| 604344240 hypothetical protein MIMGU_mgv1a008776mg [*Erythranthe guttata*]

Query	Observed	Mr(expt)	Mr(calc)	ppm	Miss	Score	Expect	Rank	Unique	Peptide
2	856.5215	855.5142	855.4926	25.3	0	11	3.1	8	U	R.SIRPLDR.S
3	905.4855	904.4782	904.4614	18.6	0	15	2.8	2	U	R.STINESVR.K
9	1270.7157	1269.7084	1269.6751	26.2	0	(55)	0.00012	1	U	K.MAVPQIEDIVR.A
11	1286.7303	1285.7230	1285.6700	41.2	0	56	0.00013	1	U	K.MAVPQIEDIVR.A + Oxidation (M)
15	1723.8722	1722.8650	1722.8287	21.1	0	(18)	0.76	1	U	R.ISGADIPMPYAANFEK.M
18	1739.8613	1738.8540	1738.8236	17.5	0	42	0.0031	1	U	R.ISGADIPMPYAANFEK.M + Oxidation (M)
25	1884.0482	1883.0409	1883.0000	21.8	0	176	7.1e-017	1	U	K.AAEILAEDGISAEVINLR.S

Proteins matching the same set of peptides:

Unigene18769_SeCKS transcribed RNA sequence Mass: 15176 Score: 299 Matches: 7(4) Sequences: 5(3)

gi| 604344240 hypothetical protein MIMGU_mgv1a008776mg [*Erythranthe guttata*]

Database 2

Match to: Unigene56875_SALfmcTARAAPEI-3 Mass: 38468 Score: 803 Matches: 15(11) Sequences: 12(9)

gi| 449445580 PREDICTED: pyruvate dehydrogenase E1 component subunit beta, mitochondrial-like [*Cucumis sativus*]

Matched peptides shown in bold red.

GRLQSSSRLRALQLAARAYATSGKEMTVRDALNSAIDEEMGADRKVFLMGEEVGEYQGAYKITKGLLEKYGPERVIDTPITEA
GFAGIGVGAAYKDLKPIIEFMTFNFSMQAIDHIINSAAKSNYMSAGQISVPIVFRGPNGAAAGVGAQHSQCYAAWYGSCPGLK
VLSPYSAEDARGLLKAAIRDPDPVVFLENEILYGESFPISEEVLDPNFTLPIGKAKIERQGKDVTITAFSKMVGYALKAAEILAED
GISAEVINLRSIRPLDRSTINESVRKTTRLVTLEEGFPQHGVGAEICTSVVEDSFWYLDSPIERISGADIPMPYAANFEKMAVPQIE
DIVRAAKRACYRS

Query	Observed	Mr(expt)	Mr(calc)	ppm	Miss	Score	Expect	Rank	Unique	Peptide
2	856.5215	855.5142	855.4926	25.3	0	11	1.5	6	U	R.SIRPLDR.S
3	905.4855	904.4782	904.4614	18.6	0	15	1.3	1	U	R.STINESVR.K
6	1207.6226	1206.6153	1206.5880	22.6	0	68	5.5e-006	1	U	K.VLSPYSAEDAR.G
9	1270.7157	1269.7084	1269.6751	26.2	0	(55)	6.6e-005	1	U	K.MAVPQIEDIVR.A
11	1286.7303	1285.7230	1285.6700	41.2	0	56	6.7e-005	1	U	K.MAVPQIEDIVR.A + Oxidation (M)
14	1622.7624	1621.7551	1621.6890	40.8	0	53	0.0001	1	U	R.DALNSAIDEEMGADR.K + Oxidation (M)
15	1723.8722	1722.8650	1722.8287	21.1	0	(18)	0.35	1	U	R.ISGADIPMPYAANFEK.M
18	1739.8613	1738.8540	1738.8236	17.5	0	42	0.0014	1	U	R.ISGADIPMPYAANFEK.M + Oxidation (M)
19	1750.8626	1749.8553	1749.7839	40.8	1	24	0.082	1	U	R.DALNSAIDEEMGADRK.V + Oxidation (M)
21	1784.9417	1783.9344	1783.8927	23.4	0	61	1.8e-005	1	U	K.SNYMSAGQISVPIVFR.G + Oxidation (M)
22	1819.8952	1818.8880	1818.8498	21.0	0	(67)	4.6e-006	1	U	K.VFLMGEEVGEYQGAYK.I
23	1835.8845	1834.8772	1834.8447	17.7	0	118	2.7e-011	1	U	K.VFLMGEEVGEYQGAYK.I + Oxidation (M)
25	1884.0482	1883.0409	1883.0000	21.8	0	176	4.2e-017	1	U	K.AAEILAEDGISAEVINLR.S
27	1963.9808	1962.9735	1962.9397	17.2	1	49	0.00025	1	U	R.KVFLMGEEVGEYQGAYK.I + Oxidation (M)
28	2050.1080	2049.1007	2049.0783	11.0	0	131	1.2e-012	1	U	R.VIDTPITEAGFAGIGVGAAYK.D

Spot 1623

NCBInr protein database

Match to: gi|595820799 Mass: 29561 Score: 227 Matches: 5(2) Sequences: 4(2)

hypothetical protein PRUPE_ppa009928mg [*Prunus persica*]

Matched peptides shown in bold red.

1 MGTLGRAIYT VGFWIRETGQ AIDRLGSRLQ GNYYFQEQLS RHRTLMNVFD

51 KAPVVDKDAF VAPSASIVGQ VQVGRGSSIW YGCVLRGDVN SISIGSGTNI

101 QDNSLVHVAK SNLSGKVLPT VIGDNVTVGH SAVLHGCTVE DEAFVGMAAT

151 LLDGVYVEKH AMVAAGALVR QNTRIPCGEV WGGNPAKFLR KLTEEEMAFI

201 SQSAINYSNL AQAHAAENAK SLDEIEFEKV LRKKFARRDE EYDSMLGIVR

251 ETPAEITLPD NVLPGKVPKT A

Query	Observed	Mr(expt)	Mr(calc)	ppm	Miss	Score	Expect	Rank	Unique	Peptide
12	1095.6450	1094.6377	1094.6019	32.8	0	59	0.0027	1	U	K.HAMVAAGALVR.Q
13	1111.6458	1110.6385	1110.5968	37.6	0	(34)	0.95	1	U	K.HAMVAAGALVR.Q +

Oxidation (M)

22	1225.7080	1224.7007	1224.6655	28.7	0	29	3	1		R.AIYTVGFWIR.E
38	1598.8307	1597.8234	1597.7406	51.8	1	29	3.2	1	U	R.RDEEYDSMLGIVR.E + Oxidation (M)
39	1645.8508	1644.8435	1644.7896	32.8	0	110	2.7e-08	1		R.LQGNYYFQEQLSR.H

Database 1

Match to: Unigene2906_Se200S transcribed RNA sequence   Mass: 25523   Score: 602   Matches: 7(7)   Sequences: 5(5)

gi| 590593478 Gamma carbonic anhydrase 1, CA1 [*Theobroma cacao*]

Matched peptides shown in bold red.

QLSRHRTLMNVFDKKPTVDKEAFVAPSASVIGDVQVGHGSSIWYGCVLRGDVNNISIGSGTNIQDNSLVHVAKTNMAGKVLPT

IVGDNVTIGHSAILHGCTVDDEAFVGMGATLLDGVVVEKHAMVAAGALVRQNTRIPSGQVWGGNPAKFLRNLTEEEIAFIAES

ATNYCNLAKVHAAENDKGFDEIEFEKVLRKKYARKDEEYDSMIGIVRETPPELILPDNILPDKAAQKSS

Query	Observed	Mr(expt)	Mr(calc)	ppm	Miss	Score	Expect	Rank	Unique	Peptide
12	1095.6450	1094.6377	1094.6019	32.8	0	59	3.4e-005	1	U	K.HAMVAAGALVR.Q
13	1111.6458	1110.6385	1110.5968	37.6	0	(34)	0.014	1	U	K.HAMVAAGALVR.Q + Oxidation (M)
26	1310.7327	1309.7254	1309.6779	36.3	0	98	7.3e-009	1	U	R.IPSGQVWGGNPAK.F
35	1554.8091	1553.8018	1553.7395	40.1	1	109	7.1e-010	1	U	R.KDEEYDSMIGIVR.E
36	1570.8107	1569.8034	1569.7345	43.9	1	(76)	1.3e-006	1	U	R.KDEEYDSMIGIVR.E + Oxidation (M)
44	1978.0058	1976.9985	1976.9116	44.0	1	154	1.9e-014	1	U	K.VHAAENDKGFDEIEFEK.V
56	2452.3492	2451.3419	2451.2354	43.5	0	183	1.1e-017	1	U	R.GDVNNISIGSGTNIQDNSLVHVAK.T

Proteins matching the same set of peptides:

Unigene32001_SeCKS transcribed RNA sequence   Mass: 19073   Score: 457   Matches: 6(5)   Sequences: 5(4)

gi| 590593478 Gamma carbonic anhydrase 1, CA1 [*Theobroma cacao*]

Database 2

Match to: Unigene26040_SALfmcTARAAPEI-3   Mass: 8568   Score: 140   Matches: 3(3)   Sequences: 2(2)

gi| 590593478 Gamma carbonic anhydrase 1, CA1 [*Theobroma cacao*]

Matched peptides shown in bold red.

CTVDDEAFVGMGATLLDGVVVEKHAMVAAGALVRQNTRIPSGQVWGGNPAKFLRNLTEEEIAFIAESATNYCNLAKVHAA

Query	Observed	Mr(expt)	Mr(calc)	ppm	Miss	Score	Expect	Rank	Unique	Peptide
12	1095.6450	1094.6377	1094.6019	32.8	0	59	2.4e-005	1	U	K.HAMVAAGALVR.Q
13	1111.6458	1110.6385	1110.5968	37.6	0	(34)	0.0085	1	U	K.HAMVAAGALVR.Q + Oxidation (M)
26	1310.7327	1309.7254	1309.6779	36.3	0	98	3.8e-009	1	U	R.IPSGQVWGGNPAK.F

Spot 1701

NCBInr protein database

Match to: gi|449457524   Mass: 29725   Score: 119   Matches: 2(1)   Sequences: 2(1)

PREDICTED: gamma carbonic anhydrase 1 [*Cucumis sativus*]

Matched peptides shown in bold red.

1 MGTLGKAIYT VGFWIRETGQ ALDRLGCRLQ GNYYFQEQLS RHRTLMNIFD

51 KAPVVDKDAF VAPSASIIGD VQVGRGSSIW YGCVLRGDVN SISVGSGTNI

101 QDNSLVHVAK SNLSGKVLPT IIGDNVTVGH SAVLHGCTIE DEAFVGMGAT

151 LLDGVYVEKH AMVAAGALVR QNTRVPCGEV WGGNPAKFLR KLTEEEMVFI

201 SQSAINYSNL SQVHAAENVK SFDEIELEKV LRKKFARRDE DYDSMLGVVR

251 ETPPELVLPD NILADKVAKS S

Query	Observed	Mr(expt)	Mr(calc)	ppm	Miss	Score	Expect	Rank	Unique	Peptide
8	1225.6699	1224.6626	1224.6655	-2.37	0	12	1.9e+02	2		R.AIYTVGFWIR.E
18	1645.7840	1644.7767	1644.7896	-7.83	0	107	5.4e-08	1	U	R.LQGNYYFQEQLSR.H

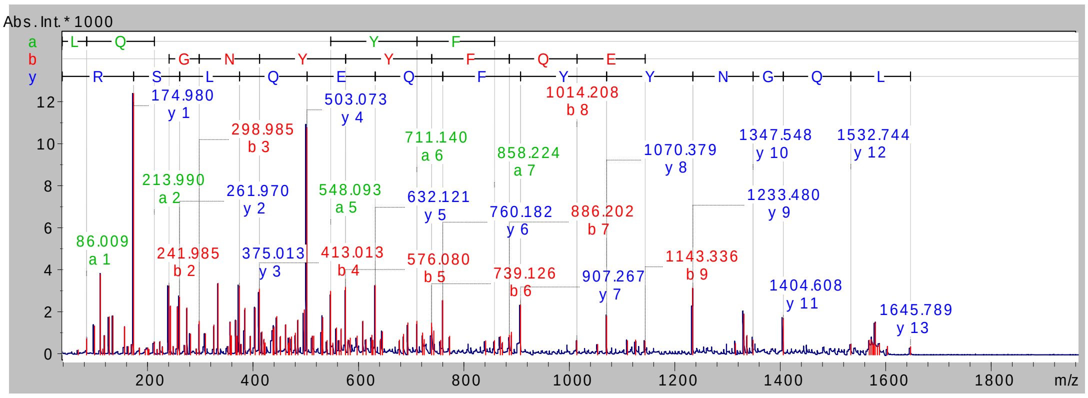

Proteins matching the same set of peptides:

gi|449457524 Mass: 29725 Score: 119 Matches: 2(1) Sequences: 2(1)

PREDICTED: gamma carbonic anhydrase 1, mitochondrial-like [*Cucumis sativus*]

gi|449499995 Mass: 29801 Score: 119 Matches: 2(1) Sequences: 2(1)

PREDICTED: gamma carbonic anhydrase 1, mitochondrial-like [*Cucumis sativus*]

gi|566156054 Mass: 31252 Score: 119 Matches: 2(1) Sequences: 2(1)

hypothetical protein POPTR_0002s03550g [*Populus trichocarpa*]

gi|586644788 Mass: 29874 Score: 119 Matches: 2(1) Sequences: 2(1)

hypothetical protein AMTR_s00011p00224870 [*Amborella trichopoda*]

gi|587903703 Mass: 29629 Score: 119 Matches: 2(1) Sequences: 2(1)

hypothetical protein L484_009014 [Morus notabilis]

gi|595820791 Mass: 20436 Score: 119 Matches: 2(1) Sequences: 2(1)

hypothetical protein PRUPE_ppa009928mg [*Prunus persica*]

gi|595820799 Mass: 29561 Score: 119 Matches: 2(1) Sequences: 2(1)

hypothetical protein PRUPE_ppa009928mg [*Prunus persica*]

Database 1

Match to: Unigene2906_Se200S transcribed RNA sequence Mass: 25523 Score: 431 Matches: 5(5) Sequences: 4(4)

gi| 590593478 Gamma carbonic anhydrase 1, CA1 [*Theobroma cacao*]

Matched peptides shown in bold red.

QLSRHRTLMNVFDKKPTVDKEAFVAPSASVIGDVQVGHGSSIWYGCVLRGDVNNISIGSGTNIQDNSLVHVAKTNMAGKVLPT

IVGDNVTIGHSAILHGCTVDDEAFVGMGATLLDGVVVEKHAMVAAGALVRQNTRIPSGQVWGGNPAKFLRNLTEEEIAFIAES

ATNYCNLAKVHAAENDKGFDEIEFEKVLRKKYARKDEEYDSMIGIVRETPPELILPDNILPDKAAQKSS

Query	Observed	Mr(expt)	Mr(calc)	ppm	Miss	Score	Expect	Rank	Unique	Peptide
11	1310.6734	1309.6661	1309.6779	-9.01	0	93	2.7e-008	1	U	R.IPSGQVWGGNPAK.F
16	1554.7406	1553.7333	1553.7395	-3.99	1	(66)	1.3e-005	1	U	R.KDEEYDSMIGIVR.E
17	1570.7273	1569.7201	1569.7345	-9.17	1	75	1.3e-006	1	U	R.KDEEYDSMIGIVR.E + Oxidation (M)

20	1803.9766	1802.9693	1802.9666	1.52	0	105	1.4e-009	1	U	R.ETPPELILPDNILPDK.A
21	1977.9291	1976.9219	1976.9116	5.21	1	157	6.7e-015	1	U	K.VHAAENDKGFDEIEFEK.V

Proteins matching the same set of peptides:

Unigene32001_SeCKS transcribed RNA sequence Mass: 19073 Score: 431 Matches: 5(5) Sequences: 4(4)

gi| 590593478 Gamma carbonic anhydrase 1, CA1 [*Theobroma cacao*]

Spot 1742

NCBInr protein database

Match to: gi|118488068 Mass: 27673 Score: 172 Matches: 3(1) Sequences: 3(1)

unknown [*Populus trichocarpa*]

Matched peptides shown in bold red.

1 MATTLARIAR KSLTTTTAFS LNRHFGTEAV AAATASTKSI TPSADRVKWD

51 YRGQRQIIPL GQWLPKVAVD AYVAPNVVLA GQVTVYDGAS VWNGAVLRGD

101 LNKITVGFCS NVQERCVVHA AWNSPTGLPA ETSIERYVTI GAYSLLRSCT

151 IEPECIIGQH SILMEGSLVE THSILEAGSV VPPGRRIPTG ELWAGNPARF

201 VRTLTHEETL EIPKLAVAIN DLSKTHFFEF LPYSTVYLEV EKLKKKLEIK

251 V

Query	Observed	Mr(expt)	Mr(calc)	ppm	Miss	Score	Expect	Rank	Unique	Peptide
13	1381.7552	1380.7480	1380.7150	23.9	0	91	2.3e-06	1		R.IPTGELWAGNPAR.F
16	1409.7251	1408.7178	1408.6769	29.0	0	46	0.066	1		K.ITVGFCSNVQER.C
21	1537.8548	1536.8475	1536.8161	20.5	1	35	0.69	1		R.RIPTGELWAGNPAR.F

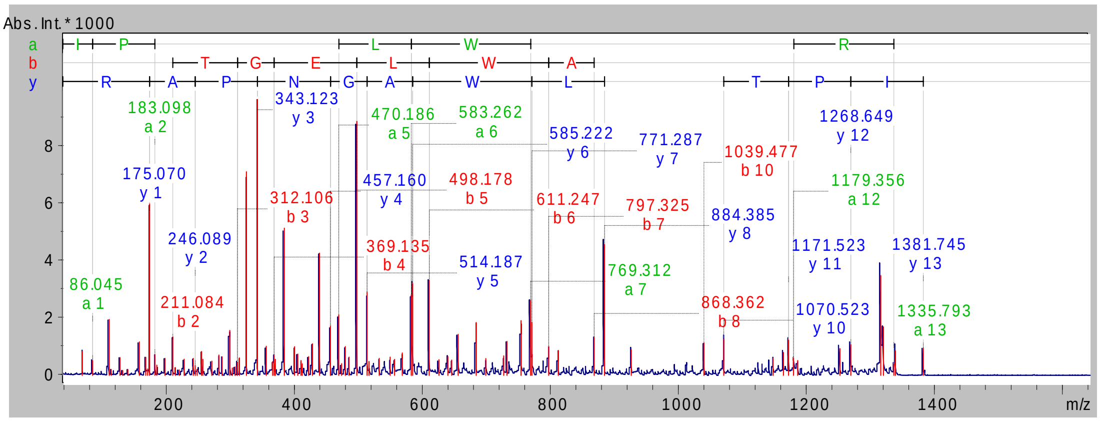

Proteins matching the same set of peptides:

gi|255540193 Mass: 27825 Score: 172 Matches: 3(1) Sequences: 3(1)

Protein yrdA, putative [*Ricinus communis*]

gi|567888678 Mass: 28350 Score: 172 Matches: 3(1) Sequences: 3(1)

hypothetical protein CICLE_v10032492mg [*Citrus clementina*]

gi|587834461 Mass: 28251 Score: 172 Matches: 3(1) Sequences: 3(1)

hypothetical protein L484_010119 [*Morus notabilis*]

gi|590670270 Mass: 40864 Score: 172 Matches: 3(1) Sequences: 3(1)

Gamma carbonic anhydrase like 1, CAL1 isoform 1 [*Theobroma cacao*]

gi|590670273 Mass: 38021 Score: 172 Matches: 3(1) Sequences: 3(1)

Gamma carbonic anhydrase like 1, CAL1 isoform 2, partial [*Theobroma cacao*]

gi|595848384 Mass: 27298 Score: 172 Matches: 3(1) Sequences: 3(1)

hypothetical protein PRUPE_ppa010461mg [*Prunus persica*]

Database 1

Match to: Unigene46277_Se200S transcribed RNA sequence Mass: 17969 Score: 159 Matches: 3(3) Sequences: 3(3)

gi| 449520511 PREDICTED: gamma carbonic anhydrase-like 2, mitochondrial-like [*Cucumis sativus*]

Matched peptides shown in **bold red.**

RGQRLIIPLGQWNPKIAVDAYVAPNVVLAGQVNVCDGSSVWNGSVLRGDLNKITVGFCSNVQEKCVLHAAWSSPTGLPAETSI

DR**FVSVGANCLLR**SCTIEPECIVGQHSILMEGSLMETHSILEAGSVVPPGR**RIPTGELWAGNPAR**FVRTLTHEETLEIPKL

Query	Observed	Mr(expt)	Mr(calc)	ppm	Miss	Score	Expect	Rank	Unique	Peptide
9	1235.6846	1234.6773	1234.6492	22.7	0	33	0.022	1	U	R.FVSVGANCLLR.S
13	1381.7552	1380.7480	1380.7150	23.9	0	91	4e-008	1	U	R.IPTGELWAGNPAR.F
21	1537.8548	1536.8475	1536.8161	20.5	1	35	0.012	1	U	R.RIPTGELWAGNPAR.F

Database 2

Match to: Unigene51783_SALfmcTARAAPEI-3 Mass: 17032 Score: 187 Matches: 5(5) Sequences: 5(5)

gi| 449520511 PREDICTED: gamma carbonic anhydrase-like 2, mitochondrial-like [*Cucumis sativus*]

Matched peptides shown in **bold red.**

DLNK**ITVGFCSNVQERCVLHAAWSSPTGLPAETSVDRFVSVGANCLLR**SCTIEPECIVGQHSILMEGSLMETHSILEAGSVVPPG

R**RIPTGELWAGNPAR**FVRTLTHEETLEIPKLAVAINDLSKSYFSEFLPYSTVYLEVEKLKKKLEINI

Query	Observed	Mr(expt)	Mr(calc)	ppm	Miss	Score	Expect	Rank	Unique	Peptide
9	1235.6846	1234.6773	1234.6492	22.7	0	33	0.012	1	U	R.FVSVGANCLLR.S
13	1381.7552	1380.7480	1380.7150	23.9	0	91	2e-008	1	U	R.IPTGELWAGNPAR.F
16	1409.7251	1408.7178	1408.6769	29.0	0	46	0.00058	1	U	K.ITVGFCSNVQER.C
21	1537.8548	1536.8475	1536.8161	20.5	1	35	0.0064	1	U	R.RIPTGELWAGNPAR.F
34	2254.1334	2253.1261	2253.0848	18.3	0	83	8.5e-008	1	U	R.CVLHAAWSSPTGLPAETSVDR.F

Spot 1766

NCBInr protein database

Match to: gi|118488068 Mass: 27673 Score: 164 Matches: 3(2) Sequences: 3(2)

unknown [*Populus trichocarpa*]

Matched peptides shown in **bold red.**

1 MATTLARIAR KSLTTTTAFS LNRHFGTEAV AAATASTKSI TPSADRVKWD

51 YRGQRQIIPL GQWLPKVAVD AYVAPNVVLA GQVTVYDGAS VWNGAVLRGD

101 LNK**ITVGFCS NVQER**CVVHA AWNSPTGLPA ETSIERYVTI GAYSLLRSCT

151 IEPECIIGQH SILMEGSLVE THSILEAGSV VPPGR**RIPTG ELWAGNPAR**F

201 VRTLTHEETL EIPKLAVAIN DLSKTHFFEF LPYSTVYLEV EKLKKKLEIK

251 V

Query	Observed	Mr(expt)	Mr(calc)	ppm	Miss	Score	Expect	Rank	Unique	Peptide
5	1381.7128	1380.7055	1380.7150	-6.85	0	91	2.5e-06	1		R.IPTGELWAGNPAR.F
7	1409.6782	1408.6710	1408.6769	-4.22	0	51	0.023	1	U	K.ITVGFCSNVQER.C
10	1537.8199	1536.8127	1536.8161	-2.23	1	23	13	1		R.RIPTGELWAGNPAR.F

Proteins matching the same set of peptides:

gi|255540193 Mass: 27825 Score: 164 Matches: 3(2) Sequences: 3(2)

Protein yrdA, putative [*Ricinus communis*]

gi|567888678 Mass: 28350 Score: 164 Matches: 3(2) Sequences: 3(2)

hypothetical protein CICLE_v10032492mg [*Citrus clementina*]

gi|587834461 Mass: 28251 Score: 164 Matches: 3(2) Sequences: 3(2)

hypothetical protein L484_010119 [*Morus notabilis*]

gi|590670270 Mass: 40864 Score: 164 Matches: 3(2) Sequences: 3(2)

Gamma carbonic anhydrase like 1, CAL1 isoform 1 [*Theobroma cacao*]

gi|590670273 Mass: 38021 Score: 164 Matches: 3(2) Sequences: 3(2)

Gamma carbonic anhydrase like 1, CAL1 isoform 2, partial [*Theobroma cacao*]

gi|595848384 Mass: 27298 Score: 164 Matches: 3(2) Sequences: 3(2)

hypothetical protein PRUPE_ppa010461mg [*Prunus persica*]

Database 1

Match to: Unigene46277_Se200S transcribed RNA sequence Mass: 17969 Score: 151 Matches: 3(2) Sequences: 3(2)

gi| 449520511 PREDICTED: gamma carbonic anhydrase-like 2, mitochondrial-like [*Cucumis sativus*]

Matched peptides shown in **bold red.**

RGQRLIIPLGQWNPKIAVDAYVAPNVVLAGQVNVCDGSSVWNGSVLRGDLNKITVGFCSNVQEKCVLHAAWSSPTGLPAETSI

DR**FVSVGANCLLR**SCTIEPECIVGQHSILMEGSLMETHSILEAGSVVPPGR**RIPTGELWAGNPAR**FVRTLTHEETLEIPKL

Query	Observed	Mr(expt)	Mr(calc)	ppm	Miss	Score	Expect	Rank	Unique	Peptide
2	1235.6480	1234.6408	1234.6492	-6.86	0	39	0.008	1	U	R.FVSVGANCLLR.S
5	1381.7128	1380.7055	1380.7150	-6.85	0	91	5.1e-008	1		R.IPTGELWAGNPAR.F
10	1537.8199	1536.8127	1536.8161	-2.23	1	23	0.25	1		R.RIPTGELWAGNPAR.F

Database 2

Match to: Unigene51783_SALfmcTARAAPEI-3 Mass: 17032 Score: 276 Matches: 6(4) Sequences: 6(4)

gi| 449520511 PREDICTED: gamma carbonic anhydrase-like 2, mitochondrial-like [*Cucumis sativus*]

Matched peptides shown in **bold red.**

DLNK**ITVGFCSNVQERCVLHAAWSSPTGLPAETSVDRFVSVGANCLLR**SCTIEPECIVGQHSILMEGSLMETHSILEAGSVVPPG

R**RIPTGELWAGNPAR**FVRTLTHEETLEIPKLAVAINDLSK**SYFSEFLPYSTVYLEVEK**LKKKLEINI

Query	Observed	Mr(expt)	Mr(calc)	ppm	Miss	Score	Expect	Rank	Unique	Peptide
2	1235.6480	1234.6408	1234.6492	-6.86	0	39	0.0038	1	U	R.FVSVGANCLLR.S
5	1381.7128	1380.7055	1380.7150	-6.85	0	91	2.1e-008	1	U	R.IPTGELWAGNPAR.F
7	1409.6782	1408.6710	1408.6769	-4.22	0	51	0.0002	1	U	K.ITVGFCSNVQER.C
10	1537.8199	1536.8127	1536.8161	-2.23	1	23	0.12	1	U	R.RIPTGELWAGNPAR.F
16	2201.0225	2200.0152	2200.0616	-21.09	0	16	0.36	1	U	K.SYFSEFLPYSTVYLEVEK.L
18	2254.0658	2253.0585	2253.0848	-11.68	0	57	3.6e-005	1	U	R.CVLHAAWSSPTGLPAETSVDR.F

Spot 571

NCBInr protein database

Match to: gi|15240075 Mass: 70240 Score: 141 Matches: 3(1) Sequences: 3(1)

succinate dehydrogenase [ubiquinone] flavoprotein subunit 1 [*Arabidopsis thaliana*]

Matched peptides shown in **bold red**.

1 MWRCVSRGFR APASKTSSLF DGVSGSRFSR FFSTGSTDTR SSYTIVDHTY

51 DAVVVGAGGA GLRAAIGLSE HGFNTACITK LFPTRSHTVA AQGGINAALG

101 NMSEDDWRWH MYDTVK**GSDW LGDQDAIQYM CR**EAPK**AVIE LENYGLPFSR**

151 TEEGKIYQRA FGGQSLDFGK GGQAYRCACA ADRTGHALLH TLYGQAMKHN

201 TQFFVEYFAL DLLMASDGSC QGVIALNMED GTLHRFRSSQ TILATGGYGR

251 AYFSATSAHT CTGDGNAMVA RAGLPLQDLE FVQFHPTGIY GAGCLITEGS

301 RGEGGILRNS EGERFMERYA PTAKDLASRD VVSRSMTMEI REGRGVGPHK

351 DHIYLHLNHL PPEVLKERLP GISETAAIFA GVDVTKEPIP VLPTVHYNMG

401 GIPTNYHGEV VTIKGDDPDA VIPGLMAAGE AACASVHGAN RLGANSLLDI

451 VVFGRACANR VAEISKPGEK QKPLEKDAGE KTIAWLDRLR NSNGSLPTST

501 IRLNMQRIMQ NNAAVFRTQE TLEEGCQLID KAWESFGDVQ VKDRSMIWNS

551 DLIETLELEN LLINASITMH SAEARKESRG AHAREDFTKR EDGEWMKHTL

601 GYWEDEKVRL DYRPVHMDTL DDEIDTFPPK ARVY

Query	Observed	Mr(expt)	Mr(calc)	ppm	Miss	Score	Expect	Rank	Unique	Peptide
15	1532.7153	1531.7081	1531.7419	-22.10	1	28	4.9	1	U	K.HTLGYWEDEKVR.L
17	1607.8060	1606.7988	1606.8355	-22.86	0	71	0.00022	1		K.AVIELENYGLPFSR.T
18	1914.7602	1913.7529	1913.8036	-26.50	0	42	0.062	1		K.GSDWLGDQDAIQYMCR.E

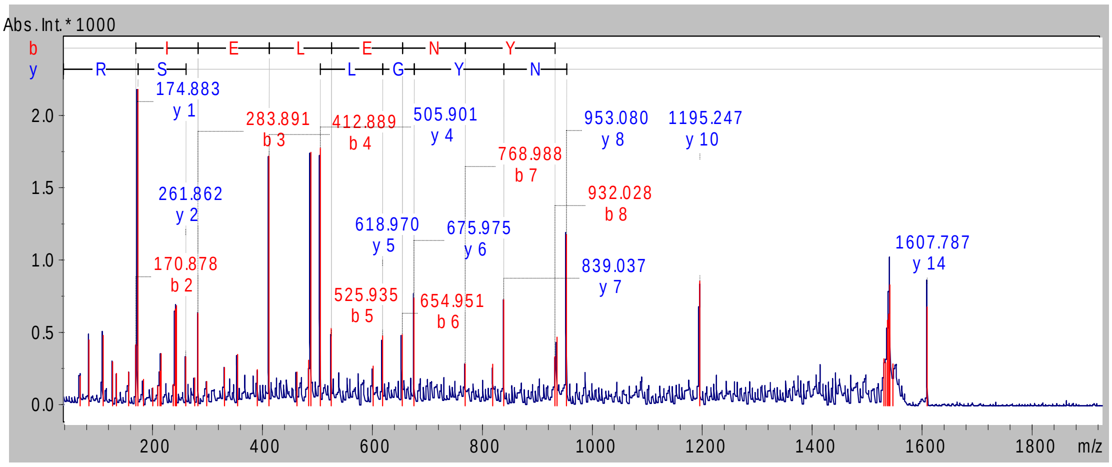

Proteins matching the same set of peptides:

gi|297797713 Mass: 70227 Score: 141 Matches: 3(1) Sequences: 3(1)

succinate dehydrogenase 1-1 [*Arabidopsis lyrata subsp. lyrata*]

gi|565431839 Mass: 76262 Score: 141 Matches: 3(1) Sequences: 3(1)

hypothetical protein CARUB_v10026004mg, partial [*Capsella rubella*]

gi|567136140 Mass: 69655 Score: 141 Matches: 3(1) Sequences: 3(1)

hypothetical protein EUTSA_v10003821mg [*Eutrema salsugineum*]

Database 1

Match to: Unigene 54839_SeCKS transcribed RNA Mass: 32308 Score: 150 Matches: 5(2) Sequences: 4(2)

gi| 502161129 PREDICTED: succinate dehydrogenase [ubiquinone] flavoprotein subunit 1, mitochondrial-like [*Cicer arietinum*]

Matched peptides shown in bold red.

NSETPYINTEQQIPKKKIKMWRCVTRGLRSHSWKTSKISPISSSSSFSRLFSSDSNYTVVDHTYDAVVVGAGGAGLRAAIGLSES

GFNTACITKLFPTRSHTVAAQGGINAALGNMSEDDWRWHMYDTVKGSDWLGDQDAIQYMCREAPKAVIELENYGLPFSRTED

GKIYQRAFGGQSLNFGKGGQAYRCACAADRTGHALLHTLYGQAMRHNTQFFVEYFALDLIMDDEGNCQGVIALNLEDGTLH

RFRSKSTILATGGYGRAYFSATSAHTCTGDGNAMVARAGLPLED

Query	Observed	Mr(expt)	Mr(calc)	ppm	Miss	Score	Expect	Rank	Unique	Peptide
2	1095.5490	1094.5417	1094.5720	-27.72	0	20	0.58	1	U	K.STILATGGYGR.A
3	1125.5398	1124.5326	1124.5615	-25.71	0	17	0.83	1	U	R.AFGGQSLNFGK.G

17	1607.8060	1606.7988	1606.8355	-22.86	0	71	4.6e-006	1	U	K.AVIELENYGLPFSR.T
18	1914.7602	1913.7529	1913.8036	-26.50	0	42	0.00037	1	U	K.GSDWLGDQDAIQYMCR.E
19	1930.7495	1929.7423	1929.7986	-29.17	0	(4)	1.6	2	U	K.GSDWLGDQDAIQYMCR.E

+ Oxidation (M)

Database 2

Match to: Unigene55313_SALfmcTARAAPEI-3 Mass: 38850 Score: 148 Matches: 5(2) Sequences: 4(2)

gi| 502161129 PREDICTED: succinate dehydrogenase [ubiquinone] flavoprotein subunit 1, mitochondrial-like [*Cicer arietinum*]

Matched peptides shown in bold red.

MWRCVTRGLRSHSWKTSKISPISSSSSFSRLISSDSNYTVVDHTYDAVVVGAGGAGLRAAIGLSESGFNTACITKLFPTRSHTVA

AQGGINAALGNMSEDDWRWHMYDTVKGSDWLGDQDAIQYMCREAPKAVIELENYGLPFSRTEDGKIYQRAFGGQSLNFGK

GGQAYRCACAADRTGHALLHTLYGQAMRHNTQFFVEYFALDLIMDDEGNCQGVIALNLEDGTLHRFRSKSTILATGGYGRAY

FSATSAHTCTGDGNAMVARAGLPLEDLEFVQFHPTGIYGAGCLITEGSRGEGGILRNSEGERFMERYAPTAKDLASRDVVSRSM

TMEIREGRGVGPMKDHIFLHL

Query	Observed	Mr(expt)	Mr(calc)	ppm	Miss	Score	Expect	Rank	Unique	Peptide
2	1095.5490	1094.5417	1094.5720	-27.72	0	20	0.3	1	U	K.STILATGGYGR.A
3	1125.5398	1124.5326	1124.5615	-25.71	0	17	0.44	1	U	R.AFGGQSLNFGK.G
17	1607.8060	1606.7988	1606.8355	-22.86	0	71	2.2e-006	1	U	K.AVIELENYGLPFSR.T
18	1914.7602	1913.7529	1913.8036	-26.50	0	42	0.00065	1	U	K.GSDWLGDQDAIQYMCR.E
19	1930.7495	1929.7423	1929.7986	-29.17	0	(4)	3.4	7	U	K.GSDWLGDQDAIQYMCR.E + Oxidation (M)

Spot 2216

NCBInr protein database

Match to: gi|567124582 Mass: 15424 Score: 71 Matches: 1(1) Sequences: 1(1)

hypothetical protein EUTSA_v10019305mg [*Eutrema salsugineum*]

Matched peptides shown in bold red.

1 MGEYWNGFKG FWGERFAFLE NYTRFTKRDT PLPSWSSSDV DEFIASDPVH

51 GPTLKTAREA ATFGVTGAAL GAVSTAAFAW KYSRSPHGAA LSFLGGGVFG

101 WTFGQEVANH TLQLYKLDTM AAQVKFMEWW ERKSQGRF

Query	Observed	Mr(expt)	Mr(calc)	ppm	Miss	Score	Expect	Rank	Unique	Peptide
13	1160.5946	1159.5873	1159.5662	18.2	0	71	0.00028	1	U	R.FAFLENYTR.F

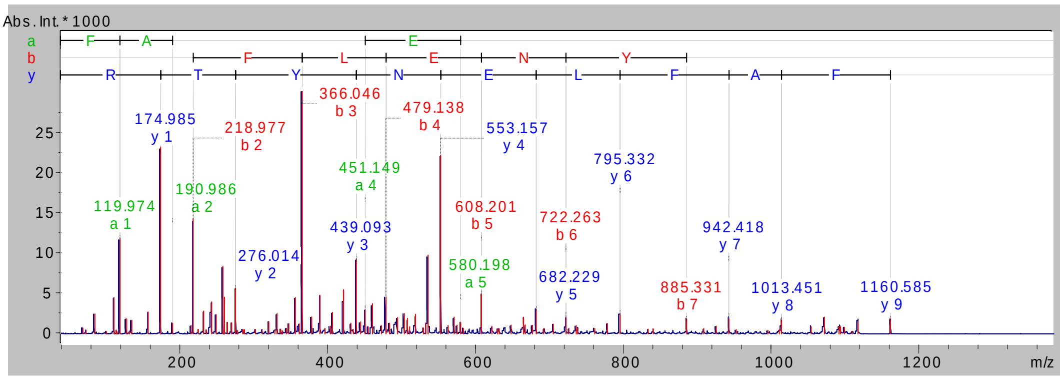

Database 1

Match to: Unigene5676_Se200S transcribed RNA sequence Mass: 21320 Score: 110 Matches: 3(3) Sequences: 2(2)

gi| 18390902 succinate dehydrogenase 6 [*Arabidopsis thaliana*]

Matched peptides shown in bold red.

RKKKRQSLEKKERNKKRKKHRKREKLRARKEEEMGDVAKDGDSMMETTKSYFEGFKGFWIDRFAFLENYTRFTNRDKPLPS

WSEADVEAFIASDPVHGPALRTAREAVNYCLVGSAVGAVTTAGFCWKYSKSPHGAVLGLGAGALFGWTFGHEVGNHALQLYK

LDTMTAQTKFMEWWEKKSEGRS

Query	Observed	Mr(expt)	Mr(calc)	ppm	Miss	Score	Expect	Rank	Unique	Peptide
7	1055.4889	1054.4816	1054.4582	22.2	0	39	0.0046	1	U	K.FMEWWEK.K
8	1071.4803	1070.4731	1070.4531	18.6	0	(33)	0.014	1	U	K.FMEWWEK.K + Oxidation (M)
13	1160.5946	1159.5873	1159.5662	18.2	0	71	5.4e-006	1	U	R.FAFLENYTR.F

Proteins matching the same set of peptides:

Unigene89227_SeCKS transcribed RNA sequence Mass: 20821 Score: 110 Matches: 3(3) Sequences: 2(2)

gi| 567124582 hypothetical protein EUTSA_v10019305mg [*Eutrema salsugineum*]

Database 2

Match to: Unigene47492_SALfmcTARAAPEI-3 Mass: 15980 Score: 110 Matches: 3(3) Sequences: 2(2)

gi| 18390902 succinate dehydrogenase 6 [*Arabidopsis thaliana*]

Matched peptides shown in bold red.

GDSMMETTKSYFEGFKGFWIDRFAFLENYTRFTNRDKPLPSWSEADVEAFIASDPVHGPALRTAREAVNYCLVGSAVGAVTTA

GFCWKYSKSPHGAVLGLGAGALFGWTFGHEVGNHALQLYKLDTMTAQTKFMEWWEKKSE

Query	Observed	Mr(expt)	Mr(calc)	ppm	Miss	Score	Expect	Rank	Unique	Peptide
7	1055.4889	1054.4816	1054.4582	22.2	0	39	0.0024	1	U	K.FMEWWEK.K
8	1071.4803	1070.4731	1070.4531	18.6	0	(33)	0.0092	1	U	K.FMEWWEK.K + Oxidation (M)
13	1160.5946	1159.5873	1159.5662	18.2	0	71	2.6e-006	1	U	R.FAFLENYTR.F

Spot 1267

NCBInr protein database

Match to: gi|1168410 Mass: 38638 Score: 326 Matches: 3(2) Sequences: 3(2)

RecName: Full=Fructose-bisphosphate aldolase, cytoplasmic isozyme 2 [*Pisum sativum*]

Matched peptides shown in bold red.

1 MSHFKSKYHD ELIANAAYIG TPGKGILAAD ESTGTIGKRL SSINVENVES

51 NRQALRELLF TASWLFLQYL SGVILFEETL YQKTAAGKPF VDVLNEAGVL

101 PGIKVDKGTV ELAGTDGETT TQGLDGLGAR CRKYYEAGAR FAKWRAVLKI

151 GANEPSEHSI HENAYGLARY AVICQENGLV PIVEPEILVD GSHDILKCAA

201 ITERVLAATY KALSDHHVIL EGTLLKPNMV TPGSDAPKVA PEVIAEHTVR

251 ALQRTVPAAV PAVVFLSGGQ SEEEASVNLN AINQIKGKKP WTLSFSFGRA

301 LQQSTLKAWG GKTENVKAAQ DALLTRAKAN SEATLGTYKG ASNLGAGASE

351 SLHVKDYKY

Query	Observed	Mr(expt)	Mr(calc)	ppm	Miss	Score	Expect	Rank	Unique	Peptide
2	829.4059	828.3987	828.3766	26.7	0	27	5.8	7	U	K.YYEAGAR.F
24	2219.0682	2218.0610	2218.0714	-4.69	0	147	3.9e-12	1	U	K.GTVELAGTDGETTTQGLDGLGAR.C
27	2561.2551	2560.2478	2560.2617	-5.43	1	153	7.6e-13	1	U	

K.VDKGTVELAGTDGETTTQGLDGLGAR.C

Proteins matching the same set of peptides:

gi|4586596 Mass: 15917 Score: 314 Matches: 3(2) Sequences: 3(2)

fructose-bisphosphate aldolase [*Cicer arietinum*]

gi|388511487 Mass: 38392 Score: 314 Matches: 3(2) Sequences: 3(2)

unknown [*Medicago truncatula*]

gi|525313856 Mass: 38599 Score: 314 Matches: 3(2) Sequences: 3(2)

fructose-bisphosphate aldolase, cytoplasmic isozyme [*Cicer arietinum*]

gi|357490465 Mass: 78758 Score: 309 Matches: 3(2) Sequences: 3(2)

Fructose-bisphosphate aldolase [*Medicago truncatula*]

Spot 1807

NCBInr protein database

Match to: gi|1168410 Mass: 38638 Score: 270 Matches: 2(2) Sequences: 2(2)

RecName: Full=Fructose-bisphosphate aldolase, cytoplasmic isozyme 2 [*Pisum sativum*]

Matched peptides shown in **bold red**.

1 MSHFKSKYHD ELIANAAYIG TPGKGILAAD ESTGTIGKRL SSINVENVES

51 NRQALRELLF TASWLFLQYL SGVILFEETL YQKTAAGKPF VDVLNEAGVL

101 PGIK**VDKGTV ELAGTDGETT TQGLDGLGAR** CRKYYEAGAR FAKWRAVLKI

151 GANEPSEHSI HENAYGLARY AVICQENGLV PIVEPEILVD GSHDILKCAA

201 ITERVLAATY KALSDHHVIL EGTLLKPNMV TPGSDAPKVA PEVIAEHTVR

251 ALQRTVPAAV PAVVFLSGGQ SEEEASVNLN AINQIKGKKP WTLSFSFGRA

301 LQQSTLKAWG GKTENVKAAQ DALLTRAKAN SEATLGTYKG ASNLGAGASE

351 SLHVKDYKY

Query	Observed	Mr(expt)	Mr(calc)	ppm	Miss	Score	Expect	Rank	Unique	Peptide
23	2219.0916	2218.0844	2218.0714	5.86	0	127	3.4e-10	1	U	

K.GTVELAGTDGETTTQGLDGLGAR.C

25	2561.2670	2560.2597	2560.2617	-0.77	1	143	7.6e-12	1	U	

K.VDKGTVELAGTDGETTTQGLDGLGAR.C

Proteins matching the same set of peptides:

gi|4586596 Mass: 15917 Score: 270 Matches: 2(2) Sequences: 2(2)

fructose-bisphosphate aldolase [*Cicer arietinum*]

gi|388511487 Mass: 38392 Score: 270 Matches: 2(2) Sequences: 2(2)

unknown [*Medicago truncatula*]

gi|388515869 Mass: 34469 Score: 270 Matches: 2(2) Sequences: 2(2)

unknown [*Lotus japonicus*]

gi|525313856 Mass: 38599 Score: 270 Matches: 2(2) Sequences: 2(2)

fructose-bisphosphate aldolase, cytoplasmic isozyme [*Cicer arietinum*]

gi|357490465 Mass: 78758 Score: 267 Matches: 2(2) Sequences: 2(2)

Fructose-bisphosphate aldolase [*Medicago truncatula*]

Database 1

Match to: Unigene45974_SeCKS transcribed RNA sequence Mass: 23763 Score: 96 Matches: 2(1) Sequences: 2(1)

gi|113624 RecName: Full=Fructose-bisphosphate aldolase, cytoplasmic isozyme [*Spinacia oleracea*]

Matched peptides shown in **bold red**.

KPFVDVMKEGGVLPGIKVDKGTVELAGTNGETTTQGLDGLGARCAK**YYEAGAR**FAKWRAVLK**IGPTEPSPLAILENANGLAR**

YAIICQENGLVPIVEPEILVDGSHDIDRCAEVSERVLAACYKALNDHHVLLEGTLLKPNMVTPGSDSKKVSNDVIAEYTVRTLQ

RTVPPAVPGVMFLSGGQSEEEATLNLNAMNKLQSKKPWTLSFSYGRALQQSTL

Query	Observed	Mr(expt)	Mr(calc)	ppm	Miss	Score	Expect	Rank	Unique	Peptide
4	829.3880	828.3807	828.3766	5.02	0	14	1	1	U	K.YYEAGAR.F
18	2033.1084	2032.1012	2032.0953	2.90	0	82	2e-007	1	U	K.IGPTEPSPLAILENANGLAR.Y

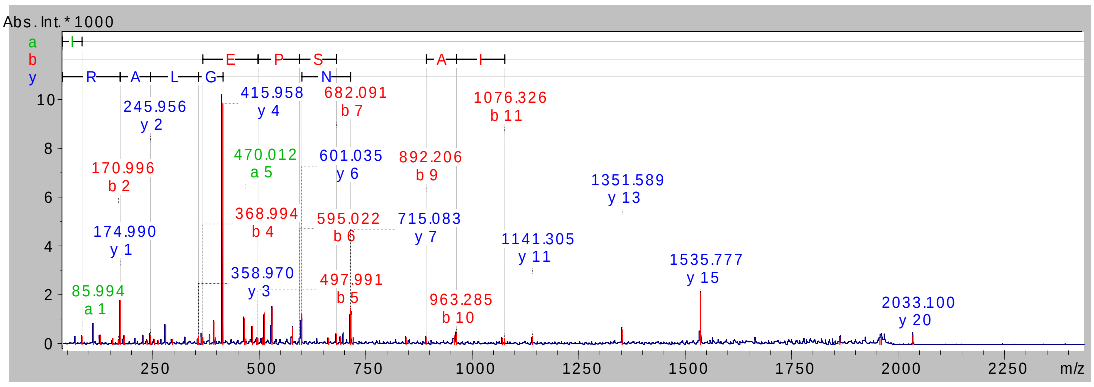

Proteins matching the same set of peptides:

Unigene46666_Se200S transcribed RNA sequence Mass: 28501 Score: 96 Matches: 2(1) Sequences: 2(1)

gi|113624 RecName: Full=Fructose-bisphosphate aldolase, cytoplasmic isozyme [*Spinacia oleracea*]

Spot 1827

NCBInr protein database

Match to: gi|1351274 Mass: 20756 Score: 73 Matches: 2(1) Sequences: 2(1)

RecName: Full=Triosephosphate isomerase, cytosolic; Short=TIM; Short=Triose-phosphate isomerase , partial [*Lactuca sativa*]

Matched peptides shown in bold red.

1 IQVAAQNCWV KKGGAFTGEV SAEMLANLGV PWVILGHSER R**ALLNETNEF**

51 **VGDK**VAYALS QGLK**VIACVG ETLEQR**EAGT TMEVVAAQTK AIADKISSWD

101 NVVLAYEPVW AIGTGKVASP AQAQEVHAGL RKWFCDNVSA EVSASTRIIY

151 GGSVSGSNCK ELGGQTDVDG FLVGGASLKP EFIDIIKAAE VKKSA

Query	Observed	Mr(expt)	Mr(calc)	ppm	Miss	Score	Expect	Rank	Unique	Peptide
23	1374.7160	1373.7088	1373.6973	8.35	0	52	0.018	1	U	K.VIACVGETLEQR.E
26	1449.7238	1448.7165	1448.7147	1.22	0	20	25	1	U	R.ALLNETNEFVGDK.V

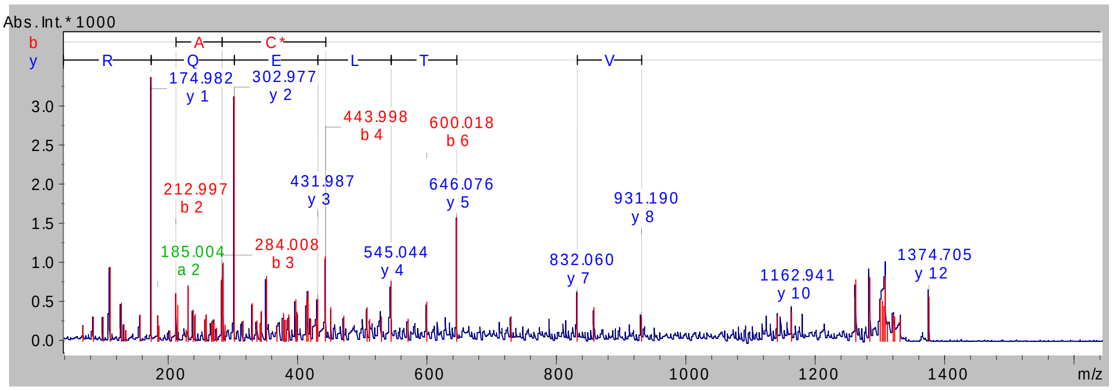

Database 1

Match to: Unigene72_SeCKS transcribed RNA sequence Mass: 29468 Score: 230 Matches: 5(3) Sequences: 5(3)

gi| 165973012 triosephosphate isomerase [*Oryza coarctata*]

Matched peptides shown in bold red.

TTPRKKSQFHEKISSSSKNMGRK**FFVGGNWK**CNGTTGEVKKIVDTLNAGNVPSTDVVEVVLSPPFVFLPFVKSSLRPEFQVAA

QNCWVKKGGAFTGEISAEMLADLEVPWVILGHSERR**ALLNESNEFVGDK**VAYALSKGLK**VIACVGETLEQR**EAGTTMDVVAA

QTKAIADRVTDWTNVVVAYEPVWAIGTGK**VATPEQAQEVHCDLR**K**WLSANVSPEVASSTR**IIYGGSVNGANCKELAAKPDVD

GFLVGGASLKPEFIDIINSATVKSA

Query	Observed	Mr(expt)	Mr(calc)	ppm	Miss	Score	Expect	Rank	Unique	Peptide
6	954.4824	953.4751	953.4760	-0.87	0	8	8.6	1	U	K.FFVGGNWK.C
23	1374.7160	1373.7088	1373.6973	8.35	0	52	0.00037	1	U	K.VIACVGETLEQR.E
25	1435.7209	1434.7136	1434.6991	10.1	0	12	4.1	1	U	R.ALLNESNEFVGDK.V
28	1603.8160	1602.8087	1602.8002	5.31	0	74	2e-006	1	U	K.WLSANVSPEVASSTR.I
29	1752.8488	1751.8415	1751.8261	8.82	0	84	1.9e-007	1	U	K.VATPEQAQEVHCDLR.K

Proteins matching the same set of peptides:

Unigene73_SeCKS transcribed RNA sequence Mass: 30020 Score: 230 Matches: 5(3) Sequences: 5(3)

gi| 165973012 triosephosphate isomerase [*Oryza coarctata*]

Unigene15247_Se200S transcribed RNA sequence Mass: 29468 Score: 230 Matches: 5(3) Sequences: 5(3)

gi| 165973012 triosephosphate isomerase [*Oryza coarctata*]

Database 2

Match to: Unigene37328_SALfmcTARAAPEI-3 Mass: 11710 Score: 158 Matches: 2(2) Sequences: 2(2)

gi| 149391828 triosephosphate isomerase, cytosolic [*Oryza sativa Indica Group*]

Matched peptides shown in bold red.

GTTMDVVAAQTKAIADRVTDWTNVVVAYEPVWAIGTGK**VATPEQAQEVHCDLR**K**WLSANVSPEVASSTR**IIYGGSVNGANCK

ELAAKPDVDGFLVGGASLKPEFIDIINS

Query	Observed	Mr(expt)	Mr(calc)	ppm	Miss	Score	Expect	Rank	Unique	Peptide
28	1603.8160	1602.8087	1602.8002	5.31	0	74	8.5e-007	1	U	K.WLSANVSPEVASSTR.I
29	1752.8488	1751.8415	1751.8261	8.82	0	84	8.8e-008	1	U	K.VATPEQAQEVHCDLR.K

Spot 755

NCBInr protein database

Match to: gi|114408 Mass: 55847 Score: 306 Matches: 7(2) Sequences: 7(2)

RecName: Full=ATP synthase subunit alpha, mitochondrial [*Oenothera biennis*]

Matched peptides shown in bold red.

1 MEFSPRAAEL TTLLESR**ITN FYTNFQVDEI GR**VISVGDGI ARVYGLNEIQ

51 AGEMVEFASG VKGIALNLEN ENVGIVVFGS DTAIKEGDLV KRTGSIVDVP

101 AGKSLLGR**VV DALGVPIDGR** GALGDHERRR VEVKVPGIIE RKSVHEPMQT

151 GLK**AVDSLVP IGR**GQRELII GDRQTGKTAI AIDTILNQKQ MNSRATSESE

201 TLYCVYVAIG QKRSTVAQLV QILSEGNALE YSILVAATAS DPAPLQFLAP

251 YSGCAMGEYF RDNGMHALII YDDLSKQAVA YRQMSLLLRR PPGR**EAFPGD**

301 **VFYLHSR**LLE RAAKRSDQTG AGSLTALPVI ETQAGDVSAY IPTNVISITD

351 GQICLETELF YRGIRPAINV GLSVSRVGSA AQLKAMKQVC GSLK**LELAQY**

401 **REVAAFAQFG SDLDAATQAL LNR**GARLTEI LKQP**QYAPLP IEKQIIVIYA**

451 **AVNGFCDR**MP LDRISQYERA IPQSVKQELL QSLVEKGGLN NERKIEPDAF

501 LKENAKPYIK G

Query	Observed	Mr(expt)	Mr(calc)	ppm	Miss	Score	Expect	Rank	Unique	Peptide
3	892.5129	891.5056	891.4814	27.2	0	17	69	2		K.LELAQYR.E
4	1026.6220	1025.6147	1025.5869	27.0	0	25	6.4	1		K.AVDSLVPIGR.G

9	1210.7109	1209.7036	1209.6718	26.3	0	44	0.082	1		R.VVDALGVPIDGR.G
14	1537.7760	1536.7687	1536.7361	21.2	0	80	2.8e-05	1		R.EAFPGDVFYLHSR.L
18	1738.9221	1737.9149	1737.8872	15.9	0	16	53	5	U	K.QIIVIYAAVNGFCDR.M
20	1816.9160	1815.9087	1815.8792	16.3	0	100	2.5e-07	1		R.ITNFYTNFQVDEIGR.V
26	2308.2053	2307.1980	2307.1495	21.0	0	23	8.4	1		R.EVAAFAQFGSDLDAATQALLNR.G

Proteins matching the same set of peptides:

gi|576303588 Mass: 55721 Score: 302 Matches: 7(2) Sequences: 7(2)

ATPase subunit 1 (mitochondrion) [*Vaccinium macrocarpon*]

2. gi|372450254 Mass: 55602 Score: 304 Matches: 7(2) Sequences: 7(2)

ATPase subunit 1 (mitochondrion) [*Millettia pinnata*]

Query	Observed	Mr(expt)	Mr(calc)	ppm	Miss	Score	Expect	Rank	Unique	Peptide
3	892.5129	891.5056	891.4814	27.2	0	17	69	2		K.LELAQYR.E
4	1026.6220	1025.6147	1025.5869	27.0	0	25	6.4	1		K.AVDSLVPIGR.G
9	1210.7109	1209.7036	1209.6718	26.3	0	44	0.082	1		R.VVDALGVPIDGR.G
14	1537.7760	1536.7687	1536.7361	21.2	0	80	2.8e-05	1		R.EAFPGDVFYLHSR.L
18	1738.9221	1737.9149	1737.8872	15.9	0	16	53	5	U	K.QILVIYAAVNGFCDR.M
20	1816.9160	1815.9087	1815.8792	16.3	0	100	2.5e-07	1		R.ITNFYTNFQVDEIGR.V
26	2308.2053	2307.1980	2307.1495	21.0	0	23	8.4	1		R.EVAAFAQFGSDLDAATQALLNR.G

Proteins matching the same set of peptides:

gi|357982 Mass: 55326 Score: 302 Matches: 7(2) Sequences: 7(2)

ATPase alpha,F1

gi|543865 Mass: 55246 Score: 302 Matches: 7(2) Sequences: 7(2)

RecName: Full=ATP synthase subunit alpha, mitochondrial

gi|543866 Mass: 55296 Score: 302 Matches: 7(2) Sequences: 7(2)

RecName: Full=ATP synthase subunit alpha, mitochondrial

gi|162279935 Mass: 55294 Score: 302 Matches: 7(2) Sequences: 7(2)

atp1 gene product (mitochondrion) [*Beta vulgaris subsp. vulgaris*]

gi|295311633 Mass: 55573 Score: 302 Matches: 7(2) Sequences: 7(2)

ATPase subunit 1 [*Citrullus lanatus*]

gi|320148078 Mass: 55308 Score: 302 Matches: 7(2) Sequences: 7(2)

ATPase subunit 1 [*Beta vulgaris subsp. maritima*]

gi|372450305 Mass: 55158 Score: 302 Matches: 7(2) Sequences: 7(2)

ATPase subunit 1 (mitochondrion) [*Lotus japonicus*]

gi|429465362 Mass: 54717 Score: 302 Matches: 7(2) Sequences: 7(2)

ATP synthase subunit 1, partial (mitochondrion) [*Gossypium hirsutum*]

gi|476507691 Mass: 55581 Score: 302 Matches: 7(2) Sequences: 7(2)

ATPase subunit 1 (mitochondrion) [*Glycine max*]

3. gi|224020956 Mass: 55447 Score: 264 Matches: 5(2) Sequences: 5(2)

ATP synthase F1 subunit 1 [*Carica papaya*]

Query	Observed	Mr(expt)	Mr(calc)	ppm	Miss	Score	Expect	Rank	Unique	Peptide
3	892.5129	891.5056	891.4814	27.2	0	17	69	2		K.LELAQYR.E

4	1026.6220	1025.6147	1025.5869	27.0	0	25	6.4	1		K.AVDSLVPIGR.G
9	1210.7109	1209.7036	1209.6718	26.3	0	44	0.082	1		R.VVDALGVPIDGR.G
14	1537.7760	1536.7687	1536.7361	21.2	0	80	2.8e-05	1		R.EAFPGDVFYLHSR.L
20	1816.9160	1815.9087	1815.8792	16.3	0	100	2.5e-07	1	U	R.LTNFYTNFQVDEIGR.V

Database 1

Match to: Unigene89272_SeCKS transcribed RNA sequence Mass: 19896 Score: 167 Matches: 3(2) Sequences: 3(2)

gi|22742 pseudo-atpA [*Glycine max*]

Matched peptides shown in **bold red**.

IMEFSPRAAELTTLLESR**ITNFYTNFQVDEIGR**VVSVGDGIARVYGLNEIQAGEMVEFASGVKGIALNLENENVGIVVFGSDTAI

KEGDLVKRTGSIVDVPAGKAMLGR**VVDALGVPIDGR**GALSDHERRRVEVKAPGIIERKSVHEPMQTGLK**AVDSLVPIGR**GQRE

LIIGDRQTGKTAIAIDTI

Query	Observed	Mr(expt)	Mr(calc)	ppm	Miss	Score	Expect	Rank	Unique	Peptide
4	1026.6220	1025.6147	1025.5869	27.0	0	25	0.091	1	U	K.AVDSLVPIGR.G
9	1210.7109	1209.7036	1209.6718	26.3	0	44	0.00085	1	U	R.VVDALGVPIDGR.G
20	1816.9160	1815.9087	1815.8792	16.3	0	100	5.3e-009	1	U	R.ITNFYTNFQVDEIGR.V

Database 2

Match to: Unigene16541_SALfmcTARAAPEI-3 Mass: 20907 Score: 174 Matches: 4(2) Sequences: 4(2)

gi|22742 pseudo-atpA [*Glycine max*]

Matched peptides shown in **bold red**.

MEFSPRAAELTTLLESR**ITNFYTNFQVDEIGR**VVSVGDGIARVYGLNEIQAGEMVEFASGVKGIALNLENENVGIVVFGSDTAIK

EGDLVKRTGSIVDVPAGKAMLGR**VVDALGVPIDGR**GALSDHERRRVEVKAPGIIERKSVHEPMQTGLK**AVDSLVPIGR**GQRELI

IGDRQTGKTAIAIDTILNQKQLNSKA

Query	Observed	Mr(expt)	Mr(calc)	ppm	Miss	Score	Expect	Rank	Unique	Peptide
1	782.3731	781.3658	781.3429	29.4	0	7	4.4	2	U	-.MEFSPR.A + Oxidation (M)
4	1026.6220	1025.6147	1025.5869	27.0	0	25	0.057	1	U	K.AVDSLVPIGR.G
9	1210.7109	1209.7036	1209.6718	26.3	0	44	0.00063	1	U	R.VVDALGVPIDGR.G
20	1816.9160	1815.9087	1815.8792	16.3	0	100	2.3e-009	1	U	R.ITNFYTNFQVDEIGR.V

2. Unigene53297_SALfmcTARAAPEI-3 Mass: 33461 Score: 137 Matches: 4(1) Sequences: 4(1)

gi| 162279935 atp1 gene product (mitochondrion) [*Beta vulgaris subsp. vulgaris]*

Query	Observed	Mr(expt)	Mr(calc)	ppm	Miss	Score	Expect	Rank	Unique	Peptide
3	892.5129	891.5056	891.4814	27.2	0	17	0.63	1	U	K.LELAQYR.E
14	1537.7760	1536.7687	1536.7361	21.2	0	80	2.6e-007	1	U	R.EAFPGDVFYLHSR.L
18	1738.9221	1737.9149	1737.8872	15.9	0	16	0.47	1	U	K.QILVIYAAVNGFCDR.M
26	2308.2053	2307.1980	2307.1495	21.0	0	23	0.067	1	U	R.EVAAFAQFGSDLDAATQALLNR.G

Spot 760

NCBInr protein database

gi|372450254 Mass: 55602 Score: 944 Matches: 16(9) Sequences: 16(9)

ATPase subunit 1 (mitochondrion) [*Millettia pinnata*]

Matched peptides shown in **bold red**.

1 MDFSVR**AAEL TTLLESRITN FYTNFQVDEI GRVVSVGDGI ARVYGLNEIQ**

51 AGEMVEFASG VKGVALNLEN ENVGIIVFGS DTAIKEGDLV KRTGSIVDVP

101 AGKAMLGRVV DALGVPIDGR GALSDHERRR VDVKAPGIIE RKSVHEPMQT

151 GLKAVDSLVP IGRGQRELII GDRQTGKTAI AIDTILNQKQ MNSRATSESE

201 TLYCVYVAIG QKRSTVAQLV QILSEANALE YSILVAATAS DPAPLQYLAP

251 YSGCAMGEYF RDNGMHALII YDDLSKQAVA YRQMSLLLRR PPGREAFPGD

301 VFYLHSRLLE RAAKRSDQTG AGSLTALPVI ETQAGDVSAY IPTNVISITD

351 GQICLETELF YRGIRPAINV GLSVSRVGSA AQLKAMKQVC GSLKLELAQY

401 REVAAFAQFG SDLDAATQAL LNRGARLTEV LKQPQYAPLP IEKQILVIYA

451 AVNGFCDRMP LEKIPQYERD ILTTIKPELL QSLKGVLNSE RKIELDAFLK

501 EKAKTYLI

Query	Observed	Mr(expt)	Mr(calc)	ppm	Miss	Score	Expect	Rank	Unique	Peptide
2	755.4491	754.4418	754.4337	10.7	0	33	1	1		K.APGIIER.K
6	815.4770	814.4697	814.4548	18.2	0	18	58	2		R.ELIIGDR.Q
8	876.5105	875.5032	875.4899	15.3	0	19	49	2		R.QMSLLLR.R + Oxidation (M)
9	884.4394	883.4321	883.4148	19.6	0	18	44	3		R.GALSDHER.R
10	892.5034	891.4962	891.4814	16.6	0	31	3.2	1		K.LELAQYR.E
11	972.5626	971.5553	971.5400	15.7	0	28	5.7	2		R.VVSVGDGIAR.V
12	1026.6175	1025.6102	1025.5869	22.7	0	72	0.00012	1		K.AVDSLVPIGR.G
13	1203.6928	1202.6855	1202.6506	29.0	0	53	0.014	1	U	R.AAELTTLLESR.I
14	1210.7082	1209.7010	1209.6718	24.1	0	64	0.001	1		R.VVDALGVPIDGR.G
17	1242.6447	1241.6374	1241.6074	24.2	0	32	1.9	1		K.SVHEPMQTGLK.A + Oxidation (M)
19	1300.7765	1299.7693	1299.7398	22.7	0	63	0.00099	1		K.TAIAIDTILNQK.Q
21	1537.7854	1536.7781	1536.7361	27.3	0	98	4.6e-07	1		R.EAFPGDVFYLHSR.L
27	1816.9327	1815.9255	1815.8792	25.5	0	113	1.3e-08	1	U	R.ITNFYTNFQVDEIGR.V
28	1967.1528	1966.1455	1966.1139	16.1	1	84	4.8e-06	1		R.LTEVLKQPQYAPLPIEK.Q
30	2141.1138	2140.1065	2140.0510	25.9	0	79	2.2e-05	1		R.VYGLNEIQAGEMVEFASGVK.G
37	2308.2314	2307.2242	2307.1495	32.4	0	138	2.8e-11	1		R.EVAAFAQFGSDLDAATQALLNR.G

Proteins matching the same set of peptides:

gi|476507691 Mass: 55581 Score: 942 Matches: 16(9) Sequences: 16(9)

ATPase subunit 1 (mitochondrion) [*Glycine max*]

gi|372450305 Mass: 55158 Score: 940 Matches: 16(9) Sequences: 16(9)

ATPase subunit 1 (mitochondrion) [*Lotus japonicus*]

2. gi|55824792 Mass: 46438 Score: 776 Matches: 14(7) Sequences: 14(7)

F1-ATPase alpha subunit [*Humbertia madagascariensis*]

Query	Observed	Mr(expt)	Mr(calc)	ppm	Miss	Score	Expect	Rank	Unique	Peptide
2	755.4491	754.4418	754.4337	10.7	0	33	1	1		K.APGIIER.K
6	815.4770	814.4697	814.4548	18.2	0	18	58	2		R.ELIIGDR.Q
8	876.5105	875.5032	875.4899	15.3	0	19	49	2		R.QMSLLLR.R + Oxidation (M)
9	884.4394	883.4321	883.4148	19.6	0	18	44	3		R.GALSDHER.R
10	892.5034	891.4962	891.4814	16.6	0	31	3.2	1		K.LELAQYR.E
11	972.5626	971.5553	971.5400	15.7	0	28	5.7	2		R.VVSVGDGIAR.V

12	1026.6175	1025.6102	1025.5869	22.7	0	72	0.00012	1		K.AVDSLVPIGR.G
14	1210.7082	1209.7010	1209.6718	24.1	0	64	0.001	1		R.VVDALGVPIDGR.G
17	1242.6447	1241.6374	1241.6074	24.2	0	32	1.9	1		K.SVHEPMQTGLK.A + Oxidation (M)
19	1300.7765	1299.7693	1299.7398	22.7	0	63	0.00099	1		K.TAIAIDTILNQK.Q
21	1537.7854	1536.7781	1536.7361	27.3	0	98	4.6e-07	1		R.EAFPGDVFYLHSR.L
28	1967.1528	1966.1455	1966.1139	16.1	1	84	4.8e-06	1		R.LTEVLKQPQYAPLPIEK.E
30	2141.1138	2140.1065	2140.0874	8.92	1	79	2.2e-05	1	U	R.VYGLNEIKAGEMVEFASGVK.G
37	2308.2314	2307.2242	2307.1495	32.4	0	138	2.8e-11	1		R.EVAAFAQFGSDLDAATQALLNR.G

3. gi|55976884 Mass: 45248 Score: 742 Matches: 14(6) Sequences: 14(6)

F1-ATPase alpha subunit [*Pilostyles thurberi*]

Query	Observed	Mr(expt)	Mr(calc)	ppm	Miss	Score	Expect	Rank	Unique	Peptide
2	755.4491	754.4418	754.4337	10.7	0	33	1	1		K.APGIIER.K
6	815.4770	814.4697	814.4548	18.2	0	18	58	2		R.ELIIGDR.Q
8	876.5105	875.5032	875.4899	15.3	0	19	49	2		R.QMSLLLR.R + Oxidation (M)
9	884.4394	883.4321	883.4148	19.6	0	18	44	3		R.GALSDHER.R
10	892.5034	891.4962	891.4814	16.6	0	31	3.2	1		K.LELAQYR.E
11	972.5626	971.5553	971.5400	15.7	0	28	5.7	2		R.VVSVGDGIAR.V
12	1026.6175	1025.6102	1025.5869	22.7	0	72	0.00012	1		K.AVDSLVPIGR.G
14	1210.7082	1209.7010	1209.6718	24.1	0	30	2.4	10	U	R.VVDGLGLPIDGR.G
17	1242.6447	1241.6374	1241.6074	24.2	0	32	1.9	1		K.SVHEPMQTGLK.A + Oxidation (M)
19	1300.7765	1299.7693	1299.7398	22.7	0	63	0.00099	1		K.TAIAIDTILNQK.Q
21	1537.7854	1536.7781	1536.7361	27.3	0	98	4.6e-07	1		R.EAFPGDVFYLHSR.L
28	1967.1528	1966.1455	1966.1139	16.1	1	84	4.8e-06	1		R.LTEVLKQPQYAPLPIEK.Q
30	2141.1138	2140.1065	2140.0510	25.9	0	79	2.2e-05	1		R.VYGLNEIQAGEMVEFASGVK.G
37	2308.2314	2307.2242	2307.1495	32.4	0	138	2.8e-11	1		R.EVAAFAQFGSDLDAATQALLNR.G

4. gi|55976896 Mass: 45242 Score: 633 Matches: 12(5) Sequences: 12(5)

F1-ATPase alpha subunit [*Rhizanthes infanticida*]

Query	Observed	Mr(expt)	Mr(calc)	ppm	Miss	Score	Expect	Rank	Unique	Peptide
2	755.4491	754.4418	754.4337	10.7	0	33	1	1		K.APGIIER.K
6	815.4770	814.4697	814.4548	18.2	0	18	58	2		R.ELIIGDR.Q
8	876.5105	875.5032	875.4899	15.3	0	19	49	2		R.QMSLLLR.R + Oxidation (M)
9	884.4394	883.4321	883.4148	19.6	0	18	44	3	U	R.GAISDHER.R
10	892.5034	891.4962	891.4814	16.6	0	31	3.2	1		K.LELAQYR.E
11	972.5626	971.5553	971.5400	15.7	0	28	5.7	2		R.VVSVGDGIAR.V
12	1026.6175	1025.6102	1025.5869	22.7	0	72	0.00012	1		K.AVDSLVPIGR.G
14	1210.7082	1209.7010	1209.6718	24.1	0	64	0.001	1		R.VVDALGVPIDGR.G
17	1242.6447	1241.6374	1241.6074	24.2	0	32	1.9	1		K.SVHEPMQTGLK.A + Oxidation (M)
21	1537.7854	1536.7781	1536.7361	27.3	0	98	4.6e-07	1		R.EAFPGDVFYLHSR.L
28	1967.1528	1966.1455	1966.1139	16.1	1	84	4.8e-06	1		R.LTEVLKQPQYAPLPIEK.Q
37	2308.2314	2307.2242	2307.1495	32.4	0	138	2.8e-11	1		R.EVAAFAQFGSDLDAATQALLNR.G

5. gi|224020956 Mass: 55447 Score: 619 Matches: 14(5) Sequences: 13(5)

ATP synthase F1 subunit 1 [*Carica papaya*]

Query	Observed	Mr(expt)	Mr(calc)	ppm	Miss	Score	Expect	Rank	Unique	Peptide
2	755.4491	754.4418	754.4337	10.7	0	33	1	1		K.APGIIER.K

3	766.3632	765.3560	765.3479	10.5	0	(24)	14	3		-.MEFSPR.A
4	782.3536	781.3463	781.3429	4.46	0	31	2	1		-.MEFSPR.A + Oxidation (M)
6	815.4770	814.4697	814.4548	18.2	0	18	58	2		R.ELIIGDR.Q
8	876.5105	875.5032	875.4899	15.3	0	19	49	2		R.QMSLLLR.R + Oxidation (M)
9	884.4394	883.4321	883.4148	19.6	0	18	44	3		R.GALSDHER.R
10	892.5034	891.4962	891.4814	16.6	0	31	3.2	1		K.LELAQYR.E
11	972.5626	971.5553	971.5400	15.7	0	28	5.7	2		R.VVSVGDGIAR.V
12	1026.6175	1025.6102	1025.5869	22.7	0	72	0.00012	1		K.AVDSLVPIGR.G
14	1210.7082	1209.7010	1209.6718	24.1	0	64	0.001	1		R.VVDALGVPIDGR.G
17	1242.6447	1241.6374	1241.6074	24.2	0	32	1.9	1		K.SVHEPMQTGLK.A + Oxidation (M)
19	1300.7765	1299.7693	1299.7398	22.7	0	63	0.00099	1		K.TAIAIDTILNQK.Q
21	1537.7854	1536.7781	1536.7361	27.3	0	98	4.6e-07	1		R.EAFPGDVFYLHSR.L
27	1816.9327	1815.9255	1815.8792	25.5	0	113	1.3e-08	1	U	R.LTNFYTNFQVDEIGR.V

6. gi|55824802 Mass: 45589 Score: 612 Matches: 12(5) Sequences: 12(5)

F1-ATPase alpha subunit [*Justicia carnea*]

Query	Observed	Mr(expt)	Mr(calc)	ppm	Miss	Score	Expect	Rank	Unique	Peptide
2	755.4491	754.4418	754.4337	10.7	0	33	1	1		K.APGIIER.K
6	815.4770	814.4697	814.4548	18.2	0	18	58	2		R.ELLIGDR.Q
8	876.5105	875.5032	875.4899	15.3	0	19	49	2		R.QMSLLLR.R + Oxidation (M)
9	884.4394	883.4321	883.4148	19.6	0	18	44	3		R.GALSDHER.R
10	892.5034	891.4962	891.4814	16.6	0	31	3.2	1		K.LELAQYR.E
11	972.5626	971.5553	971.5400	15.7	0	28	5.7	2		R.VVSVGDGIAR.V
12	1026.6175	1025.6102	1025.5869	22.7	0	72	0.00012	1		K.AVDSLVPIGR.G
14	1210.7082	1209.7010	1209.6718	24.1	0	64	0.001	1		R.VVDALGVPIDGR.G
17	1242.6447	1241.6374	1241.6074	24.2	0	32	1.9	1		K.SVHEPMQTGLK.A + Oxidation (M)
19	1300.7765	1299.7693	1299.7398	22.7	0	63	0.00099	1		K.TAIAIDTILNQK.Q
21	1537.7854	1536.7781	1536.7361	27.3	0	98	4.6e-07	1		R.EAFPGDVFYLHSR.L
37	2308.2314	2307.2242	2307.1495	32.4	0	138	2.8e-11	1		R.EVAAFAQFGSDLDAATQALLNR.G

7. gi|27461619 Mass: 44865 Score: 602 Matches: 11(5) Sequences: 11(5)

ATPase F1 alpha subunit, partial (mitochondrion) [*Xyris bicephala*]

Query	Observed	Mr(expt)	Mr(calc)	ppm	Miss	Score	Expect	Rank	Unique	Peptide
2	755.4491	754.4418	754.4337	10.7	0	33	1	1		K.APGIIER.K
6	815.4770	814.4697	814.4548	18.2	0	18	58	2		R.ELLIGDR.Q
8	876.5105	875.5032	875.4899	15.3	0	19	49	2		R.QMSLLLR.R + Oxidation (M)
9	884.4394	883.4321	883.4148	19.6	0	18	44	3		K.GALSDHER.R
10	892.5034	891.4962	891.4814	16.6	0	31	3.2	1		K.LELAQYR.E
12	1026.6175	1025.6102	1025.5869	22.7	0	72	0.00012	1		K.AVDSLVPIGR.G
17	1242.6447	1241.6374	1241.6074	24.2	0	32	1.9	1		K.SVHEPMQTGLK.A + Oxidation (M)
19	1300.7765	1299.7693	1299.7398	22.7	0	63	0.00099	1		K.TAIAIDTILNQK.Q
21	1537.7854	1536.7781	1536.7361	27.3	0	98	4.6e-07	1		R.EAFPGDVFYLHSR.L
30	2141.1138	2140.1065	2140.0510	25.9	0	79	2.2e-05	1	U	R.VYGLNEIQAGEMVEFASGVK.G
37	2308.2314	2307.2242	2307.1495	32.4	0	138	2.8e-11	1		R.EVAAFAQFGSDLDAATQALLNR.G

8. gi|34539349 Mass: 44976 Score: 599 Matches: 11(5) Sequences: 11(5)

F1-ATPase alpha subunit [*Lacandonia schismatica*]

Query	Observed	Mr(expt)	Mr(calc)	ppm	Miss	Score	Expect	Rank	Unique	Peptide
2	755.4491	754.4418	754.4337	10.7	0	33	1	1		K.APGIIER.K
6	815.4770	814.4697	814.4548	18.2	0	18	58	2		R.ELLIGDR.Q
8	876.5105	875.5032	875.4899	15.3	0	19	49	2		R.QMSLLLR.R + Oxidation (M)
9	884.4394	883.4321	883.4148	19.6	0	18	44	3		R.GALSDHER.R
10	892.5034	891.4962	891.4814	16.6	0	31	3.2	1		K.LELAQYR.E
12	1026.6175	1025.6102	1025.5869	22.7	0	72	0.00012	1		K.AVDSLVPIGR.G
14	1210.7082	1209.7010	1209.6718	24.1	0	64	0.001	1		R.VVDALGVPIDGR.G
17	1242.6447	1241.6374	1241.6074	24.2	0	32	1.9	1		K.SVHEPMQTGLK.A + Oxidation (M)
21	1537.7854	1536.7781	1536.7361	27.3	0	98	4.6e-07	1		R.EAFPGDVFYLHSR.L
30	2141.1138	2140.1065	2140.0510	25.9	0	79	2.2e-05	1		R.VYGLNEIQAGEMVEFASGVK.G
37	2308.2314	2307.2242	2307.1495	32.4	0	138	2.8e-11	1		R.EVAAFAQFGSDLDAATQALLNR.G

9. gi|34539225 Mass: 44857 Score: 596 Matches: 11(5) Sequences: 11(5)

F1-ATPase alpha subunit [*Burmannia lutescens*]

Query	Observed	Mr(expt)	Mr(calc)	ppm	Miss	Score	Expect	Rank	Unique	Peptide
2	755.4491	754.4418	754.4337	10.7	0	33	1	1		K.APGIIER.K
6	815.4770	814.4697	814.4548	18.2	0	18	58	2		R.ELIIGDR.Q
8	876.5105	875.5032	875.4899	15.3	0	19	49	2		R.QMSLLLR.R + Oxidation (M)
9	884.4394	883.4321	883.4148	19.6	0	18	44	3		R.GALSDHER.R
10	892.5034	891.4962	891.4814	16.6	0	31	3.2	1		K.LELAQYR.E
12	1026.6175	1025.6102	1025.5869	22.7	0	72	0.00012	1		K.AVDSLVPIGR.G
14	1210.7082	1209.7010	1209.6718	24.1	0	64	0.001	1		R.VVDALGVPIDGR.G
17	1242.6447	1241.6374	1241.6074	24.2	0	32	1.9	1		K.SVHEPMQTGLK.A + Oxidation (M)
21	1537.7854	1536.7781	1536.7361	27.3	0	98	4.6e-07	1		R.EAFPGDVFYLHSR.L
30	2141.1138	2140.1065	2140.0510	25.9	0	79	2.2e-05	1		R.VYGINEIQAGEMVEFASGVK.G
37	2308.2314	2307.2242	2307.1495	32.4	0	138	2.8e-11	1		R.EVAAFAQFGSDLDAATQALLNR.G

10. gi|269819632 Mass: 42993 Score: 567 Matches: 10(5) Sequences: 10(5)

F1-ATPase alpha subunit, partial (mitochondrion) [*Kupea martinetugei*]

Query	Observed	Mr(expt)	Mr(calc)	ppm	Miss	Score	Expect	Rank	Unique	Peptide
2	755.4491	754.4418	754.4337	10.7	0	33	1	1		K.APGIIER.K
6	815.4770	814.4697	814.4548	18.2	0	18	58	2		R.ELLIGDR.Q
8	876.5105	875.5032	875.4899	15.3	0	19	49	2		R.QMSLLLR.R + Oxidation (M)
10	892.5034	891.4962	891.4814	16.6	0	31	3.2	1		K.LELAQYR.E
12	1026.6175	1025.6102	1025.5869	22.7	0	72	0.00012	1		K.AVDSLVPIGR.G
14	1210.7082	1209.7010	1209.6718	24.1	0	64	0.001	1		R.VVDALGVPLDGR.G
17	1242.6447	1241.6374	1241.6074	24.2	0	32	1.9	1		K.SVHEPMQTGLK.A + Oxidation (M)
19	1300.7765	1299.7693	1299.7398	22.7	0	63	0.00099	1		K.TAIAIDTILNQK.Q
21	1537.7854	1536.7781	1536.7361	27.3	0	98	4.6e-07	1		R.EAFPGDVFYLHSR.L
37	2308.2314	2307.2242	2307.1495	32.4	0	138	2.8e-11	1		R.EVAAFAQFGSDLDAATQALLNR.G

Proteins matching the same set of peptides:

gi|532219832 Mass: 42541 Score: 567 Matches: 10(5) Sequences: 10(5)

F1-ATPase alpha subunit, partial (mitochondrion) [*Kupea martinetugei*]

11. gi|57115583 Mass: 43597 Score: 557 Matches: 9(5) Sequences: 9(5)

F1-ATPase alpha subunit [*Goodenia ovata*]

Query	Observed	Mr(expt)	Mr(calc)	ppm	Miss	Score	Expect	Rank	Unique	Peptide
2	755.4491	754.4418	754.4337	10.7	0	33	1	1		K.APGIIER.K
6	815.4770	814.4697	814.4548	18.2	0	18	58	2		R.ELIIGDR.Q
8	876.5105	875.5032	875.4899	15.3	0	19	49	2		R.QMSLLLR.R + Oxidation (M)
10	892.5034	891.4962	891.4814	16.6	0	31	3.2	1		K.LELAQYR.E
12	1026.6175	1025.6102	1025.5869	22.7	0	72	0.00012	1		K.AVDSLVPIGR.G
19	1300.7765	1299.7693	1299.7398	22.7	0	63	0.00099	1		K.TAIAIDTIINQK.Q
21	1537.7854	1536.7781	1536.7361	27.3	0	98	4.6e-07	1		R.EAFPGDVFYLHSR.L
28	1967.1528	1966.1455	1966.1139	16.1	1	84	4.8e-06	1		R.LTEVLKQPQYAPLPIEK.Q
37	2308.2314	2307.2242	2307.1495	32.4	0	138	2.8e-11	1		R.EVAAFAQFGSDLDAATQALLNR.G

12. gi|112253900 Mass: 55382 Score: 526 Matches: 9(4) Sequences: 9(4)

ATPase subunit 1 [*Brassica napus*]

Query	Observed	Mr(expt)	Mr(calc)	ppm	Miss	Score	Expect	Rank	Unique	Peptide
2	755.4491	754.4418	754.4337	10.7	0	33	1	1		K.APGILER.K
6	815.4770	814.4697	814.4548	18.2	0	18	58	2		R.ELLIGDR.Q
8	876.5105	875.5032	875.4899	15.3	0	19	49	2		R.QMSLLLR.R + Oxidation (M)
10	892.5034	891.4962	891.4814	16.6	0	31	3.2	1		K.LELAQYR.E
12	1026.6175	1025.6102	1025.5869	22.7	0	72	0.00012	1		K.AVDSLVPIGR.G
17	1242.6447	1241.6374	1241.6074	24.2	0	32	1.9	1		K.SVHEPMQTGLK.A + Oxidation (M)
21	1537.7854	1536.7781	1536.7361	27.3	0	98	4.6e-07	1		R.EAFPGDVFYLHSR.L
28	1967.1528	1966.1455	1966.1139	16.1	1	84	4.8e-06	1		R.LTEVLKQPQYAPLPIEK.Q
37	2308.2314	2307.2242	2307.1495	32.4	0	138	2.8e-11	1		R.EVAAFAQFGSDLDAATQALLNR.G

13. gi|3273574 Mass: 45419 Score: 524 Matches: 11(5) Sequences: 11(5)

F1-ATPase alpha subunit [*Piper nigrum*]

Query	Observed	Mr(expt)	Mr(calc)	ppm	Miss	Score	Expect	Rank	Unique	Peptide
2	755.4491	754.4418	754.4337	10.7	0	33	1	1		K.APGIIER.K
6	815.4770	814.4697	814.4548	18.2	0	18	58	2		R.ELIIGDR.Q
8	876.5105	875.5032	875.4899	15.3	0	19	49	2		R.QMSLLLR.R + Oxidation (M)
9	884.4394	883.4321	883.4148	19.6	0	18	44	3	U	R.GAISDHER.R
10	892.5034	891.4962	891.4814	16.6	0	31	3.2	1		K.LELAQYR.E
12	1026.6175	1025.6102	1025.5869	22.7	0	72	0.00012	1		K.AVDSLVPIGR.G
14	1210.7082	1209.7010	1209.6718	24.1	0	64	0.001	1		R.VVDALGVPIDGR.G
17	1242.6447	1241.6374	1241.6074	24.2	0	32	1.9	1		K.SVHEPMQTGLK.A + Oxidation (M)
19	1300.7765	1299.7693	1299.7398	22.7	0	63	0.00099	1		K.TAIAIDTILNQK.Q
21	1537.7854	1536.7781	1536.7361	27.3	0	98	4.6e-07	1		R.EAFPGDVFYLHSR.L
30	2141.1138	2140.1065	2140.0510	25.9	0	79	2.2e-05	1		R.VYGLNEIQAGEMVEFASGVK.G

14. gi|34539415 Mass: 44989 Score: 520 Matches: 11(5) Sequences: 11(5)

F1-ATPase alpha subunit [*Pleea tenuifolia*]

Query	Observed	Mr(expt)	Mr(calc)	ppm	Miss	Score	Expect	Rank	Unique	Peptide
2	755.4491	754.4418	754.4337	10.7	0	33	1	1		K.APGIIER.K
6	815.4770	814.4697	814.4548	18.2	0	18	58	2		R.ELIIGDR.Q
8	876.5105	875.5032	875.4899	15.3	0	19	49	2		R.QMSLLLR.R + Oxidation (M)
9	884.4394	883.4321	883.4148	19.6	0	18	44	3		R.GALSDHER.R
10	892.5034	891.4962	891.4814	16.6	0	31	3.2	1		K.LELAQYR.E

12	1026.6175	1025.6102	1025.5869	22.7	0	72	0.00012	1		K.AVDSLVPIGR.G
14	1210.7082	1209.7010	1209.6718	24.1	0	64	0.001	1	U	R.VVDALGVPIDGR.G
17	1242.6447	1241.6374	1241.6074	24.2	0	32	1.9	1		K.SVHEPMQTGLK.A + Oxidation (M)
19	1300.7765	1299.7693	1299.7398	22.7	0	63	0.00099	1		K.TAIAIDTILNQK.Q
21	1537.7854	1536.7781	1536.7361	27.3	0	98	4.6e-07	1		R.EAFPGDVFYLHSR.L
30	2141.1138	2140.1065	2140.0510	25.9	0	79	2.2e-05	1		R.VYGINEIQAGEMVEFASGVK.G

Proteins matching the same set of peptides:

gi|34539379 Mass: 44993 Score: 519 Matches: 11(5) Sequences: 11(5)

F1-ATPase alpha subunit [*Narthecium ossifragum*]

15. gi|34539401 Mass: 44859 Score: 511 Matches: 11(5) Sequences: 11(5)

F1-ATPase alpha subunit [*Petermannia cirrosa*]

Query	Observed	Mr(expt)	Mr(calc)	ppm	Miss	Score	Expect	Rank	Unique	Peptide
2	755.4491	754.4418	754.4337	10.7	0	33	1	1		K.APGIIER.K
6	815.4770	814.4697	814.4548	18.2	0	18	58	2		R.ELIIGDR.Q
8	876.5105	875.5032	875.4899	15.3	0	19	49	2		R.QMSLLLR.R + Oxidation (M)
9	884.4394	883.4321	883.4148	19.6	0	18	44	3		R.GALSDHER.R
10	892.5034	891.4962	891.4814	16.6	0	31	3.2	1		K.LELAQYR.E
12	1026.6175	1025.6102	1025.5869	22.7	0	72	0.00012	1		K.AVDSLVPIGR.G
14	1210.7082	1209.7010	1209.6718	24.1	0	64	0.001	1		R.VVDALGVPIDGR.G
17	1242.6447	1241.6374	1241.6074	24.2	0	32	1.9	1		K.SVHEPMQTGLK.A + Oxidation (M)
19	1300.7765	1299.7693	1299.7398	22.7	0	63	0.00099	1		K.TAIAIDTILNQK.Q
21	1537.7854	1536.7781	1536.7361	27.3	0	98	4.6e-07	1		R.EAFPGDVFYLHSR.L
37	2308.2314	2307.2242	2307.1131	48.1	0	65	0.0005	5	U	R.EVAAFAQFGSDLDSATQALPNR.G

16. gi|20799692 Mass: 43472 Score: 504 Matches: 10(5) Sequences: 10(5)

ATPase alpha subunit [*Hydnora africana*]

Query	Observed	Mr(expt)	Mr(calc)	ppm	Miss	Score	Expect	Rank	Unique	Peptide
2	755.4491	754.4418	754.4337	10.7	0	33	1	1		K.APGIIER.Q
6	815.4770	814.4697	814.4548	18.2	0	18	58	2		R.ELIIGDR.Q
8	876.5105	875.5032	875.4899	15.3	0	19	49	2		R.QMSLLLR.R + Oxidation (M)
10	892.5034	891.4962	891.4814	16.6	0	31	3.2	1		K.LELAQYR.E
11	972.5626	971.5553	971.5400	15.7	0	28	5.7	2		R.VVSVGDGIAR.V
12	1026.6175	1025.6102	1025.5869	22.7	0	72	0.00012	1		K.AVDSLVPIGR.G
14	1210.7082	1209.7010	1209.6718	24.1	0	64	0.001	1		R.VVDALGVPLDGR.G
19	1300.7765	1299.7693	1299.7398	22.7	0	63	0.00099	1		K.TAIAIDTILNQK.Q
21	1537.7854	1536.7781	1536.7361	27.3	0	98	4.6e-07	1		R.EAFPGDVFYLHSR.L
30	2141.1138	2140.1065	2140.0510	25.9	0	79	2.2e-05	1		R.VYGLNEIQAGEMVEFASGVK.G

17. gi|55976886 Mass: 44570 Score: 496 Matches: 11(4) Sequences: 11(4)

F1-ATPase alpha subunit [*Rafflesia tuan-mudae*]

Query	Observed	Mr(expt)	Mr(calc)	ppm	Miss	Score	Expect	Rank	Unique	Peptide
2	755.4491	754.4418	754.4337	10.7	0	33	1	1		K.APGIIER.K
6	815.4770	814.4697	814.4548	18.2	0	18	58	2		R.ELIIGDR.Q
8	876.5105	875.5032	875.4899	15.3	0	19	49	2		R.QMSLLLR.R + Oxidation (M)
9	884.4394	883.4321	883.4148	19.6	0	18	44	3		R.GALSDHER.R
10	892.5034	891.4962	891.4814	16.6	0	31	3.2	1		K.LELAQYR.E

11	972.5626	971.5553	971.5400	15.7	0	28	5.7	2		-.VVSVGDGIAR.V
12	1026.6175	1025.6102	1025.5869	22.7	0	72	0.00012	1		K.AVDSLVPIGR.G
14	1210.7082	1209.7010	1209.6718	24.1	0	64	0.001	1		R.VVDALGVPLDGR.G
17	1242.6447	1241.6374	1241.6074	24.2	0	32	1.9	1		K.SVHEPMQTGLK.A + Oxidation (M)
21	1537.7854	1536.7781	1536.7361	27.3	0	98	4.6e-07	1		R.EAFPGDVFYLHSR.L
28	1967.1528	1966.1455	1966.1139	16.1	1	84	4.8e-06	1		R.LTEVLKQPQYAPLPIEK.Q

Proteins matching the same set of peptides:

gi|55976888 Mass: 45335 Score: 496 Matches: 11(4) Sequences: 11(4)

F1-ATPase alpha subunit [*Rafflesia pricei*]

18. gi|34539443 Mass: 44731 Score: 489 Matches: 10(5) Sequences: 10(5)

F1-ATPase alpha subunit [*Stauntonia hexaphylla*]

Query	Observed	Mr(expt)	Mr(calc)	ppm	Miss	Score	Expect	Rank	Unique	Peptide
6	815.4770	814.4697	814.4548	18.2	0	18	58	2		R.ELIIGDR.Q
8	876.5105	875.5032	875.4899	15.3	0	19	49	2		R.QMSLLLR.R + Oxidation (M)
9	884.4394	883.4321	883.4148	19.6	0	18	44	3		R.GALSDHER.R
10	892.5034	891.4962	891.4814	16.6	0	31	3.2	1		K.LELAQYR.E
12	1026.6175	1025.6102	1025.5869	22.7	0	72	0.00012	1		K.AVDSLVPIGR.G
14	1210.7082	1209.7010	1209.6718	24.1	0	64	0.001	1		R.VVDALGVPIDGR.G
17	1242.6447	1241.6374	1241.6438	-5.14	1	32	1.9	1	U	K.SVHEPMKTGLK.A + Oxidation (M)
19	1300.7765	1299.7693	1299.7398	22.7	0	63	0.00099	1	U	K.TAIAIDTILNQK.Q
21	1537.7854	1536.7781	1536.7361	27.3	0	98	4.6e-07	1		R.EAFPGDVFYLHSR.L
30	2141.1138	2140.1065	2140.0510	25.9	0	79	2.2e-05	1		R.VYGLNEIQAGEMVEFASGVK.G

19. gi|34539463 Mass: 43197 Score: 452 Matches: 11(4) Sequences: 11(4)

F1-ATPase alpha subunit [*Tofieldia calyculata*]

Query	Observed	Mr(expt)	Mr(calc)	ppm	Miss	Score	Expect	Rank	Unique	Peptide
2	755.4491	754.4418	754.4337	10.7	0	33	1	1		K.APGIIER.K
6	815.4770	814.4697	814.4548	18.2	0	18	58	2		R.ELIIGDR.Q
8	876.5105	875.5032	875.4899	15.3	0	19	49	2		R.QMSLLLR.R + Oxidation (M)
9	884.4394	883.4321	883.4148	19.6	0	18	44	3		R.GALSDHER.R
10	892.5034	891.4962	891.4814	16.6	0	31	3.2	1		K.LELAQYR.E
12	1026.6175	1025.6102	1025.5869	22.7	0	72	0.00012	1		K.AVDSLVPIGR.G
14	1210.7082	1209.7010	1209.6718	24.1	0	64	0.001	1	U	R.VVDALGVPIDGR.G
17	1242.6447	1241.6374	1241.6074	24.2	0	32	1.9	1		K.SVHEPMQTGLK.A + Oxidation (M)
19	1300.7765	1299.7693	1299.7398	22.7	0	63	0.00099	1		K.TAIAIDTILNQK.Q
21	1537.7854	1536.7781	1536.7361	27.3	0	98	4.6e-07	1		R.EAFPGDVFYLHSR.L
33	2163.1097	2162.1025	2162.0869	7.21	1	12	1.1e+02	6	U	R.VWDALGVPIDGRGALSDHER.R

Proteins matching the same set of peptides:

gi|312145150 Mass: 37994 Score: 451 Matches: 11(4) Sequences: 11(4)

ATPase subunit 1 [*Tofieldia pusilla*]

20. gi|164685618 Mass: 44664 Score: 447 Matches: 10(4) Sequences: 10(4)

atp1, partial (mitochondrion) [*Krameria lanceolata*]

Query	Observed	Mr(expt)	Mr(calc)	ppm	Miss	Score	Expect	Rank	Unique	Peptide
2	755.4491	754.4418	754.4337	10.7	0	33	1	1		K.APGIIER.K
6	815.4770	814.4697	814.4548	18.2	0	18	58	2		R.ELIIGDR.Q

8	876.5105	875.5032	875.4899	15.3	0	19	49	2		R.QMSLLLR.R + Oxidation (M)
9	884.4394	883.4321	883.4148	19.6	0	18	44	3		R.GALSDHER.R
10	892.5034	891.4962	891.4814	16.6	0	31	3.2	1		K.LELAQYR.E
12	1026.6175	1025.6102	1025.5869	22.7	0	72	0.00012	1		K.AVDSLVPIGR.G
14	1210.7082	1209.7010	1209.6718	24.1	0	64	0.001	1		R.VVDALGVPLDGR.G
17	1242.6447	1241.6374	1241.6074	24.2	0	32	1.9	1		K.SVHEPMQTGLK.A + Oxidation (M)
19	1300.7765	1299.7693	1299.7398	22.7	0	63	0.00099	1		K.TAIAIDTILNQK.Q
21	1537.7854	1536.7781	1536.7361	27.3	0	98	4.6e-07	1		R.EAFPGDVFYLHSR.L

21. gi|34539457 Mass: 44893 Score: 443 Matches: 10(4) Sequences: 10(4)

F1-ATPase alpha subunit [*Zephyra cyanocrocus*]

Query	Observed	Mr(expt)	Mr(calc)	ppm	Miss	Score	Expect	Rank	Unique	Peptide
2	755.4491	754.4418	754.4337	10.7	0	33	1	1		K.APGIIER.K
6	815.4770	814.4697	814.4548	18.2	0	18	58	2		R.ELIIGDR.Q
8	876.5105	875.5032	875.4899	15.3	0	19	49	2		R.QMSLLLR.R + Oxidation (M)
9	884.4394	883.4321	883.4148	19.6	0	18	44	3		R.GALSDHER.R
10	892.5034	891.4962	891.4814	16.6	0	31	3.2	1		K.LELAQYR.E
12	1026.6175	1025.6102	1025.5869	22.7	0	72	0.00012	1		K.AVDSIVPIGR.G
14	1210.7082	1209.7010	1209.6718	24.1	0	64	0.001	1		R.VVDALGVPIDGR.G
17	1242.6447	1241.6374	1241.6074	24.2	0	32	1.9	1		K.SVHEPMQTGLK.A + Oxidation (M)
19	1300.7765	1299.7693	1299.7398	22.7	0	63	0.00099	1		K.TAIAIDTILNQK.Q
21	1537.7854	1536.7781	1536.7361	27.3	0	98	4.6e-07	1		R.EAFPGDVFYLHSR.L

22. gi|3273598 Mass: 45285 Score: 425 Matches: 10(4) Sequences: 10(4)

F1-ATPase alpha subunit [*Veratrum viride*]

Query	Observed	Mr(expt)	Mr(calc)	ppm	Miss	Score	Expect	Rank	Unique	Peptide
2	755.4491	754.4418	754.4337	10.7	0	33	1	1		K.APGIIER.K
6	815.4770	814.4697	814.4548	18.2	0	18	58	2		R.ELIIGDR.Q
8	876.5105	875.5032	875.4899	15.3	0	19	49	2		R.QMSLLLR.R + Oxidation (M)
9	884.4394	883.4321	883.4148	19.6	0	18	44	3		R.GALSDHER.R
10	892.5034	891.4962	891.4814	16.6	0	31	3.2	1		K.LELAQYR.E
12	1026.6175	1025.6102	1025.5869	22.7	0	72	0.00012	1		K.AVDSIVPIGR.G
14	1210.7082	1209.7010	1209.6718	24.1	0	64	0.001	1		R.VVDALGVPIDGR.G
17	1242.6447	1241.6374	1241.6074	24.2	0	32	1.9	1		K.SVHEPMQTGLK.A + Oxidation (M)
19	1300.7765	1299.7693	1299.7398	22.7	0	63	0.00099	1	U	K.TAIAIDTILNQK.Q
30	2141.1138	2140.1065	2140.0510	25.9	0	79	2.2e-05	1		R.VYGLNEIQAGEMVEFASGVK.G

23. gi|133853924 Mass: 34928 Score: 416 Matches: 9(4) Sequences: 9(4)

F1-ATPase complex subunit [*Silene vulgaris*]

Query	Observed	Mr(expt)	Mr(calc)	ppm	Miss	Score	Expect	Rank	Unique	Peptide
2	755.4491	754.4418	754.4337	10.7	0	33	1	1		K.APGILER.K
6	815.4770	814.4697	814.4548	18.2	0	18	58	2		R.ELIIGDR.Q
8	876.5105	875.5032	875.4899	15.3	0	19	49	2		R.QMSLLLR.R + Oxidation (M)
9	884.4394	883.4321	883.4148	19.6	0	18	44	3		R.GALSDHER.R
12	1026.6175	1025.6102	1025.5869	22.7	0	72	0.00012	1		K.AVDSLVPIGR.G

14	1210.7082	1209.7010	1209.6718	24.1	0	64	0.001	1		R.VVDALGVPIDGR.G
17	1242.6447	1241.6374	1241.6074	24.2	0	32	1.9	1		K.SVHEPMQTGLK.A + Oxidation (M)
19	1300.7765	1299.7693	1299.7398	22.7	0	63	0.00099	1		K.TAIAIDTILNQK.Q
21	1537.7854	1536.7781	1536.7361	27.3	0	98	4.6e-07	1		R.EAFPGDVFYLHSR.L

Proteins matching the same set of peptides:

gi|302747368 Mass: 37211 Score: 416 Matches: 9(4) Sequences: 9(4)

Atp1 [*Bursera sp. Qiu 94206*]

gi|302747598 Mass: 37607 Score: 416 Matches: 9(4) Sequences: 9(4)

Atp1 [*Pittosporum tobira*]

gi|302747662 Mass: 37555 Score: 416 Matches: 9(4) Sequences: 9(4)

Atp1 [*Swietenia macrophylla*]

24. gi|302747658 Mass: 37038 Score: 416 Matches: 9(4) Sequences: 9(4)

Atp1 [*Strasburgeria robusta*]

Query	Observed	Mr(expt)	Mr(calc)	ppm	Miss	Score	Expect	Rank	Unique	Peptide
2	755.4491	754.4418	754.4337	10.7	0	33	1	1		K.APGIIER.K
6	815.4770	814.4697	814.4548	18.2	0	18	58	2		R.ELIIGDR.Q
8	876.5105	875.5032	875.4899	15.3	0	19	49	2		R.QMSLLLR.R + Oxidation (M)
9	884.4394	883.4321	883.4148	19.6	0	18	44	3		R.GALSDHER.R
12	1026.6175	1025.6102	1025.5869	22.7	0	72	0.00012	1		K.AVDSLVPIGR.G
14	1210.7082	1209.7010	1209.6718	24.1	0	64	0.001	1		R.VVDALGVPIDGR.G
17	1242.6447	1241.6374	1241.6074	24.2	0	32	1.9	1		K.SVHEPMQTGLK.A + Oxidation (M)
19	1300.7765	1299.7693	1299.7398	22.7	0	63	0.00099	1		K.TAIAIDTIINQK.Q
21	1537.7854	1536.7781	1536.7361	27.3	0	98	4.6e-07	1		R.EAFPGDVFYLHSR.L

25. gi|113197031 Mass: 44924 Score: 412 Matches: 9(4) Sequences: 9(4)

F1-ATPase alpha subunit [*Epipactis helleborine*]

Query	Observed	Mr(expt)	Mr(calc)	ppm	Miss	Score	Expect	Rank	Unique	Peptide
2	755.4491	754.4418	754.4337	10.7	0	33	1	1		K.APGIIER.Q
6	815.4770	814.4697	814.4548	18.2	0	18	58	2		R.ELIIGDR.Q
8	876.5105	875.5032	875.4899	15.3	0	19	49	2		R.QMSLLLR.R + Oxidation (M)
9	884.4394	883.4321	883.4148	19.6	0	18	44	3		R.GALSDHER.R
10	892.5034	891.4962	891.4814	16.6	0	31	3.2	1		K.LELAQYR.E
12	1026.6175	1025.6102	1025.5869	22.7	0	72	0.00012	1		K.AVDSLVPIGR.G
14	1210.7082	1209.7010	1209.6718	24.1	0	64	0.001	1	U	R.VVDAIGVPIDGR.G
19	1300.7765	1299.7693	1299.7398	22.7	0	63	0.00099	1		K.TAIAIDTILNQK.Q
21	1537.7854	1536.7781	1536.7361	27.3	0	98	4.6e-07	1		R.EAFPGDVFYLHSR.L

26. gi|114509214 Mass: 39116 Score: 366 Matches: 8(3) Sequences: 8(3)

ATPase alpha subunit [*Najas guadalupensis*]

Query	Observed	Mr(expt)	Mr(calc)	ppm	Miss	Score	Expect	Rank	Unique	Peptide
2	755.4491	754.4418	754.4337	10.7	0	33	1	1		K.APGILER.R
6	815.4770	814.4697	814.4548	18.2	0	18	58	2		R.ELIIGDR.Q
8	876.5105	875.5032	875.4899	15.3	0	19	49	2		R.QMSLLLR.R + Oxidation (M)
10	892.5034	891.4962	891.4814	16.6	0	31	3.2	1		K.LELAQYR.E
12	1026.6175	1025.6102	1025.5869	22.7	0	72	0.00012	1		K.AVDSLVPIGR.G
14	1210.7082	1209.7010	1209.6718	24.1	0	64	0.001	1		R.VVDALGVPIDGR.G

17	1242.6447	1241.6374	1241.6074	24.2	0	32	1.9	1		R.SVHEPMQTGLK.A + Oxidation (M)
21	1537.7854	1536.7781	1536.7361	27.3	0	98	4.6e-07	1		R.EAFPGDVFYLHSR.L

27. gi|150406456 Mass: 55686 Score: 334 Matches: 7(3) Sequences: 7(3)

ATP synthase F1 subunit alpha [*Chlorokybus atmophyticus*]

Query	Observed	Mr(expt)	Mr(calc)	ppm	Miss	Score	Expect	Rank	Unique	Peptide
6	815.4770	814.4697	814.4548	18.2	0	18	58	2		R.ELIIGDR.Q
8	876.5105	875.5032	875.4899	15.3	0	19	49	2		R.QMSLLLR.R + Oxidation (M)
10	892.5034	891.4962	891.4814	16.6	0	31	3.2	1		K.LELAQYR.E
12	1026.6175	1025.6102	1025.5869	22.7	0	72	0.00012	1		K.AVDSLVPIGR.G
17	1242.6447	1241.6374	1241.6074	24.2	0	32	1.9	1		K.SVHEPMQTGLK.A + Oxidation (M)
19	1300.7765	1299.7693	1299.7398	22.7	0	63	0.00099	1		K.TAIAIDTIINQK.E
21	1537.7854	1536.7781	1536.7361	27.3	0	98	4.6e-07	1		R.EAFPGDVFYLHSR.L

Proteins matching the same set of peptides:

gi|255088599 Mass: 60061 Score: 334 Matches: 7(3) Sequences: 7(3)

H+-or Na+-translocating f-type, v-type and A-type ATPase superfamily [*Micromonas sp. RCC299*]

gi|303274717 Mass: 60369 Score: 334 Matches: 7(3) Sequences: 7(3)

ATP synthase [*Micromonas pusilla CCMP1545*]

28. gi|475560090 Mass: 52569 Score: 327 Matches: 7(3) Sequences: 7(3)

ATP synthase subunit alpha, mitochondrial [*Aegilops tauschii*]

Query	Observed	Mr(expt)	Mr(calc)	ppm	Miss	Score	Expect	Rank	Unique	Peptide
2	755.4491	754.4418	754.4337	10.7	0	33	1	1		K.APGIIER.L
6	815.4770	814.4697	814.4548	18.2	0	18	58	2		R.ELIIGDR.Q
8	876.5105	875.5032	875.4899	15.3	0	19	49	2		R.QMSLLLR.R + Oxidation (M)
9	884.4394	883.4321	883.4148	19.6	0	18	44	3		K.GALSDHER.R
19	1300.7765	1299.7693	1299.7762	-5.31	1	63	0.00099	1	U	K.TAIAIDTILNKK.Q
21	1537.7854	1536.7781	1536.7361	27.3	0	98	4.6e-07	1		R.EAFPGDVFYLHSR.L
30	2141.1138	2140.1065	2140.0510	25.9	0	79	2.2e-05	1		R.VYGLNEIQAGEMVEFASGVK.G

29. gi|507118370 Mass: 30289 Score: 304 Matches: 5(2) Sequences: 5(2)

Atp1, partial (mitochondrion) [*Gentianella rapunculoides*]

Query	Observed	Mr(expt)	Mr(calc)	ppm	Miss	Score	Expect	Rank	Unique	Peptide
6	815.4770	814.4697	814.4548	18.2	0	18	58	2	U	R.EIIIGDR.Q
8	876.5105	875.5032	875.4899	15.3	0	19	49	2		R.QMSLLLR.R + Oxidation (M)
10	892.5034	891.4962	891.4814	16.6	0	31	3.2	1		K.LELAQYR.E
21	1537.7854	1536.7781	1536.7361	27.3	0	98	4.6e-07	1		R.EAFPGDVFYLHSR.L
37	2308.2314	2307.2242	2307.1495	32.4	0	138	2.8e-11	1		R.EVAAFAQFGSDLDAATQALLNR.G

Proteins matching the same set of peptides:

gi|27461579 Mass: 44698 Score: 302 Matches: 5(2) Sequences: 5(2)

ATPase F1 alpha subunit, partial (mitochondrion) [*Ecdeiocolea monostachya*]

30. gi|110225671 Mass: 55085 Score: 304 Matches: 7(2) Sequences: 7(2)

ATP synthase F1 subunit alpha [*Nephroselmis olivacea*]

Query	Observed	Mr(expt)	Mr(calc)	ppm	Miss	Score	Expect	Rank	Unique	Peptide
2	755.4491	754.4418	754.4337	10.7	0	33	1	1		K.APGIIER.K
6	815.4770	814.4697	814.4548	18.2	0	18	58	2		R.ELIIGDR.Q

8	876.5105	875.5032	875.4899	15.3	0	19	49	2		R.QMSLLLR.R + Oxidation (M)
10	892.5034	891.4962	891.4814	16.6	0	31	3.2	1		K.LELAQYR.E
12	1026.6175	1025.6102	1025.5869	22.7	0	72	0.00012	1		K.AVDSLVPIGR.G
17	1242.6447	1241.6374	1241.6074	24.2	0	32	1.9	1	U	K.SVHEPMQTGIK.A + Oxidation (M)
21	1537.7854	1536.7781	1536.7361	27.3	0	98	4.6e-07	1		R.EAFPGDVFYLHSR.L

Database 1

Match to: Unigene89272_SeCKS transcribed RNA sequence Mass: 19896 Score: 509 Matches: 10(7) Sequences: 10(7)

gi|22742 pseudo-atpA [*Glycine max*]

Matched peptides shown in **bold red**.

IMEFSPR**AAELTTLLESRITNFYTNFQVDEIGRVVSVGDGIARVYGLNEIQAGEMVEFASGVK**GIALNLENENVGIVVFGSDTAI

KEGDLVKRTGSIVDVPAGKAMLGR**VVDALGVPIDGRGALSDHER**RRVEVK**APGIIER**K**SVHEPMQTGLKAVDSLVPIGR**GQR**E**

LIIGDRQTGKTAIAIDTI

Query	Observed	Mr(expt)	Mr(calc)	ppm	Miss	Score	Expect	Rank	Unique	Peptide
2	755.4491	754.4418	754.4337	10.7	0	33	0.013	1	U	K.APGIIER.K
6	815.4770	814.4697	814.4548	18.2	0	18	1	1		R.ELIIGDR.Q
9	884.4394	883.4321	883.4148	19.6	0	18	0.66	1	U	R.GALSDHER.R
11	972.5626	971.5553	971.5400	15.7	0	28	0.097	1	U	R.VVSVGDGIAR.V
12	1026.6175	1025.6102	1025.5869	22.7	0	72	1.8e-006	1	U	K.AVDSLVPIGR.G
13	1203.6928	1202.6855	1202.6506	29.0	0	53	0.00022	1	U	R.AAELTTLLESR.I
14	1210.7082	1209.7010	1209.6718	24.1	0	64	1.1e-005	1	U	R.VVDALGVPIDGR.G
17	1242.6447	1241.6374	1241.6074	24.2	0	32	0.033	1	U	K.SVHEPMQTGLK.A + Oxidation (M)
27	1816.9327	1815.9255	1815.8792	25.5	0	113	2.6e-010	1	U	R.ITNFYTNFQVDEIGR.V
30	2141.1138	2140.1065	2140.0510	25.9	0	79	4.5e-007	1	U	R.VYGLNEIQAGEMVEFASGVK.G

Proteins matching the same set of peptides:

Unigene89273_SeCKS transcribed RNA sequence Mass: 19896 Score: 509 Matches: 10(7) Sequences: 10(7)

gi| 22742 pseudo-atpA [*Glycine max*]

2. Unigene55779_Se200S transcribed RNA sequence Mass: 20305 Score: 298 Matches: 4(4) Sequences: 4(4)

gi|162279935 atp1 gene product (mitochondrion) [*Beta vulgaris subsp. vulgaris*]

Query	Observed	Mr(expt)	Mr(calc)	ppm	Miss	Score	Expect	Rank	Unique	Peptide
10	892.5034	891.4962	891.4814	16.6	0	31	0.049	1	U	K.LELAQYR.E
25	1725.0490	1724.0417	1724.0083	19.4	0	46	0.00019	1	U	K.TILNSVKPELLQELK.G
28	1967.1528	1966.1455	1966.1139	16.1	1	84	4.3e-008	1	U	R.LTEVLKQPQYAPLPIEK.Q
37	2308.2314	2307.2242	2307.1495	32.4	0	138	4.9e-013	1	U	R.EVAAFAQFGSDLDAATQALLNR.G

Database 2

Match to: Unigene16541_SALfmcTARAAPEI-3 Mass: 20907 Score: 345 Matches: 13(10) Sequences: 12(10)

gi|22742 pseudo-atpA [*Glycine max*]

Matched peptides shown in **bold red**.

MEFSPRAAELTTLLESRITNFYTNFQVDEIGRVVSVGDGIARVYGLNEIQAGEMVEFASGVKGIALNLENENVGIVVFGSDTAIK

EGDLVKRTGSIVDVPAGKAMLGR**VVDALGVPIDGRGALSDHER**RRVEVK**APGIIER**K**SVHEPMQTGLKAVDSLVPIGR**GQR**ELI**

IGDRQTGKTAIAIDTILNQKQLNSKA

Query	Observed	Mr(expt)	Mr(calc)	ppm	Miss	Score	Expect	Rank	Unique	Peptide
2	755.4491	754.4418	754.4337	10.7	0	33	0.0086	1	U	K.APGIIER.K
3	766.3632	765.3560	765.3479	10.5	0	(24)	0.1	1	U	-.MEFSPR.A
4	782.3536	781.3463	781.3429	4.46	0	31	0.015	1	U	-.MEFSPR.A + Oxidation (M)
6	815.4770	814.4697	814.4548	18.2	0	18	0.48	1		R.ELIIGDR.Q
9	884.4394	883.4321	883.4148	19.6	0	18	0.37	1	U	R.GALSDHER.R
11	972.5626	971.5553	971.5400	15.7	0	28	0.048	1	U	R.VVSVGDGIAR.V
12	1026.6175	1025.6102	1025.5869	22.7	0	72	1.1e-006	1	U	K.AVDSLVPIGR.G
13	1203.6928	1202.6855	1202.6506	29.0	0	53	0.00013	1	U	R.AAELTTLLESR.I
14	1210.7082	1209.7010	1209.6718	24.1	0	64	7.9e-006	1	U	R.VVDALGVPIDGR.G
17	1242.6447	1241.6374	1241.6074	24.2	0	32	0.016	1	U	K.SVHEPMQTGLK.A + Oxidation (M)
19	1300.7765	1299.7693	1299.7398	22.7	0	63	8e-006	1	U	K.TAIAIDTILNQK.Q
27	1816.9327	1815.9255	1815.8792	25.5	0	113	1.1e-010	1	U	R.ITNFYTNFQVDEIGR.V
30	2141.1138	2140.1065	2140.0510	25.9	0	79	1.8e-007	1	U	R.VYGLNEIQAGEMVEFASGVK.G

2. Unigene53297_SALfmcTARAAPEI-3 Mass: 33461 Score: 298 Matches: 6(5) Sequences: 6(5)

gi|162279935 atp1 gene product (mitochondrion) [*Beta vulgaris subsp. vulgaris*]

Query	Observed	Mr(expt)	Mr(calc)	ppm	Miss	Score	Expect	Rank	Unique	Peptide
8	876.5105	875.5032	875.4899	15.3	0	19	0.45	1		R.QMSLLLR.R + Oxidation (M)
10	892.5034	891.4962	891.4814	16.6	0	31	0.029	1	U	K.LELAQYR.E
21	1537.7854	1536.7781	1536.7361	27.3	0	98	4.2e-009	1	U	R.EAFPGDVFYLHSR.L
25	1725.0490	1724.0417	1724.0083	19.4	0	46	0.00023	1	U	K.TILNSVKPELLQELK.G
28	1967.1528	1966.1455	1966.1139	16.1	1	84	3.7e-008	1	U	R.LTEVLKQPQYAPLPIEK.Q
37	2308.2314	2307.2242	2307.1495	32.4	0	138	2.3e-013	1	U	R.EVAAFAQFGSDLDAATQALLNR.G

Spot 774

NCBInr protein database

Match to: gi|372450254 Mass: 55602 Score: 555 Matches: 9(5) Sequences: 9(5)

ATPase subunit 1 (mitochondrion) [*Millettia pinnata*]

Matched peptides shown in bold red.

1 MDFSVRAAEL TTLLESRITN FYTNFQVDEI GRVVSVGDGI ARVYGLNEIQ

51 AGEMVEFASG VKGVALNLEN ENVGIIVFGS DTAIKEGDLV KRTGSIVDVP

101 AGKAMLGRVV DALGVPIDGR GALSDHERRR VDVKAPGIIE RKSVHEPMQT

151 GLKAVDSLVP IGRGQRELII GDRQTGKTAI AIDTILNQKQ MNSRATSESE

201 TLYCVYVAIG QKRSTVAQLV QILSEANALE YSILVAATAS DPAPLQYLAP

251 YSGCAMGEYF RDNGMHALII YDDLSKQAVA YRQMSLLLRR PPGREAFPGD

301 VFYLHSRLLE RAAKRSDQTG AGSLTALPVI ETQAGDVSAY IPTNVISITD

351 GQICLETELF YRGIRPAINV GLSVSRVGSA AQLKAMKQVC GSLKLELAQY

401 REVAAFAQFG SDLDAATQAL LNRGARLTEV LKQPQYAPLP IEKQILVIYA

451 AVNGFCDRMP LEKIPQYERD ILTTIKPELL QSLKGVLNSE RKIELDAFLK

501 EKAKTYLI

Query	Observed	Mr(expt)	Mr(calc)	ppm	Miss	Score	Expect	Rank	Unique	Peptide

1	755.4208	754.4136	754.4337	-26.73	0	16	61	5		K.APGIIER.K
5	892.4621	891.4548	891.4814	-29.78	0	19	52	1		K.LELAQYR.E
6	1026.5668	1025.5595	1025.5869	-26.73	0	41	0.24	1		K.AVDSLVPIGR.G
8	1203.6340	1202.6267	1202.6506	-19.89	0	37	0.72	1	U	R.AAELTTLLESR.I
9	1210.6586	1209.6513	1209.6718	-16.87	0	61	0.0024	2		R.VVDALGVPIDGR.G
14	1537.7089	1536.7016	1536.7361	-22.46	0	84	9.5e-06	1		R.EAFPGDVFYLHSR.L
15	1816.8493	1815.8420	1815.8792	-20.47	0	111	1.8e-08	1	U	R.ITNFYTNFQVDEIGR.V
16	1967.0756	1966.0683	1966.1139	-23.18	1	78	4.4e-05	1		R.LTEVLKQPQYAPLPIEK.Q
25	2308.1348	2307.1275	2307.1495	-9.54	0	108	2.6e-08	1		R.EVAAFAQFGSDLDAATQALLNR.G

Proteins matching the same set of peptides:

gi|357982 Mass: 55326 Score: 553 Matches: 9(5) Sequences: 9(5)

ATPase alpha,F1

gi|543866 Mass: 55296 Score: 553 Matches: 9(5) Sequences: 9(5)

RecName: Full=ATP synthase subunit alpha, mitochondrial

gi|357436047 Mass: 93692 Score: 553 Matches: 9(5) Sequences: 9(5)

ATP synthase subunit alpha [*Medicago truncatula*]

gi|476507691 Mass: 55581 Score: 553 Matches: 9(5) Sequences: 9(5)

ATPase subunit 1 (mitochondrion) [*Glycine max*]

gi|576303588 Mass: 55721 Score: 553 Matches: 9(5) Sequences: 9(5)

ATPase subunit 1 (mitochondrion) [*Vaccinium macrocarpon*]

gi|372450305 Mass: 55158 Score: 552 Matches: 9(5) Sequences: 9(5)

ATPase subunit 1 (mitochondrion) [*Lotus japonicus*]

2. gi|50058567 Mass: 46474 Score: 500 Matches: 8(5) Sequences: 8(5)

F1-ATPase alpha subunit [Astripomoea malvacea]

Query	Observed	Mr(expt)	Mr(calc)	ppm	Miss	Score	Expect	Rank	Unique	Peptide
1	755.4208	754.4136	754.4337	-26.73	0	16	61	5		K.APGIIER.K
5	892.4621	891.4548	891.4814	-29.78	0	19	52	1		K.LELAQYR.E
6	1026.5668	1025.5595	1025.5869	-26.73	0	41	0.24	1		K.AVDSLVPIGR.G
9	1210.6586	1209.6513	1209.6718	-16.87	0	61	0.0024	2		R.VVDALGVPIDGR.G
14	1537.7089	1536.7016	1536.7361	-22.46	0	84	9.5e-06	1		R.EAFPGDVFYLHSR.L
16	1967.0756	1966.0683	1966.1139	-23.18	1	78	4.4e-05	1		R.LTEVLKQPQYAPLPIEK.Q
25	2308.1348	2307.1275	2307.1495	-9.54	0	108	2.6e-08	1		R.EVAAFAQFGSDLDAATQALLNR.G
27	3170.6694	3169.6622	3169.6619	0.08	1	95	3.4e-07	1	U	K.GIALNLENENVGIVVFGSDTAIKEGDLVQR.T

Proteins matching the same set of peptides:

gi|50058579 Mass: 46315 Score: 500 Matches: 8(5) Sequences: 8(5)

F1-ATPase alpha subunit [*Iseia luxurians*]

3. gi|223667959 Mass: 45842 Score: 422 Matches: 7(4) Sequences: 7(4)

F1-ATPase alpha subunit [*Cleistes sp. Chase O-430*]

Query	Observed	Mr(expt)	Mr(calc)	ppm	Miss	Score	Expect	Rank	Unique	Peptide
1	755.4208	754.4136	754.4337	-26.73	0	16	61	5		K.APGIIER.Q
5	892.4621	891.4548	891.4814	-29.78	0	19	52	1		K.LELAQYR.E
6	1026.5668	1025.5595	1025.5869	-26.73	0	41	0.24	1		K.AVDSLVPIGR.G

9	1210.6586	1209.6513	1209.6718	-16.87	0	61	0.0024	2		R.VVDALGVPIDGR.G
14	1537.7089	1536.7016	1536.7361	-22.46	0	84	9.5e-06	1		R.EAFPGDVFYLHSR.L
25	2308.1348	2307.1275	2307.1495	-9.54	0	108	2.6e-08	1		R.EVAAFAQFGSDLDAATQALLNR.G
27	3170.6694	3169.6622	3169.6619	0.08	1	95	3.4e-07	1	U	K.GIALNLENENVGIVVFGSDTAIQEGDLVKR.T

Proteins matching the same set of peptides:

gi|223667976 Mass: 46540 Score: 422 Matches: 7(4) Sequences: 7(4)

F1-ATPase alpha subunit [*Isotria medeoloides*]

gi|223667978 Mass: 46813 Score: 422 Matches: 7(4) Sequences: 7(4)

F1-ATPase alpha subunit [*Isotria verticillata*]

gi|223667983 Mass: 45632 Score: 422 Matches: 7(4) Sequences: 7(4)

F1-ATPase alpha subunit [*Pogonia japonica*]

gi|223667985 Mass: 44616 Score: 422 Matches: 7(4) Sequences: 7(4)

F1-ATPase alpha subunit [*Pogonia minor*]

4. gi|55976884 Mass: 45248 Score: 373 Matches: 7(3) Sequences: 7(3)

F1-ATPase alpha subunit [*Pilostyles thurberi*]

Query	Observed	Mr(expt)	Mr(calc)	ppm	Miss	Score	Expect	Rank	Unique	Peptide
1	755.4208	754.4136	754.4337	-26.73	0	16	61	5		K.APGIIER.K
5	892.4621	891.4548	891.4814	-29.78	0	19	52	1		K.LELAQYR.E
6	1026.5668	1025.5595	1025.5869	-26.73	0	41	0.24	1		K.AVDSLVPIGR.G
9	1210.6586	1209.6513	1209.6718	-16.87	0	29	4.4	10	U	R.VVDGLGLPIDGR.G
14	1537.7089	1536.7016	1536.7361	-22.46	0	84	9.5e-06	1		R.EAFPGDVFYLHSR.L
16	1967.0756	1966.0683	1966.1139	-23.18	1	78	4.4e-05	1		R.LTEVLKQPQYAPLPIEK.Q
25	2308.1348	2307.1275	2307.1495	-9.54	0	108	2.6e-08	1		R.EVAAFAQFGSDLDAATQALLNR.G

Proteins matching the same set of peptides:

gi|164685324 Mass: 44670 Score: 373 Matches: 7(3) Sequences: 7(3)

atp1, partial (mitochondrion) [*Pilostyles thurberi*]

gi|164685552 Mass: 44700 Score: 373 Matches: 7(3) Sequences: 7(3)

atp1, partial (mitochondrion) [*Pilostyles thurberi*]

5. gi|224020956 Mass: 55447 Score: 350 Matches: 8(3) Sequences: 7(3)

ATP synthase F1 subunit 1 [*Carica papaya*]

Query	Observed	Mr(expt)	Mr(calc)	ppm	Miss	Score	Expect	Rank	Unique	Peptide
1	755.4208	754.4136	754.4337	-26.73	0	16	61	5		K.APGIIER.K
2	766.3342	765.3269	765.3479	-27.49	0	(18)	47	2		-.MEFSPR.A
3	782.3300	781.3227	781.3429	-25.81	0	19	23	1		-.MEFSPR.A + Oxidation (M)
5	892.4621	891.4548	891.4814	-29.78	0	19	52	1		K.LELAQYR.E
6	1026.5668	1025.5595	1025.5869	-26.73	0	41	0.24	1		K.AVDSLVPIGR.G
9	1210.6586	1209.6513	1209.6718	-16.87	0	61	0.0024	2		R.VVDALGVPIDGR.G
14	1537.7089	1536.7016	1536.7361	-22.46	0	84	9.5e-06	1		R.EAFPGDVFYLHSR.L
15	1816.8493	1815.8420	1815.8792	-20.47	0	111	1.8e-08	1	U	R.LTNFYTNFQVDEIGR.V

6. gi|14916970 Mass: 55296 Score: 346 Matches: 6(3) Sequences: 6(3)

RecName: Full=ATP synthase subunit alpha, mitochondrial

Query	Observed	Mr(expt)	Mr(calc)	ppm	Miss	Score	Expect	Rank	Unique	Peptide

1	755.4208	754.4136	754.4337	-26.73	0	16	61	5		K.APGILER.K
5	892.4621	891.4548	891.4814	-29.78	0	19	52	1		K.LELAQYR.E
6	1026.5668	1025.5595	1025.5869	-26.73	0	41	0.24	1		K.AVDSLVPIGR.G
14	1537.7089	1536.7016	1536.7361	-22.46	0	84	9.5e-06	1		R.EAFPGDVFYLHSR.L
16	1967.0756	1966.0683	1966.1139	-23.18	1	78	4.4e-05	1		R.LTEVLKQPQYAPLPIEK.Q
25	2308.1348	2307.1275	2307.1495	-9.54	0	108	2.6e-08	1		R.EVAAFAQFGSDLDAATQALLNR.G

Proteins matching the same set of peptides:

gi|112253900 Mass: 55382 Score: 346 Matches: 6(3) Sequences: 6(3)

ATPase subunit 1 [*Brassica napus*]

7. gi|269819632 Mass: 42993 Score: 330 Matches: 6(3) Sequences: 6(3)

F1-ATPase alpha subunit, partial (mitochondrion) [*Kupea martinetugei*]

Query	Observed	Mr(expt)	Mr(calc)	ppm	Miss	Score	Expect	Rank	Unique	Peptide
1	755.4208	754.4136	754.4337	-26.73	0	16	61	5		K.APGIIER.K
5	892.4621	891.4548	891.4814	-29.78	0	19	52	1		K.LELAQYR.E
6	1026.5668	1025.5595	1025.5869	-26.73	0	41	0.24	1		K.AVDSLVPIGR.G
9	1210.6586	1209.6513	1209.6718	-16.87	0	64	0.0013	1		R.VVDALGVPLDGR.G
14	1537.7089	1536.7016	1536.7361	-22.46	0	84	9.5e-06	1		R.EAFPGDVFYLHSR.L
25	2308.1348	2307.1275	2307.1495	-9.54	0	108	2.6e-08	1		R.EVAAFAQFGSDLDAATQALLNR.G

Proteins matching the same set of peptides:

gi|532219832 Mass: 42541 Score: 330 Matches: 6(3) Sequences: 6(3)

F1-ATPase alpha subunit, partial (mitochondrion) [*Kupea martinetugei*]

8. gi|55976886 Mass: 44570 Score: 300 Matches: 6(3) Sequences: 6(3)

F1-ATPase alpha subunit [*Rafflesia tuan-mudae*]

Query	Observed	Mr(expt)	Mr(calc)	ppm	Miss	Score	Expect	Rank	Unique	Peptide
1	755.4208	754.4136	754.4337	-26.73	0	16	61	5		K.APGIIER.K
5	892.4621	891.4548	891.4814	-29.78	0	19	52	1		K.LELAQYR.E
6	1026.5668	1025.5595	1025.5869	-26.73	0	41	0.24	1		K.AVDSLVPIGR.G
9	1210.6586	1209.6513	1209.6718	-16.87	0	64	0.0013	1		R.VVDALGVPLDGR.G
14	1537.7089	1536.7016	1536.7361	-22.46	0	84	9.5e-06	1		R.EAFPGDVFYLHSR.L
16	1967.0756	1966.0683	1966.1139	-23.18	1	78	4.4e-05	1		R.LTEVLKQPQYAPLPIEK.Q

Proteins matching the same set of peptides:

gi|55976888 Mass: 45335 Score: 300 Matches: 6(3) Sequences: 6(3)

F1-ATPase alpha subunit [*Rafflesia pricei*]

gi|164685556 Mass: 44788 Score: 300 Matches: 6(3) Sequences: 6(3)

atp1, partial (mitochondrion) [*Rafflesia pricei*]

9. gi|34539401 Mass: 44859 Score: 267 Matches: 6(3) Sequences: 6(3)

F1-ATPase alpha subunit [*Petermannia cirrosa*]

Query	Observed	Mr(expt)	Mr(calc)	ppm	Miss	Score	Expect	Rank	Unique	Peptide
1	755.4208	754.4136	754.4337	-26.73	0	16	61	5		K.APGIIER.K
5	892.4621	891.4548	891.4814	-29.78	0	19	52	1		K.LELAQYR.E
6	1026.5668	1025.5595	1025.5869	-26.73	0	41	0.24	1		K.AVDSLVPIGR.G
9	1210.6586	1209.6513	1209.6718	-16.87	0	61	0.0024	2		R.VVDALGVPIDGR.G

14	1537.7089	1536.7016	1536.7361	-22.46	0	84	9.5e-06	1		R.EAFPGDVFYLHSR.L
25	2308.1348	2307.1275	2307.1131	6.23	0	47	0.034	5	U	R.EVAAFAQFGSDLDSATQALPNR.G

10. gi|114509214 Mass: 39116 Score: 220 Matches: 5(2) Sequences: 5(2)

ATPase alpha subunit [*Najas guadalupensis*]

Query	Observed	Mr(expt)	Mr(calc)	ppm	Miss	Score	Expect	Rank	Unique	Peptide
1	755.4208	754.4136	754.4337	-26.73	0	16	61	5		K.APGILER.R
5	892.4621	891.4548	891.4814	-29.78	0	19	52	1		K.LELAQYR.E
6	1026.5668	1025.5595	1025.5869	-26.73	0	41	0.24	1		K.AVDSLVPIGR.G
9	1210.6586	1209.6513	1209.6718	-16.87	0	61	0.0024	2		R.VVDALGVPIDGR.G
14	1537.7089	1536.7016	1536.7361	-22.46	0	84	9.5e-06	1		R.EAFPGDVFYLHSR.L

11. gi|113197031 Mass: 44924 Score: 219 Matches: 5(2) Sequences: 5(2)

F1-ATPase alpha subunit [*Epipactis helleborine*]

Query	Observed	Mr(expt)	Mr(calc)	ppm	Miss	Score	Expect	Rank	Unique	Peptide
1	755.4208	754.4136	754.4337	-26.73	0	16	61	5		K.APGIIER.Q
5	892.4621	891.4548	891.4814	-29.78	0	19	52	1		K.LELAQYR.E
6	1026.5668	1025.5595	1025.5869	-26.73	0	41	0.24	1		K.AVDSLVPIGR.G
9	1210.6586	1209.6513	1209.6718	-16.87	0	61	0.0024	2	U	R.VVDAIGVPIDGR.G
14	1537.7089	1536.7016	1536.7361	-22.46	0	84	9.5e-06	1		R.EAFPGDVFYLHSR.L

12. gi|34539457 Mass: 44893 Score: 218 Matches: 5(2) Sequences: 5(2)

F1-ATPase alpha subunit [*Zephyra cyanocrocus*]

Query	Observed	Mr(expt)	Mr(calc)	ppm	Miss	Score	Expect	Rank	Unique	Peptide
1	755.4208	754.4136	754.4337	-26.73	0	16	61	5		K.APGIIER.K
5	892.4621	891.4548	891.4814	-29.78	0	19	52	1		K.LELAQYR.E
6	1026.5668	1025.5595	1025.5869	-26.73	0	41	0.24	1	U	K.AVDSIVPIGR.G
9	1210.6586	1209.6513	1209.6718	-16.87	0	61	0.0024	2		R.VVDALGVPIDGR.G
14	1537.7089	1536.7016	1536.7361	-22.46	0	84	9.5e-06	1		R.EAFPGDVFYLHSR.L

Database 1

Match to: Unigene89272_SeCKS transcribed RNA sequence Mass: 19896 Score: 264 Matches: 5(4) Sequences: 5(4)

gi|22742 pseudo-atpA [*Glycine max*]

Matched peptides shown in bold red.

IMEFSPRAAELTTLLESRITNFYTNFQVDEIGRVVSVGDGIARVYGLNEIQAGEMVEFASGVKGIALNLENENVGIVVFGSDTAI
KEGDLVKRTGSIVDVPAGKAMLGRVVDALGVPIDGRGALSDHERRRVEVKAPGIIERKSVHEPMQTGLKAVDSLVPIGRGQRE
LIIGDRQTGKTAIAIDTI

Query	Observed	Mr(expt)	Mr(calc)	ppm	Miss	Score	Expect	Rank	Unique	Peptide
1	755.4208	754.4136	754.4337	-26.73	0	16	0.83	1	U	K.APGIIER.K
6	1026.5668	1025.5595	1025.5869	-26.73	0	41	0.0037	1	U	K.AVDSLVPIGR.G
8	1203.6340	1202.6267	1202.6506	-19.89	0	37	0.014	1	U	R.AAELTTLLESR.I
9	1210.6586	1209.6513	1209.6718	-16.87	0	61	3.7e-005	1	U	R.VVDALGVPIDGR.G
15	1816.8493	1815.8420	1815.8792	-20.47	0	111	3.1e-010	1	U	R.ITNFYTNFQVDEIGR.V

Proteins matching the same set of peptides:

Unigene89273_SeCKS transcribed RNA sequence Mass: 19896 Score: 264 Matches: 5(4) Sequences: 5(4)

gi|22742 pseudo-atpA [*Glycine max*]

2. Unigene55779_Se200S transcribed RNA sequence Mass: 20305 Score: 205 Matches: 3(2) Sequences: 3(2)

gi|162279935 atp1 gene product (mitochondrion) [*Beta vulgaris subsp. vulgaris*]

Query	Observed	Mr(expt)	Mr(calc)	ppm	Miss	Score	Expect	Rank	Unique	Peptide
5	892.4621	891.4548	891.4814	-29.78	0	19	1	1	U	K.LELAQYR.E
16	1967.0756	1966.0683	1966.1139	-23.18	1	78	4.9e-007	1	U	R.LTEVLKQPQYAPLPIEK.Q
25	2308.1348	2307.1275	2307.1495	-9.54	0	108	5.6e-010	1	U	R.EVAAFAQFGSDLDAATQALLNR.G

Database 2

Match to: Unigene53297_SALfmcTARAAPEI-3 Mass: 33461 Score: 289 Matches: 4(3) Sequences: 4(3)

gi|162279935 atp1 gene product (mitochondrion) [*Beta vulgaris subsp. vulgaris*]

Matched peptides shown in bold red.

QKQLNSKATSESETLYCVYVAVGQKRSTVAQLVQILSEANALEYSILVAATASDPAPLQFLAPYSGCAMGEYFRDNGMHALIIYD

DLSKQAVAYRQMSLLLRRPPGREAFPGDVFYLHSRLLERAAKRSDQTGAGSLTALPVIETQAGDVSAYIPTNVISITDGQICLET

ELFYRGIRPAINVGLSVSRVGSAAQLKAMKQVCGSLKLELAQYREVAAFAQFGSDLDAATQALLNRGARLTEVLKQPQYAPLPI

EKQILVIYAAVNGFCDRMPLDKISQYEKTILNSVKPELLQELKGGLTNEK

Query	Observed	Mr(expt)	Mr(calc)	ppm	Miss	Score	Expect	Rank	Unique	Peptide
5	892.4621	891.4548	891.4814	-29.78	0	19	0.48	1	U	K.LELAQYR.E
14	1537.7089	1536.7016	1536.7361	-22.46	0	84	8.4e-008	1	U	R.EAFPGDVFYLHSR.L
16	1967.0756	1966.0683	1966.1139	-23.18	1	78	2.5e-007	1	U	R.LTEVLKQPQYAPLPIEK.Q
25	2308.1348	2307.1275	2307.1495	-9.54	0	108	2.1e-010	1	U	R.EVAAFAQFGSDLDAATQALLNR.G

2. Unigene16541_SALfmcTARAAPEI-3 Mass: 20907 Score: 283 Matches: 7(4) Sequences: 6(4)

gi|22742 pseudo-atpA [*Glycine max*]

Query	Observed	Mr(expt)	Mr(calc)	ppm	Miss	Score	Expect	Rank	Unique	Peptide
1	755.4208	754.4136	754.4337	-26.73	0	16	0.51	1	U	K.APGIIER.K
2	766.3342	765.3269	765.3479	-27.49	0	(18)	0.34	1	U	-.MEFSPR.A
3	782.3300	781.3227	781.3429	-25.81	0	19	0.17	1	U	-.MEFSPR.A + Oxidation (M)
6	1026.5668	1025.5595	1025.5869	-26.73	0	41	0.0021	1	U	K.AVDSLVPIGR.G
8	1203.6340	1202.6267	1202.6506	-19.89	0	37	0.0065	1	U	R.AAELTTLLESR.I
9	1210.6586	1209.6513	1209.6718	-16.87	0	61	1.9e-005	1	U	R.VVDALGVPIDGR.G
15	1816.8493	1815.8420	1815.8792	-20.47	0	111	1.6e-010	1	U	R.ITNFYTNFQVDEIGR.V

Spot 2053

NCBInr protein database

Match to: gi|590564704 Mass: 19652 Score: 208 Matches: 3(2) Sequences: 3(2)

ATP synthase D chain, mitochondrial [*Theobroma cacao*]

Matched peptides shown in bold red.

1 MSGAGKKVVD VAFKASKNID WEGMAKLLVS DEARKEFATL RRAFDEVNST

51 LQTKFSQEPE PIDWEYYRKG IGSRLVDMYK EAYDSVEIPK FVDTVTPQYK

101 PKFDALLIEL KEAEEKSLKE SVRLEKEIAE VQELKKKIST MTADEYFEKH

151 PELKKKFDDE IRNDYWGY

Query	Observed	Mr(expt)	Mr(calc)	ppm	Miss	Score	Expect	Rank	Unique	Peptide

21 1079.5206 1078.5133 1078.4753 35.2 0 46 0.079 1 K.NIDWEGMAK.L + Oxidation (M)

35 1450.6988 1449.6915 1449.6333 40.1 0 61 0.0021 1 K.ISTMTADEYFEK.H + Oxidation (M)

47 1858.8925 1857.8852 1857.8210 34.6 0 100 2e-07 1 K.FSQEPEPIDWEYYR.K

2. gi|470143505 Mass: 20172 Score: 187 Matches: 2(2) Sequences: 2(2)

PREDICTED: ATP synthase subunit d, mitochondrial-like [*Fragaria vesca subsp. vesca*]

Query Observed Mr(expt) Mr(calc) ppm Miss Score Expect Rank Unique Peptide

47 1858.8925 1857.8852 1857.8210 34.6 0 100 2.1e-07 1 K.FSQEPEPIDWEYYR.K

54 2067.0690 2066.0617 2065.9779 40.6 1 87 4.1e-06 3 U K.IATMTADEYFEKHPELR.K + Oxidation (M)

3. gi|357122954 Mass: 19609 Score: 177 Matches: 3(2) Sequences: 3(2)

PREDICTED: ATP synthase subunit d, mitochondrial-like [*Brachypodium distachyon*]

Query Observed Mr(expt) Mr(calc) ppm Miss Score Expect Rank Unique Peptide

35 1450.6988 1449.6915 1449.6333 40.1 0 61 0.0021 1 K.ISTMTADEYFEK.H + Oxidation (M)

41 1592.7284 1591.7211 1591.7954 -46.66 1 27 4.6 1 R.RTFEDVNNQLQTK.F

54 2067.0690 2066.0617 2065.9779 40.6 1 89 2.6e-06 1 U K.ISTMTADEYFEKHPELR.K

4. gi|449449330 Mass: 19773 Score: 161 Matches: 2(2) Sequences: 2(2)

PREDICTED: ATP synthase subunit d, mitochondrial-like [*Cucumis sativus*]

Query Observed Mr(expt) Mr(calc) ppm Miss Score Expect Rank Unique Peptide

25 1134.6022 1133.5949 1133.5604 30.4 0 61 0.003 1 U K.EAYDGIEIPK.F

47 1858.8925 1857.8852 1857.8210 34.6 0 100 2.1e-07 1 K.FSQEPEPIDWEYYR.K

5. gi|351727204 Mass: 19944 Score: 150 Matches: 2(2) Sequences: 2(2)

uncharacterized protein LOC100500032 [*Glycine max*]

Query Observed Mr(expt) Mr(calc) ppm Miss Score Expect Rank Unique Peptide

35 1450.6988 1449.6915 1449.6333 40.1 0 61 0.0021 1 U K.LSTMTADEYFEK.H + Oxidation (M)

54 2067.0690 2066.0617 2065.9779 40.6 1 89 2.6e-06 1 U K.LSTMTADEYFEKHPELR.K

Proteins matching the same set of peptides:

gi|351734514 Mass: 19911 Score: 150 Matches: 2(2) Sequences: 2(2)

uncharacterized protein LOC100306184 [*Glycine max*]

7. gi|514797048 Mass: 24317 Score: 116 Matches: 2(2) Sequences: 2(2)

PREDICTED: ATP synthase subunit d, mitochondrial-like isoform X1 [*Setaria italica*]

Query Observed Mr(expt) Mr(calc) ppm Miss Score Expect Rank Unique Peptide

17 1050.5895 1049.5823 1049.5142 64.9 1 55 0.011 1 K.QKFDDEIR.N

35 1450.6988 1449.6915 1449.6333 40.1 0 61 0.0021 1 K.ISTMTADEYFEK.H + Oxidation (M)

Proteins matching the same set of peptides:

gi|514797052 Mass: 19723 Score: 116 Matches: 2(2) Sequences: 2(2)

PREDICTED: ATP synthase subunit d, mitochondrial-like isoform X2 [*Setaria italica*]

gi|573954514 Mass: 19540 Score: 116 Matches: 2(2) Sequences: 2(2)

PREDICTED: ATP synthase subunit d, mitochondrial-like [*Oryza brachyantha*]

Database 1

Match to: Unigene505_SeCKS transcribed RNA sequence Mass: 22469 Score: 618 Matches: 11(9) Sequences: 10(9)

gi| 590564704 ATP synthase D chain [*Theobroma cacao*]

Matched peptides shown in bold red.

PNIKICSSSQDHRAREKRETPKMSGVGKKVADVTFKASKNIDWEGMAKLLVSDEARKEFFTLRRTFDEVNTQLQTKFSQEPEPI
DWEYYRKGIGNKLVDMYKEAYDGIEIPKYVDKVTPEYKPKIEALLVELKDAEQQSLKESERLEKEIADVQELKQKLSTMTADE
YFEKHPELRKKFDDEIRNDYWGY

Query	Observed	Mr(expt)	Mr(calc)	ppm	Miss	Score	Expect	Rank	Unique	Peptide
16	1044.5887	1043.5814	1043.5499	30.2	0	36	0.015	1	U	K.EIADVQELK.Q
19	1063.5276	1062.5203	1062.4804	37.6	0	(19)	0.89	1	U	K.NIDWEGMAK.L
21	1079.5206	1078.5133	1078.4753	35.2	0	46	0.0015	1	U	K.NIDWEGMAK.L + Oxidation (M)
25	1134.6022	1133.5949	1133.5604	30.4	0	61	5.9e-005	1	U	K.EAYDGIEIPK.Y
33	1423.7563	1422.7491	1422.6991	35.1	0	80	4.5e-007	1	U	R.TFDEVNTQLQTK.F
35	1450.6988	1449.6915	1449.6333	40.1	0	61	3.9e-005	1	U	K.LSTMTADEYFEK.H + Oxidation (M)
37	1466.8422	1465.8349	1465.7817	36.3	1	65	1e-005	1	U	K.YVDKVTPEYKPK.I
40	1579.8714	1578.8641	1578.8002	40.5	1	76	9.4e-007	1	U	R.RTFDEVNTQLQTK.F
41	1592.7284	1591.7211	1591.6579	39.7	1	4	13	1	U	K.FDDEIRNDYWGY.-
47	1858.8925	1857.8852	1857.8210	34.6	0	100	3.8e-009	1	U	K.FSQEPEPIDWEYYR.K
54	2067.0690	2066.0617	2065.9779	40.6	1	89	5.9e-008	1	U	K.LSTMTADEYFEKHPELR.K

Proteins matching the same set of peptides:

Unigene506_SeCKS transcribed RNA sequence Mass: 22469 Score: 618 Matches: 11(9) Sequences: 10(9)

gi| 661887145 unnamed protein product [*Coffea canephora*]

Unigene1307_Se200S transcribed RNA sequence Mass: 22005 Score: 618 Matches: 11(9) Sequences: 10(9)

gi| 661887145 unnamed protein product [*Coffea canephora*]

Unigene5065_SeCKS transcribed RNA sequence Mass: 22469 Score: 618 Matches: 11(9) Sequences: 10(9)

gi| 661887145 unnamed protein product [*Coffea canephora*]

Unigene46701_Se200S transcribed RNA sequence Mass: 22005 Score: 618 Matches: 11(9) Sequences: 10(9)

gi| 661887145 unnamed protein product [*Coffea canephora*]

Unigene46702_Se200S transcribed RNA sequence Mass: 22005 Score: 618 Matches: 11(9) Sequences: 10(9)

gi| 661887145 unnamed protein product [*Coffea canephora*]

Database 2

Match to: Unigene26231_SALfmcTARAAPEI-3 Mass: 7076 Score: 135 Matches: 4(3) Sequences: 4(3)

gi|255570779 ATP synthase D chain [*Ricinus communis*]

Matched peptides shown in bold red.

KDAEQQSLKESERLEKEIADVQELKQKLSTMTADEYFEKHPELRKKFDDEIRNDYWGY

Query	Observed	Mr(expt)	Mr(calc)	ppm	Miss	Score	Expect	Rank	Unique	Peptide
16	1044.5887	1043.5814	1043.5499	30.2	0	36	0.0081	1	U	K.EIADVQELK.Q
35	1450.6988	1449.6915	1449.6333	40.1	0	61	1.9e-005	1	U	K.LSTMTADEYFEK.H + Oxidation (M)
41	1592.7284	1591.7211	1591.6579	39.7	1	4	9.4	1	U	K.FDDEIRNDYWGY.-
54	2067.0690	2066.0617	2065.9779	40.6	1	89	2.8e-008	1	U	K.LSTMTADEYFEKHPELR.K

Spot 2171

NCBInr protein database

Match to: gi|460378721 Mass: 100274 Score: 337 Matches: 4(4) Sequences: 3(3)

PREDICTED: metal-nicotianamine transporter YSL2-like [*Solanum lycopersicum*]

Matched peptides shown in bold red.

MSRVGVELMEIEREVTEEMRDGDDEVKRIPPWTKQITVRGIVASVLIGVIYSVIVTKLNLTTGLVPNLNV
SAALLAYVILQSWTKVLKKANFTCTPFTKQENTIIQTCAVACYSIAVGGGFGSYLLGLNKKTYQQAGVDT
SGNTPGSYKEPKLDWMIGFLFVVSFVGLLALVPLRKIMIIDYKLPYPSGTATAVLINGFHTPKGDKLAKK
QVKGFMKVFTFSFFWSFFQWFYSGGDHCGFANFPTFGLKAWKQSFFFDFDMTYVGAGMICSHLVNFSLLL
GAVLSWGIMWPLITDRDGYWFPSSLPQSSMKSLMGYKVFISIALLLGDGLYNFVRTLYFTFRNIYATLKT
KRGKTLSPSVEILLLTLHFLAAPSEAKNLPLEELQRNEIFIRESIPFWLACIGYLIFSLISIIVIPIMFP
ALKWYYYVLVAYVFAPALSFCNAYGAGLTDLNMAYNYGKVALFVLAALSGKENGVVAGLIGCGLIKSMVSI
SSDLMHDFKTSHLTLTSPRSMLLSQAIGTAIGCVVAPLTFFLFYKSFDVGGPNGEYKAPYALIYRNMAIL
GVEGFSALPRHCLQLCYGFFAFAVLANLVRDMAPERVGKLVPLPMAIAVPFLVGASFAIDMAAGSLIVYV
WHKLNSKKADLMVPAVASGFICGDGLWILPSALLALLKVRPPICMAFTDVVVRVRVRLFKETHEADGGRK
RRSIGDGRRHGVLTEDQWAIRYLNFYINIDPVLPPPHAKRVLGAQSETETESKELSLVSQEKMASDRKIH
AFEEVAKHNKTKDCWLIISGKVYDVTPFMDDHPGGDEVLLSATGKDATNDFEDVGHSDSAREMMDKYYIG
EIDMSTVPLKRAYIPSEQTAYNPDKTPEFIIKILQFLVPILILGLAFTVRHYTKEQ

Query	Observed	Mr(expt)	Mr(calc)	ppm	Miss	Score	Expect	Rank	Unique	Peptide
5	1091.5810	1090.5738	1090.5481	23.5	0	56	0.01	1		K.DCWLIISGK.V
27	1735.7561	1734.7488	1734.7081	23.5	0	138	2.9e-11	1		K.DATNDFEDVGHSDSAR.E
40	2563.2602	2562.2530	2562.1949	22.7	0	(101)	1.2e-07	1	U	K.VYDVTPFMDDHPGGDEVLLSATGK.D
41	2579.2576	2578.2504	2578.1898	23.5	0	143	7.1e-12	1	U	K.VYDVTPFMDDHPGGDEVLLSATGK.D + Oxidation (M)

Proteins matching the same set of peptides:

gi|565367967 Mass: 96902 Score: 337 Matches: 4(4) Sequences: 3(3)

PREDICTED: metal-nicotianamine transporter YSL2-like [*Solanum tuberosum*]

gi|590674420 Mass: 15179 Score: 337 Matches: 4(4) Sequences: 3(3)

Cytochrome B5, n2,ATCB5-E,CB5-E isoform 1 [*Theobroma cacao*]

gi|590674427 Mass: 18257 Score: 337 Matches: 4(4) Sequences: 3(3)

Cytochrome B5, n2,ATCB5-E,CB5-E isoform 3, partial [*Theobroma cacao*]

2. gi|357464067 Mass: 93737 Score: 293 Matches: 4(4) Sequences: 3(3)

YSL transporter [*Medicago truncatula*]

Query	Observed	Mr(expt)	Mr(calc)	ppm	Miss	Score	Expect	Rank	Unique	Peptide
5	1091.5810	1090.5738	1090.5481	23.5	0	56	0.01	1		K.DCWLILSGK.V
27	1735.7561	1734.7488	1734.7081	23.5	0	138	2.9e-11	1		K.DATNDFEDVGHSDSAR.E
40	2563.2602	2562.2530	2562.1948	22.7	0	(62)	0.00099	2	U	K.VYDVSPFMEDHPGGDEVLLSATGK.D
41	2579.2576	2578.2504	2578.1898	23.5	0	99	1.7e-07	2	U	K.VYDVSPFMEDHPGGDEVLLSATGK.D + Oxidation (M)

Proteins matching the same set of peptides:

gi|388502872 Mass: 15217 Score: 293 Matches: 4(4) Sequences: 3(3)

unknown [*Medicago truncatula*]

gi|388504050 Mass: 17270 Score: 293 Matches: 4(4) Sequences: 3(3)

unknown [*Medicago truncatula*]

3. gi|593268612 Mass: 15191 Score: 286 Matches: 3(3) Sequences: 3(3)

hypothetical protein PHAVU_009G048900g [*Phaseolus vulgaris*]

Query	Observed	Mr(expt)	Mr(calc)	ppm	Miss	Score	Expect	Rank	Unique	Peptide
19	1568.8488	1567.8415	1567.8134	18.0	0	60	0.0027	1		K.YYIGEIDASTVPLK.R
26	1724.9555	1723.9482	1723.9145	19.6	1	88	3.7e-06	1		K.YYIGEIDASTVPLKR.T
27	1735.7561	1734.7488	1734.7081	23.5	0	138	2.9e-11	1		K.DATNDFEDVGHSDSAR.D

4. gi|330318688 Mass: 15251 Score: 226 Matches: 4(2) Sequences: 3(2)

cytochrome b5 (chloroplast) [*Camellia sinensis*]

Query	Observed	Mr(expt)	Mr(calc)	ppm	Miss	Score	Expect	Rank	Unique	Peptide
5	1091.5810	1090.5738	1090.5481	23.5	0	56	0.01	1		K.DCWLILSGK.V
27	1735.7561	1734.7488	1734.7081	23.5	0	138	2.9e-11	1		K.DATNDFEDVGHSDSAR.E
41	2579.2576	2578.2504	2578.1898	23.5	0	32	0.99	4		K.VYDVTPFMDDHPGGDEVLLSSTGK.D
42	2595.2598	2594.2525	2594.1847	26.2	0	(12)	1e+02	2		K.VYDVTPFMDDHPGGDEVLLSSTGK.D + Oxidation (M)

5. gi|470146999 Mass: 96365 Score: 203 Matches: 3(3) Sequences: 3(3)

PREDICTED: LOW QUALITY PROTEIN: metal-nicotianamine transporter YSL3-like [*Fragaria vesca subsp. vesca*]

Query	Observed	Mr(expt)	Mr(calc)	ppm	Miss	Score	Expect	Rank	Unique	Peptide
5	1091.5810	1090.5738	1090.5481	23.5	0	56	0.01	1		K.DCWLIISGK.V
19	1568.8488	1567.8415	1567.8134	18.0	0	60	0.0027	1		K.YYIGEIDASTVPLK.R
26	1724.9555	1723.9482	1723.9145	19.6	1	88	3.7e-06	1		K.YYIGEIDASTVPLKR.T

6. gi|596010715 Mass: 15197 Score: 197 Matches: 2(2) Sequences: 2(2)

hypothetical protein PRUPE_ppa013232mg [*Prunus persica*]

Query	Observed	Mr(expt)	Mr(calc)	ppm	Miss	Score	Expect	Rank	Unique	Peptide
27	1735.7561	1734.7488	1734.7081	23.5	0	138	2.9e-11	1		K.DATNDFEDVGHSDSAR.D
40	2563.2602	2562.2530	2562.1949	22.7	0	59	0.0019	3	U	K.VFDVTPFMDDHPGGDEVLLSATGK.D + Oxidation (M)

Database 1

Match to: Unigene1174_SeCKS transcribed RNA sequence Mass: 15548 Score: 644 Matches: 9(8) Sequences: 8(7)

gi|351726704 uncharacterized protein LOC100305929 [*Glycine max*]

gi|587850936 Cytochrome b5 isoform 1 [*Morus notabilis*]

Matched peptides shown in **bold red**.

KMSSDR**KVHSFDEVSQHNSTKDCWLIISGKVYDVTPFMDDHPGGDEVLLSATGKDATNDFEDVGHSDSAR**EMMDK**YYIGEIDASTVPLK**R**SYVPPQQVHYNHDK**TPEFIIKILQFLVPILILGLAFVVRHYTKKED

Query	Observed	Mr(expt)	Mr(calc)	ppm	Miss	Score	Expect	Rank	Unique	Peptide
5	1091.5810	1090.5738	1090.5481	23.5	0	56	0.00021	1	U	K.DCWLIISGK.V
19	1568.8488	1567.8415	1567.8134	18.0	0	60	4.8e-005	1	U	K.YYIGEIDASTVPLK.R
20	1614.7839	1613.7766	1613.7434	20.6	0	102	3e-009	1	U	K.VHSFDEVSQHNSTK.D
23	1711.8495	1710.8423	1710.8114	18.0	0	36	0.013	1	U	R.SYVPPQQVHYNHDK.T
26	1724.9555	1723.9482	1723.9145	19.6	1	88	5.4e-008	1	U	K.YYIGEIDASTVPLKR.S
27	1735.7561	1734.7488	1734.7081	23.5	0	138	3.4e-013	1	U	K.DATNDFEDVGHSDSAR.E
28	1742.8871	1741.8798	1741.8384	23.8	1	21	0.36	1	U	R.KVHSFDEVSQHNSTK.D

40 2563.2602 2562.2530 2562.1949 22.7 0 (101) 2.7e-009 1 U

K.VYDVTPFMDDHPGGDEVLLSATGK.D

41 2579.2576 2578.2504 2578.1898 23.5 0 143 1.5e-013 1 U

K.VYDVTPFMDDHPGGDEVLLSATGK.D + Oxidation (M)

Proteins matching the same set of peptides:

Unigene1175_SeCKS transcribed RNA sequence Mass: 15534 Score: 644 Matches: 9(8) Sequences: 8(7)

gi|351726704 uncharacterized protein LOC100305929 [*Glycine max*]

Unigene2848_SeCKS transcribed RNA sequence Mass: 15534 Score: 644 Matches: 9(8) Sequences: 8(7)

gi|351726704 uncharacterized protein LOC100305929 [*Glycine max*]

Unigene15085_SeCKS transcribed RNA sequence Mass: 15548 Score: 644 Matches: 9(8) Sequences: 8(7)

gi|351726704 uncharacterized protein LOC100305929 [*Glycine max*]

Unigene15086_SeCKS transcribed RNA sequence Mass: 15534 Score: 644 Matches: 9(8) Sequences: 8(7)

gi|351726704 uncharacterized protein LOC100305929 [*Glycine max*]

Unigene18116_SeCKS transcribed RNA sequence Mass: 15534 Score: 644 Matches: 9(8) Sequences: 8(7)

gi|351726704 uncharacterized protein LOC100305929 [*Glycine max*]

Unigene46385_Se200S transcribed RNA sequence Mass: 13791 Score: 644 Matches: 9(8) Sequences: 8(7)

gi|351726704 uncharacterized protein LOC100305929 [*Glycine max*]

Spot 1008

NCBInr protein database

Match to: gi|2695711 Mass: 15364 Score: 58 Matches: 1(1) Sequences: 1(1)

cytochome b5 [*Olea europaea*]

Matched peptides shown in **bold red**.

1 MASDPKIYVY EEVEKHDKTK DCWLVINGKV YDVTPFMDDH PGGDEVLLSA

51 TGK**DATNDFE DVGHSDSAR**E MMDKYYIGEI DVSTVPTKRT YTPPQQAQYN

101 PDKTPEFLIK ILQFLVPLLI LGLAFVVRHY TKEK

Query	Observed	Mr(expt)	Mr(calc)	ppm	Miss	Score	Expect	Rank	Unique	Peptide
14	1735.7912	1734.7839	1734.7081	43.7	0	58	0.0033	1	U	K.DATNDFEDVGHSDSAR.E

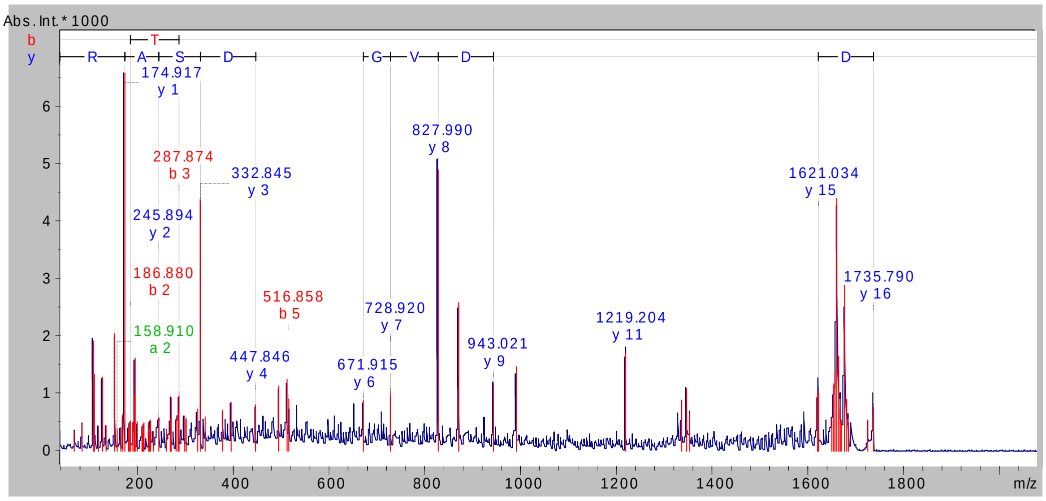

Proteins matching the same set of peptides:

gi|50844673 Mass: 15162 Score: 58 Matches: 1(1) Sequences: 1(1)

cytochrome b5 isoform Cb5-A [*Vernicia fordii*]

gi|219547603 Mass: 15243 Score: 58 Matches: 1(1) Sequences: 1(1)

cytochrome b5 [*Malus domestica*]

gi|255554361 Mass: 15218 Score: 58 Matches: 1(1) Sequences: 1(1)

cytochrome B5 isoform 1, putative [*Ricinus communis*]

gi|284433804 Mass: 15078 Score: 58 Matches: 1(1) Sequences: 1(1)

cytochrome b5 [*Jatropha curcas*]

gi|330318688 Mass: 15251 Score: 58 Matches: 1(1) Sequences: 1(1)

cytochrome b5 (chloroplast) [*Camellia sinensis*]

gi|351723153 Mass: 15323 Score: 58 Matches: 1(1) Sequences: 1(1)

uncharacterized protein LOC100499823 [*Glycine max*]

gi|351726704 Mass: 15333 Score: 58 Matches: 1(1) Sequences: 1(1)

uncharacterized protein LOC100305929 [*Glycine max*]

gi|357464067 Mass: 93737 Score: 58 Matches: 1(1) Sequences: 1(1)

YSL transporter [*Medicago truncatula*]

gi|388496008 Mass: 15337 Score: 58 Matches: 1(1) Sequences: 1(1)

unknown [*Lotus japonicus*]

gi|388502872 Mass: 15217 Score: 58 Matches: 1(1) Sequences: 1(1)

unknown [*Medicago truncatula*]

gi|388504050 Mass: 17270 Score: 58 Matches: 1(1) Sequences: 1(1)

unknown [*Medicago truncatula*]

gi|388510986 Mass: 15385 Score: 58 Matches: 1(1) Sequences: 1(1)

unknown [*Lotus japonicus*]

gi|449456423 Mass: 15164 Score: 58 Matches: 1(1) Sequences: 1(1)

PREDICTED: cytochrome b5 isoform A-like [*Cucumis sativus*]

gi|460378721 Mass: 100274 Score: 58 Matches: 1(1) Sequences: 1(1)

PREDICTED: metal-nicotianamine transporter YSL2-like [*Solanum lycopersicum*]

gi|565367967 Mass: 96902 Score: 58 Matches: 1(1) Sequences: 1(1)

PREDICTED: metal-nicotianamine transporter YSL2-like [*Solanum tuberosum*]

gi|565403824 Mass: 15159 Score: 58 Matches: 1(1) Sequences: 1(1)

PREDICTED: cytochrome b5 isoform A-like [*Solanum tuberosum*]

gi|567897406 Mass: 15232 Score: 58 Matches: 1(1) Sequences: 1(1)

hypothetical protein CICLE_v10022811mg [*Citrus clementina*]

gi|567897412 Mass: 14569 Score: 58 Matches: 1(1) Sequences: 1(1)

hypothetical protein CICLE_v10022811mg [*Citrus clementina*]

gi|568880500 Mass: 15187 Score: 58 Matches: 1(1) Sequences: 1(1)

PREDICTED: cytochrome b5 isoform A-like isoform X1 [*Citrus sinensis*]

gi|571448290 Mass: 15324 Score: 58 Matches: 1(1) Sequences: 1(1)

PREDICTED: uncharacterized protein LOC100499823 isoform X1 [*Glycine max*]

gi|587850936 Mass: 15253 Score: 58 Matches: 1(1) Sequences: 1(1)

Cytochrome b5 isoform 1 [*Morus notabilis*]

gi|590674420 Mass: 15179 Score: 58 Matches: 1(1) Sequences: 1(1)

Cytochrome B5, n2,ATCB5-E,CB5-E isoform 1 [*Theobroma cacao*]

gi|590674427 Mass: 18257 Score: 58 Matches: 1(1) Sequences: 1(1)

Cytochrome B5, n2,ATCB5-E,CB5-E isoform 3, partial [*Theobroma cacao*]

gi|593268612 Mass: 15191 Score: 58 Matches: 1(1) Sequences: 1(1)

hypothetical protein PHAVU_009G048900g [*Phaseolus vulgaris*]

gi|596010715 Mass: 15197 Score: 58 Matches: 1(1) Sequences: 1(1)

hypothetical protein PRUPE_ppa013232mg [*Prunus persica*]

Database 1

Match to: Unigene1174_SeCKS transcribed RNA sequence Mass: 15548 Score: 58 Matches: 1(1) Sequences: 1(1)

gi| 351726704 uncharacterized protein LOC100305929 [*Glycine max*]

Matched peptides shown in bold red.

KMSSDRKVHSFDEVSQHNSTKDCWLIISGKVYDVTPFMDDHPGGDEVLLSATGK**DATNDFEDVGHSDSAR**EMMDKYYIGEID

ASTVPLKRSYVPPQQVHYNHDKTPEFIIKILQFLVPILILGLAFVVRHYTKKED

Query	Observed	Mr(expt)	Mr(calc)	ppm	Miss	Score	Expect	Rank	Unique	Peptide
14	1735.7912	1734.7839	1734.7081	43.7	0	58	5.3e-005	1	U	K.DATNDFEDVGHSDSAR.E

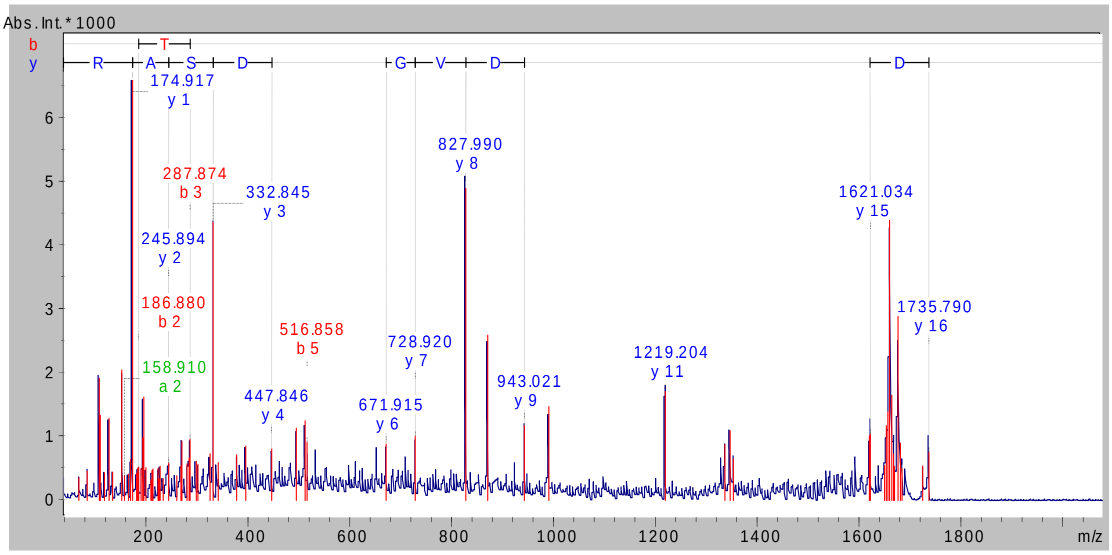

Proteins matching the same set of peptides:

Unigene1175_SeCKS transcribed RNA sequence Mass: 15534 Score: 644 Matches: 9(8) Sequences: 8(7)

gi|351726704 uncharacterized protein LOC100305929 [*Glycine max*]

Unigene2848_SeCKS transcribed RNA sequence Mass: 15534 Score: 644 Matches: 9(8) Sequences: 8(7)

gi|351726704 uncharacterized protein LOC100305929 [*Glycine max*]

Unigene15085_SeCKS transcribed RNA sequence Mass: 15548 Score: 644 Matches: 9(8) Sequences: 8(7)

gi|351726704 uncharacterized protein LOC100305929 [*Glycine max*]

Unigene15086_SeCKS transcribed RNA sequence Mass: 15534 Score: 644 Matches: 9(8) Sequences: 8(7)

gi|351726704 uncharacterized protein LOC100305929 [*Glycine max*]

Unigene18116_SeCKS transcribed RNA sequence Mass: 15534 Score: 644 Matches: 9(8) Sequences: 8(7)

gi|351726704 uncharacterized protein LOC100305929 [*Glycine max*]

Unigene46385_Se200S transcribed RNA sequence Mass: 13791 Score: 644 Matches: 9(8) Sequences: 8(7)

gi|351726704 uncharacterized protein LOC100305929 [*Glycine max*]

Spot 439

NCBInr protein database

Match to: gi|502137547 Mass: 82237 Score: 104 Matches: 4(1) Sequences: 3(1)

PREDICTED: NADH dehydrogenase [ubiquinone] iron-sulfur protein 1, mitochondrial-like [*Cicer arietinum*]

Matched peptides shown in **bold red.**

1 MGLGLLASKA IRPTSRLLSF QNPNNLLLRT IVSKPELRNP EPAAQPQPEQ

51 PLVDLSPRAP VGGARVHFPN PDDAIEVFVD GYPVKIPKGM SVLQACEIAG

101 VDIPR**FCYHS R**LSIAGNCRM CLVEVEKSPK PVASCAMPAL LGMKIKTDTP

151 VAKKAREGVM EFLLMNHPLD CPICDQGGEC DLQDQSMAFG SDRGRFTEMK

201 RSVVDKNLGP LVKTVMTRCI QCTRCVR**FAS EVAGVQDLGM LGR**GSGEEIG

251 TYVEKLMTSE LSGNVIDICP VGALTSKPFA FKARNWELK**G TESIDVTDAV**

301 **GSNIR**IDSRG PEVMRIVPRL NEDINEEWIS DKTRFCYDGL KRQRLNDPMI

351 RGPDGRFKAV NWREALALVA EVAHQVKPEE IVGISGKLSD AESMIALKDF

401 LNRMGSNNVW GEGIGVNTNA DFRSGYIMNT SIAGLEKADA FLLVGTQPRV

451 EAAMVNARIR KSVGSNHASV GYIGPATDFN YDYQHLGIGP QTLLEIAEGR

501 HPFSKTISKA KYPVIIVGAG IFERKDQDAI FAAVETIAKQ GNVVRSDWNG

551 LNVLLLHAAQ AAALDLGLVP QSEKNLESAK FVYLMGADDT NIDKIPEDAF

601 VVYQGHHGDK SVYRANVILP AAAFSEKEGI YENTEGCTQQ TWPAVPTVGD

651 SRDDWKIIRA LSEVAGVRLP YDTVGGVRAR LRTVAPNLVH IDEREPAALP

701 SSLRPSFTQK VDPTPFGIAV ENFYMTDAIT RASKIMAQCS SVLLKK

Query	Observed	Mr(expt)	Mr(calc)	ppm	Miss	Score	Expect	Rank	Unique	Peptide
2	869.3564	868.3491	868.3650	-18.30	0	22	7.7	1		R.FCYHSR.L
16	1633.7709	1632.7636	1632.7955	-19.53	0	47	0.052	1	U	K.GTESIDVTDAVGSNIR.I
17	1649.7930	1648.7858	1648.8243	-23.37	0	(31)	2.1	1	U	R.FASEVAGVQDLGMLGR.G
18	1665.7773	1664.7700	1664.8192	-29.54	0	35	0.7	1	U	R.FASEVAGVQDLGMLGR.G

+

Oxidation (M)

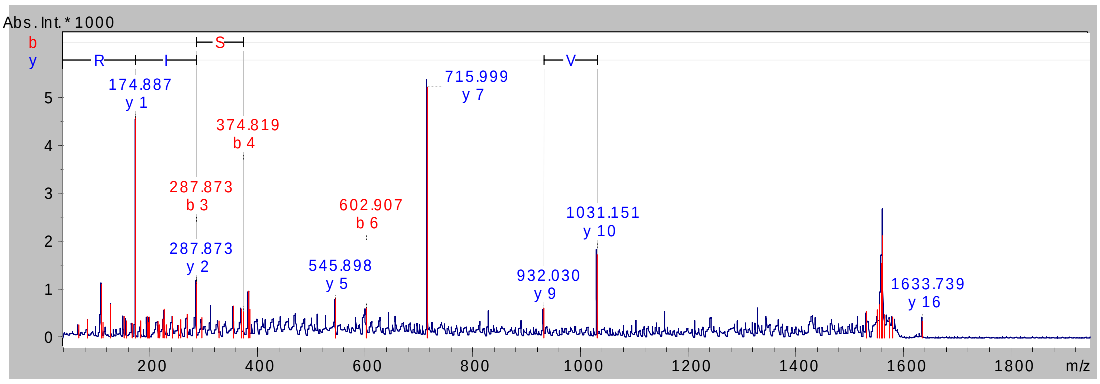

Proteins matching the same set of peptides:

gi|586728846 Mass: 85125 Score: 104 Matches: 4(1) Sequences: 3(1)

hypothetical protein AMTR_s00176p00030330 [*Amborella trichopoda*]

Database 1

Match to: Unigene 90143_Se200S transcribed RNA sequence Mass: 42404 Score: 139 Matches: 6(4) Sequences: 5(3)

gi|568215262 NADH dehydrogenase [ubiquinone] iron-sulfur protein 1, mitochondrial precursor [*Solanum tuberosum*]

Matched peptides shown in **bold red.**

RGRFTEMKRSVVDKNLGPLVKTVMTRCIQCTRCVR**FASEVAGVQDLGMLGRGSGEEIGTYVER**LMTSELSGNVIDICPVGALT

SKPFAFKARNWELK**GTESIDVTDAVGSNIR**IDSRGPEVMRVIPRLNEDVNEEWISDKTR**FCYDGLKR**QRLNDPMIRGSDGRFKA

VSWRDALEIIAEVMHKVNPKEIAGVAGKLSDAESMMALKDFLNKMGSNNVWCEGNGGQPQADLRSGYLLNTGIADLENAD

VFLLIGTQPRVEAAMVNARIQKAVRGHHGKVGYIGPAAEFNYDVVHLGTGPQTLQEIAEGNHSFFSALKNAKNPAIVVGAGLF

ERDDKDAILCLVETIAKSASVIRPDWNGLNVLLLNAAQAAALDLGLVPESDKSIES

Query	Observed	Mr(expt)	Mr(calc)	ppm	Miss	Score	Expect	Rank	Unique	Peptide
4	1058.4726	1057.4653	1057.5015	-34.24	1	7	4.6	2	U	R.FCYDGLKR.Q
10	1296.5794	1295.5721	1295.5994	-21.00	0	31	0.029	1	U	R.GSGEEIGTYVER.L
13	1342.7128	1341.7055	1341.7405	-26.08	0	19	0.63	1	U	K.NPAIVVGAGLFER.D
16	1633.7709	1632.7636	1632.7955	-19.53	0	47	0.00085	1	U	K.GTESIDVTDAVGSNIR.I
17	1649.7930	1648.7858	1648.8243	-23.37	0	(31)	0.041	1	U	R.FASEVAGVQDLGMLGR.G
18	1665.7773	1664.7700	1664.8192	-29.54	0	35	0.014	1	U	R.FASEVAGVQDLGMLGR.G

+ Oxidation (M)

Database 2

Match to: Unigene55081_SALfmcTARAAPEI-3 Mass: 44552 Score: 142 Matches: 6(4) Sequences: 5(3)

gi| 255582280 NADH-ubiquinone oxidoreductase, putative [*Ricinus communis*]

Matched peptides shown in bold red.

AEAVPETPPPPPRPPVNDARVHFPNPEDAIEVFVDGYSVKIPKGFTVLQACEVAGIDIPRFCYHSRLSIAGNCRMCLVEVEKSPKP

VASCAMPALPGMKIKTDTPLAKKAREGVMEFLLMNHPLDCPICDQGGECDLQDQSMAFGSDRGRFTEMKRSVVDKNLGPLV

KTVMTRCIQCTRCVRFASEVAGVQDLGMLGRGSGEEIGTYVERLMTSELSGNVIDICPVGALTSKPFAFKARNWELKGTESIDV

TDAVGSNIRIDSRGPEVMRVIPRLNEDVNEEWISDKTRFCYDGLKRQRLNDPMIRGSDGRFKAVSWRDALEIIAEVMHKVNPK

EIAGVAGKLSDAESMMALKDFLNKMGSNNVWCEGNGGQPQADLRSGYLLNTGIADLENADVFLLI

Query	Observed	Mr(expt)	Mr(calc)	ppm	Miss	Score	Expect	Rank	Unique	Peptide
2	869.3564	868.3491	868.3650	-18.30	0	22	0.079	1	U	R.FCYHSR.L
4	1058.4726	1057.4653	1057.5015	-34.24	1	7	3.8	2	U	R.FCYDGLKR.Q
10	1296.5794	1295.5721	1295.5994	-21.00	0	31	0.018	1	U	R.GSGEEIGTYVER.L
16	1633.7709	1632.7636	1632.7955	-19.53	0	47	0.00041	1	U	K.GTESIDVTDAVGSNIR.I
17	1649.7930	1648.7858	1648.8243	-23.37	0	(31)	0.018	1	U	R.FASEVAGVQDLGMLGR.G
18	1665.7773	1664.7700	1664.8192	-29.54	0	35	0.0063	1	U	R.FASEVAGVQDLGMLGR.G

+ Oxidation (M)

Spot 1831

NCBInr protein database

Match to: gi|567861210 Mass: 26545 Score: 403 Matches: 6(4) Sequences: 6(4)

hypothetical protein CICLE_v10029226mg [*Citrus clementina*]

Matched peptides shown in bold red.

1 MASILARKSL SALRARHLAV SGQALQGSQH YGLRFNAHPY SSYFPSKKDD

51 EEKEQLLKEI SKDWSSVFER SINMLFLTEM VRGLGLTLKY FFDKKVTINY

101 PFEKGPLSPR FRGEHALRRY PTGEERCIAC KLCEAVCPAQ AITIEAEERE

151 DGSRRTTRYD IDMTKCIYCG FCQEACPVDA IVEGPNFEYS TETHEELLYD

201 KEKLLENGDR WETEIAENLR SESLYR

Query	Observed	Mr(expt)	Mr(calc)	ppm	Miss	Score	Expect	Rank	Unique	Peptide
12	1007.5069	1006.4997	1006.4832	16.4	1	43	0.21	1	U	R.RYPTGEER.C
13	1025.4770	1024.4698	1024.4614	8.14	0	37	0.46	1	U	K.DWSSVFER.S
17	1110.5995	1109.5922	1109.5757	14.9	0	57	0.0053	1	U	K.VTINYPFEK.G
21	1260.6286	1259.6213	1259.6146	5.34	0	67	0.0007	1	U	R.WETEIAENLR.S
29	1482.7172	1481.7099	1481.7150	-3.46	1	106	6.9e-08	1	U	K.EISKDWSSVFER.S
40	2059.9752	2058.9680	2058.9714	-1.67	0	93	9.6e-07	1	U	K.LCEAVCPAQAITIEAEER.E

Database 1

Match to: Unigene15189_SeCKS transcribed RNA sequence Mass: 27914 Score: 435 Matches: 8(8) Sequences: 7(7)

gi| 568384762 NADH dehydrogenase [ubiquinone] iron-sulfur protein 8, mitochondrial precursor [*Solanum tuberosum*]

Matched peptides shown in bold red.

CVEPPLSVSPSHIRTILLATTPQEQPQQSESGRLTLFSPASMAAILARRSLLALRSRQLAASQGFSHLETRSFATKHSFSNDKDDME

REKLAREISKDWSSVFERSINTLFLTEMARGLSMTLKYFFEDKVTINYPFEKGPLSPRFRGEHALRRYPTGEERCIACKLCEAVC

PAQAITIEAEEREDGSRRTTRYDIDMTKCIYCGFCQEACPVDAIVEGPNFEFATETHEELLYDKEKLLE

Query	Observed	Mr(expt)	Mr(calc)	ppm	Miss	Score	Expect	Rank	Unique	Peptide
12	1007.5069	1006.4997	1006.4832	16.4	1	43	0.0042	1		R.RYPTGEER.C
13	1025.4770	1024.4698	1024.4614	8.14	0	37	0.0081	1		K.DWSSVFER.S
17	1110.5995	1109.5922	1109.5757	14.9	0	57	9.5e-005	1		K.VTINYPFEK.G
24	1395.7335	1394.7262	1394.7228	2.49	0	(49)	0.00079	1		R.SINTLFLTEMAR.G
25	1411.7274	1410.7201	1410.7177	1.73	0	51	0.0004	1		R.SINTLFLTEMAR.G + Oxidation (M)
29	1482.7172	1481.7099	1481.7150	-3.46	1	106	1.3e-009	1		R.EISKDWSSVFER.S
34	1939.9164	1938.9092	1938.9404	-16.08	1	48	0.00067	1		K.YFFEDKVTINYPFEK.G
40	2059.9752	2058.9680	2058.9714	-1.67	0	93	1.7e-008	1		K.LCEAVCPAQAITIEAEER.E

2. Unigene33352_Se200S transcribed RNA sequence Mass: 16766 Score: 307 Matches: 5(5) Sequences: 5(5)

gi|255536839 NADH-ubiquinone oxidoreductase 1, chain, putative [*Ricinus communis*]

Query	Observed	Mr(expt)	Mr(calc)	ppm	Miss	Score	Expect	Rank	Unique	Peptide
12	1007.5069	1006.4997	1006.4832	16.4	1	43	0.0042	1		R.RYPTGEER.C
17	1110.5995	1109.5922	1109.5757	14.9	0	57	9.5e-005	1		K.VTINYPFEK.G
21	1260.6286	1259.6213	1259.6146	5.34	0	67	1.2e-005	1	U	R.WETEIAENLR.S
34	1939.9164	1938.9092	1938.9404	-16.08	1	48	0.00067	1		K.YFFEDKVTINYPFEK.G
40	2059.9752	2058.9680	2058.9714	-1.67	0	93	1.7e-008	1		K.LCEAVCPAQAITIEAEER.E

3. Unigene6812_Se200S transcribed RNA sequence Mass: 13553 Score: 290 Matches: 5(5) Sequences: 4(4)

gi| 388493828 unknown [*Lotus japonicus*]

Query	Observed	Mr(expt)	Mr(calc)	ppm	Miss	Score	Expect	Rank	Unique	Peptide
13	1025.4770	1024.4698	1024.4614	8.14	0	37	0.0081	1		K.DWSSVFER.S
24	1395.7335	1394.7262	1394.7228	2.49	0	(49)	0.00079	1		R.SINTLFLTEMAR.G
25	1411.7274	1410.7201	1410.7177	1.73	0	51	0.0004	1		R.SINTLFLTEMAR.G + Oxidation (M)
27	1448.6573	1447.6500	1447.6328	11.9	1	95	1e-008	1	U	K.HSFSNDKDDVER.E
29	1482.7172	1481.7099	1481.7150	-3.46	1	106	1.3e-009	1		R.EISKDWSSVFER.S

Database 2

Unigene43444_SALfmcTARAAPEI-3 Mass: 10736 Score: 67 Matches: 1(1) Sequences: 1(1)

gi| 255536839 NADH-ubiquinone oxidoreductase 1, chain, putative [*Ricinus communis*]

Matched peptides shown in bold red.

CPAQAITIEAEEREDGSRRTTRYDIDMTKCIYCGFCQEACPVDAIVEGPNFEFATETHEELLYDKEKLLENGDRWETEIAENLRSE

SLYR

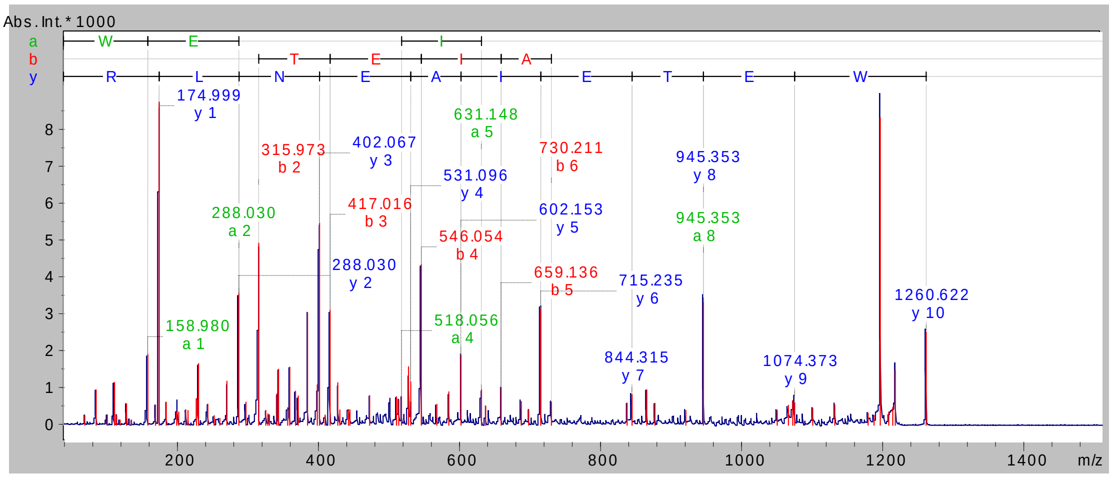

Query	Observed	Mr(expt)	Mr(calc)	ppm	Miss	Score	Expect	Rank	Unique	Peptide
21	1260.6286	1259.6213	1259.6146	5.34	0	67	5.9e-006	1	U	R.WETEIAENLR.S

Spot 2222

NCBInr protein database

Match to: gi|224100271 Mass: 12479 Score: 66 Matches: 1(1) Sequences: 1(1)

hypothetical protein POPTR_0008s20110g [*Populus trichocarpa*]

Matched peptides shown in **bold red**.

1 MATSAVDATG NPIPTSAVLT TASKHIATRC FSENVEFLKC KKKDPNPEK**C**

51 **LDKGQQVTR**C VLGLLKDLHQ KCTKEMDAYV GCMYYYTNEF DLCRKEQQAF

101 EKACPLE

Query	Observed	Mr(expt)	Mr(calc)	ppm	Miss	Score	Expect	Rank	Unique	Peptide
6	1204.6123	1203.6050	1203.6030	1.68	1	66	0.00087	1	U	K.CLDKGQQVTR.C

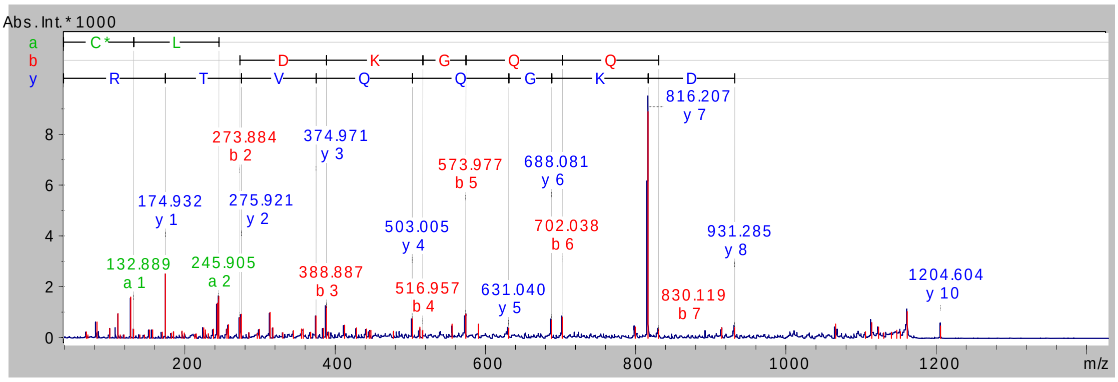

Proteins matching the same set of peptides:

gi|224110528 Mass: 12431 Score: 66 Matches: 1(1) Sequences: 1(1)

NADH-ubiquinone oxidoreductase 19 kDa subunit family protein [*Populus trichocarpa*]

gi|255551591 Mass: 12432 Score: 66 Matches: 1(1) Sequences: 1(1)

NADH dehydrogenase, putative [*Ricinus communis*]

gi|587918182 Mass: 12528 Score: 66 Matches: 1(1) Sequences: 1(1)

hypothetical protein L484_011279 [*Morus notabilis*]

Database 1

Match to: Unigene11554_SeCKS transcribed RNA sequence Mass: 12440 Score: 216 Matches: 5(3) Sequences: 5(3)

gi| 449435544 PREDICTED: NADH dehydrogenase [ubiquinone] 1 alpha subcomplex subunit 8-B-like [*Cucumis sativus*]

Matched peptides shown in **bold red**.

MAANASTAEPIPTSAVLMASSKHINIK**CRDENVAFLK**CKQKDPNPEK**CLDKGQQVTR**CVLSLLKDLHQKCTK**EMDSYSSCMY**

YHTNEFELCRK**EQEEFEKACPLE**

Query	Observed	Mr(expt)	Mr(calc)	ppm	Miss	Score	Expect	Rank	Unique	Peptide
2	938.4507	937.4434	937.4029	43.2	0	28	0.082	1	U	K.EQEEFEK.A
6	1204.6123	1203.6050	1203.6030	1.68	1	66	1.8e-005	1	U	K.CLDKGQQVTR.C
8	1251.6113	1250.6041	1250.6077	-2.95	1	56	0.00016	1	U	K.CRDENVAFLK.C
12	1508.6376	1507.6304	1507.6500	-13.06	1	66	4.5e-006	1	U	K.EQEEFEKACPLE.-
26	2637.8679	2636.8606	2636.9916	-49.68	0	1	0.84	1	U	

K.EMDSYSSCMYYHTNEFELCR.K + Oxidation (M)

Proteins matching the same set of peptides:

Unigene13929_Se200S transcribed RNA sequence Mass: 12440 Score: 216 Matches: 5(3) Sequences: 5(3)

gi| 449435544 PREDICTED: NADH dehydrogenase [ubiquinone] 1 alpha subcomplex subunit 8-B-like [*Cucumis sativus*]

Database 2

Match to: Unigene42479_SALfmcTARAAPEI-3 Mass: 11795 Score: 122 Matches: 3(2) Sequences: 3(2)

gi| 449435544 PREDICTED: NADH dehydrogenase [ubiquinone] 1 alpha subcomplex subunit 8-B-like [*Cucumis sativus*]

Matched peptides shown in **bold red**.

STAEPIPTSAVLMASSKHINIKCRDENVAFLKCKQKDPNPEKCLDKGQQVTRCVLSLLKDLHQKCTKEMDSYSSCMYYHTNEF

ELCRKEQAEFEKACPL

Query	Observed	Mr(expt)	Mr(calc)	ppm	Miss	Score	Expect	Rank	Unique	Peptide
6	1204.6123	1203.6050	1203.6030	1.68	1	66	8e-006	1	U	K.CLDKGQQVTR.C
8	1251.6113	1250.6041	1250.6077	-2.95	1	56	7.4e-005	1	U	K.CRDENVAFLK.C
26	2637.8679	2636.8606	2636.9916	-49.68	0	1	0.92	1	U	

K.EMDSYSSCMYYHTNEFELCR.K + Oxidation (M)

Spot 2336

Database 1

Match to: Unigene32535_SeCKS transcribed RNA sequence Mass: 10740 Score: 388 Matches: 7(5) Sequences: 6(5)

gi| 590692431 NADH-ubiquinone oxidoreductase 11 kDa subunit [*Theobroma cacao*]

Matched peptides shown in **bold red**.

MGFIMEFAENLVLR**LMEDPNER**DKKFR**EHLYDLHDR**CKKTKEMWALPLRPYGFWTFER**HNSQLAWDAQITNVQGRRDPYDE**

VLAESSK

Query	Observed	Mr(expt)	Mr(calc)	ppm	Miss	Score	Expect	Rank	Unique	Peptide
5	1003.4865	1002.4792	1002.4440	35.1	0	(10)	5.8	2	U	R.LMEDPNER.D
6	1019.4903	1018.4830	1018.4389	43.3	0	19	0.7	1	U	R.LMEDPNER.D + Oxidation (M)
12	1197.5955	1196.5882	1196.5574	25.7	0	66	1.2e-005	1	U	R.EHLYDLHDR.C
24	1500.7797	1499.7725	1499.7269	30.3	1	50	0.00057	1	U	K.FREHLYDLHDR.C
25	1508.7712	1507.7639	1507.7154	32.1	1	40	0.0049	1	U	R.RDPYDEVLAESSK.-
27	1554.8497	1553.8425	1553.7912	33.0	0	43	0.002	1	U	M.GFIMEFAENLVLR.L + Oxidation (M)
32	1938.0167	1937.0094	1936.9504	30.5	0	170	4.9e-016	1	U	R.HNSQLAWDAQITNVQGR.R

Proteins matching the same set of peptides:

Unigene33519_Se200S transcribed RNA sequence Mass: 10740 Score: 388 Matches: 7(5) Sequences: 6(5)

gi| 255547718 conserved hypothetical protein [*Ricinus communis*]

Database 2

Match to: Unigene33917_SALfmcTARAAPEI-3 Mass: 7046 Score: 110 Matches: 5(3) Sequences: 4(3)

gi| 590692431 NADH-ubiquinone oxidoreductase 11 kDa subunit [*Theobroma cacao*]

Matched peptides shown in **bold red**.

MGFIMEFAENLVLRLMEDPNERDKKFREHLYDLHDRCKKTKEMWALPLRPYGFWTF

Query	Observed	Mr(expt)	Mr(calc)	ppm	Miss	Score	Expect	Rank	Unique	Peptide
5	1003.4865	1002.4792	1002.4440	35.1	0	(10)	3.3	2	U	R.LMEDPNER.D
6	1019.4903	1018.4830	1018.4389	43.3	0	19	0.39	1	U	R.LMEDPNER.D + Oxidation (M)
12	1197.5955	1196.5882	1196.5574	25.7	0	66	5.4e-006	1	U	R.EHLYDLHDR.C
24	1500.7797	1499.7725	1499.7269	30.3	1	50	0.00028	1	U	K.FREHLYDLHDR.C
27	1554.8497	1553.8425	1553.7912	33.0	0	43	0.0011	1	U	M.GFIMEFAENLVLR.L + Oxidation (M)

Spot 1380

NCBInr protein database

Match to: gi|1706965 Mass: 42204 Score: 91 Matches: 2(1) Sequences: 2(1)

delta-24-sterol methyltransferase [*Triticum aestivum*]

Matched peptides shown in **bold red**.

1 MFVFCLCTRC RICRVSSFPV LLLFMFIHLS YFFLVLLLIL GQFFFTRYEK

51 YHGYYGGKEE SRKSNYTDMV NKYYDLATSF YEYGWGESFH FAHRWNGESL

101 RESIKRHEHF LALQLELKPG MK**VLDVGCGI GGPLR**EIAR**F SSTSVTGLNN**

151 **NDYQITR**GKA LNRSVGLGAT CDFVKADFMK MPFSDNTFDA VYAIEATCHA

201 PDPVGCYKEI YRVLKPGQCF AVYEWCITDH YDPNNATHKR IKDEIELGNG

251 LPDIRSTRQC LQAVKDAGFE VIWDKDLAED SPLPWYLPLD PSRFSLSSFR

301 LTTVGRIITR NMVKVLEYVG LAPEGSQRVS SFLEKAAEGL VEGGKKEIFT

351 PVYFFVVRKP LSE

Query	Observed	Mr(expt)	Mr(calc)	ppm	Miss	Score	Expect	Rank	Unique	Peptide
4	1312.7644	1311.7572	1311.6969	45.9	0	30	2.3	1		K.VLDVGCGIGGPLR.E
11	2017.0165	2016.0092	2015.9548	27.0	0	61	0.0014	1	U	R.FSSTSVTGLNNNDYQITR.G

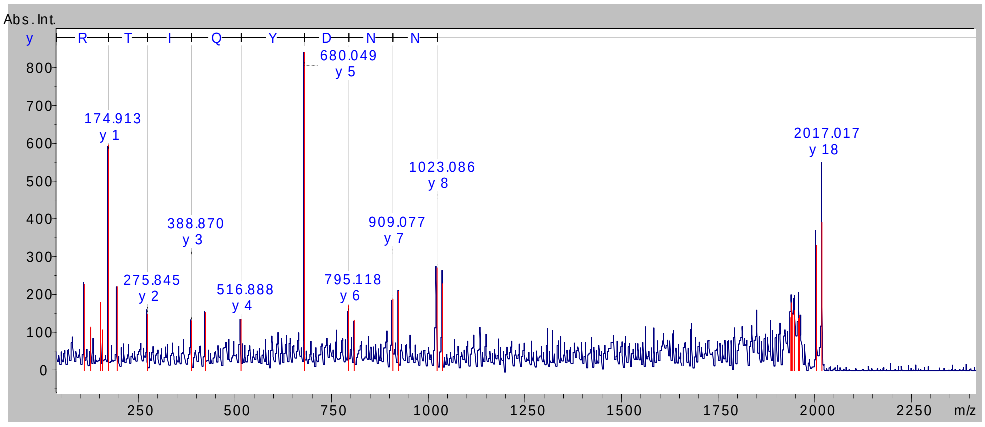

Database 1

Match to: Unigene85516_Se200S transcribed RNA sequence Mass: 19215 Score: 78 Matches: 3(2) Sequences: 3(2)

gi| 470143101 PREDICTED: cycloartenol-C-24-methyltransferase-like [*Fragaria vesca subsp. vesca*]

Matched peptides shown in bold red.

ISLLFSFSLYLGFFNLQILFNHKMSKSGAFDLATGVGGKIEKNDVLSAVDKYEK**YHGYYGGEEEER**KSNYTDMVNKYYDLVTS

FYEFGWGESFHFAHRWNGESLRESIKRHEHFLALQLGLKPGNK**VLDVGCGIGGPLR**EISR**FSSSSVTGLNNNEYQITR**GKELNRI

Query	Observed	Mr(expt)	Mr(calc)	ppm	Miss	Score	Expect	Rank	Unique	Peptide
4	1312.7644	1311.7572	1311.6969	45.9	0	30	0.035	1	U	K.VLDVGCGIGGPLR.E
7	1488.6707	1487.6634	1487.5953	45.8	0	17	0.57	1	U	K.YHGYYGGEEEER.K
11	2017.0165	2016.0092	2015.9548	27.0	0	31	0.033	1	U	R.FSSSSVTGLNNNEYQITR.G

Database 2

Match to: Unigene55463_SALfmcTARAAPEI-3 Mass: 39263 Score: 145 Matches: 5(4) Sequences: 5(4)

gi| 590724517 Sterol methyltransferase 1 [*Theobroma cacao*]

Matched peptides shown in bold red.

MSKSGAFDLATGVGGKIEKNDVLSAVDKYEK**YHGYYGGEEEER**KSNYTDMVNKYYDLVTSFYEFGWGESFHFAHRWNGESL

RESIKRHEHFLALQLGLKPGNK**VLDVGCGIGGPLR**EISR**FSSSSVTGLNNNEYQITR**GKELNRIAGVDKTCNFVKADFMKMPFP

DNSYDAIYAIEATCHAPDAVGCYKEIYRVLKPGQHFAAYEWCMTDAFDAKNQDHQK**IKAEIELGDGLPDIR**STKQCLDALKKA

GFEIIWERDLASDSPVPWYLPLDTNHFSLSSFRLTAVGRFFTRNLVK**TLEYVGLAPSGSLR**VQSFLEKAAEGLVDGGRREIFTPM

YFFLARKPDSDS

Query	Observed	Mr(expt)	Mr(calc)	ppm	Miss	Score	Expect	Rank	Unique	Peptide
4	1312.7644	1311.7572	1311.6969	45.9	0	30	0.02	1	U	K.VLDVGCGIGGPLR.E
6	1462.8511	1461.8438	1461.7827	41.8	0	38	0.0031	1	U	K.TLEYVGLAPSGSLR.V
7	1488.6707	1487.6634	1487.5953	45.8	0	17	0.37	1	U	K.YHGYYGGEEEER.K
9	1638.9714	1637.9641	1637.8988	39.9	1	28	0.02	1	U	K.IKAEIELGDGLPDIR.S
11	2017.0165	2016.0092	2015.9548	27.0	0	31	0.014	1	U	R.FSSSSVTGLNNNEYQITR.G

Spot 741

Database 1

Match to: Unigene18209_Se200S transcribed RNA sequence Mass: 47364 Score: 90 Matches: 2(2) Sequences: 2(2)

gi| 470136427 PREDICTED: beta-glucosidase 24-like [*Fragaria vesca subsp. vesca*]

Matched peptides shown in bold red.

KKKKKKNMAMLYSTPLQSLPPANKLNLKKQNLLVSKIESKNNLNVRLPKSGPYNNNGNKISCNLFSKVDKDALKNELRESATS

MVDWLDPATFTPPNHHLKRKDFPPDFIFGASTSALQTEGNGTEGGRGPSTWDFMIQAGDGKK**AVDSYNLYKEDVR**ILKEMGM

NTYRFSVAWPRILPTGTVEGGINQEGIDFYNKFIDELLANGITPFITLFHFDLPVPLQKEYYGFMRPNIVEQFKEFADLCFEKFGD

RVKYWGTINEPYVFGSYGYKMGLPGDLKNDPLGPYIATHNIILAHAAAAKLYKDKYQKSQGGKIGISLPCKWYVPHSADPKDF

AASKVQLDFSLGWYMDPLTRGEYPESMKKNVKGLPEFTKSEKQMVK**GAFDYIGINYYTAR**YVQHTCNVDAMAGEITIKYSD

KT

Query	Observed	Mr(expt)	Mr(calc)	ppm	Miss	Score	Expect	Rank	Unique	Peptide
15	1571.7666	1570.7593	1570.7627	-2.16	1	44	0.002	1	U	K.AVDSYNLYKEDVR.I
16	1623.7813	1622.7740	1622.7729	0.67	0	47	0.0011	1	U	K.GAFDYIGINYYTAR.Y

Spot 1203

NCBInr protein database

Match to: gi|449438723 Mass: 41143 Score: 233 Matches: 5(3) Sequences: 5(3)

PREDICTED: alpha-1,4-glucan-protein synthase [UDP-forming] 2-like [*Cucumis sativus*]

Matched peptides shown in bold red.

1 MAESASSATS MLKDELDIVI PTIRNLDFLE MWRPFFQPYH LIIVQDGDPS

51 KTIKVPEGFD YELYNRNDIN RILGPRANCI SFKDSACRCF GYMVSKKKYI

101 FTIDDDCFVA SDPSGKPINA LGQHIKNLLC PSTPFFFNTL YDPYRDGADF

151 VRGYPFSLRE GVPTAVSHGL WLNIPDYDAP TQLVKPLERN TRFVDAVLTI

201 PKGTLFPMCG MNLAFDRDLI GAAMYFGLMG DGQPIGRYDD MWAGWCIKVI

251 CDHLGLGVKT GLPYIYHSKA SNPFVNLRKE YKGIFWQEDI IPFFQQVVLP

301 KDCTSVQKCY IELAKQVKDK LSKVDPYFDK LADAMVTWIE AWDDLNPAGA

351 PAKLPNGKA

Query	Observed	Mr(expt)	Mr(calc)	ppm	Miss	Score	Expect	Rank	Unique	Peptide
7	1017.5454	1016.5381	1016.5403	-2.17	0	28	6.5	1		K.ASNPFVNLR.K
13	1210.6599	1209.6526	1209.6540	-1.14	0	22	22	2		K.VICDHLGLGVK.T
22	1501.6977	1500.6904	1500.6885	1.27	0	67	0.00057	1		K.VPEGFDYELYNR.N
27	1843.9288	1842.9216	1842.9152	3.46	1	66	0.00052	1		K.TIKVPEGFDYELYNR.N
36	2447.3950	2446.3878	2446.2625	51.2	1	51	0.0094	1	U	M.AESASSATSMLKDELDIVIPTIR.N

2. gi|502101859 Mass: 40584 Score: 225 Matches: 6(2) Sequences: 5(2)

PREDICTED: UDP-arabinopyranose mutase 2-like [*Cicer arietinum*]

Query	Observed	Mr(expt)	Mr(calc)	ppm	Miss	Score	Expect	Rank	Unique	Peptide
7	1017.5454	1016.5381	1016.5403	-2.17	0	28	6.5	1		K.ASNPFVNLR.K
13	1210.6599	1209.6526	1209.6540	-1.14	0	22	22	2		K.VICDHLGLGVK.T
19	1430.5990	1429.5918	1429.5795	8.58	0	43	0.077	1		R.YDDMWAGWCVK.V
20	1446.5868	1445.5795	1445.5744	3.52	0	(34)	0.52	1		R.YDDMWAGWCVK.V + Oxidation (M)
22	1501.6977	1500.6904	1500.6885	1.27	0	67	0.00057	1		K.VPEGFDYELYNR.N
27	1843.9288	1842.9216	1842.9152	3.46	1	66	0.00052	1		K.TIKVPEGFDYELYNR.N

3. gi|242033731 Mass: 41964 Score: 225 Matches: 6(2) Sequences: 5(2)

hypothetical protein SORBIDRAFT_01g015090 [*Sorghum bicolor*]

Query	Observed	Mr(expt)	Mr(calc)	ppm	Miss	Score	Expect	Rank	Unique	Peptide
7	1017.5454	1016.5381	1016.5403	-2.17	0	28	6.5	1		K.ASNPFVNLR.K
19	1430.5990	1429.5918	1429.5795	8.58	0	43	0.077	1		R.YDDMWAGWCVK.V
20	1446.5868	1445.5795	1445.5744	3.52	0	(34)	0.52	1		R.YDDMWAGWCVK.V + Oxidation (M)
22	1501.6977	1500.6904	1500.6885	1.27	0	67	0.00057	1		K.VPEGFDYELYNR.N
27	1843.9288	1842.9216	1842.9152	3.46	1	66	0.00052	1		K.TIKVPEGFDYELYNR.N
29	2120.9684	2119.9611	2119.9772	-7.61	1	21	15	1		K.YIYTIDDDCFVAKDPTGK.D

4. gi|242058779 Mass: 40473 Score: 219 Matches: 6(2) Sequences: 5(2)

hypothetical protein SORBIDRAFT_03g035320 [*Sorghum bicolor*]

Query	Observed	Mr(expt)	Mr(calc)	ppm	Miss	Score	Expect	Rank	Unique	Peptide
13	1210.6599	1209.6526	1209.6540	-1.14	0	22	22		2	K.VICDHLGLGVK.T
19	1430.5990	1429.5918	1429.5795	8.58	0	43	0.077	1		R.YDDMWAGWCVK.V
20	1446.5868	1445.5795	1445.5744	3.52	0	(34)	0.52	1		R.YDDMWAGWCVK.V + Oxidation (M)
22	1501.6977	1500.6904	1500.6885	1.27	0	67	0.00057	1		K.VPEGFDYELYNR.N
27	1843.9288	1842.9216	1842.9152	3.46	1	66	0.00052	1		K.TIKVPEGFDYELYNR.N

29	2120.9684	2119.9611	2119.9772	-7.61	1	21	15	1		K.YIYTIDDDCFVAKDPTGK.D

Database 1

Match to: Unigene45970_SeCKS transcribed RNA sequence Mass: 18774 Score: 172 Matches: 3(3) Sequences: 3(3)

gi| 34582499 RecName: Full=Alpha-1,4-glucan-protein synthase [UDP-forming] 2; AltName: Full=Reversibly glycosylated polypeptide 2; Short=RGP2; AltName: Full=UDP-glucose:protein transglucosylase 2; Short=UPTG 2 [*Solanum tuberosum*]

Matched peptides shown in **bold red**.

LIIVQDGDPSKTIKVPEGFDYELYNRNDINRILGPKASCISFKDSACRCFGYMVSKKKYIYTIDDDCFVAQEPSGKNINALAQHIE

NLLSPSTPHFFNTLYDPYREGADFVRGYPFSMREGAHTAVSHGLWLNVPDYDAPTQLVKPRERNTRYVDAVLTIPKGS

Query	Observed	Mr(expt)	Mr(calc)	ppm	Miss	Score	Expect	Rank	Unique	Peptide
22	1501.6977	1500.6904	1500.6885	1.27	0	67	8.4e-006	1	U	K.VPEGFDYELYNR.N
27	1843.9288	1842.9216	1842.9152	3.46	1	66	1.1e-005	1	U	K.TIKVPEGFDYELYNR.N
29	2120.9684	2119.9611	2119.9408	9.55	0	39	0.0037	1	U	K.YIYTIDDDCFVAQEPSGK.N

Proteins matching the same set of peptides:

Unigene47058_Se200S transcribed RNA sequence Mass: 18923 Score: 172 Matches: 3(3) Sequences: 3(3)

gi| 641851444 hypothetical protein CISIN_1g018080mg [*Citrus sinensis*]

2. Unigene5362_Se200S transcribed RNA sequence Mass: 17360 Score: 107 Matches: 5(2) Sequences: 4(1)

gi| 353441070 putative reversibly glycosylatable polypeptide [*Elaeis guineensis*]

Query	Observed	Mr(expt)	Mr(calc)	ppm	Miss	Score	Expect	Rank	Unique	Peptide
7	1017.5454	1016.5381	1016.5403	-2.17	0	28	0.13	1	U	K.ASNPFVNLR.K
12	1197.6486	1196.6413	1196.6441	-2.36	1	15	1.5	1	U	K.LGKVDEYFVK.L
13	1210.6599	1209.6526	1209.6540	-1.14	0	22	0.34	1	U	K.VICDHLGLGVK.T
19	1430.5990	1429.5918	1429.5795	8.58	0	43	0.00068	1	U	R.YDDMWAGWCVK.V
20	1446.5868	1445.5795	1445.5744	3.52	0	(34)	0.0034	1	U	R.YDDMWAGWCVK.V + Oxidation (M)

Proteins matching the same set of peptides:

Unigene8956_SeCKS transcribed RNA sequence Mass: 17736 Score: 107 Matches: 5(2) Sequences: 4(1)

gi| 353441070 putative reversibly glycosylatable polypeptide [*Elaeis guineensis*]

Spot 1232

NCBInr protein database

Match to: gi|449438723 Mass: 41143 Score: 254 Matches: 5(3) Sequences: 5(3)

PREDICTED: alpha-1,4-glucan-protein synthase [UDP-forming] 2-like [*Cucumis sativus*]

Matched peptides shown in **bold red**.

1 M**AESASSATS MLKDELDIVI PTIR**NLDFLE MWRPFFQPYH LIIVQDGDPS

51 K**TIKVPEGFD YELYNR**NDIN RILGPRANCI SFKDSACRCF GYMVSKKKYI

101 FTIDDDCFVA SDPSGKPINA LGQHIKNLLC PSTPFFFNTL YDPYRDGADF

151 VRGYPFSLRE GVPTAVSHGL WLNIPDYDAP TQLVKPLERN TRFVDAVLTI

201 PKGTLFPMCG MNLAFDRDLI GAAMYFGLMG DGQPIGRYDD MWAGWCIKVI

251 CDHLGLGVK**T GLPYIYHSKA SNPFVNLR**KE YKGIFWQEDI IPFFQQVVLP

301 KDCTSVQKCY IELAKQVKDK LSKVDPYFDK LADAMVTWIE AWDDLNPAGA

351 PAKLPNGKA

Query	Observed	Mr(expt)	Mr(calc)	ppm	Miss	Score	Expect	Rank	Unique	Peptide
6	1017.5788	1016.5715	1016.5403	30.7	0	32	2.3	2		K.ASNPFVNLR.K
7	1178.6555	1177.6482	1177.6131	29.8	0	25	8.6	1	U	K.TGLPYIYHSK.A

17	1501.7302	1500.7229	1500.6885	22.9	0	89	3.3e-06	1		K.VPEGFDYELYNR.N
20	1843.9647	1842.9574	1842.9152	22.9	1	65	0.00074	1		K.TIKVPEGFDYELYNR.N
31	2447.4878	2446.4805	2446.2625	89.1	1	42	0.035	1	U	M.AESASSATSMLKDELDIVIPTIR.N

2. gi|350537551 Mass: 41736 Score: 165 Matches: 3(2) Sequences: 3(2)

UDP-glucose:protein transglucosylase-like protein SlUPTG1 [*Solanum lycopersicum*]

Query	Observed	Mr(expt)	Mr(calc)	ppm	Miss	Score	Expect	Rank	Unique	Peptide
4	857.4258	856.4186	856.3902	33.2	0	11	2.4e+02	5		R.GYPFSMR.E
17	1501.7302	1500.7229	1500.6885	22.9	0	89	3.3e-06	1		K.VPEGFDYELYNR.N
20	1843.9647	1842.9574	1842.9152	22.9	1	65	0.00074	1		K.TIKVPEGFDYELYNR.N

Database 1

Match to: Unigene45970_SeCKS transcribed RNA sequence Mass: 18774 Score: 165 Matches: 3(2) Sequences: 3(2)

gi| 34582499 RecName: Full=Alpha-1,4-glucan-protein synthase [UDP-forming] 2; AltName: Full=Reversibly glycosylated polypeptide 2; Short=RGP2; AltName: Full=UDP-glucose:protein transglucosylase 2; Short=UPTG 2 [*Solanum tuberosum*]

Matched peptides shown in bold red.

LIIVQDGDPSK**TIKVPEGFDYELYNR**NDINRILGPKASCISFKDSACRCFGYMVSKKKYIYTIDDDCFVAQEPSGKNINALAQHIE
NLLSPSTPHFFNTLYDPYREGADFVR**GYPFSMR**EGAHTAVSHGLWLNVPDYDAPTQLVKPRERNTRYVDAVLTIPKGS

Query	Observed	Mr(expt)	Mr(calc)	ppm	Miss	Score	Expect	Rank	Unique	Peptide
4	857.4258	856.4186	856.3902	33.2	0	11	3	1	U	R.GYPFSMR.E
17	1501.7302	1500.7229	1500.6885	22.9	0	89	6.4e-008	1	U	K.VPEGFDYELYNR.N
20	1843.9647	1842.9574	1842.9152	22.9	1	65	1.5e-005	1	U	K.TIKVPEGFDYELYNR.N

Proteins matching the same set of peptides:

Unigene47058_Se200S transcribed RNA sequence Mass: 18923 Score: 165 Matches: 3(2) Sequences: 3(2)

gi| 641851444 hypothetical protein CISIN_1g018080mg [*Citrus sinensis*]

Spot 449

Database 1

Match to: Unigene 44116_SeCKS transcribed RNA sequence Mass: 34444 Score: 61 Matches: 2(2) Sequences: 2(2)

gi| 470123037 PREDICTED: probable inactive purple acid phosphatase 27-like [*Fragaria vesca subsp. vesca*]

Matched peptides shown in bold red.

GALNTTDQLTKDLDNIDAVFLIGDLPYANGYVSQWDQFTAQVEPIASVKPFMVASGNHERDVPDTGSFYANNDSGGECGVPAE
TTFFVPAKNRAKFWYSADYGMFHFCIADTEHDWRAGSEQYK**FIEECLASANR**HKQPWLIFAGHRPLGYSSNK**WFGEEGSFEE
PMGR**DDLQKLWQKYRVDLAFFGHVHNYERTCPVYQNQCVKEGTSHYSGVVNGTIHVVAGGGGSHLNKFSPLKTVWSIYRDE
DFGYVKLTAYNYSSMKFEYMKSSDGKVYDTFTISRDYKDVLSCVHDSCEPYTLAA

Query	Observed	Mr(expt)	Mr(calc)	ppm	Miss	Score	Expect	Rank	Unique	Peptide
7	1309.6667	1308.6594	1308.6132	35.3	0	31	0.041	2	U	K.FIEECLASANR.H
12	1657.7344	1656.7271	1656.6879	23.7	0	29	0.031	1	U	K.WFGEEGSFEEPMGR.D

Database 2

Match to: Unigene40990_SALfmcTARAAPEI-3 Mass: 14316 Score: 49 Matches: 1(1) Sequences: 1(1)

gi| 565393581 PREDICTED: probable inactive purple acid phosphatase 27-like [*Solanum tuberosum*]

Matched peptides shown in bold red.

SPARTVGWRDPGFIHTSYLKDLWPNTLYSYRMGHKLSDGSCVWSKKFQFK**SPPVPGESSLQR**IVIFGDLGKGERDGSNEYANY
QPGALNTTDQLTKDLDNIDAVFLIGDLPYANGYVSQWDQFTAQV

Query	Observed	Mr(expt)	Mr(calc)	ppm	Miss	Score	Expect	Rank	Unique	Peptide
5	1253.6959	1252.6887	1252.6412	37.9	0	49	0.00027	1	U	K.SPPVPGESSLQR.I

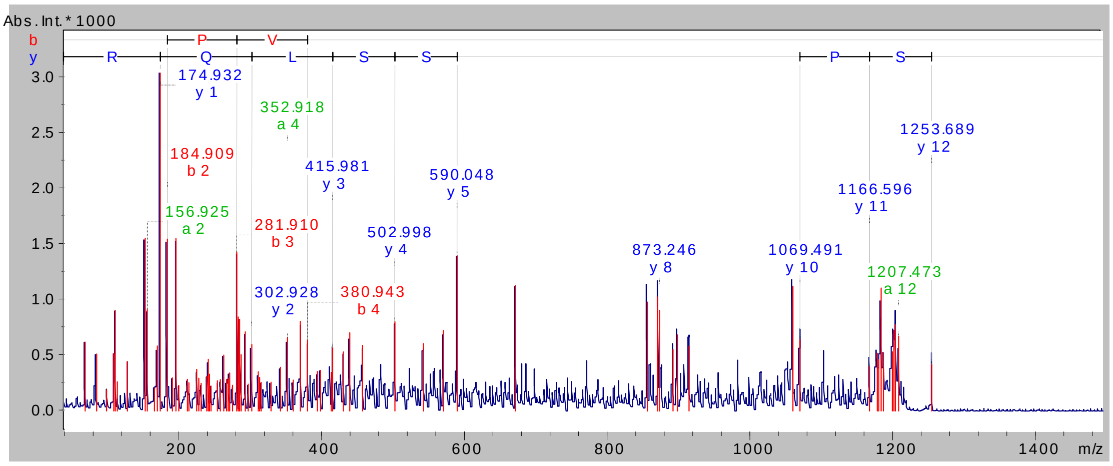

Spot 459

NCBInr protein database

Match to: gi|460399851 Mass: 69177 Score: 70 Matches: 2(1) Sequences: 1(1)

PREDICTED: probable inactive purple acid phosphatase 27-like [*Solanum lycopersicum*]

Matched peptides shown in **bold red**.

1 MKKFVVCFLV LLGVASGHSG EQPLSNIAIH KATVALDASL TIKAYPFILA

51 PKGGDTEWVT LHLDNPNPSH DDWVGVFSPA KFNGSTCYLE NDGKQQPPYI

101 CTAPIKYNFA NFSNPDYAKT GNTSLKFQLI NQRADFSFAL FTGGLSNPKL

151 VGVSNYISFA NPKAPLYPRL ALGKSWNEMT LTWTSGYNLL EAVPFIEWGR

201 KGDPQHRSPA GTLTFDRNTM CGSPARTVGW RDPGFIHTSF MKDLWPSTLY

251 TYKMGHMLSN GSYVWSKMYS FRSSPYPGQD SLQRVIIFGD MGKAERDGSN

301 EYSNYQPGSL NTTDQLINDL KNIDIVFHIG DITYANGYIS QWDQFTAQVE

351 PVASTVPYMI ASGNHERDWP GTGSFYDVMD SGGECGVLAQ TMFYVPADNR

401 ANFWYSTDYG MFHFCIADSE HDWREGSEQY RFIEHCLASV DRQKQPWLIF

451 AAHRVLGYSS DK**WYGLEGSF EEPMGR**ESLQ KLWQKYKVDI AFYGHVHNYE

501 RTCPIYQNQC VNSERSHYSG IVNGTIHVVV GGGGSHLSEF TPINTTWSLH

551 RDYDWGFVKL TAFNHSSLLF EYKKSRDGKV YDSFTISRDY KDVLACVHDG

601 CEPTTFAS

Query	Observed	Mr(expt)	Mr(calc)	ppm	Miss	Score	Expect	Rank	Unique	Peptide
25	1657.7313	1656.7240	1656.7242	-0.15	0	70	0.00019	1	U	K.WYGLEGSFEEPMGR.E
26	1673.7303	1672.7230	1672.7191	2.31	0	(15)	55	6	U	K.WYGLEGSFEEPMGR.E + Oxidation (M)

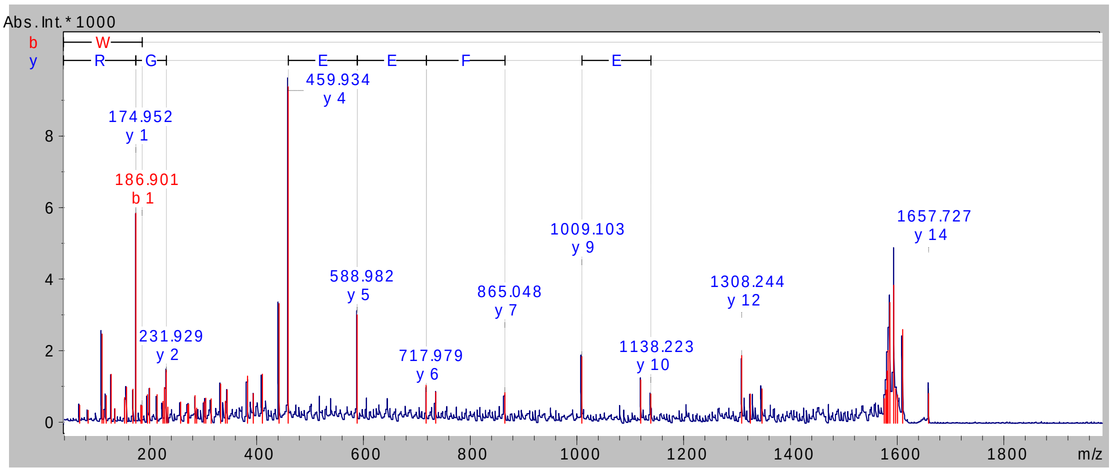

Database 1

Match to: Unigene 44116_SeCKS transcribed RNA sequence Mass: 34444 Score: 147 Matches: 4(3) Sequences: 3(3)

gi| 470123037 PREDICTED: probable inactive purple acid phosphatase 27-like [*Fragaria vesca subsp. vesca*]

Matched peptides shown in bold red.

GALNTTDQLTKDLDNIDAVFLIGDLPYANGYVSQWDQFTAQVEPIASVKPFMVASGNHERDVPDTGSFYANNDSGGECGVPAE

TTFFVPAKNRAKFWYSADYGMFHFCIADTEHDWRAGSEQYKFIEECLASANRHKQPWLIFAGHRPLGYSSNKWFGEEGSFEE

PMGRDDLQKLWQKYRVDLAFFGHVHNYERTCPVYQNQCVKEGTSHYSGVVNGTIHVVAGGGGSHLNKFSPLKTVWSIYRDE

DFGYVKLTAYNYSSMKFEYMKSSDGKVYDTFTISRDYKDVLSCVHDSCEPYTLAA

Query	Observed	Mr(expt)	Mr(calc)	ppm	Miss	Score	Expect	Rank	Unique	Peptide
16	1309.6625	1308.6553	1308.6132	32.1	0	45	0.0018	2	U	K.FIEECLASANR.H
18	1396.6802	1395.6729	1395.6275	32.5	0	37	0.012	1	U	R.TCPVYQNQCVK.E
25	1657.7313	1656.7240	1656.6879	21.8	0	65	8.8e-006	1	U	K.WFGEEGSFEEPMGR.D
26	1673.7303	1672.7230	1672.6828	24.1	0	(18)	0.34	1	U	K.WFGEEGSFEEPMGR.D + Oxidation (M)

Proteins matching the same set of peptides:

Unigene47262_Se200S transcribed RNA sequence Mass: 34387 Score: 147 Matches: 4(3) Sequences: 3(3)

gi| 470123037 PREDICTED: probable inactive purple acid phosphatase 27-like [*Fragaria vesca subsp. vesca*]

Database 2

Match to: Unigene16240_SALfmcTARAAPEI-3 Mass: 19928 Score: 70 Matches: 2(1) Sequences: 1(1)

gi| 502176175 PREDICTED: probable inactive purple acid phosphatase 27-like [*Cicer arietinum*]

Matched peptides shown in bold red.

SQWDQFTSQVEPIASTVPYMIASGNHERDWPNSGSFYDGVDSGGECGVLAQTMFYVPAENRDKFWYKADYGMFRFCIADTE

HDWREGTEQYKFIEHCLSTADRQKQPWLIFAAHRVLGYSSDKWYGLEGSFEEPMGREALQKLWQRYKVDIAFFGHVHNYERS

CPIYQ

Query	Observed	Mr(expt)	Mr(calc)	ppm	Miss	Score	Expect	Rank	Unique	Peptide
25	1657.7313	1656.7240	1656.7242	-0.15	0	70	1.8e-006	1	U	K.WYGLEGSFEEPMGR.E
26	1673.7303	1672.7230	1672.7191	2.31	0	(15)	0.51	1	U	K.WYGLEGSFEEPMGR.E + Oxidation (M)

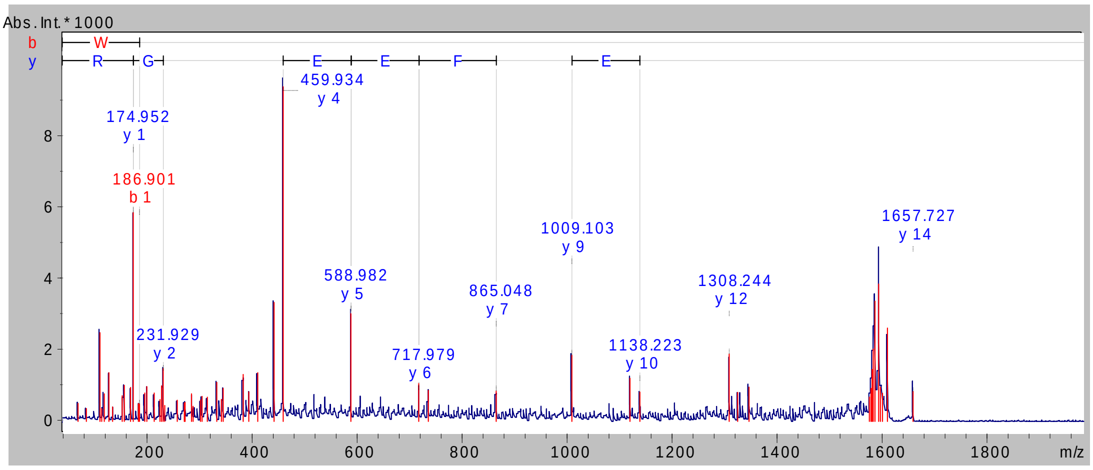

Spot 739

NCBInr protein database

Match to: gi|527200206 Mass: 58761 Score: 150 Matches: 2(1) Sequences: 2(1)

mitochondrial processing peptidase [*Genlisea aurea*]

Matched peptides shown in **bold red.**

1 MTIRQLLNLA RRSRRNPHVL VPLQPLSTAV SASAEVSLPS PPPPTAMIYD

51 RLAESVKEKL KRLEEPDPRF LRYNSPHPAV VSHTDILPAP LTRVTTLPNG

101 LRIATESNLA LKTATVGVFI DAGSRFESDE SNGTAHFLEH MIFKGTDRRT

151 ARELEEEIEN MGGHLNAYTS REQTTYYAKV LDKDVPVALD ILSDILQNSK

201 FDEHRINRER DVILREMEEV EGQTEEVIFD HLHATAFQYT PLGRTILGPA

251 ENVKRIGKDH LKSYISTHYT APRTVVVASG AVKHEDIVEQ VKKLFTKLSS

301 SPTTASELVA KEPAIFTGSE VRMLDDDIPL AQFAVAFEGA SWTDPDSIAL

351 MVMQSMLGAW NKNAGGGKHM GSGLAQRVGI NEIAESIMAF NTNYKDTGLF

401 GVYAVAKPDC LDDLSHAIVN EITKLCYRVS EADVIRAQNQ LK**SSLLLHID**

451 **GTSPVAEDIG RQMLTYGR**RI PYAELFARIE AVDASTVKRV ANRFIFDRDV

501 AISAIGPIQG LPDYNWFRRR TYWLRY

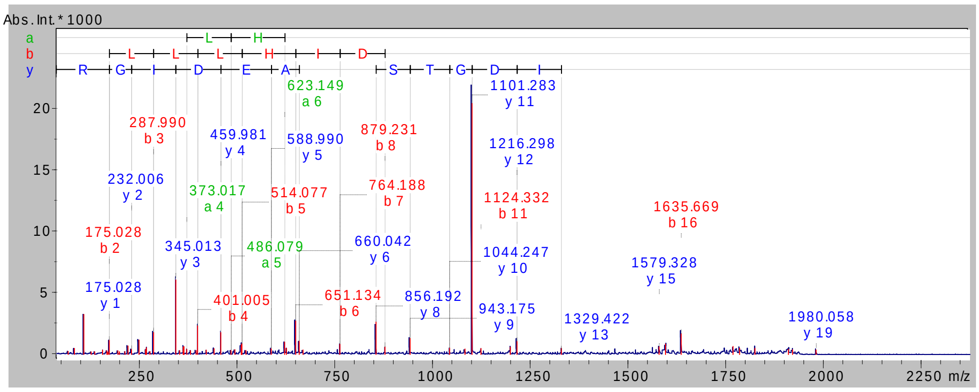

Query	Observed	Mr(expt)	Mr(calc)	ppm	Miss	Score	Expect	Rank	Unique	Peptide
5	884.4782	883.4709	883.4222	55.2	0	31	2.1	1	U	R.QMLTYGR.R + Oxidation (M)
23	1980.0374	1979.0301	1979.0324	-1.15	0	119	2.7e-09	1		K.SSLLLHIDGTSPVAEDIGR.Q

Database 1

Match to: Unigene 18209 Mass: 25712 Score: 240 Matches: 8(2) Sequences: 8(2)

gi| 225452974 PREDICTED: probable mitochondrial-processing peptidase subunit beta [*Vitis vinifera*]

Matched peptides shown in bold red.

KLSTDPTTASQLVAKEPATFTGSEVRMIDDDLPLAQFAVAFNGASYTDPDSIALMVMQSMLGSWNKNAGGGKHMGSELAQRV

CINGIAESMMAFNTNYKDTGLFGVYAVAKPDSLDDLAWAIMHEISKMCYRVSDADVTRACNQLKSSLLLHIDGTSPVAEDIGR

QFLTYGRRIPFAELFARIDAVDASTVKRVANRFIYDRDIAIAAMGPVKNLPDYNWFRRRTYWNRY

Query	Observed	Mr(expt)	Mr(calc)	ppm	Miss	Score	Expect	Rank	Unique	Peptide
1	713.3792	712.3719	712.3544	24.5	0	8	4.5	1	U	R.FIYDR.D
5	884.4782	883.4709	883.4552	17.8	0	31	0.033	1	U	R.QFLTYGR.R
13	1063.6086	1062.6014	1062.5862	14.3	0	18	0.71	1	U	R.IPFAELFAR.I
16	1193.5964	1192.5891	1192.5724	14.0	0	11	4.6	1	U	K.EPATFTGSEVR.M
17	1219.7076	1218.7003	1218.6873	10.7	1	4	15	6	U	R.RIPFAELFAR.I
18	1224.5966	1223.5893	1223.5723	13.8	0	28	0.08	1	U	K.NLPDYNWFR.R
22	1795.9511	1794.9438	1794.9338	5.57	1	20	0.39	1	U	R.FIYDRDIAIAAMGPVK.N + Oxidation (M)
23	1980.0374	1979.0301	1979.0324	-1.15	0	119	5.6e-011	1	U	K.SSLLLHIDGTSPVAEDIGR.Q

Database 2

Match to: Unigene51650_SALfmcTARAAPEI-3 Mass: 26173 Score: 127 Matches: 8(3) Sequences: 8(3)

gi| 225452974 PREDICTED: probable mitochondrial-processing peptidase subunit beta [*Vitis vinifera*]

Matched peptides shown in bold red.

KSFVKLSTDPTTASQLVAKEPATFTGSEVRMIDDDLPLAQFAVAFNGASYTDPDSIALMVMQSMLGSWNKNAGGGKHMGSEL

AQRVCINGIAESMMAFNTNYKDTGLFGVYAVAKPDSLDDLAWAIMHEISKMCYRVSDADVTRACNQLKSSLLLHIDGTSPVAE

DIGRQFLTYGRRIPFAELFARIDAVDASTVKRVANRFIYDRDIAIAAMGPVKNLPDYNWFRRRTYWNRY

Query	Observed	Mr(expt)	Mr(calc)	ppm	Miss	Score	Expect	Rank	Unique	Peptide
1	713.3792	712.3719	712.3544	24.5	0	8	2.5	1	U	R.FIYDR.D
5	884.4782	883.4709	883.4552	17.8	0	31	0.018	1	U	R.QFLTYGR.R
13	1063.6086	1062.6014	1062.5862	14.3	0	18	0.42	1	U	R.IPFAELFAR.I
16	1193.5964	1192.5891	1192.5724	14.0	0	11	2.2	1	U	K.EPATFTGSEVR.M
17	1219.7076	1218.7003	1218.6873	10.7	1	4	10	2	U	R.RIPFAELFAR.I
18	1224.5966	1223.5893	1223.5723	13.8	0	28	0.038	1	U	K.NLPDYNWFR.R
22	1795.9511	1794.9438	1794.9338	5.57	1	20	0.19	1	U	R.FIYDRDIAIAAMGPVK.N + Oxidation (M)
23	1980.0374	1979.0301	1979.0324	-1.15	0	119	2.5e-011	1	U	K.SSLLLHIDGTSPVAEDIGR.Q

Spot 913

NCBInr protein database

Match to: gi|118488735 Mass: 45306 Score: 230 Matches: 5(2) Sequences: 5(2)

unknown [*Populus trichocarpa*]

Matched peptides shown in bold red.

1 MDGTEGLVRG QPVLNTGSPI TVPVGRATLG RIINVIGEAI DEKGDLKTEH

51 YLPIHREAPS FVEQATEQQV LVTGIKVVDL LAPYQRGGKI GLFGGAGVGK

101 TVLIMELINN VAKAHGGFSV FAGVGERTRE GNDLYREMIE SGVIKLGDQQ

151 AESKCALVYG QMNEPPGARA RVGLTGLTVA EHFRDAEGQD VLLFIDNIFR

201 FTQANSEVSA LLGRIPSAVG YQPTLATDLG GLQERITTTK KGSITSVQAI

251 YVPADDLTDP APATTFAHLD ATTVLSRQIS ELGIYPAVDP LDSTSRMLSP

301 HILGEEHYNT ARGVQKVLQN YKNLQDIIAI LGMDELSEDD KLTVARARKI

351 QRFLSQPFHV AEVFTGAPGK YVELKEGVQS FQGVLDGKYD DLPEQSFYMV

401 GGIEEVIAKA EKISKESAAS

Query	Observed	Mr(expt)	Mr(calc)	ppm	Miss	Score	Expect	Rank	Unique	Peptide
13	1173.6463	1172.6390	1172.6553	-13.94	0	38	0.48	1	U	K.VVDLLAPYQR.G
19	1390.6533	1389.6460	1389.6790	-23.70	0	75	9.3e-05	1	U	K.AHGGFSVFAGVGER.T
25	1492.7326	1491.7254	1491.7681	-28.67	0	37	0.6	1	U	R.FTQANSEVSALLGR.I
27	1691.8828	1690.8755	1690.9366	-36.15	0	32	1.5	1	U	R.GQPVLNTGSPITVPVGR.A
35	1864.8987	1863.8915	1863.9367	-24.26	0	48	0.036	1	U	R.DAEGQDVLLFIDNIFR.F

Proteins matching the same set of peptides:

gi|224099437 Mass: 60047 Score: 230 Matches: 5(2) Sequences: 5(2)

H+-transporting two-sector ATPase family protein [*Populus trichocarpa*]

gi|242129046 Mass: 59987 Score: 230 Matches: 5(2) Sequences: 5(2)

ATP synthase beta subunit 1 [*Gossypium hirsutum*]

gi|565389366 Mass: 59924 Score: 230 Matches: 5(2) Sequences: 5(2)

PREDICTED: ATP synthase subunit beta, mitochondrial-like [*Solanum tuberosum*]

gi|566190494 Mass: 59888 Score: 230 Matches: 5(2) Sequences: 5(2)

H+-transporting two-sector ATPase family protein [*Populus trichocarpa*]

2. gi|225445041 Mass: 54552 Score: 167 Matches: 4(2) Sequences: 3(2)

PREDICTED: mitochondrial-processing peptidase subunit alpha [*Vitis vinifera*]

Query	Observed	Mr(expt)	Mr(calc)	ppm	Miss	Score	Expect	Rank	Unique	Peptide
11	1149.5796	1148.5723	1148.5859	-11.89	0	29	4.2	2	U	K.TAVLMNLESR.M + Oxidation (M)
17	1302.5996	1301.5923	1301.5962	-2.97	0	(47)	0.061	1		R.EQMGYTFDALK.T
18	1318.5706	1317.5633	1317.5911	-21.07	0	71	0.00019	1		R.EQMGYTFDALK.T + Oxidation (M)
23	1460.6920	1459.6848	1459.7267	-28.71	0	67	0.00054	1	U	R.EVEAIGGNVTASASR.E

Database 1

Match to: Unigene32364_Se200S transcribed RNA sequence Mass: 31677 Score: 451 Matches: 7(6) Sequences: 5(5)

gi| 225445041 PREDICTED: mitochondrial-processing peptidase subunit alpha [*Vitis vinifera*]

Matched peptides shown in bold red.

AQNHLTSSLNFLIFSSINQSIMYRVASSRLRALKKAREGSSCLARYASSSAVAAKANVSGGFLSSLFGGGSKSLPPLDFPLEGVTV

PPPLPDYVEPAKTKITTLPNGVKVASQASPNPVASIGLYVDSGSIYETPFSFGATHLLERMAFKTTTNRSHLRLVREVEAIGGNVT

ASASREQMGYTFDALKTYVPEMVELLVDSVRNPAFLDWEVKETLEKLKHEINEASNNPQGLLVEAIHSAGYSGALSNSLMAQ

EATISRLDSSVLEQFVADNYTAPRMVLAASGVEHEDLLSIA

Query	Observed	Mr(expt)	Mr(calc)	ppm	Miss	Score	Expect	Rank	Unique	Peptide
14	1218.5981	1217.5908	1217.6081	-14.14	0	80	5.8e-007	1		R.NPAFLDWEVK.E
17	1302.5996	1301.5923	1301.5962	-2.97	0	(47)	0.00076	1		R.EQMGYTFDALK.T
18	1318.5706	1317.5633	1317.5911	-21.07	0	71	1.8e-006	1		R.EQMGYTFDALK.T + Oxidation (M)

23 1460.6920 1459.6848 1459.7267 -28.71 0 67 8.7e-006 1 R.EVEAIGGNVTASASR.E

32 1749.8702 1748.8629 1748.9019 -22.26 0 (28) 0.074 1

K.TYVPEMVELLVDSVR.N

33 1765.8565 1764.8493 1764.8968 -26.93 0 73 2.5e-006 1

K.TYVPEMVELLVDSVR.N + Oxidation (M)

36 2024.9406 2023.9333 2023.9851 -25.57 0 160 3.5e-015 1 U

R.LDSSVLEQFVADNYTAPR.M

2. Unigene31944_SeCKS transcribed RNA sequence Mass: 25622 Score: 352 Matches: 7(6) Sequences: 5(5)

gi| 225445041 PREDICTED: mitochondrial-processing peptidase subunit alpha [*Vitis vinifera*]

Query Observed Mr(expt) Mr(calc) ppm Miss Score Expect Rank Unique Peptide

14 1218.5981 1217.5908 1217.6081 -14.14 0 80 5.8e-007 1 R.NPAFLDWEVK.E

17 1302.5996 1301.5923 1301.5962 -2.97 0 (47) 0.00076 1 R.EQMGYTFDALK.T

18 1318.5706 1317.5633 1317.5911 -21.07 0 71 1.8e-006 1 R.EQMGYTFDALK.T + Oxidation

(M)

23 1460.6920 1459.6848 1459.7267 -28.71 0 67 8.7e-006 1 R.EVEAIGGNVTASASR.E

26 1572.7222 1571.7149 1571.7580 -27.42 0 61 2.6e-005 1 U K.SNASGGFLSSLFGGGSK.S

32 1749.8702 1748.8629 1748.9019 -22.26 0 (28) 0.074 1 K.TYVPEMVELLVDSVR.N

33 1765.8565 1764.8493 1764.8968 -26.93 0 73 2.5e-006 1 K.TYVPEMVELLVDSVR.N +

Oxidation (M)

3. Unigene46932_Se200S transcribed RNA sequence Mass: 34029 Score: 203 Matches: 5(4) Sequences: 5(4)

gi| 645270102 PREDICTED: ATP synthase subunit beta, mitochondrial [*Prunus mume*]

Query Observed Mr(expt) Mr(calc) ppm Miss Score Expect Rank Unique Peptide

17 1302.5996 1301.5923 1301.6364 -33.86 0 30 0.038 2 U R.ILGEEHYNTAR.G

19 1390.6533 1389.6460 1389.6790 -23.70 0 75 1.5e-006 1 U K.AHGGFSVFAGVGER.T

20 1399.7342 1398.7269 1398.7620 -25.05 0 14 1.8 1 U R.VGLTGLTVAEHFR.D

25 1492.7326 1491.7254 1491.7681 -28.67 0 37 0.012 1 U R.FTQANSEVSALLGR.I

35 1864.8987 1863.8915 1863.9367 -24.26 0 48 0.00076 1 U R.DAEGQDVLLFIDNIFR.F

4. Unigene1186_SeCKS transcribed RNA sequence Mass: 24359 Score: 115 Matches: 4(2) Sequences: 3(2)

gi| 225445041 PREDICTED: mitochondrial-processing peptidase subunit alpha [*Vitis vinifera*]

Query Observed Mr(expt) Mr(calc) ppm Miss Score Expect Rank Unique Peptide

7 1023.5749 1022.5677 1022.5800 -12.10 0 47 0.00092 1 U R.KPVEYFLK.T

10 1133.5827 1132.5755 1132.5910 -13.72 0 (13) 4.1 2 U K.SAILMNLESR.M

11 1149.5796 1148.5723 1148.5859 -11.88 0 29 0.082 1 U K.SAILMNLESR.M +

Oxidation (M)

37 2038.0693 2037.0620 2037.1218 -29.39 0 39 0.0045 1 U

K.ELIAVTRPGEVTQIQLDR.A

Proteins matching the same set of peptides:

Unigene15238_Se200S transcribed RNA sequence Mass: 25717 Score: 115 Matches: 4(2) Sequences: 3(2)

gi| 225445041 PREDICTED: mitochondrial-processing peptidase subunit alpha [*Vitis vinifera*]

Database 2

Match to: Unigene41649_SALfmcTARAAPEI-3 Mass: 14289 Score: 369 Matches: 7(7) Sequences: 5(5)

gi| 225445041 PREDICTED: mitochondrial-processing peptidase subunit alpha [*Vitis vinifera*]

Matched peptides shown in **bold red**.

LVR**EVEAIGGNVTASASREQMGYTFDALKTYVPEMVELLVDSVRNPAFLDWEVK**ETLEKLKHEINEASNNPQGLLVEAIHSAG

YSGALSNSLMAQEATISRLDSSVLEQFVADNYTAPRMVLAASGVEHED

Query	Observed	Mr(expt)	Mr(calc)	ppm	Miss	Score	Expect	Rank	Unique	Peptide
14	1218.5981	1217.5908	1217.6081	-14.14	0	80	2.9e-007	1	U	R.NPAFLDWEVK.E
17	1302.5996	1301.5923	1301.5962	-2.97	0	(47)	0.00061	1	U	R.EQMGYTFDALK.T
18	1318.5706	1317.5633	1317.5911	-21.07	0	71	1.7e-006	1	U	R.EQMGYTFDALK.T + Oxidation (M)
23	1460.6920	1459.6848	1459.7267	-28.71	0	67	4.9e-006	1	U	R.EVEAIGGNVTASASR.E
32	1749.8702	1748.8629	1748.9019	-22.26	0	(28)	0.03	1	U	K.TYVPEMVELLVDSVR.N
33	1765.8565	1764.8493	1764.8968	-26.93	0	73	1.1e-006	1	U	K.TYVPEMVELLVDSVR.N + Oxidation (M)
36	2024.9406	2023.9333	2023.9851	-25.57	0	160	1.5e-015	1	U	R.LDSSVLEQFVADNYTAPR.M

Spot 914

NCBInr protein database

Match to: gi|224035589 Mass: 50276 Score: 109 Matches: 3(1) Sequences: 3(1)

unknown [*Zea mays*]

Matched peptides shown in **bold red**.

1 MYRAAGSHLR SLKHHGASRL ASTSVAKQSS EPSKTKVTTL PNGVKIASET

51 SSSPAASVGL YIDCGSIYET PASSGVSHLL ERMAFKSTVN RTHLRLVREV

101 EAIGGNVSAS ASREQMSYTY DALKSYTPEM VEVLIDSVRN PAFLDWEVKE

151 QLQNIKSEIA DASANPQGLL LEALHSVGYS GALAKPLMAS ESAVNRLDVS

201 SLEEFVAEHY TAPRMVLAAS GVDHDALISV VEPLLSDLPC VKRPEEPKSV

251 YVGGDYRCQA DSPNTHIALA FEVPGGWNQE KTAMVVTVLQ MLMGGGGSFS

301 AGGPGKGMHS RLYLRVLTNF QQIESFSAFN SVYNNSGLFG IYAVTSPDFS

351 SKAVDLAAGE LLEIATPGKV TQEQLDRAKE ATKSAVLMNL ESRSIASEDI

401 GRQVLTYGER KPIEYFLKTV EEITLNDILS TAKEMMSTPL TMASWGDVIH

451 VPSYESVSRK FHSK

Query	Observed	Mr(expt)	Mr(calc)	ppm	Miss	Score	Expect	Rank	Unique	Peptide
3	965.5477	964.5404	964.4978	44.2	0	28	5	1		R.QVLTYGER.K
9	1218.6558	1217.6485	1217.6081	33.2	0	56	0.009	1		R.NPAFLDWEVK.E
14	1446.7951	1445.7878	1445.7110	53.1	0	25	8.6	2	U	R.EVEAIGGNVSASASR.

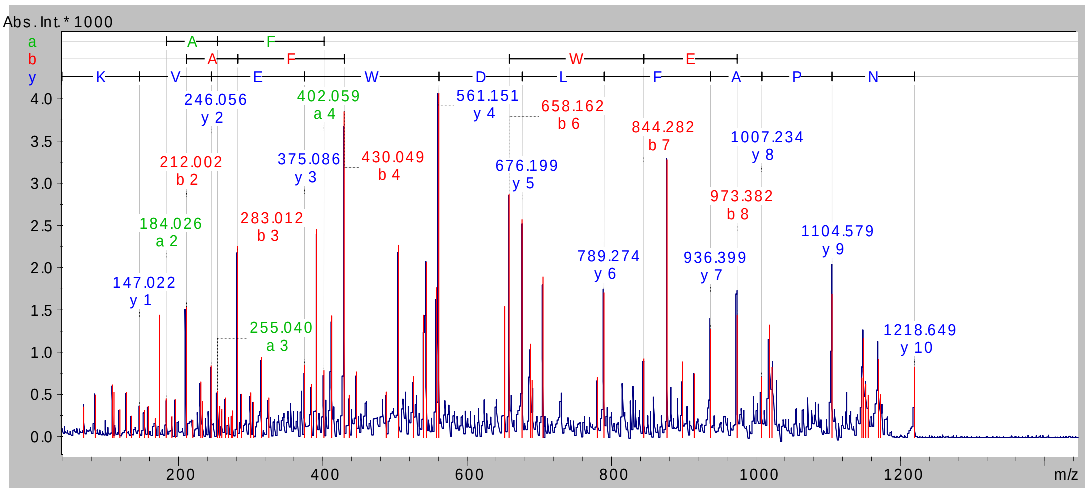

Proteins matching the same set of peptides:

gi|226498098 Mass: 53831 Score: 109 Matches: 3(1) Sequences: 3(1)

uncharacterized protein LOC100280280 [*Zea mays*]

gi|413946162 Mass: 53859 Score: 109 Matches: 3(1) Sequences: 3(1)

putative mitochondrial processing peptidase alpha subunit family protein [*Zea mays*]

gi|514747737 Mass: 53889 Score: 109 Matches: 3(1) Sequences: 3(1)

PREDICTED: mitochondrial-processing peptidase subunit alpha-like [*Setaria italica*]

Database 1

Match to: Unigene32364_Se200S transcribed RNA sequence Mass: 31677 Score: 259 Matches: 5(4) Sequences: 4(4)

gi| 225445041 PREDICTED: mitochondrial-processing peptidase subunit alpha [*Vitis vinifera*]

Matched peptides shown in **bold red**.

AQNHLTSSLNFLIFSSINQSIMYRVASSRLRALKKAREGSSCLARYASSSAVAAKANVSGGFLSSLFGGGSKSLPPLDFPLEGVTV

PPPLPDYVEPAKTKITTLPNGVKVASQASPNPVASIGLYVDSGSIYETPFSFGATHLLERMAFKTTTNRSHLRLVREVEAIGGNVT

ASASR**EQMGYTFDALKTYVPEMVELLVDSVRNPAFLDWEVK**ETLEKLKHEINEASNNPQGLLVEAIHSAGYSGALSNSLMAQ

EATISR**LDSSVLEQFVADNYTAPR**MVLAASGVEHEDLLSIA

Query	Observed	Mr(expt)	Mr(calc)	ppm	Miss	Score	Expect	Rank	Unique	Peptide
9	1218.6558	1217.6485	1217.6081	33.2	0	56	0.00018	1	U	R.NPAFLDWEVK.E
11	1318.6401	1317.6329	1317.5911	31.7	0	38	0.0094	1	U	R.EQMGYTFDALK.T + Oxidation (M)
18	1749.9728	1748.9655	1748.9019	36.4	0	(10)	3.4	2	U	K.TYVPEMVELLVDSVR.N
19	1765.9666	1764.9593	1764.8968	35.4	0	41	0.0028	1	U	K.TYVPEMVELLVDSVR.N + Oxidation (M)
20	2025.0590	2024.0517	2023.9851	32.9	0	124	1.4e-011	1	U	R.LDSSVLEQFVADNYTAPR.M

Database 2

Match to: Unigene41649_SALfmcTARAAPEI-3 Mass: 14289 Score: 259 Matches: 6(5) Sequences: 4(4)

gi| 225445041 PREDICTED: mitochondrial-processing peptidase subunit alpha [*Vitis vinifera*]

Matched peptides shown in **bold red**.

LVREVEAIGGNVTASASR**EQMGYTFDALKTYVPEMVELLVDSVRNPAFLDWEVK**ETLEKLKHEINEASNNPQGLLVEAIHSAG

YSGALSNSLMAQEATISR**LDSSVLEQFVADNYTAPR**MVLAASGVEHED

Query	Observed	Mr(expt)	Mr(calc)	ppm	Miss	Score	Expect	Rank	Unique	Peptide
9	1218.6558	1217.6485	1217.6081	33.2	0	56	8.7e-005	1	U	R.NPAFLDWEVK.E
10	1302.6899	1301.6826	1301.5962	66.4	0	(33)	0.015	1	U	R.EQMGYTFDALK.T
11	1318.6401	1317.6329	1317.5911	31.7	0	38	0.0049	1	U	R.EQMGYTFDALK.T + Oxidation (M)
18	1749.9728	1748.9655	1748.9019	36.4	0	(10)	1.9	2	U	K.TYVPEMVELLVDSVR.N
19	1765.9666	1764.9593	1764.8968	35.4	0	41	0.0015	1	U	K.TYVPEMVELLVDSVR.N + Oxidation (M)
20	2025.0590	2024.0517	2023.9851	32.9	0	124	5.7e-012	1	U	R.LDSSVLEQFVADNYTAPR.M

2. Unigene29638_SALfmcTARAAPEI-3 Mass: 9243 Score: 81 Matches: 3(2) Sequences: 3(2)

gi| 641836914 hypothetical protein CISIN_1g0104472mg, partial [*Citrus sinensis*]

Query	Observed	Mr(expt)	Mr(calc)	ppm	Miss	Score	Expect	Rank	Unique	Peptide
3	965.5477	964.5404	964.4978	44.2	0	28	0.042	1	U	R.QVLTYGER.K
4	1023.6182	1022.6109	1022.5800	30.2	0	39	0.0023	1	U	R.KPVEYFLK.T

6	1149.6460	1148.6387	1148.5859	45.9	0	14	1.1	2	U	K.SAILMNLESR.M + Oxidation (M)

Spot 920

NCBInr protein database

gi| 225445041 Mass: 54552 Score: 187 Matches: 5(3) Sequences: 3(2)

PREDICTED: mitochondrial-processing peptidase subunit alpha [*Vitis vinifera*]

Matched peptides shown in bold red.

1 MYRTAASRVR ALKGRAGSRA LIRFASSSAV ATSSSSSGGL FSWLIGDKSK

51 TLPPLDFPLP NVALPPALPD YVEPSKVKIT TISNGVKIAS ETSANPAASI

101 GLYVDCGSIY ETPISFGATH LLERMAFKST INRSYLRVIR **EVEAIGGNVT**

151 **ASASREQMGY TFDALK**TYVP EMVELLIDSV RNPAFLDWEV SEQLEKVKAE

201 IGEASNNPQG LLLEALHSAG YSGALANPLL APESAINRLD STILEEFVAL

251 NYTAPRMVLA ASGVEHEELL SVAEPLLSDL PSVPRPEEPK SVYVGGDYRC

301 QADSGKTHFA LAFEVPGGWH KEKEAMTLTV LQMLMGGGGS FSAGGPGKGM

351 YSRLYLRVLN TYPQIQSFSA FNSIYNNTGL FGIQATTGSD FVSKAIDIAA

401 KELVAVATPG QVDQVQLDRA KQTTK**TAVLM NLESR**MVASE DIGRQILTYG

451 ERKPVDHFLK AVDEVTLKDI ASITQKLLSS PLTMASYGDV IFVPSYENVS

501 SKFQSK

Query	Observed	Mr(expt)	Mr(calc)	ppm	Miss	Score	Expect	Rank	Unique	Peptide
6	1133.5858	1132.5785	1132.5910	-11.04	0	(30)	3.9	1	U	K.TAVLMNLESR.M
7	1149.5824	1148.5751	1148.5859	-9.39	0	34	1.2	2	U	K.TAVLMNLESR.M + Oxidation (M)
9	1302.5980	1301.5907	1301.5962	-4.17	0	(71)	0.00022	1		R.EQMGYTFDALK.T
10	1318.5941	1317.5869	1317.5911	-3.22	0	73	0.00013	1		R.EQMGYTFDALK.T + Oxidation (M)
12	1460.7248	1459.7176	1459.7267	-6.24	0	80	3e-05	1	U	R.EVEAIGGNVTASASR.E

2. gi|224035589 Mass: 50276 Score: 182 Matches: 3(2) Sequences: 3(2)

unknown [*Zea mays*]

Query	Observed	Mr(expt)	Mr(calc)	ppm	Miss	Score	Expect	Rank	Unique	Peptide
2	965.4902	964.4829	964.4978	-15.45	0	49	0.045	1		R.QVLTYGER.K
8	1218.6104	1217.6031	1217.6081	-4.06	0	92	2e-06	1		R.NPAFLDWEVK.E
11	1446.7038	1445.6966	1445.7110	-10.00	0	41	0.25	1	U	R.EVEAIGGNVSASASR.E

Proteins matching the same set of peptides:

gi|226498098 Mass: 53831 Score: 182 Matches: 3(2) Sequences: 3(2)

uncharacterized protein LOC100280280 [*Zea mays*]

gi|413946162 Mass: 53859 Score: 182 Matches: 3(2) Sequences: 3(2)

putative mitochondrial processing peptidase alpha subunit family protein [*Zea mays*]

gi|514747737 Mass: 53889 Score: 182 Matches: 3(2) Sequences: 3(2)

PREDICTED: mitochondrial-processing peptidase subunit alpha-like [*Setaria italica*]

3. gi|217075747 Mass: 54978 Score: 155 Matches: 5(3) Sequences: 3(2)

unknown [*Medicago truncatula*]

Query	Observed	Mr(expt)	Mr(calc)	ppm	Miss	Score	Expect	Rank	Unique	Peptide
2	965.4902	964.4829	964.4978	-15.45	0	49	0.045	1		R.QVLTYGER.K
6	1133.5858	1132.5785	1132.5910	-11.03	0	(29)	4.4	2		K.SAILMNLESR.M

7	1149.5824	1148.5751	1148.5859	-9.38	0	33	1.9	3		K.SAILMNLESR.M + Oxidation (M)
9	1302.5980	1301.5907	1301.5962	-4.17	0	(71)	0.00022	1		R.EQMGYTFDALK.T
10	1318.5941	1317.5869	1317.5911	-3.22	0	73	0.00013	1		R.EQMGYTFDALK.T + Oxidation (M)

Proteins matching the same set of peptides:

gi|357520795 Mass: 55092 Score: 155 Matches: 5(3) Sequences: 3(2)

Mitochondrial-processing peptidase subunit alpha [*Medicago truncatula*]

gi|593794457 Mass: 55098 Score: 155 Matches: 5(3) Sequences: 3(2)

hypothetical protein PHAVU_002G306900g [*Phaseolus vulgaris*]

Database 1

Match to: Unigene31944_SeCKS transcribed RNA sequence Mass: 25622 Score: 483 Matches: 8(8) Sequences: 6(6)

gi| 225445041 PREDICTED: mitochondrial-processing peptidase subunit alpha [*Vitis vinifera*]

AQNHLTSSLNFLIFSSINQSIMYRVASSRLRALKKAREGSSCLARYASSSAVVAKSNASGGFLSSLFGGGSKSLPPLDFPLEGVTVPPPLPDYVEPSKTKITTLPNGVKVASQASPNPVASIGLYVDSGSIYETPFSFGATHLLERMAFKTTTNRSHLRLVREVEAIGGNVTASASREQMGYTFDALKTYVPEMVELLVDSVRNPAFLDWEVKETLEKLKHEINEASNNPQGLLVEA

Query	Observed	Mr(expt)	Mr(calc)	ppm	Miss	Score	Expect	Rank	Unique	Peptide
8	1218.6104	1217.6031	1217.6081	-4.06	0	92	3.8e-008	1		R.NPAFLDWEVK.E
9	1302.5980	1301.5907	1301.5962	-4.17	0	(71)	2.7e-006	1		R.EQMGYTFDALK.T
10	1318.5941	1317.5869	1317.5911	-3.22	0	73	1.6e-006	1		R.EQMGYTFDALK.T + Oxidation (M)
12	1460.7248	1459.7176	1459.7267	-6.24	0	80	5.9e-007	1		R.EVEAIGGNVTASASR.E
14	1572.7510	1571.7437	1571.7580	-9.07	0	107	8.1e-010	1	U	K.SNASGGFLSSLFGGGSK.S
18	1749.8923	1748.8851	1748.9019	-9.61	0	(70)	5e-006	1		K.TYVPEMVELLVDSVR.N
19	1765.8915	1764.8842	1764.8968	-7.12	0	76	1.1e-006	1		K.TYVPEMVELLVDSVR.N + Oxidation (M)
30	2803.4284	2802.4211	2802.4732	-18.57	0	54	0.00011	1	U	K.SLPPLDFPLEGVTVPPPLPDYVEPSK.T

2. Unigene32364_Se200S transcribed RNA sequence Mass: 31677 Score: 479 Matches: 7(7) Sequences: 5(5)

PREDICTED: mitochondrial-processing peptidase subunit alpha [*Vitis vinifera*]

Query	Observed	Mr(expt)	Mr(calc)	ppm	Miss	Score	Expect	Rank	Unique	Peptide
8	1218.6104	1217.6031	1217.6081	-4.06	0	92	3.8e-008	1		R.NPAFLDWEVK.E
9	1302.5980	1301.5907	1301.5962	-4.17	0	(71)	2.7e-006	1		R.EQMGYTFDALK.T
10	1318.5941	1317.5869	1317.5911	-3.22	0	73	1.6e-006	1		R.EQMGYTFDALK.T + Oxidation (M)
12	1460.7248	1459.7176	1459.7267	-6.24	0	80	5.9e-007	1		R.EVEAIGGNVTASASR.E
18	1749.8923	1748.8851	1748.9019	-9.61	0	(70)	5e-006	1		K.TYVPEMVELLVDSVR.N
19	1765.8915	1764.8842	1764.8968	-7.12	0	76	1.1e-006	1		K.TYVPEMVELLVDSVR.N + Oxidation (M)
22	2024.9707	2023.9634	2023.9851	-10.71	0	157	8.3e-015	1	U	R.LDSSVLEQFVADNYTAPR.M

3. Unigene1186_SeCKS transcribed RNA sequence Mass: 24359 Score: 182 Matches: 6(4) Sequences: 5(4)

PREDICTED: mitochondrial-processing peptidase subunit alpha [*Vitis vinifera*]

Query	Observed	Mr(expt)	Mr(calc)	ppm	Miss	Score	Expect	Rank	Unique	Peptide
2	965.4902	964.4829	964.4978	-15.45	0	49	0.00088	1	U	R.QVLTYGER.K
3	993.4633	992.4560	992.4597	-3.66	0	14	1.9	2	U	R.MVASEDIGR.Q + Oxidation (M)
4	1023.5725	1022.5653	1022.5800	-14.45	0	54	0.00018	1	U	R.KPVEYFLK.T

6	1133.5858	1132.5785	1132.5910	-11.03	0	(29)	0.091	1	U	K.SAILMNLESR.M
7	1149.5824	1148.5751	1148.5859	-9.38	0	33	0.039	1	U	K.SAILMNLESR.M + Oxidation (M)
23	2038.1092	2037.1020	2037.1218	-9.76	0	32	0.017	1	U	K.ELIAVTRPGEVTQIQLDR.A

Proteins matching the same set of peptides:

Unigene15238_Se200S transcribed RNA sequence Mass: 25717 Score: 182 Matches: 6(4) Sequences: 5(4)

PREDICTED: mitochondrial-processing peptidase subunit alpha [*Vitis vinifera*]

Database 2

Match to: Unigene41649_SALfmcTARAAPEI-3 Mass: 14289 Score: 479 Matches: 7(7) Sequences: 5(5)

gi| 225445041 PREDICTED: mitochondrial-processing peptidase subunit alpha [*Vitis vinifera*]

LVREVEAIGGNVTASASREQMGYTFDALKTYVPEMVELLVDSVRNPAFLDWEVKETLEKLKHEINEASNNPQGLLVEAIHSAG

YSGALSNSLMAQEATISRLDSSVLEQFVADNYTAPRMVLAASGVEHED

Query	Observed	Mr(expt)	Mr(calc)	ppm	Miss	Score	Expect	Rank	Unique	Peptide
8	1218.6104	1217.6031	1217.6081	-4.06	0	92	1.9e-008	1	U	R.NPAFLDWEVK.E
9	1302.5980	1301.5907	1301.5962	-4.17	0	(71)	2.1e-006	1	U	R.EQMGYTFDALK.T
10	1318.5941	1317.5869	1317.5911	-3.22	0	73	1.2e-006	1	U	R.EQMGYTFDALK.T + Oxidation (M)
12	1460.7248	1459.7176	1459.7267	-6.24	0	80	2.7e-007	1	U	R.EVEAIGGNVTASASR.E
18	1749.8923	1748.8851	1748.9019	-9.61	0	(70)	2e-006	1	U	K.TYVPEMVELLVDSVR.N
19	1765.8915	1764.8842	1764.8968	-7.12	0	76	5e-007	1	U	K.TYVPEMVELLVDSVR.N + Oxidation (M)
22	2024.9707	2023.9634	2023.9851	-10.71	0	157	3.3e-015	1	U	R.LDSSVLEQFVADNYTAPR.M

2. Unigene42446_SALfmcTARAAPEI-3 Mass: 10029 Score: 162 Matches: 2(2) Sequences: 2(2)

gi| 661893420 unnamed protein product [*Coffea canephora*]

Query	Observed	Mr(expt)	Mr(calc)	ppm	Miss	Score	Expect	Rank	Unique	Peptide
14	1572.7510	1571.7437	1571.7580	-9.07	0	107	4.4e-010	1	U	K.SNASGGFLSSLFGGGSK.S
30	2803.4284	2802.4211	2802.4732	-18.57	0	54	4.7e-005	1	U	K.SLPPLDFPLEGVTVPPPLPDYVEPSK.S

3. Unigene29638_SALfmcTARAAPEI-3 Mass: 9243 Score: 150 Matches: 5(4) Sequences: 4(3)

gi| 641836914 hypothetical protein CISIN_1g0104472mg, partial [*Citrus sinensis*]

Query	Observed	Mr(expt)	Mr(calc)	ppm	Miss	Score	Expect	Rank	Unique	Peptide
2	965.4902	964.4829	964.4978	-15.45	0	49	0.0004	1	U	R.QVLTYGER.K
3	993.4633	992.4560	992.4597	-3.66	0	14	1.2	2	U	R.MVASEDIGR.Q + Oxidation (M)
4	1023.5725	1022.5653	1022.5800	-14.45	0	54	9.9e-005	1	U	R.KPVEYFLK.T
6	1133.5858	1132.5785	1132.5910	-11.03	0	(29)	0.041	1	U	K.SAILMNLESR.M
7	1149.5824	1148.5751	1148.5859	-9.38	0	33	0.019	1	U	K.SAILMNLESR.M + Oxidation (M)

Spot 1752

Database 2

Match to: Unigene40287_SALfmcTARAAPEI-3 Mass: 12199 Score: 156 Matches: 1(1) Sequences: 1(1)

gi| 566211115 trypsin protein inhibitor 3 [*Populus trichocarpa*]

VYDVYGEPLVAGGEYYIFPYDNKPIGGGFTWKKQRKSLSICPEASVNQVTDNLEYGEPAAIYPAERRSGNQITVSTDVNIMFPE

VSPPSGLPILCDSFSNIWRVENGKK

Query	Observed	Mr(expt)	Mr(calc)	ppm	Miss	Score	Expect	Rank	Unique	Peptide

37	3293.5887	3292.5814	3292.5557	7.79	0	156	2.8e-015	1	U

K.SLSICPEASVNQVTDNLEYGEPAAIYPAER.R

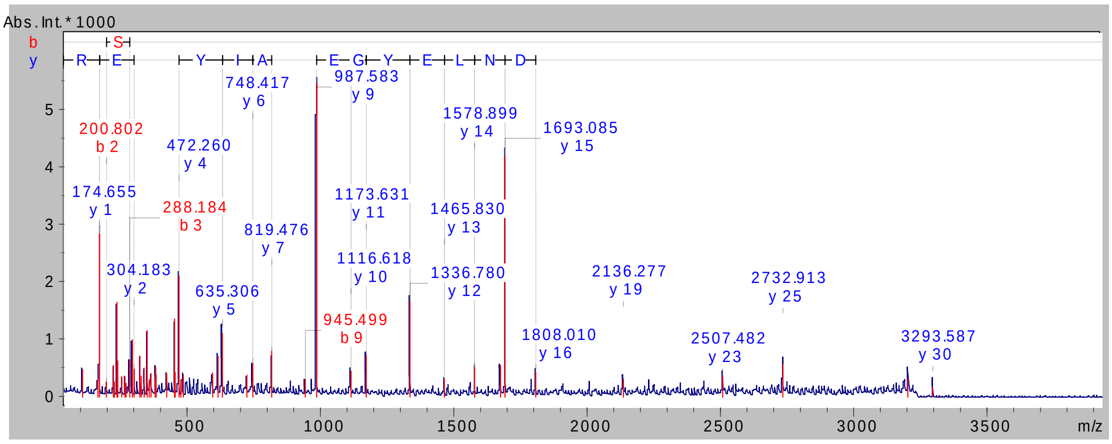

Figure S4.

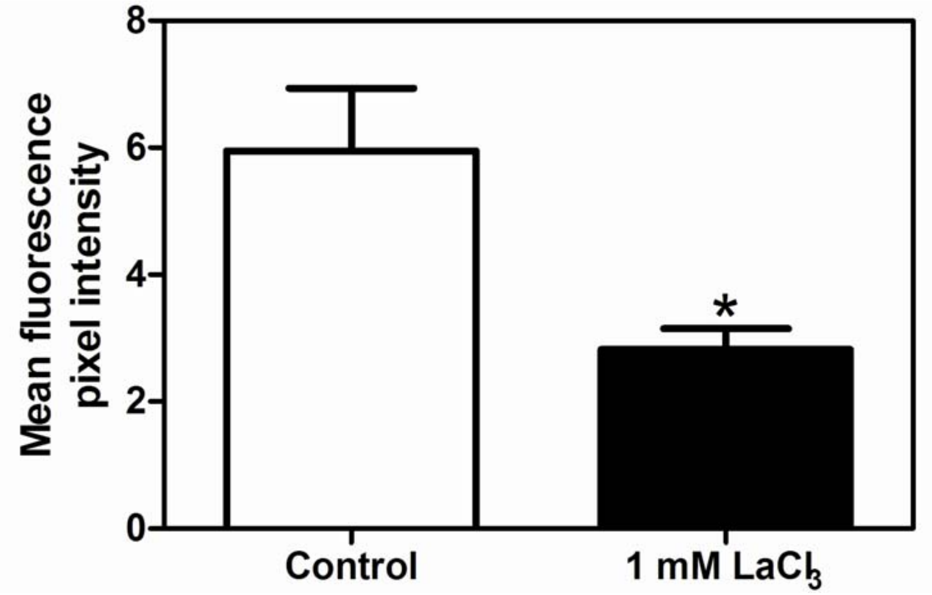

Mean fluorescence pixel intensity
8
6
4
2
0
*
Control
1 mM LaCl3
